# Heterocycle‐Tethered Aurones: Synthesis and Evaluation of *α*‐Glucosidase Inhibition

**DOI:** 10.1155/bmri/5193886

**Published:** 2026-04-17

**Authors:** Oleksandr L. Kobzar, Antonina V. Popova, Svitlana P. Bondarenko, Galyna P. Mrug, Andriy I. Vovk, Mykhaylo S. Frasinyuk

**Affiliations:** ^1^ Department of Bioorganic Mechanisms, V. P. Kukhar Institute of Bioorganic Chemistry and Petrochemistry of the NAS of Ukraine, Kyiv, Ukraine; ^2^ Department of Chemistry of Bioactive Nitrogen-Containing Heterocyclic Bases, V. P. Kukhar Institute of Bioorganic Chemistry and Petrochemistry of the NAS of Ukraine, Kyiv, Ukraine; ^3^ Selvita Services, Krakow, Poland; ^4^ Department of Food Chemistry, National University of Food Technologies, Kyiv, Ukraine, nuft.edu.ua; ^5^ Enamine Ltd., Kyiv, Ukraine

**Keywords:** *α*-glucosidase, aurone, chromone, hybrid compounds, inhibition, nitrogen-containing heterocycles

## Abstract

A wide range of heterocycle‐tethered aurones were synthesized using an efficient approach for the capture of in situ generated aurone‐based *ortho*‐quinone methide intermediates by 3‐(2‐hydroxyphenyl)enaminones and further ring‐opening and ring‐closure reaction with bidentate nucleophiles. The synthesized compounds were investigated in vitro as inhibitors of *Saccharomyces cerevisiae α*‐glucosidase. The activity of the methylene‐linked heterocycle‐aurone hybrids with substituted pyrazole or isoxazole moieties was found to be similar to that of chromone‐aurones and significantly exceeded the activity of corresponding aurones with pyrimidine or pyrazolo[1,5‐*a*]pyrimidine fragments. The azole‐aurone compounds can exhibit high inhibitory potency towards *α*‐glucosidase with IC_50_ values ranging from 3.0 ± 0.8 to 23.2 ± 5.1 * μ*M, being much more active than acarbose with an IC_50_ value of 760 ± 120 * μ*M. According to the results of kinetic studies, these compounds were found to be mixed‐type inhibitors, affecting enzyme activity through multiple binding sites, depending on the type of heterocycle. Given the ability of the azole‐aurone hybrids to inhibit *α*‐glucosidase in a parabolic mixed‐type manner, the values of apparent inhibition constants were calculated. The molecular docking simulations revealed that, in addition to the dominant role of the aurone moiety, the substituted heterocyclic part also contributes to the stabilization of the inhibitor within the enzyme active site.

## 1. Introduction

Type 2 diabetes mellitus (T2DM) is a persistent acquired metabolic disorder marked by dysregulated glucose homeostasis, which poses a significant health challenge worldwide. Medications approved as first‐line drugs for T2DM treatment can lower blood glucose levels, reduce the degree of insulin resistance, or improve insulin secretion [1]. One of the therapeutic targets for T2DM is *α*‐glucosidase, the small intestine brush border enzyme playing an important role in the hydrolysis of oligo‐ and polysaccharides to release *α*‐glucose in the intestinal lumen. The inhibition of this enzyme reduces postprandial hyperglycemia. The existing market drugs targeting *α*‐glucosidase, namely, acarbose, miglitol, and voglibose, have been approved since the 1990s. It should be noted that pyrazole[2], isoxazole[3], pyrimidine[4, 5], and pyrazolo [1,5‐*a*]pyrimidine[6] derivatives are promising compounds for the development of antidiabetic drugs.

**Figure 1:**
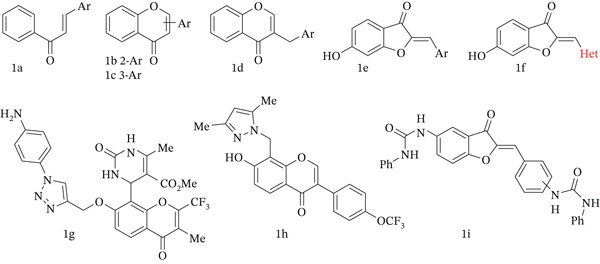
Subclasses of flavonoids: (a) chalcone, (b) flavone, (c) isoflavone, (d) homoisoflavonoid, (e) aurones, (f) heterocycle‐based aurone, and (g–i) representative *α*‐glucosidase flavonoid inhibitors.

The use of herbs in the treatment of diabetes is a known practice, and many natural compounds isolated from plant sources can exhibit medicinal properties [7]. It may be noted that numerous plant extracts and their isolated compounds, as well as various synthetic and semisynthetic molecules, have been identified as *α*‐glucosidase inhibitors (Figure 1) [8, 9]. Therefore, the design of effective *α*‐glucosidase inhibitors based on the structure of natural compounds is considered a promising direction of research in medicinal chemistry. Aurones, being a distinct and minor subclass of flavonoids, represent the least studied yet potentially promising scaffolds for designing compounds targeting this enzyme. Licoagroaurone 6‐*O*‐*α*‐L‐arabinopyranoside and its 6‐*O*‐methylated aglycone, coryaurone A, as well as Altilisin H, I, and J, all isolated from the plants, demonstrated inhibitory activity with IC_50_ values from the low to high micromolar range [10–13]. A similar inhibitory potential against *α*‐glucosidase was observed in the case of synthetic 6‐hydroxyaurone and arylureidoaurone derivatives [14, 15]. Thus, the natural aurones and their synthetic derivatives may be considered as drug candidates with antidiabetic activity [16, 17].

Aurones (2‐benzylidenebenzofuran‐3(2*H*)‐ones), a small subclass of flavonoids, are identified as one of the privileged structures in medicinal chemistry and are commonly associated with various pharmacologically active compounds [18–21]. Aurones are structurally isomeric to flavones and isoflavones with an exocyclic double bond that allows the formation of two isomeric forms (Figure 1). The thermodynamically more stable (*Z*)‐configuration is preferred for most synthetic and naturally occurring aurones [22]. Similarly to biosynthesis of aurones which involves the chalcone oxidative cyclization catalyzed by aureusidin synthase [23], the chemical route for obtaining aurones employs chalcones or their heteroaryl analogues as crucial intermediates for the oxidative cyclization using different reagents and catalysts [18, 24]. Another method for the synthesis of aurones is aldol condensation of benzofuran‐3(2*H*) ones with aryl/heteroaryl aldehydes in the presence of acidic or basic reagents. In contrast to nitrogen‐containing naturally occurring benzopyrones (flavoalkaloids), there is no data about the isolation of related nitrogen‐containing aurones from plants. At the same time, the known structural analogues and derivatives of aurones with remarkable bioactivity are described [18]. For example, among the 2‐heteroarylinene‐benzofuran‐3(2*H*)‐ones, pyrazole‐ [25–27], indol‐ [28–30], 7‐azaindol‐ [31], and pyridine‐based [29] aurones exhibit anticancer activity, indazol‐based aurones were identified as PIM1 inhibitors, [32, 33] and nitrofuran‐ [34] and nitroimidazole‐based [35] aurones were active against a wide spectrum of Gram‐positive and Gram‐negative bacteria. The aurone derivatives with 1,2,3‐triazolyl fragment attached to Position 6 can inhibit cathepsin B and show cytotoxic effects on the adenocarcinoma gastric cell line [36, 37]. The 7‐(1‐methylpiperidin‐4‐yl)‐substituted aurones mimicking the structure of flavopiridol, a clinically important synthetic flavoalkaloid, [38] effectively inhibit CDK1 and CDK2 [39]. However, nitrogen‐containing heterocycles linked to Position 7 of the aurone core are less represented in the literature.

In this context, we developed the synthesis of new methylene‐linked chromone‐aurone hybrids for obtaining the heterocycle‐tethered aurones with pyrazole, isoxazole, pyrimidine, or pyrazolo [1,5‐*a*] pyrimidine moieties at Position 7. The structure of the starting compounds was similar to that of coumarin‐aurone hybrids, which can inhibit *α*‐glucosidase and show glucose consumption‐promoting activity as possible antidiabetic agents [40].

Previously, the methylene‐linked azole‐benzopyrone hybrids were developed as promising *α*‐glucosidase inhibitors [41, 42]. In the present paper, the new approach to the synthesis of the heterocycle‐tethered aurones with pyrazole, isoxazole, pyrimidine, or pyrazolo [1,5‐*a*] pyrimidine moieties is reported. Further in vitro evaluation of the compounds as inhibitors of *α*‐glucosidase was carried out to analyze the relationship between their structure and activity.

## 2. Methods and Materials

Dichloromethane was purified by distillation. All solvents used in the syntheses were supplied by Enamine Ltd. *p*‐Nitrophenyl‐*α*‐D‐glucopyranoside (*p*‐NPG) and dimethyl sulfoxide were purchased from Sigma‐Aldrich. Sodium phosphate dibasic dodecahydrate and potassium dihydrogen phosphate obtained from other commercial suppliers were analytically pure. Purity of dimethyl sulfoxide was 99.9%. It was shown in additional experiments that the compounds studied did not affect the spectrophotometric determination of the enzyme activity.

^1^H, ^13^C, and ^19^F NMR spectra were measured on Bruker AVANCE DRX 500 (500/125/470 MHz) or AVANCE III 400 (400/100/376 MHz) spectrometers in CDCl_3_, referenced to residual CHCl_3_ (*δ*_H_ 7.26 ppm) or CDCl_3_ (*δ*_C_ 77.16 ppm), or DMSO‐*d*_6_, referenced to residual SO(CD_3_)(CD_2_H) (*δ*_H_ 2.50 ppm) or SO(CD_3_)_2_ (*δ*_C_ 39.52 ppm). Melting point values were recorded in open capillary tubes on a Büchi B‐535 apparatus (uncorrected). IR spectra were determined on a Bruker Vertex 70. Mass spectra were recorded on an Agilent 1100 LC‐MS system equipped with an APCI (atmospheric‐pressure chemical ionization) source. Elemental analysis was carried out on a Vario MICRO Cube CHNS analyzer. Column chromatography was performed on silica gel 60 (MACHEREY‐NAGEL, 0.04–0.063 mm).

### 2.1. Synthesis

#### 2.1.1. Synthesis of Aurone‐Based Homoisoflavonoids **5a**–**5i**

A mixture of corresponding aurone Mannich base **2a–c** [43] (2 mmol) and enaminone **4a–c** (2.2 mmol) was stirred in DMF (10 mL) at reflux for 4–6 h. After cooling, the reaction mixture was diluted with 30 mL of water and acidified with 1N HCl to pH 5–6. The resulting solid was filtered off, washed with water, and then purified by recrystallization from a mixture of DMF and MeOH (1:5).

#### 2.1.2. Synthesis of Pyrazole‐Tethered Aurones **6a**–**6g**

Hydrazine hydrate (0.5 mL) was added to a suspension of homoisoflavonoid **5** (1 mmol) in 10 mL of EtOH at 70°C. The mixture was stirred at reflux for 8  h, then cooled, diluted with 30 mL of water, and acidified with 1N HCl to pH 6–7. The resulting solid was filtered off, washed with water, and then purified by recrystallization from EtOH or by column chromatography using a mixture of CH_2_Cl_2_ and MeOH (20:1).

#### 2.1.3. Synthesis of Isoxazole‐Tethered Aurones **7a**–**7f**

DBU (4 mmol) was added to a suspension of homoisoflavonoid **5** (1 mmol) and hydroxylamine (2.2 mmol) in methyl cellosolve (5 mL) at 70°C. The mixture was stirred at reflux for 8 h and cooled. The solvent was evaporated, water (10 mL) was added, and the solution was acidified with 1N HCl to pH 6–7. The resulting solid was filtered off, washed with water, and then purified by recrystallization from EtOH or by column chromatography using a mixture of CH_2_Cl_2_ and MeOH (20:1).

#### 2.1.4. Synthesis of Pyrimidine‐Tethered Aurones **8a**–**8d**

DBU (4 mmol) was added to a suspension of homoisoflavonoid **5** (1 mmol) and guanidine hydroxylamine (2.2 mmol) in DMF (6 mL) at 70°C. The reaction mixture was stirred at 140°C–150°C for 8 h and cooled. The solvent was evaporated, water (10 mL) was added, and the solution was acidified with 1N HCl to pH 6–7. The resulting solid was filtered off, washed with water, and then purified by recrystallization from EtOH or by column chromatography using a mixture of CH_2_Cl_2_ and MeOH (10:1).

#### 2.1.5. Synthesis of Pyrimidine‐Tethered Aurones **9a**–**9d**

DBU (4 mmol) was added to a suspension of homoisoflavonoid **5** (1 mmol) and 3‐aminopyrazole (4 mmol) in DMF (6 mL) at 70°C. The mixture was stirred at reflux for 12 h and cooled. The solvent was evaporated, water (10 mL) was added, and the solution was acidified with 1N HCl to pH 6–7. The resulting solid was filtered off, washed with water, and then purified by column chromatography using a mixture of CH_2_Cl_2_ and MeOH (20:1).

Characteristics of the synthesized compounds and NMR spectra are provided in the Supporting Information.

### 2.2. *α*‐Glucosidase Inhibition

The synthesized compounds were studied in vitro as inhibitors of *α*‐glucosidase from *Saccharomyces cerevisiae* using *p*‐NPG as a substrate. The enzyme and substrate were purchased from Sigma‐Aldrich. All experiments to determine IC_50_ values were carried out in a reaction system (total volume of 2 mL) containing 57 mM phosphate buffer (pH 6.8), 0.85 mM substrate, 1% DMSO, the tested inhibitor, and *α*‐glucosidase. After incubating the mixture with an inhibitor and the enzyme at 37°C for 5 min, the reaction was initiated by the addition of *p*‐NPG. The control sample did not contain the inhibitor. The activity of *α*‐glucosidase was determined spectrophotometrically by monitoring the optical density of the reaction mixture at 400 nm over 10 min. Before the experiment, the compounds were dissolved in pure DMSO. The IC_50_ values were calculated from the semilogarithmic dependence of the remaining *α*‐glucosidase activity percentage against inhibitor concentration. The residual *α*‐glucosidase activity after inhibitor treatment was calculated as (*A*_sample_/*A*_control_) × 100.

### 2.3. Kinetics of the Enzyme Inhibition

The kinetics of *α*‐glucosidase inhibition by compounds **5c**, **6e**, and **7e** were studied under the same conditions as for the determination of the IC_50_ value. The only difference was the use of several concentrations of inhibitor (or without inhibitor in the control sample) at different concentrations of substrate. The dependence of velocity on substrate concentration obtained at different concentrations or without an inhibitor was used for the kinetics analysis. Apparent *K*_*m*_ and *V*_max_ values were determined by fitting the Michaelis–Menten equation to the experimental data of the saturation curves. The inhibition constants *K*_*i*_ and ${K_{i}}^{\prime }$ and parameters *n* and *n* ^′^ were calculated from dependences of *K*_*m*_/*V*_max_ versus [I] and 1/*V*_max_ versus [I], respectively. The calculations were performed using Python packages in a Jupyter Notebook within the Conda environment.

### 2.4. Molecular Docking Study

Compound **7e** was docked into the active site gorge of *α*‐glucosidase using AutoDock Vina software [44]. The model crystal structure of the *α*‐glucosidase MAL12 from *S. cerevisiae* (P53341) was downloaded from the AlphaFold Protein Structure Database (https://alphafold.ebi.ac.uk) [45]. The compound structure was drawn in MarvinSketch [46], optimized using MOPAC2016 [47] with the AM1 semiempirical quantum mechanical method, and saved in PDBQT format. Subsequently, the enzyme structure was loaded into AutoDockTools 1.5.6. [48] After adding hydrogen atoms and computing Gasteiger partial atomic charges, the structure was saved in PDBQT format. The ligand file in PDBQT format was loaded into AutoDockTools 1.5.6 and resaved while keeping partial atomic charges. Molecular docking calculations were performed using a grid box size set at 4 and grid centers of 10.792, 4.71, and −0.733.

## 3. Results

### 3.1. Synthesis of Heterocycle‐Tethered Aurones

**Figure 2:**
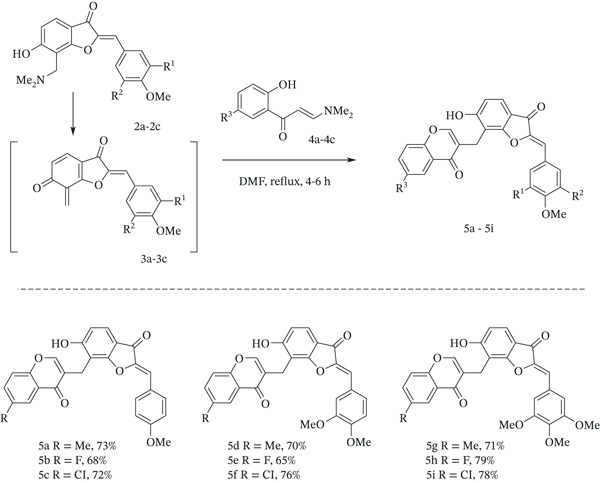
Products of the reaction of aurone Mannich bases with 3‐(2‐hydroxyphenyl)enaminones.

Oxidative cyclization of 2 ^′^‐methoxychalcones is a biosynthetic pathway for obtaining the structurally diverse homoisoflavonoids [49]. Their heterocycle‐based analogues were prepared using different chemical methods [50]. According to the recently developed procedure [51, 52], which includes the formation of *ortho*‐quinone methides and their trapping by 3‐(2‐hydroxyphenyl) enaminones, followed by a further cascade reaction, the synthesis of an aurone conjugated with a chromone ring was carried out as follows. 7‐Dimethylaminomethylaurones **2a**–**2c** were refluxed in DMF to afford aurone‐based *ortho*‐quinine methides **3a**–**3c**, which were trapped by enaminones **4a**–**4c** with subsequent formation of chromone‐aurone hybrids **5a**–**5i** in 65%–79% yield (Figure 2).

**Figure 3:**
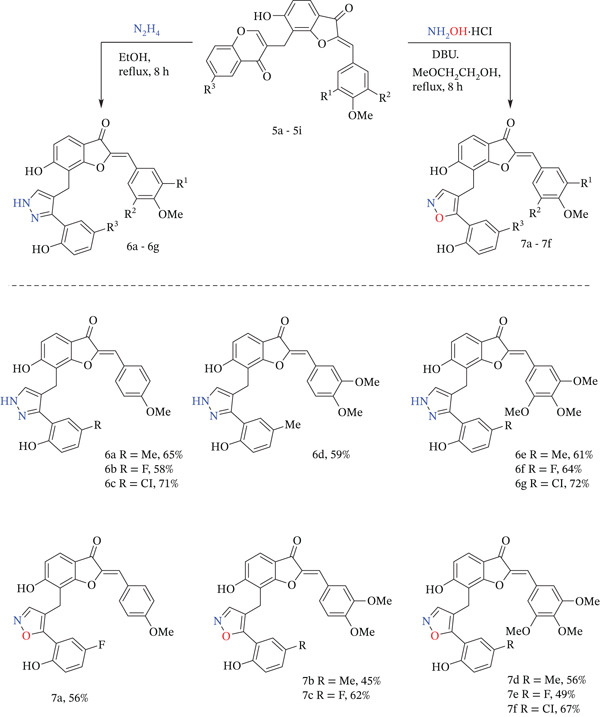
Substrate scope for the reaction of aurone‐based homoisoflavonoids with 1,2‐bidentate nucleophiles.

The reaction of compound **5** with excess hydrazine in EtOH resulted in the formation of aurone–pyrazole hybrids **6а**–**6g** with good yield (Figure 3). This approach could be a simple and effective route to obtain hydroxylated pyrazole‐tethered aurones. However, two bulky substituents at the pyrazole ring afforded a low rate of tautomerization in NMR scale experiments. As a result, the pyrazole ring protons were observed as one or two broad signals in ^1^H NMR spectra, with a concomitant disappearance of the carbon peaks in ^13^C NMR spectra. Adding CF_3_SO_3_H or CF_3_COOH to the solution of compounds **6а**–**6g** increased the tautomerization rate, and only one tautomeric form was observed with well‐resolved peaks in NMR spectra.

The direction of the chromone reaction with hydroxylamine and outcome products depends on substituents at Positions 2 and/or 3 of the chromone fragment. Terminated C‐2 chromones usually afford a mixture of isomeric 5‐(2‐hydroxyphenyl) isoxazoles and 3‐(2‐hydroxyphenyl)isoxazoles. [53, 54] However, using an appropriate solvent and organic base affords it possible to minimize side reactions, promoting the formation of one regioisomer. [55, 56] As we reported earlier for related coumarin‐based homoisoflavonoids, [52] the reaction of Compounds **5a**–**5i** with hydroxylamine with addition of DBU in methyl cellosolve allows to obtain of Compounds **7а**–**7f** at 125°C for 8 h with good yield (Figure 3).

**Figure 4:**
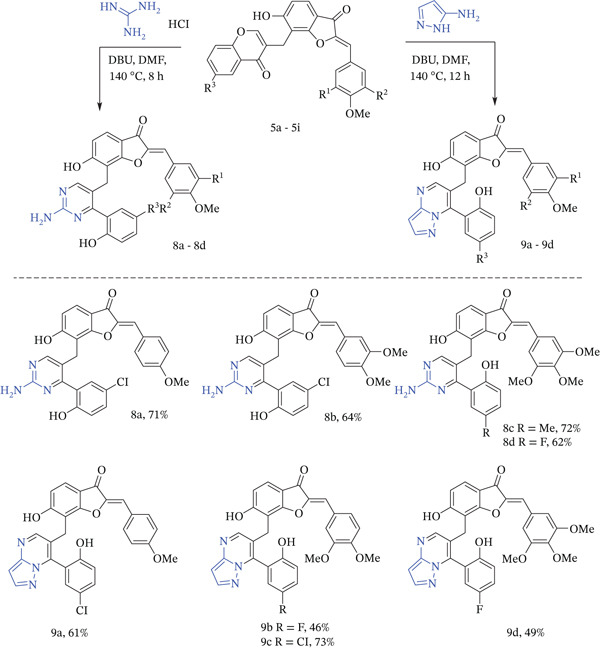
Substrate scope for the reaction of aurone‐based homoisoflavonoids with 1,3‐bidentate nucleophiles.

The next efforts were aimed at the synthesis of 8‐(2‐aminopyrimidin‐4‐yl)methyl‐3‐aurone derivatives obtained from the reaction of Compound **5** with guanidine hydrochloride. It turned out that this reaction of chromones with 1,3‐bidentate nucleophiles requires a strong base for the cascade transformations [52]. Thus, satisfactory yields of pyrimidine‐aurone hybrids were not obtained in ethanol using *N*‐morpholine or DBU. Meanwhile, good results were obtained when DBU was used in DMF. The best yields of Compounds **8a**–**8d** (Figure 4) were achieved by using homoisoflavonoids, guanidine hydrochloride, and DBU in a ratio of 1:2:3.

As reported earlier, 3(5)‐aminopyrazoles are efficient bidentate nucleophiles in reaction with chromone derivatives as masked 1,3‐dicarbonyl compounds in the presence of base. Despite the unsymmetrical nature of aminopyrazole, only 7‐(2‐hydroxyphenyl) pyrazolo [1,5‐*a*] pyrimidines were formed in the reaction of 3‐substituted benzopyran‐4‐one derivatives with 3‐aminopyrazole using MeOH [57–59] or EtOH [60] as a solvent. Involvement in the reaction of 3,4‐unsubstituted chromones led to formation of mixture 7‐(2‐hydroxyphenyl) pyrazolo [1,5‐*a*] pyrimidines and 5‐(2‐hydroxyphenyl) pyrazolo [1,5‐*a*]pyrimidines. [61] The best results for the reaction of chromone‐aurone hybrids **5** with 3‐aminopyrazole were obtained using DBU in DMF with the formation of Compounds **9a**–**9d** in 46%–73% yield (Figure 4). This can be explained by the initial attack of C‐2 chromone by the amino group of 3‐aminopyrazole with subsequent ring‐opening and ring‐closure stages.

The structure of the synthesized compounds was confirmed by NMR spectra. Thus, the peak of the CH_2_ group of compounds **5a–5i** was observed as a singlet at *δ*_H_ 3.84–3.90 ppm and as an intense signal at *δ*_c_ 8.5–18.7 ppm. The signal of the H‐2 proton of chromone was observed at *δ*_H_ 7.98–8.01 ppm. Shifts of CH_2_ singlets were at *δ*_H_ 3.91–3.99 ppm and *δ*_C_ 17.7–18.2 ppm, and the H‐3(5) proton of the pyrazole ring appeared at *δ*_H_ 7.67–8.01 ppm for the pyrazole‐aurone hybrids **6a–6g**. Notably, the signals of the H‐3 and CH_2_ group of the isoxazole ring did not significantly change compared with the starting compound **5**. Signals of the methylene group of compounds **7a–7f** were observed at *δ*_H_ 3.84–4.20 ppm and *δ*_C_ 16.8–17.2 ppm; the signal of the H‐3 proton of the isoxazole was at *δ*_H_ 7.79–8.13 ppm. In the NMR spectra of synthesized pyrimidines **8a–8d**, signals of the CH_2_ group were present at *δ*_H_ 3.80–3.83 ppm and slightly shifted to *δ*_C_ 22.2–22.6 ppm due to the electron‐withdrawing pyrimidine ring. The pyrimidine H‐6 signal was observed at *δ*_H_ 7.94–8.06 ppm. Signals of the CH_2_ group of pyrazolo[1,5‐*a*]pyrimidines **9a–9d** derivatives were observed as two doublets at *δ*_H_ 4.02–4.10 ppm with ^2^ *J* 15.1–15.8 Hz as a result of the chirality axis due to bulky *ortho*‐substituents. The position of their peat in ^13^C spectra is practically identical (*δ*_C_ 22.6–22.8 ppm). The signal of pyrazolo[1,5‐*a*]pyrimidine H‐5 was observed at *δ*_H_ 8.64–8.66 ppm.

### 3.2. Structure and Activity of Heterocycle‐Tethered Aurones as *α*‐Glucosidase Inhibitors

The synthesized compounds were investigated in vitro as inhibitors of *α*‐glucosidase from *S. cerevisiae*. The inhibition of the yeast enzyme is considered an approach to search for inhibitors of human *α*‐glucosidase catalyzed the hydrolytic release of *α*‐glucose from disaccharides and some other substrates in the mucosal brush border of the small intestine. [62] The IC_50_ values were determined from the dose‐response curves as the compound concentrations that reduce enzyme activity by 50%, using *p*‐NPG as a substrate (Figure 5).

Figure 5Dose‐dependent curves for *α*‐glucosidase inhibition by Compounds (a) **5c**, (b) **6e**, and (c) **7e**.

**Figure:**
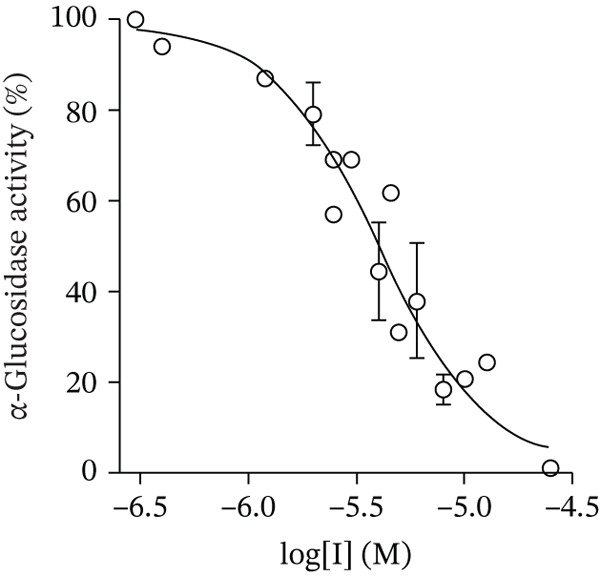
(a)

**Figure:**
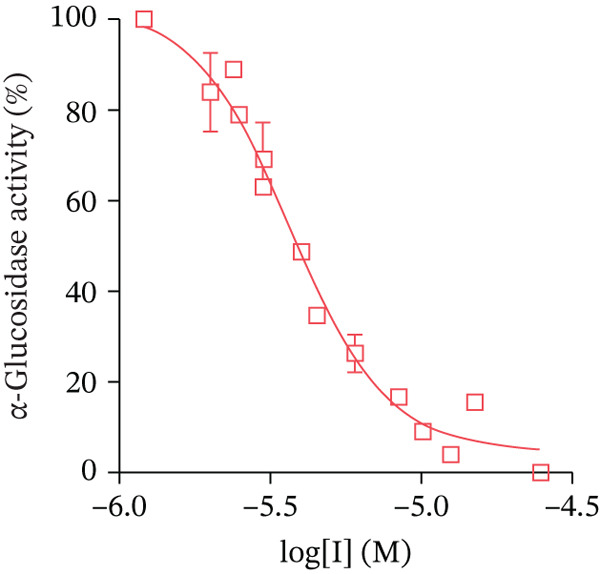
(b)

**Figure:**
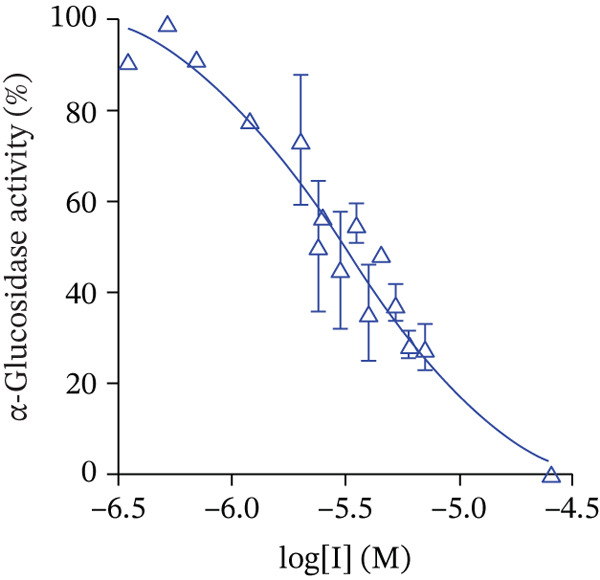
(c)

As seen from Table 1, the tested 7‐((chromen‐4‐on‐3‐yl)methyl) aurones are able to inhibit *α*‐glucosidase with IC_50_ values ranging from 4.0 to 28.4 *μ*M, which are similar to the inhibitory effects of 7‐coumarin‐substituted aurones [40] and approximately 25–200‐fold better than that obtained for acarbose. The analysis of structure and activity relationship revealed that compounds **5a**–**5c** bearing a single methoxy group at position 4 ^′^ of the B‐ring of the aurone scaffold were more effective inhibitors of the enzyme than derivatives **5d**–**5f** and **5** g‐**5i** with two or three methoxy groups, respectively. The inhibition was improved when the methyl group at position 6 of the chromen‐4‐one ring of the 3 ^′^,4 ^′^‐dimethoxy substituted derivative **5d** was replaced by a fluorine or chlorine atom (compound **5f**), while the inhibitory activity of derivatives **5b** and **5c** remained the same as compared to compound **5a**.

**Table 1:** In vitro inhibitory properties of novel heterocycle‐tethered aurones against *α*‐glucosidase.

Compound	IC_50_, *μ*M^a^
**5a**	4.4 ± 1.0
**5b**	4.7 ± 1.1
**5c**	4.0 ± 1.1
**5d**	20.2 ± 3.8
**5e**	18.1 ± 2.1
**5f**	8.1 ± 2.1
**5g**	28.4 ± 3.6
**5h**	21.7 ± 1.9
**5i**	18.5 ± 1.7
**6a**	8.0 ± 1.9
**6b**	6.7 ± 1.4
**6c**	23.2 ± 5.1
**6d**	8.1 ± 0.4
**6e**	4.1 ± 0.5
**6f**	7.9 ± 1.9
**6g**	9.2 ± 0.9
**7a**	8.9 ± 1.9
**7b**	6.3 ± 1.2
**7c**	3.3 ± 0.4
**7d**	4.5 ± 1.0
**7e**	3.0 ± 0.8
**7f**	6.7 ± 1.8
**8a**	10.8 ± 2.1
**8b**	> 25
**8c**	> 25
**8d**	> 25
**9a**	15.7 ± 5.1
**9b**	> 25
**9c**	> 25
**9d**	> 25
Acarbose^b^	760 ± 120

^a^The data are shown as mean ± standard deviation.

^b^Reference compound.

Among the 7‐((3‐(2‐hydroxyphenyl)‐1*H*‐pyrazol‐4‐yl)methyl) aurones **6a**–**6g**, excepting compound **6c**, the IC_50_ values depended little on the number of methoxy groups on the B ring, as well as on the nature of the metasubstituent in the phenyl ring. The bioisosteric replacement of the pyrazole fragment with an isoxazole ring led to compounds **7a**–**7f**, expanding the series of compounds active against this enzyme. The most effective imidazole and isoxazole tethered *α*‐glucosidase inhibitors were compounds **6e**, **7c**, and **7e** with IC_50_ values of 4.1, 3.3, and 3.0 *μ*M, respectively.

Unexpectedly, 7‐((2‐amino‐4‐(2‐hydroxyphenyl)pyrimidin‐5‐yl)methyl) aurones **8a**–**8d** and 7‐((7‐(2‐hydroxyphenyl)pyrazolo[1,5‐*a*]pyrimidin‐6‐yl)methyl) aurones **9a**–**9c** were less effective *α*‐glucosidase inhibitors than the corresponding azole‐containing derivatives. Only monomethoxy‐substituted compounds **8a** and **9a** demonstrated some activity, whereas the IC_50_ values for the other tested compounds in these series turned out to be greater than 25 *μ*M.

### 3.3. Kinetic Features of *α*‐Glucosidase Inhibition by Aurone Derivatives

The kinetic experiments were undertaken with compounds **5c**, **6e**, and **7e** to comprehend the molecular mechanism of *α*‐glucosidase inhibition. Figure 6 demonstrates the Michaelis–Menten saturation curves for the enzymatic hydrolysis of *p*‐NPG to the D‐glucopyranoside and *p*‐nitrophenol in the absence and presence of the inhibitors. The Michaelis constants (*K*_*m*_) and the maximum rate of the enzyme‐catalyzed reaction (*V*_max_) derived from these plots showed that with increasing inhibitor concentration, *K*_*m*_ value increased, while the value of *V*_max_ decreased. These are consistent with a mixed‐type inhibition.

Figure 6Dependence of the rate of reaction catalyzed by *α*‐glucosidase on the substrate concentration in the absence and presence of (a) 2, 4, and 8 *μ*M Compound **5c**, (b) 2, 4, and 6 *μ*M Compound **6e**, and (c) 2, 3, and 4 *μ*M Compound **7e**.

**Figure:**
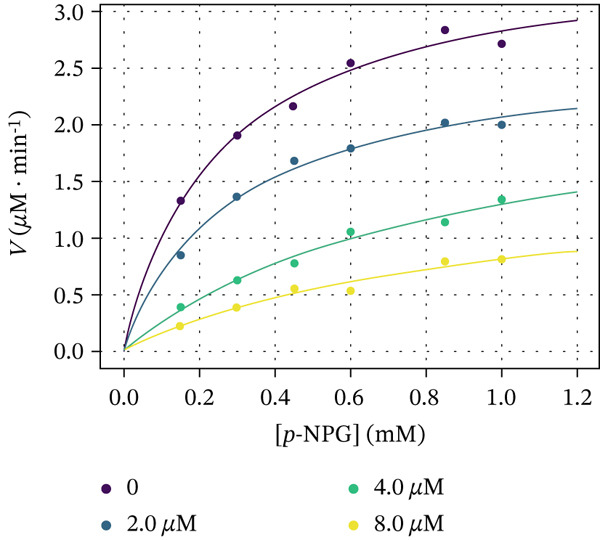
(a)

**Figure:**
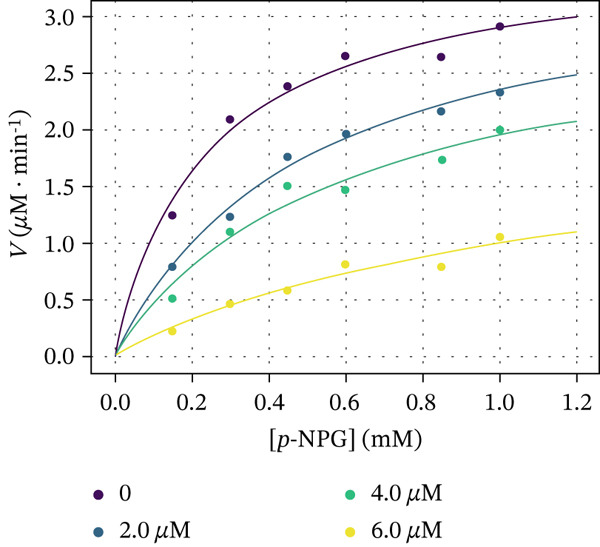
(b)

**Figure:**
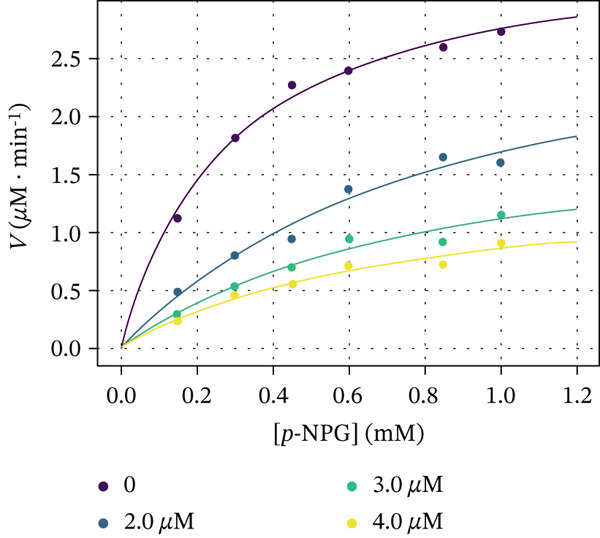
(c)

The secondary plots shown in Figure 7 demonstrate a nonlinear dependence of *K*_*m*_/*V*_max_ versus [I] for pyrazole‐containing compound **6e** and 1/*V*_max_ versus [I] for aurone derivatives **6e** and **7e**. In the case of inhibition by chromen‐4‐one‐containing derivative **5c**, such dependences seem to be close to linear. The parabolic character of the inhibition by compounds **6e** and **7e** suggests that more than one molecule of inhibitor may interact with free enzyme and enzyme–substrate complex. Using the Hill‐type model for such inhibition [63], the rate of enzymatic reaction in the presence of an inhibitor can be described by the following equation [64]:

V=VmaxSKm1+I/Kin+S1+I/Ki′n′

where *V*_max_ represents the maximum rate of an enzymatic reaction, [S] and [I] are substrate and inhibitor concentrations, *K*_*m*_ is the Michaelis constant, and *K*_*i*_ and ${K_{i}}^{\prime }$ are the dissociation constants of the enzyme–inhibitor complex and enzyme–substrate–inhibitor complex, respectively, in the mixed‐type inhibition. The coefficients *n* and *n* ^′^ are the number of inhibitor molecules that bind to the free enzyme and the enzyme–substrate complex, respectively. It is obvious that the inhibition constants *K*_*i*_ and ${K_{i}}^{\prime }$ are composite values representing dissociation constants of the complexes that might be formed in the previous steps.

Figure 7Secondary plots of *K*_*m*_/*V*_max_ versus [I] and 1/*V*_max_ versus [I] for *α*‐glucosidase inhibition by Compounds (a) **5c**, (b) **6e**, and (c) **7e**.

**Figure:**
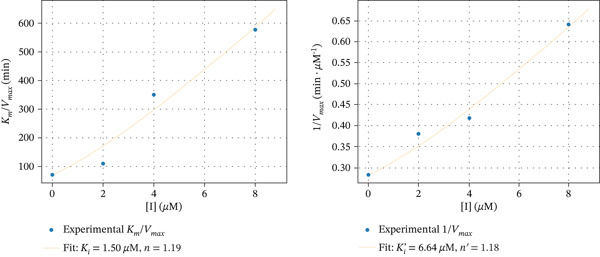
(a)

**Figure:**
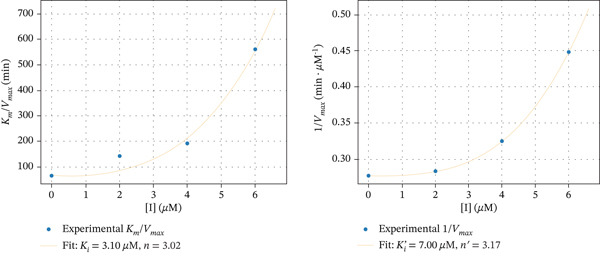
(b)

**Figure:**
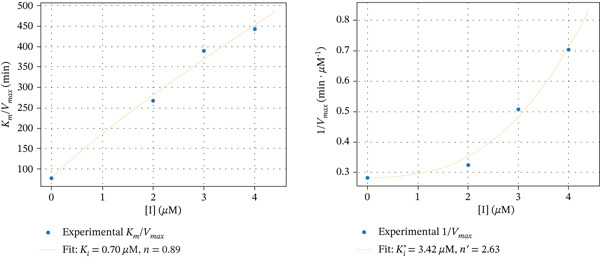
(c)

The calculated values of inhibition constants *K*_*i*_ and ${K_{i}}^{\prime }$ are 1.50 and 6.64 *μ*M for compound **5c**, 3.10 and 7.00 *μ*M for compound **6e**, and 0.70 and 3.42 *μ*M for compound **7e**. Coefficients *n* and *n* ^′^ are approximately 1.19 and 1.18 for compound **5c**, 3.02 and 3.17 for compound **6e**, and 0.89 and 2.63 for compound **7e**, respectively.

### 3.4. Molecular Docking Studies

To gain insight into the intermolecular interactions between the inhibitor and the enzyme, the isoxazole‐containing aurone **7e** was docked into the active site of the *α*‐glucosidase from *S. cerevisiae* (AlphaFold identifier AF‐P53341‐F1). The obtained docking model (Figure 8) showed that the ligand fits well in the active site gorge with a calculated affinity of −9.5 kcal/mol. Benzofuran‐3(2*H*)‐one fragment located in the substrate binding site has hydrophobic bonds with Phe157, Phe177, Thr215, Ala278, and Phe300. The 3 ^′^,4 ^′^,5 ^′^‐trimethoxyphenyl substituent at the C‐2 position of benzofuran‐3(2*H*)‐one moiety occupies the entrance to the active site, providing electrostatic and hydrophobic interactions with Phe157, His239, Asn241, Pro309, His279, and Glu304. Isoxazole ring forms a *π*‐cation interaction with the guanidinium group of Arg312. The 2‐hydroxy‐5‐fluorophenyl fragment of the inhibitor is located at the hydrophobic entrance of the active site, where it engages in an intramolecular *π*‐stacking interaction with the 3 ^′^,4 ^′^,5 ^′^‐trimethoxyphenyl substituent. This fragment also participates in hydrophobic, electrostatic, and van der Waals interactions with Phe157, Arg312, and Tyr313 and forms a hydrogen bond with Asp408.

Figure 8(a) Possible binding mode of Compound **7e** in the active site cavity of *α*‐glucosidase from *S. cerevisiae* and (b) amino acid residues surrounding the ligand.

**Figure:**
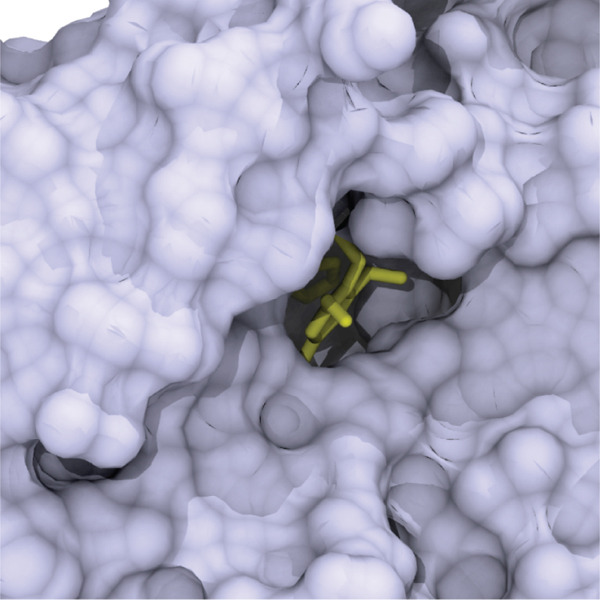
(a)

**Figure:**
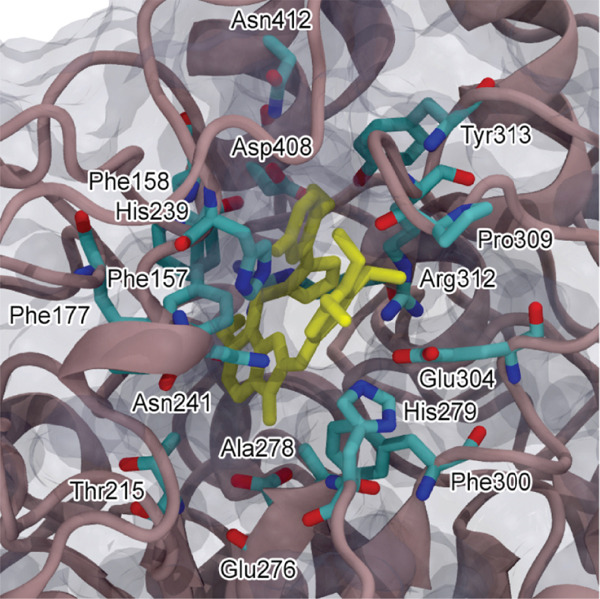
(b)

## 4. Discussion

Herein, we propose a new synthetic route to aurone derivatives with pyrazole, isoxazole, pyrimidine, or pyrazolo [1,5‐*a*] pyrimidine ring attached to Position 7 through a methylene link. The compounds were synthesized in a reaction of corresponding bidentate nucleophiles with an aurone and chromen‐4‐one hybrid. According to previous experience, [41] the aurone‐based homoisoflavonoids were obtained from 7‐dimetnylaminomethyl‐6‐hydroxyaurones using the thermally generated *ortho*‐quinone methides and their subsequent reaction with 3‐(dimethylamino)‐1‐(2‐hydroxyphenyl)prop‐2‐en‐1‐ones. All synthesized aurone derivatives were of interest as possible inhibitors of *α*‐glucosidase, considering the known effects on the activity of this enzyme of flavonoid hybrids [40] as well as triazoles and other heterocycles [66, 67]. The combination of two fragments, at least one of which has a natural skeleton, increases the biological relevance of such hybrid structures, since the aurone and other moiety connected through a methylene bridge can retain their ability to recognize a target protein [68].

Considering the activity against *α*‐glucosidase and the structural features of the synthesized compounds, we noted that the heterocyclic moiety and the number of methoxy substituents in ring B of the aurone part may be important for inhibition. At the same time, the replacement of the methyl group of the 2‐hydroxy‐5‐methylphenyl fragment on the fluorine or chlorine atom had virtually no effect on the inhibition efficiency of the hybrid molecule. According to IC_50_ values, the compounds studied can be divided into three groups. The tested 7‐((chromen‐4‐on‐3‐yl)methyl) aurones represent the first group. Among them, the monomethoxy‐substituted derivatives have the better values of IC_50_. The activity of azole‐containing hybrid compounds forming the second group depended a little on the number of methoxy groups, but in general, the 7‐((3‐(2‐hydroxyphenyl‐5‐substituted)‐1,2‐oxazol‐4‐yl)methyl) aurones had slightly better inhibitory potency than their 1*H*‐pyrazol‐4‐yl analogues. On the contrary, 7‐((2‐amino‐4‐(2‐hydroxyphenyl)pyrimidin‐5‐yl)methyl) aurones **8a**–**8d** and 7‐((7‐(2‐hydroxyphenyl)pyrazolo[1,5‐*a*]pyrimidin‐6‐yl)methyl) aurones **9a**–**9c** belonging to the third group demonstrated low activity against *α*‐glucosidase.

Given this, the aurone derivatives with chromen‐4‐one, pyrazole, and isoxazole moieties were chosen for kinetic studies. The results showed that these compounds affect both *K*_*m*_ and *V*_max_ values, which indicates that the inhibitor binds to two sites of *α*‐glucosidase, namely, to free enzyme and to the enzyme with bound substrate. The kinetic analysis showed that the replots of *K*_*m*_/*V*_max_ and 1/*V*_max_ (corresponding to the slope and the *y*‐intercept in Lineweaver–Burk) versus inhibitor concentration can be nonlinear. In this case, more than one molecule of inhibitor binds to one of the two sites or both sites. To estimate the contribution of a possible parabolic mechanism, the apparent values of *n* and *n* ^′^ for complexes EI^*n*^ and $\text{ES}{\text{I}}^{n^{\prime }}$, as well as the inhibition constants, were calculated. The data obtained indicate that the inhibitors bind to the free enzyme approximately 2–5 times more tightly than to the enzyme–substrate complex. The values of *n* and *n* ^′^ are close to 1 only in the case of compound **5с**, that is, one molecule of this inhibitor binds both to the free enzyme and the enzyme–substrate complex. It was interesting to observe that aurone derivatives with pyrazole and isoxazole moieties demonstrated differences in mechanisms of *α*‐glucosidase inhibition. Comparison of compounds **6e** and **7e** indicates the role of the heterocyclic substituent at position 7 of the aurone A‐ring. Although in both cases the enzyme–substrate complex is capable of tethering more than one inhibitor molecule, only one molecule of the isoxazole‐containing derivative forms a simple complex with the free enzyme. Thus, the oxazole derivative forms the EI complex and can form the ESI^2^ complex, apparently through the formation of an ESI intermediate. This highlights the peculiarities of the interaction between the enzyme and the azole‐containing aurone derivatives.

The docking results demonstrate the interactions of isoxazole‐containing aurone **7e** with amino acid residues of the AlphaFold model of *α*‐glucosidase from *S. cerevisiae*. Being fully located in the catalytic site, the compound **7e** makes bonds with the residues that are essential for catalysis and are involved in the binding of many inhibitors to *α*‐glucosidase [70]. Besides the dominant role of hydrophobic contacts of the aurone moiety, the isoxazole part of the inhibitor additionally forms a *π*‐cation interaction with the guanidinium group of Arg312, and the 2‐hydroxy‐5‐fluorophenyl group at the isoxazole ring has a hydrogen bond with Asp408. Thus, both the aurone moiety and the substituted isoxazole fragment of the hybrid molecule contribute to the stability of the complex formed during the interaction of the inhibitor with the active site region of the enzyme.

The SAR analysis of the heterocycle‐tethered aurones as *α*‐glucosidase inhibitors showed that the in vitro activity of these compounds is largely dependent on the nature of the heterocyclic part in their structure. The activity of the methylene‐linked heterocycle‐aurone hybrids with substituted pyrazole or isoxazole moieties was found to be similar to that of chromone‐aurone hybrids. It was further found that the activity of the methylene‐linked pyrazole‐ or isoxazole‐aurone hybrids, as well as aurones conjugated with chromones, significantly exceeded the activity of corresponding aurone derivatives with pyrimidine or pyrazolo [1,5‐*a*] pyrimidine fragments. Among the aurone derivatives bearing azole fragments, the isoxazole part may provide better inhibition of this enzyme than the pyrazole one.

## 5. Conclusion

In summary, we demonstrated that thermally generated *ortho*‐quinone methides from 7‐dimetnylaminomethyl‐6‐hydroxyaurones can be efficiently trapped with 3‐(dimethylamino)‐1‐(2‐hydroxyphenyl)prop‐2‐en‐1‐ones with formation of aurone‐based homoisoflavonoids. Further reaction of synthesized compounds with bidentate nucleophiles resulted in the formation of aza‐heterocycle‐tethered aurones. The obtained hybrid compounds were evaluated as inhibitors of *α*‐glucosidase. Among them, some of chromen‐4‐one‐, pyrazole‐, and isoxazole‐containing aurone derivatives demonstrated IC_50_ values in the micromolar range. Kinetic data indicated that the heterocyclic moiety of the aurone derivative can influence the mechanisms of inhibition. It may be suggested that the synthetic methodology and properties of heterocycle‐tethered aurones described here will be useful for designing new aurone‐based bioactive agents. These results could be a starting point for the investigation and development of different methylene‐linked flavonoid‐heterocycle hybrids as promising *α*‐glucosidase inhibitors.

## Author Contributions

Conceptualization: M.S.F., A.I.V., and O.L.K. Synthesis of compounds: A.V.P., G.P.M., and S.P.B. Biological assays: O.L.K. Writing—review and editing: M.S.F., O.L.K., and A.I.V.

## Funding

The study was funded by National Academy of Sciences of Ukraine (10.13039/501100004742, Project No. 0125U000355).

## Disclosure

Although the authors are affiliated with Enamine Ltd., Kyiv, Ukraine, and Selvita Services, Krakow, Poland, the companies had no role in the study design, data collection and analysis, decision to publish, or preparation of the manuscript. They had no impact on the outcome of the study.

## Conflicts of Interest

The authors declare no conflicts of interest.

## Supporting information


**Supporting Information** Additional supporting information can be found online in the Supporting Information section. Characterization data, copies of ^1^H, ^13^C NMR spectra, and LC‐MS spectra of synthesized compounds (PDF).

## Data Availability

The data that support the findings of this study are available in the Supporting Information of this article.
