# Supplementary material for: Heterocycle‐Tethered Aurones: Synthesis and Evaluation of α‐Glucosidase Inhibition
Source: Biomed Res Int. 2026 Apr 17;2026:5193886. doi: 10.1155/bmri/5193886 (PMC13089207; doi:10.1155/bmri/5193886)
Supplement: Supplementary file 1 — Supporting Information Additional supporting information can be found online in the Supporting Information section. Characterization data, copies of 1H, 13C NMR spectra, and LC‐MS spectra of synthesized compounds (PDF). [file BMRI-2026-5193886-s001.pdf]

Heterocycle-tethered aurones: synthesis and evaluation of α-glucosidase inhibition

Oleksandr L. Kobzar [a], Antonina V. Popova [b,c], Svitlana P. Bondarenko [d], Galyna P. Mrug [b], Andriy I. Vovk [a], and Mykhaylo S. Frasinyuk*[b,e]

[a] *Department of Bioorganic Mechanisms, V. P. Kukhar Institute of Bioorganic Chemistry and Petrochemistry of the NAS of Ukraine, Kyiv, Ukraine*
[b] *Department of Chemistry of Bioactive Nitrogen-Containing Heterocyclic Bases, V. P. Kukhar Institute of Bioorganic Chemistry and Petrochemistry of the NAS of Ukraine, Kyiv, Ukraine, E-mail: mykhaylo.frasinyuk@ukr.net*
[c] *Selvita Services, ul. Bobrzyńskiego 14, Krakow, 30-348, Poland*
[d] *Department of Food Chemistry, National University of Food Technologies, Kyiv, Ukraine*
[e] *Enamine Ltd., Kyiv, Ukraine*

Characterization of the synthesized compounds S2

NMR spectra of the synthesized compounds S10

LC-MS spectra of the synthesized compounds S81

3-{[(2Z)-6-Hydroxy-2-(4-methoxybenzylidene)-3-oxo-2,3-dihydro-1-benzofuran-7-yl]methyl}-6-methyl-4H-chromen-4-one (5a). Yellow solid; yield 643 mg (73%); mp 285-287°C; ^{1}H NMR

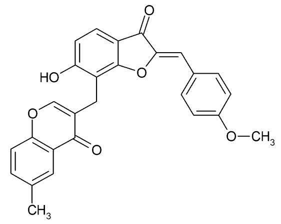

(400 MHz, DMSO-d_6): δ 11.19 (s, 1H), 8.12 (s, 1H), 7.92 – 7.85 (m, 1H), 7.81 (d, *J* = 8.4 Hz, 2H), 7.62 – 7.45 (m, 3H), 6.90 (d, *J* = 8.4 Hz, 2H), 6.81 (d, *J* = 8.4 Hz, 1H), 6.72 (s, 1H), 3.89 (s, 2H), 3.78 (s, 3H), 2.39 ppm (s, 3H); ^{13}C NMR (125 MHz, DMSO-d_6): δ 181.8, 176.2, 165.8, 163.9, 160.3, 154.2, 153.9, 146.3, 135.1, 134.9, 132.9, 124.7, 124.3, 123.5, 122.7, 121.3, 118.2, 114.4, 113.1, 112.4, 110.6, 108.9, 55.3, 20.4, 18.6 ppm; MS (APCI): *m/z* 441.2 ([M+H]$^+$, 100). Anal. cacld (%) for $C_{27}H_{20}O_6$: C, 73.63; H, 4.58. Found (%): C, 73.84; H, 4.43.

6-Fluoro-3-{[(2*Z*)-6-hydroxy-2-(4-methoxybenzylidene)-3-oxo-2,3-dihydro-1-benzofuran-7-yl]methyl}-4*H*-chromen-4-one (5b). Yellow solid; yield 604 mg (68%); mp

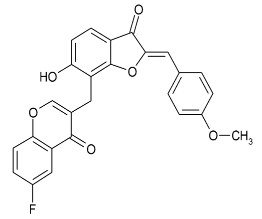

275-277°C; ^{1}H NMR (400 MHz, DMSO-d_6): δ 11.18 (s, 1H), 8.21 (s, 1H), 7.83 (d, *J* = 7.2 Hz, 2H), 7.79 – 7.60 (m, 3H), 7.52 (d, *J* = 8.3 Hz, 1H), 6.93 (d, *J* = 7.2 Hz, 2H), 6.81 (d, *J* = 8.3 Hz, 1H), 6.72 (s, 1H), 3.90 (s, 2H), 3.79 ppm (s, 3H); ^{13}C NMR (125 MHz, DMSO-d_6): δ 181.7, 175.6, 165.8, 163.8, 160.3, 158.8 (d, $J_{C\text{-}F}$ = 244.0 Hz), 154.4, 152.3, 146.2, 132.9, 124.6, 124.0 (d, $J_{C\text{-}F}$ = 7.2 Hz), 123.5, 122.2 (d, $J_{C\text{-}F}$ = 25.4 Hz), 121.2 (d, $J_{C\text{-}F}$ = 8.5 Hz), 120.9, 114.4, 113.1, 112.3, 110.5, 109.6 (d, $J_{C\text{-}F}$ = 23.8 Hz), 108.7, 55.3, 18.5 ppm; ^{19}F NMR (376 MHz, DMSO-d_6): δ -116.0 ppm; MS (APCI): *m/z* 445.0 ([M+H]$^+$, 100). Anal. cacld (%) for $C_{26}H_{17}FO_6$: C, 70.27; H, 3.86. Found (%): C, 70.14; H, 3.69.

6-Chloro-3-{[(2*Z*)-6-hydroxy-2-(4-methoxybenzylidene)-3-oxo-2,3-dihydro-1-benzofuran-7-yl]methyl}-4*H*-chromen-4-one (5c). Yellow solid; yield 664 mg (72%); mp

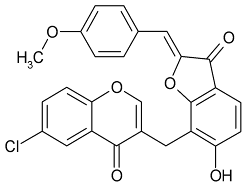

325-327°C; ^{1}H NMR (400 MHz, DMSO-d_6): δ 11.21 (s, 1H), 8.23 (s, 1H), 8.03 (d, *J* = 2.7 Hz, 1H), 7.87 – 7.78 (m, 3H), 7.70 (d, *J* = 9.0 Hz, 1H), 7.53 (d, *J* = 8.4 Hz, 1H), 6.93 (d, *J* = 8.4 Hz, 2H), 6.81 (d, *J* = 8.4 Hz, 1H), 6.73 (s, 1H), 3.90 (s, 2H), 3.80 ppm (s, 3H); ^{13}C NMR (100 MHz, DMSO-d_6): δ181.7, 175.2, 165.8, 163.9, 160.3, 154.4, 154.4, 146.2, 133.9, 132.9, 129.8, 124.6, 124.1, 124.0, 123.6, 121.7, 120.9, 114.4, 113.1, 112.3, 110.5, 108.6, 55.3, 18.5 ppm; MS (APCI): *m/z* 461.2 ([M+H]$^+$, 100). Anal. cacld (%) for $C_{26}H_{17}ClO_6$: C, 67.76; H, 3.72. Found (%): C, 67.99; H, 3.89.

3-{[(2*Z*)-2-(3,4-Dimethoxybenzylidene)-6-hydroxy-3-oxo-2,3-dihydro-1-benzofuran-7-yl]methyl}-6-methyl-4*H*-chromen-4-one (5d). Yellow solid; yield 659 mg

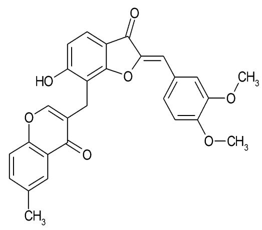

(70%); mp 229-231°C; ^{1}H NMR (400 MHz, DMSO-d_6): δ 11.20 (s, 1H), 7.98 (s, 1H), 7.91 – 7.85 (m, 1H), 7.65 (d, *J* = 2.0 Hz, 1H), 7.60 – 7.56 (m, 1H), 7.54 (d, *J* = 8.4 Hz, 1H), 7.49 (d, *J* = 8.5 Hz, 1H), 7.41 (dd, *J* = 8.4, 1.9 Hz, 1H), 6.93 (d, *J* = 8.5 Hz, 1H), 6.81 (d, *J* = 8.5 Hz, 1H), 6.74 (s, 1H), 3.87 (s, 2H), 3.78 (s, 3H), 3.66 (s, 3H), 2.41 ppm (s, 3H); ^{13}C NMR (125 MHz, DMSO-d_6): δ 181.8, 176.3, 165.8, 164.0, 154.1, 153.6, 150.2, 148.6, 146.4, 135.1, 134.9, 125.3, 124.9, 124.2, 123.7, 122.6, 120.9, 118.1, 113.4, 113.1, 112.3, 111.6, 111.0, 108.2, 55.5, 55.0, 20.4, 18.7 ppm; MS (APCI): *m/z* 471.2 ([M+H]$^+$, 100). Anal. cacld (%) for $C_{28}H_{22}O_7$: C, 71.48; H, 4.71. Found (%): C, 71.67; H, 4.83.

3-{[(2*Z*)-2-(3,4-Dimethoxybenzylidene)-6-hydroxy-3-oxo-2,3-dihydro-1-benzofuran-7-yl]methyl}-6-methyl-4*H*-chromen-4-one (5e). Yellow solid; yield 617 mg (65%); mp 254-255°C; ^{1}H NMR (400 MHz, DMSO-d_6): δ 11.21 (s, 1H), 8.08 (s, 1H), 7.81 – 7.67 (m, 3H), 7.66 (d, J = 2.0 Hz, 1H), 7.54 (d, J = 8.4 Hz, 1H), 7.42 (dd, J = 8.5, 2.0 Hz, 1H), 6.95 (d, J = 8.5 Hz, 1H), 6.81 (d, J = 8.4 Hz, 1H), 6.75 (s, 1H), 3.88 (s, 2H), 3.79 (s, 3H), 3.67 ppm (s, 3H); ^{13}C NMR (125 MHz, DMSO-d_6): δ 181.8, 175.7, 165.8, 164.1, 158.9 (d, $J_{C\text{-}F}$ = 244.3 Hz), 154.2, 152.3, 150.2, 148.6, 146.4, 125.3, 124.9, 124.0 (d, $J_{C\text{-}F}$ = 7.2 Hz), 123.8, 122.3 (d, $J_{C\text{-}F}$ = 25.4 Hz), 121.2 (d, $J_{C\text{-}F}$ = 8.5 Hz), 120.5, 113.5, 113.1, 112.3, 111.7, 111.0, 109.5 (d, $J_{C\text{-}F}$ = 23.5 Hz), 108.0, 55.5, 55.1, 18.7 ppm; ^{19}F NMR (376 MHz, DMSO-d_6): δ -115.5 ppm; MS (APCI): *m/z* 475.0 ([M+H]$^+$, 100). Anal. cacld (%) for $C_{27}H_{19}FO_7$: C, 68.35; H, 4.04. Found (%): C, 68.20; H, 3.86.

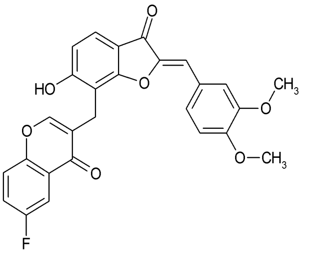

6-Chloro-3-{[(2*Z*)-2-(3,4-dimethoxybenzylidene)-6-hydroxy-3-oxo-2,3-dihydro-1-benzofuran-7-yl]methyl}-4*H*-chromen-4-one (5f). Yellow solid; yield 746 mg (76%); mp 250-252°C; ^{1}H NMR (400 MHz, DMSO-d_6): δ 11.21 (s, 1H), 8.04 (s, 1H), 8.00 (d, J = 2.8 Hz, 1H), 7.77 (dd, J = 8.9, 2.8 Hz, 1H), 7.67 – 7.59 (m, 2H), 7.53 (d, J = 8.3 Hz, 1H), 7.43 – 7.36 (m, 1H), 6.91 (d, J = 8.4 Hz, 1H), 6.80 (d, J = 8.4 Hz, 1H), 6.72 (s, 1H), 3.86 (s, 2H), 3.77 (s, 3H), 3.67 ppm (s, 3H); ^{13}C NMR (100 MHz, DMSO-d_6): δ 181.7, 175.2, 165.7, 164.0, 154.4 (2C), 154.1, 150.2, 148.6, 146.3, 133.9, 129.8, 125.2, 124.9, 123.9, 123.8, 121.3, 120.8, 113.5, 113.2, 112.3, 111.5, 111.0, 107.9, 55.5, 55.1, 18.7 ppm; MS (APCI): *m/z* 491.2 ([M+H]$^+$, 100). Anal. cacld (%) for $C_{27}H_{19}ClO_7$: C, 66.06; H, 3.90. Found (%): C, 66.26; H, 3.79.

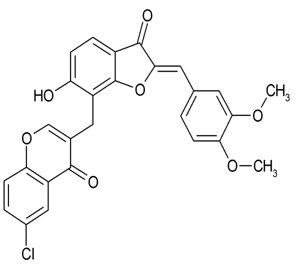

3-{[(2*Z*)-6-Hydroxy-3-oxo-2-(3,4,5-trimethoxybenzylidene)-2,3-dihydro-1-benzofuran-7-yl]methyl}-6-methyl-4*H*-chromen-4-one (5g). Yellow solid; yield 711 mg (71%); mp 275-277°C; ^{1}H NMR (400 MHz, DMSO-d_6): δ 11.26 (s, 1H), 7.87 (d, J = 12.7 Hz, 2H), 7.60 – 7.51 (m, 2H), 7.46 (dd, J = 8.6, 1.8 Hz, 1H), 7.33 (s, 2H), 6.91 – 6.59 (m, 2H), 3.84 (s, 2H), 3.69 (s, 6H), 3.67 (s, 3H), 2.40 ppm (s, 3H); ^{13}C NMR (100 MHz, DMSO-d_6): δ 181.9, 176.3, 166.0, 164.3, 154.1, 153.4, 152.9, 147.1, 138.8, 135.2, 135.0, 127.7, 124.1, 124.0, 122.6, 120.6, 118.1, 112.9, 112.5, 110.7, 108.5, 107.8, 60.1, 55.6, 20.4, 18.8 ppm; MS (APCI): *m/z* 501.1 ([M+H]$^+$, 100). Anal. cacld (%) for $C_{29}H_{24}O_8$: C, 69.59; H, 4.83. Found (%): C, 69.46; H, 4.65.

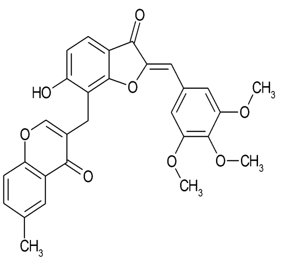

6-Fluoro-3-{[(2*Z*)-6-hydroxy-3-oxo-2-(3,4,5-trimethoxybenzylidene)-2,3-dihydro-1-benzofuran-7-yl]methyl}-4*H*-chromen-4-one (5h). Yellow solid; yield 797 mg (79%); mp 263-265°C; ^{1}H NMR (400 MHz, DMSO-d_6): δ 11.29 (s, 1H), 8.01 (s, 1H), 7.80 – 7.64 (m, 3H), 7.56 (d, J = 8.5 Hz, 1H), 7.35 (s, 2H), 6.81 (d, J = 8.5 Hz, 1H), 6.76 (s, 1H), 3.86 (s, 2H), 3.71 (s, 6H), 3.68 ppm (s, 3H); ^{13}C NMR (125 MHz, DMSO-d_6): δ 181.9, 175.8, 166.0, 164.3, 158.9 (d, $J_{C\text{-}F}$ = 244.4 Hz), 154.0, 152.9, 152.3, 147.1, 138.9, 127.7, 124.1, 123.9 (d, $J_{C\text{-}F}$ = 7.2 Hz), 122.3 (d, $J_{C\text{-}F}$ = 25.5 Hz), 121.2 (d, $J_{C\text{-}F}$ = 8.3 Hz), 120.2, 112.9, 112.4, 110.7, 109.5 (d, $J_{C\text{-}F}$ = 23.4 Hz), 108.6, 107.6, 60.1, 55.6, 18.7 ppm; ^{19}F NMR (470 MHz, DMSO-d_6): δ -115.4 ppm; MS (APCI): *m/z* 505.0 ([M+H]$^+$, 100). Anal. cacld (%) for $C_{28}H_{21}FO_8$: C, 66.67; H, 4.20. Found (%): C, 66.44; H, 4.36.

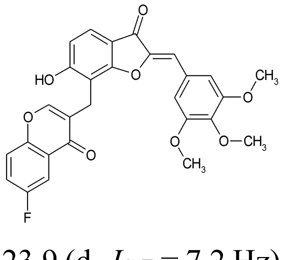

6-Chloro-3-{[(2*Z*)-6-hydroxy-3-oxo-2-(3,4,5-trimethoxybenzylidene)-2,3-dihydro-1-benzofuran-7-yl]methyl}-4*H*-chromen-4-one (5i). Yellow solid; yield 813 mg (78%); mp 265-267°C; ^{1}H NMR (400 MHz, DMSO-d_6): δ 11.27 (s, 1H), 8.03 – 7.98 (m, 2H), 7.80 (dd, *J* = 9.0, 2.7 Hz, 1H), 7.66 (d, *J* = 9.0 Hz, 1H), 7.56 (d, *J* = 8.4 Hz, 1H), 7.35 (s, 2H), 6.81 (d, *J* = 8.4 Hz, 1H), 6.75 (s, 1H), 3.86 (s, 2H), 3.72 (s, 6H), 3.68 ppm (s, 3H); ^{13}C NMR (125 MHz, DMSO-d_6): δ 181.9, 175.3, 165.9, 164.3, 154.4, 154.0, 152.8, 147.1, 138.8, 134.0, 129.8, 127.6, 124.1, 123.9, 120.9, 120.9, 112.9, 112.4, 110.7, 108.6 (2C), 107.5, 60.1, 55.6, 18.7 ppm; MS (APCI): *m/z* 521.0 ([M+H]$^+$, 100). Anal. cacld (%) for $C_{28}H_{21}ClO_8$: C, 64.56; H, 4.06. Found (%): C, 64.73; H, 4.15.

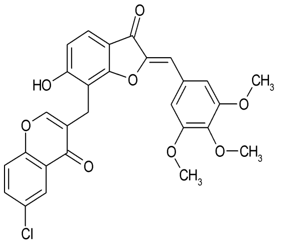

(2*Z*)-6-Hydroxy-7-{[3-(2-hydroxy-5-methylphenyl)-1*H*-pyrazol-4-yl]methyl}-2-(4-methoxybenzylidene)-1-benzofuran-3(2*H*)-one (6a). Yellow solid; yield 295 mg (65%); mp 283-285°C; ^{1}H NMR (400 MHz, DMSO-d_6+CF_3SO_3H): δ 8.06 (s, 1H), 7.57 (d, *J* = 8.3 Hz, 2H), 7.43 (d, *J* = 8.4 Hz, 1H), 7.13 – 7.01 (m, 2H), 6.92 – 6.83 (m, 3H), 6.76 (d, *J* = 8.4 Hz, 1H), 6.62 (s, 1H), 3.99 (s, 2H), 3.78 (s, 3H), 2.14 ppm (s, 3H); ^{13}C NMR (125 MHz, DMSO-d_6+CF_3SO_3H): δ 182.0, 165.8, 164.0, 160.6, 153.6, 146.6, 142.3, 133.6, 133.1, 132.6, 131.0, 128.1, 124.9, 124.0, 119.1, 116.2, 114.8, 113.3, 113.1, 112.5, 110.8, 109.4, 55.6, 20.1, 17.8 ppm; MS (APCI): *m/z* 455.2 ([M+H]$^+$, 100). Anal. cacld (%) for $C_{27}H_{22}N_2O_5$: C, 71.36; H, 4.88; N, 6.16. Found (%): C, 71.18; H, 5.03; N, 6.04.

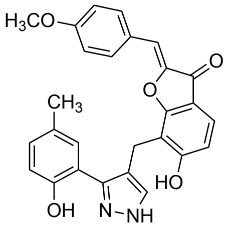

(2*Z*)-7-{[3-(5-Fluoro-2-hydroxyphenyl)-1*H*-pyrazol-4-yl]methyl}-6-hydroxy-2-(4-methoxybenzylidene)-1-benzofuran-3(2*H*)-one (6b). Yellow solid; yield 266 mg (58%); mp 232-234°C; ^{1}H NMR (400 MHz, DMSO-d_6+CF_3SO_3H): δ 8.01 (s, 1H), 7.59 (d, *J* = 8.6 Hz, 2H), 7.42 (d, *J* = 8.4 Hz, 1H), 7.21 – 7.13 (m, 1H), 7.07 – 6.99 (m, 1H), 6.96 – 6.92 (m, 1H), 6.89 (d, *J* = 8.6 Hz, 2H), 6.75 (d, *J* = 8.4 Hz, 1H), 6.62 (s, 1H), 3.99 (s, 2H), 3.77 ppm (s, 3H); ^{13}C NMR (100 MHz, DMSO-d_6+CF_3SO_3H): δ 182.2, 166.0, 164.1, 160.8, 155.4 (d, $J_{C\text{-}F}$ = 234.6 Hz), 152.3, 146.7, 141.8, 133.4, 133.2, 125.1, 124.0, 118.5, 117.5, 117.4 (d, $J_{C\text{-}F}$ = 8.9 Hz), 116.7, 116.5 (d, $J_{C\text{-}F}$ = 12.0 Hz), 114.9, 113.5, 112.7, 111.0, 110.4, 55.7, 18.2 ppm; ^{19}F NMR (376 MHz, DMSO-d_6): δ -125.2 ppm; MS (APCI): *m/z* 459.0 ([M+H]$^+$, 100). Anal. cacld (%) for $C_{26}H_{19}FN_2O_5$: C, 68.12; H, 4.18; N, 6.11. Found (%): C, 68.34; H, 4.34; N, 6.23.

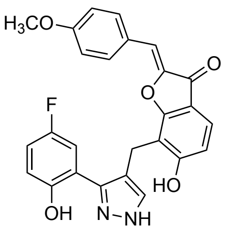

(2*Z*)-7-{[3-(5-Chloro-2-hydroxyphenyl)-1*H*-pyrazol-4-yl]methyl}-6-hydroxy-2-(4-methoxybenzylidene)-1-benzofuran-3(2*H*)-one (6c). Yellow solid; yield 337 mg (71%); mp 310-312°C; ^{1}H NMR (400 MHz, DMSO-d_6+CF_3SO_3H): δ 8.04 (s, 1H), 7.58 (d, *J* = 8.3 Hz, 2H), 7.43 (d, *J* = 8.4 Hz, 1H), 7.34 – 7.29 (m, 1H), 7.23 (d, *J* = 8.8 Hz, 1H), 6.96 – 6.86 (m, 3H), 6.75 (d, *J* = 8.4 Hz, 1H), 6.63 (s, 1H), 3.97 (s, 2H), 3.78 ppm (s, 3H); ^{13}C NMR (125 MHz, DMSO-d_6+CF_3SO_3H): δ 182.0, 165.7, 163.9, 160.7, 154.9, 146.6, 141.0, 133.6, 133.1, 131.5, 130.2, 125.0, 124.0, 122.8, 119.3, 117.9, 115.7, 114.8, 113.3, 112.5, 110.9, 109.4, 55.6, 17.7 ppm; MS (APCI): *m/z* 475.0 ([M+H]$^+$, 100). Anal. cacld (%) for $C_{26}H_{19}ClN_2O_5$: C, 65.76; H, 4.03; N, 5.90. Found (%): C, 65.89; H, 3.88; N, 5.73.

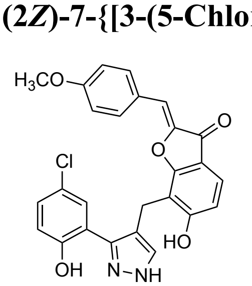

(2*Z*)-2-(3,4-Dimethoxybenzylidene)-6-hydroxy-7-{[3-(2-hydroxy-5-methylphenyl)-1*H*-pyrazol-4-yl]methyl}-1-benzofuran-3(2*H*)-one (6d). Yellow solid; yield 286 mg (59%); mp 163-165°C; ^{1}H NMR (400 MHz, DMSO-d_6+CF_3SO_3H): δ 8.00 (s, 1H), 7.46 (d, *J* = 8.4 Hz, 1H), 7.41 (s, 1H), 7.26 – 7.17 (m, 1H), 7.12 – 6.99 (m, 2H), 6.93 – 6.82 (m, 2H), 6.77 (d, *J* = 8.4 Hz, 1H), 6.65 (s, 1H), 3.96 (s, 2H), 3.78 (s, 3H), 3.54 (s, 3H), 2.13 ppm (s, 3H); ^{13}C NMR (125 MHz, DMSO-d_6+CF_3SO_3H): δ 182.0, 165.8, 164.0, 153.6, 150.5, 148.9, 146.8, 142.3, 133.5, 132.6, 131.0, 128.2, 125.4, 125.2, 124.2, 118.9, 116.3, 113.8, 113.4, 113.1, 112.6, 112.0, 111.3, 109.3, 55.9, 55.2, 20.1, 17.8 ppm; MS (APCI): *m/z* 485.2 ([M+H]$^+$, 100). Anal. cacld (%) for $C_{28}H_{24}N_2O_6$: C, 69.41; H, 4.99; N, 5.78. Found (%): C, 69.25; H, 4.88; N, 5.97.

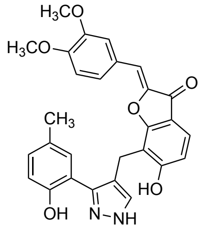

(2*Z*)-6-Hydroxy-7-{[3-(2-hydroxy-5-methylphenyl)-1*H*-pyrazol-4-yl]methyl}-2-(3,4,5-trimethoxybenzylidene)-1-benzofuran-3(2*H*)-one (6e). Yellow solid; yield 314 mg (61%); mp 159-161°C; ^{1}H NMR (400 MHz, DMSO-d_6+CF_3SO_3H): δ 7.95 (s, 1H), 7.45 (d, *J* = 8.5 Hz, 1H), 7.07 (s, 2H), 7.03 – 6.94 (m, 2H), 6.81 (d, *J* = 8.4 Hz, 1H), 6.74 (d, *J* = 8.5 Hz, 1H), 6.61 (s, 1H), 3.91 (s, 2H), 3.65 (s, 3H), 3.57 (s, 6H), 2.08 ppm (s, 3H); ^{13}C NMR (125 MHz, DMSO-d_6+CF_3SO_3H): δ 182.2, 166.0, 164.2, 153.6, 153.1, 147.5, 142.3, 139.1, 133.5, 132.6, 130.9, 128.3, 128.0, 124.4, 118.8, 116.3, 113.2, 113.1, 112.7, 111.0, 109.2, 108.7, 60.5, 55.7, 20.1, 17.7 ppm; MS (APCI): *m/z* 515.2 ([M+H]$^+$, 100). Anal. cacld (%) for $C_{29}H_{26}N_2O_7$: C, 67.70; H, 5.09; N, 5.44. Found (%): C, 67.49; H, 5.21; N, 5.27.

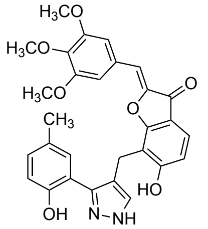

(2*Z*)-7-{[3-(5-Fluoro-2-hydroxyphenyl)-1*H*-pyrazol-4-yl]methyl}-6-hydroxy-2-(3,4,5-trimethoxybenzylidene)-1-benzofuran-3(2*H*)-one (6f). Yellow solid; yield 332 mg (64%); mp 254-256°C; ^{1}H NMR (400 MHz, DMSO-d_6): δ 7.78 (s, 1H), 7.49 (d, *J* = 8.4 Hz, 1H), 7.24 – 7.18 (m, 1H), 7.15 (s, 2H), 7.06 – 6.97 (m, 1H), 6.96 – 6.90 (m, 1H), 6.78 (d, *J* = 8.4 Hz, 1H), 6.68 (s, 1H), 3.98 (s, 2H), 3.67 (s, 3H), 3.60 ppm (s, 6H); ^{13}C NMR (125 MHz, DMSO-d_6): δ 181.8, 164.7 (d, J_{C-F} = 245.8 Hz), 154.0, 152.7, 151.9, 147.1, 141.4, 138.7, 132.4, 127.6, 123.9, 121.9, 117.6, 117.1 (d, J_{C-F} = 7.8 Hz), 117.0 (d, J_{C-F} = 6.7 Hz), 115.9, 115.7, 112.8, 112.4, 110.7, 109.4, 108.3, 60.1, 55.3, 17.7 ppm; ^{19}F NMR (376 MHz, DMSO-d_6): δ -125.1 ppm; MS (APCI): *m/z* 518.8 ([M+H]$^+$, 100). Anal. cacld (%) for $C_{28}H_{23}FN_2O_7$: C, 64.86; H, 4.47; N, 5.40. Found (%): C, 65.01; H, 4.33; N, 5.24.

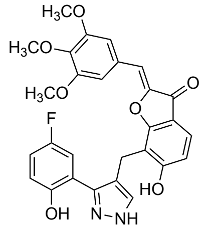

(2*Z*)-7-{[3-(5-Chloro-2-hydroxyphenyl)-1*H*-pyrazol-4-yl]methyl}-6-hydroxy-2-(3,4,5-trimethoxybenzylidene)-1-benzofuran-3(2*H*)-one (6g). Yellow solid; yield 385 mg (72%); mp 215-217°C; ^{1}H NMR (400 MHz, DMSO-d_6+CF_3SO_3H): δ 7.67 (s, 1H), 7.54 (d, *J* = 7.5 Hz, 1H), 7.44 – 7.38 (m, 1H), 7.27 (d, *J* = 8.5 Hz, 1H), 7.20 (s, 2H), 6.99 – 6.93 (m, 1H), 6.82 – 6.77 (m, 1H), 6.73 (s, 1H), 3.98 (s, 2H), 3.68 (s, 3H), 3.60 ppm (s, 6H); ^{13}C NMR (125 MHz, DMSO-d_6+CF_3SO_3H): δ 181.8, 165.8, 163.8, 154.4, 152.7, 147.1, 141.3, 138.7, 132.4, 129.9, 129.0, 127.6, 123.8, 122.5, 117.7, 117.5, 117.3, 112.8, 112.4, 110.6, 109.6, 108.3, 60.1, 55.3, 17.8 ppm; MS (APCI): *m/z* 535.0 ([M+H]$^+$, 100). Anal. cacld (%) for $C_{28}H_{23}ClN_2O_7$: C, 62.87; H, 4.33; N, 5.24. Found (%): C, 62.73; H, 4.16; N, 5.11.

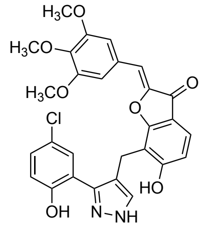

(2*Z*)-7-{[5-(5-Fluoro-2-hydroxyphenyl)isoxazol-4-yl]methyl}-6-hydroxy-2-(4-methoxybenzylidene)-1-benzofuran-3(2*H*)-one (7a). Yellow solid; yield 257 mg (56%); mp 330-332°C; ^{1}H NMR (500 MHz, $CDCl_3$+CF_3COOH): δ 8.09 (s, 1H), 7.89 – 7.78 (m, 3H), 7.67 (d, *J* = 8.5 Hz, 1H), 7.56 – 7.50 (m, 2H), 7.13 (s, 1H), 7.07 (d, *J* = 8.2 Hz, 2H), 6.94 (d, *J* = 8.5 Hz, 1H), 4.20 (s, 2H), 3.93 ppm (s, 3H); ^{13}C NMR (125 MHz, $CDCl_3$+CF_3COOH): δ 183.6, 167.9, 167.7, 165.9, 163.1, 163.0, 160.7 (d, $J_{C\text{-}F}$ = 250.9 Hz), 148.1, 146.0, 134.6, 127.0, 124.1, 124.1 (d, $J_{C\text{-}F}$ = 25.2 Hz), 119.4 (d, $J_{C\text{-}F}$ = 8.4 Hz), 121.1, 117.0 (d, $J_{C\text{-}F}$ = 9.5 Hz), 115.6, 114.8, 114.5, 110.4 (d, $J_{C\text{-}F}$ = 26.1 Hz), 106.8, 99.4, 55.9, 16.9 ppm; ^{19}F NMR (376 MHz, DMSO-d_6): δ -117.6 ppm; MS (APCI): *m/z* 460.0 ($[M+H]^+$, 100). Anal. cacld (%) for $C_{26}H_{18}FNO_6$: C, 67.97; H, 3.95; N, 3.05. Found (%): C, 67.79; H, 3.84; N, 3.20.

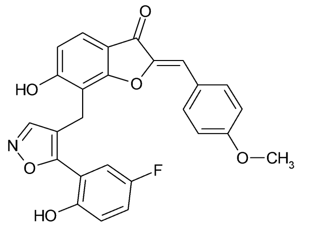

(2*Z*)-2-(3,4-Dimethoxybenzylidene)-6-hydroxy-7-{[5-(2-hydroxy-5-methylphenyl)isoxazol-4-yl]methyl}-1-benzofuran-3(2*H*)-one (7b). Yellow solid; yield 218 mg (45%); mp 318-320°C; ^{1}H NMR (500 MHz, DMSO-d_6): δ 12.22 (s, 1H), 7.81 – 7.76 (m, 2H), 7.69 – 7.63 (m, 2H), 7.50 – 7.39 (m, 3H), 7.28 (d, *J* = 8.4 Hz, 1H), 7.08 (d, *J* = 8.5 Hz, 1H), 6.77 – 6.72 (m, 2H), 3.89 (s, 2H), 3.85 (s, 3H), 3.80 (s, 3H), 2.34 ppm (s, 3H); ^{13}C NMR (125 MHz, DMSO-d_6): δ 181.6, 174.7, 165.7, 164.5, 163.2, 150.6, 150.3, 148.6, 146.2, 133.9, 133.3, 125.0, 124.5, 124.4, 123.2, 121.0, 116.1, 114.8, 113.6, 113.2, 112.1, 111.3, 110.8, 95.1, 55.6, 55.5, 20.4, 17.1 ppm; MS (APCI): *m/z* 486.2 ($[M+H]^+$, 100). Anal. cacld (%) for $C_{28}H_{23}NO_7$: C, 69.27; H, 4.78; N, 2.89. Found (%): C, 69.49; H, 4.92; N, 2.74.

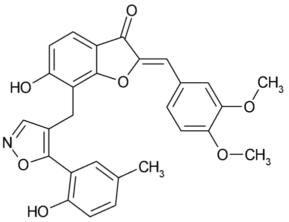

(2*Z*)-2-(3,4-Dimethoxybenzylidene)-7-{[5-(5-fluoro-2-hydroxyphenyl)isoxazol-4-yl]methyl}-6-hydroxy-1-benzofuran-3(2*H*)-one (7c). Yellow solid; yield 303 mg (62%); mp 296-298°C; ^{1}H NMR (500 MHz, $CDCl_3$+CF_3COOH): δ 8.13 (s, 1H), 7.82 (d, *J* = 7.7 Hz, 1H), 7.69 (d, *J* = 8.6 Hz, 1H), 7.65 – 7.60 (m, 1H), 7.56 – 7.52 (m, 2H), 7.47 (s, 1H), 7.13 (s, 1H), 7.11 – 7.07 (m, 1H), 6.95 (d, *J* = 8.6 Hz, 1H), 4.20 (s, 2H), 4.00 (s, 3H), 3.98 ppm (s, 3H); ^{13}C NMR (125 MHz, $CDCl_3$+CF_3COOH): δ 183.9, 167.9, 167.7, 166.1, 163.2, 159.7, 159.3, 152.5, 148.1, 147.6 (d, $J_{C\text{-}F}$ = 273.9 Hz), 128.0, 127.0, 124.7, 124.1 (d, $J_{C\text{-}F}$ = 24.9 Hz), 119.9, 119.5 (d, $J_{C\text{-}F}$ = 8.7 Hz), 116.9 (d, $J_{C\text{-}F}$ = 11.8 Hz), 116.2, 114.8, 114.5, 112.5, 110.1 (d, $J_{C\text{-}F}$ = 26.2 Hz), 106.9, 99.6, 57.0, 56.2, 16.9 ppm; ^{19}F NMR (376 MHz, DMSO-d_6): δ -117.0 ppm; MS (APCI): *m/z* 490.2 ($[M+H]^+$, 100). Anal. cacld (%) for $C_{27}H_{20}FNO_7$: C, 66.26; H, 4.12; N, 2.86. Found (%): C, 66.42; H, 3.99; N, 2.69.

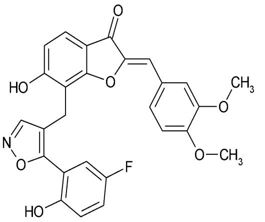

(2*Z*)-6-Hydroxy-7-{[5-(2-hydroxy-5-methylphenyl)isoxazol-4-yl]methyl}-2-(3,4,5-trimethoxybenzylidene)-1-benzofuran-3(2*H*)-one (7d). Yellow solid; yield 289 mg (56%); mp >330°C; ^{1}H NMR (500 MHz, $CDCl_3$+CF_3COOH): δ 8.05 – 7.81 (m, 2H), 7.74 (d, *J* = 8.4 Hz, 1H), 7.67 (d, *J* = 8.6 Hz, 1H), 7.42 (d, *J* = 8.4 Hz, 1H), 7.23 (s, 2H), 7.17 (s, 1H), 7.01 (d, *J* = 8.6 Hz, 1H), 4.14 (s, 2H), 4.06 (s, 3H), 4.01 (s, 6H), 2.48 ppm (s, 3H); ^{13}C NMR (125 MHz, $CDCl_3$+CF_3COOH): δ 184.5, 168.6, 167.7, 166.3, 163.2, 153.4, 150.4, 147.6, 139.7, 138.8, 137.6, 128.4, 127.3, 123.6, 118.2, 117.0, 115.0, 114.8, 114.5, 110.1, 107.3, 98.8, 61.9, 57.0, 20.8, 17.1 ppm; MS (APCI): *m/z* 516.2 ($[M+H]^+$, 100). Anal. cacld (%) for $C_{29}H_{25}NO_8$: C, 67.57; H, 4.89; N, 2.72. Found (%): C, 67.33; H, 5.02; N, 2.90.

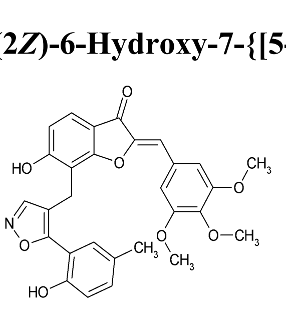

(2*Z*)-7-{[5-(5-Fluoro-2-hydroxyphenyl)isoxazol-4-yl]methyl}-6-hydroxy-2-(3,4,5-trimethoxybenzylidene)-1-benzofuran-3(2*H*)-one (7e). Yellow solid; yield 255 mg (49%); mp 327-329°C; ^{1}H NMR (500 MHz, $CDCl_3$+CF_3COOH): δ 8.07 (s, 1H), 7.79 (d, J = 7.6 Hz, 1H), 7.74 (d, J = 8.4 Hz, 1H), 7.60 – 7.51 (m, 2H), 7.22 (s, 2H), 7.16 (s, 1H), 7.00 (d, J = 8.4 Hz, 1H), 4.15 (s, 2H), 4.05 (s, 3H), 4.00 ppm (s, 6H); ^{13}C NMR (125 MHz, $CDCl_3$+CF_3COOH): δ 184.4, 168.2, 167.8, 166.2, 163.2, 160.7 (d, $J_{C\text{-}F}$ = 250.7 Hz), 153.4, 148.2, 147.5, 139.8, 128.3, 127.3, 124.2 (d, $J_{C\text{-}F}$ = 25.6 Hz), 119.5 (d, $J_{C\text{-}F}$ = 8.4 Hz), 118.2, 116.9 (d, $J_{C\text{-}F}$ = 9.3 Hz), 114.7, 114.5, 110.0, 107.0, 110.0 (d, $J_{C\text{-}F}$ = 26.1 Hz), 99.6, 61.8, 57.0, 17.2 ppm; ^{19}F NMR (376 MHz, DMSO-d_6): δ -117.6 ppm; MS (APCI): *m/z* 518.0 ($[M+H]^+$, 100). Anal. cacld (%) for $C_{28}H_{22}FNO_8$: C, 64.74; H, 4.27; N, 2.70. Found (%): C, 64.58; H, 4.16; N, 2.55.

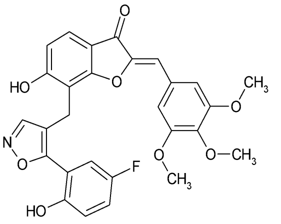

(2*Z*)-7-{[5-(5-Chloro-2-hydroxyphenyl)isoxazol-4-yl]methyl}-6-hydroxy-2-(3,4,5-trimethoxybenzylidene)-1-benzofuran-3(2*H*)-one (7f). Yellow solid; yield 359 mg (67%); mp >330°C; ^{1}H NMR (500 MHz, $CDCl_3$+CF_3COOH): δ 8.12 – 8.05 (m, 2H), 7.82 – 7.71 (m, 2H), 7.47 (d, J = 9.0 Hz, 1H), 7.23 (s, 2H), 7.19 (s, 1H), 7.01 (d, J = 8.5 Hz, 1H), 4.15 (s, 2H), 4.07 (s, 3H), 4.01 ppm (s, 6H); ^{13}C NMR (125 MHz, $CDCl_3$+CF_3COOH): δ 184.5, 167.9, 167.8, 166.3, 163.2, 153.5, 150.3, 147.7, 139.7, 136.4, 134.2, 128.5, 127.5, 123.9, 118.8, 118.3, 116.7, 114.9, 114.6, 110.1, 107.0, 99.8, 62.0, 57.2, 17.2 ppm; MS (APCI): *m/z* 536.0 ($[M+H]^+$, 100). Anal. cacld (%) for $C_{28}H_{22}ClNO_8$: C, 62.75; H, 4.14; N, 2.61. Found (%): C, 62.94; H, 4.28; N, 2.49.

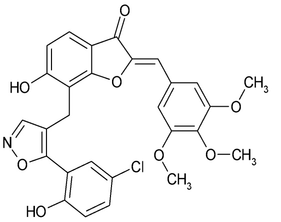

(2*Z*)-7-{[2-Amino-4-(5-chloro-2-hydroxyphenyl)pyrimidin-5-yl]methyl}-6-hydroxy-2-(4-methoxybenzylidene)-1-benzofuran-3(2*H*)-one (8a). Yellow solid; yield 356 mg (71%); mp 311-313°C; ^{1}H NMR (400 MHz, DMSO-d_6): δ 10.58 (s, 2H), 8.06 (s, 1H), 7.63 (d, J = 8.4 Hz, 2H), 7.44 (d, J = 8.5 Hz, 1H), 7.13 (dd, J = 8.8, 2.7 Hz, 1H), 7.03 (d, J = 8.4 Hz, 2H), 6.97 (d, J = 2.7 Hz, 1H), 6.82 (d, J = 8.8 Hz, 1H), 6.70 (d, J = 8.5 Hz, 1H), 6.66 (s, 1H), 6.51 (s, 2H), 3.86 (s, 2H), 3.84 ppm (s, 3H); ^{13}C NMR (125 MHz, DMSO-d_6): δ 181.6, 165.6, 164.2, 163.2, 162.0, 160.3, 158.3, 153.6, 146.2, 132.8, 129.2, 128.8, 127.3, 124.7, 123.3, 122.0, 119.2, 117.5, 114.6, 112.7, 112.2, 110.2, 109.7, 55.4, 22.6 ppm; MS (APCI): *m/z* 502.2 ($[M+H]^+$, 100). Anal. cacld (%) for $C_{27}H_{20}ClN_3O_5$: C, 64.61; H, 4.02; N, 8.37. Found (%): C, 64.46; H, 4.21; N, 8.24.

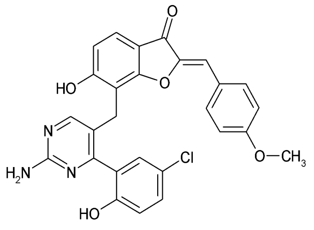

(2*Z*)-7-{[2-Amino-4-(5-chloro-2-hydroxyphenyl)pyrimidin-5-yl]methyl}-2-(3,4-dimethoxybenzylidene)-6-hydroxy-1-benzofuran-3(2*H*)-one (8b). Yellow solid; yield 340 mg (64%); mp 208-210°C; ^{1}H NMR (500 MHz, DMSO-d_6): δ 11.27 – 9.89 (m, 2H), 8.04 (s, 1H), 7.50 – 7.42 (m, 2H), 7.28 (dd, J = 8.2, 2.2 Hz, 1H), 7.13 – 7.01 (m, 2H), 6.91 – 6.88 (m, 1H), 6.76 (dd, J = 8.8, 2.0 Hz, 1H), 6.71 – 6.65 (m, 2H), 6.50 (s, 2H), 3.83 (s, 3H), 3.82 (s, 2H), 3.73 ppm (s, 3H); ^{13}C NMR (125 MHz, DMSO-d_6): δ 181.6, 165.5, 163.8, 163.3, 162.0, 158.2, 153.5, 150.2, 148.6, 146.4, 129.2, 128.8, 127.3, 124.9, 124.8, 123.5, 122.0, 119.2, 117.4, 114.0, 112.8, 112.2, 111.9, 110.8, 109.8, 55.6, 55.2, 22.4 ppm; MS (APCI): *m/z* 532.2 ($[M+H]^+$, 100). Anal. cacld (%) for $C_{28}H_{22}ClN_3O_6$: C, 63.22; H, 4.17; N, 7.90. Found (%): C, 63.02; H, 4.08; N, 8.04.

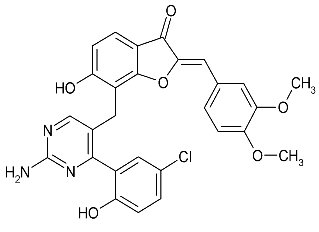

(2*Z*)-7-{[2-Amino-4-(2-hydroxy-5-methylphenyl)pyrimidin-5-yl]methyl}-6-hydroxy-2-(3,4,5-trimethoxybenzylidene)-1-benzofuran-3(2*H*)-one (8c). Yellow solid; yield 390 mg (72%); mp 305-307°C; ^{1}H NMR (400 MHz, DMSO-d_6): δ 11.36 – 8.97 (m, 2H), 7.94 (s, 1H), 7.48 (d, J = 8.4 Hz, 1H), 7.23 (s, 2H), 6.88 (d, J = 8.3 Hz, 1H), 6.80 – 6.60 (m, 4H), 6.41 (s, 2H), 3.83 (s, 2H), 3.73 (s, 9H), 2.09 ppm (s, 3H); ^{13}C NMR (125 MHz, DMSO-d_6): δ 181.9, 166.0, 164.9, 164.1, 161.9, 157.6, 152.9, 152.3, 147.1, 138.8, 130.2, 129.7, 127.8, 126.9, 125.1, 123.6, 118.9, 115.7, 112.5, 112.4, 110.5, 109.9, 108.4, 60.2, 55.6, 22.3, 20.0 ppm; MS (APCI): m/z 542.1 ([M+H]$^+$, 100). Anal. cacld (%) for $C_{30}H_{27}N_3O_7$: C, 66.54; H, 5.03; N, 7.76. Found (%): C, 66.71; H, 5.16; N, 7.61.

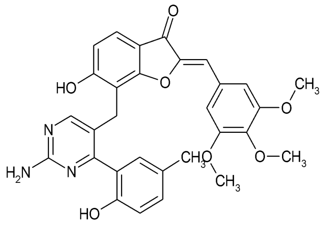

(2*Z*)-7-{[2-Amino-4-(5-fluoro-2-hydroxyphenyl)pyrimidin-5-yl]methyl}-6-hydroxy-2-(3,4,5-trimethoxybenzylidene)-1-benzofuran-3(2*H*)-one (8d). Yellow solid; yield 338 mg (62%); mp 301-303°C; ^{1}H NMR (400 MHz, DMSO-d_6): δ 10.98 (s, 1H), 9.69 (s, 1H), 8.02 (s, 1H), 7.48 (d, J = 8.4 Hz, 1H), 7.25 (s, 2H), 6.96 – 6.81 (m, 1H), 6.81 – 6.60 (m, 4H), 6.50 (s, 2H), 3.80 (s, 2H), 3.75 (s, 6H), 3.72 ppm (s, 3H); ^{13}C NMR (125 MHz, DMSO-d_6): δ 181.8, 165.8, 164.0, 163.5, 161.9, 158.1, 154.9 (d, $J_{C\text{-}F}$ = 234.5 Hz), 152.9, 150.7, 147.1, 138.8, 127.7, 126.5 (d, $J_{C\text{-}F}$ = 7.6 Hz), 123.7, 119.0, 116.7 (d, $J_{C\text{-}F}$ = 7.8 Hz), 116.0, 115.8, 115.4 (d, $J_{C\text{-}F}$ = 23.6 Hz), 112.4 (d, $J_{C\text{-}F}$ = 21.8 Hz), 110.6, 109.9, 108.5, 60.2, 55.7, 22.2 ppm; ^{19}F NMR (376 MHz, DMSO-d_6): δ-126.6 ppm; MS (APCI): m/z 546.1 ([M+H]$^+$, 100). Anal. cacld (%) for $C_{29}H_{24}FN_3O_7$: C, 63.85; H, 4.43; N, 7.70. Found (%): C, 63.61; H, 4.55; N, 7.87.

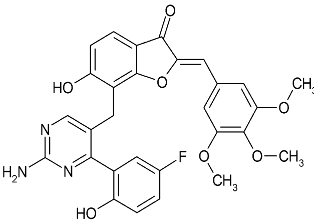

(2*Z*)-7-{[7-(5-Chloro-2-hydroxyphenyl)pyrazolo[1,5-*a*]pyrimidin-6-yl]methyl}-6-hydroxy-2-(4-methoxybenzylidene)-1-benzofuran-3(2*H*)-one (9a). Yellow solid; yield 321 mg (61%); mp 294-296°C; ^{1}H NMR (500 MHz, DMSO-d_6): δ 11.07 (s, 1H), 10.02 (s, 1H), 8.66 (s, 1H), 8.03 (s, 1H), 7.60 (d, J = 8.4 Hz, 2H), 7.45 (d, J = 8.4 Hz, 1H), 7.12 (dd, J = 8.7, 2.7 Hz, 1H), 6.97 (d, J = 2.7 Hz, 1H), 6.89 (d, J = 8.4 Hz, 2H), 6.76 (d, J = 8.8 Hz, 1H), 6.72 (s, 1H), 6.69 – 6.62 (m, 2H), 4.08 (d, J = 15.8 Hz, 1H), 3.95 (d, J = 15.8 Hz, 1H), 3.79 ppm (s, 3H); ^{13}C NMR (125 MHz, DMSO-d_6): δ 181.7, 165.4, 163.7, 160.2, 154.1, 152.0, 147.6, 146.2, 143.8, 141.5, 132.8, 130.6, 129.3, 124.6, 123.7, 122.2, 119.1, 118.6, 117.5, 114.4, 112.9, 112.0, 110.4, 109.2, 96.1, 55.3, 22.8 ppm; MS (APCI): m/z 526.2 ([M+H]$^+$, 100). Anal. cacld (%) for $C_{29}H_{20}ClN_3O_5$: C, 66.23; H, 3.83; N, 7.99. Found (%): C, 66.07; H, 3.72; N, 7.86.

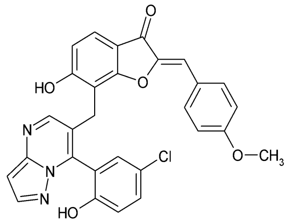

(2*Z*)-2-(3,4-Dimethoxybenzylidene)-7-{[7-(5-fluoro-2-hydroxyphenyl)pyrazolo[1,5-*a*]pyrimidin-6-yl]methyl}-6-hydroxy-1-benzofuran-3(2*H*)-one (9b). Yellow solid; yield 248 mg (46%); mp 213-215°C; ^{1}H NMR (500 MHz, DMSO-d_6): δ 11.07 (s, 1H), 9.67 (s, 1H), 8.64 (s, 1H), 8.03 (d, J = 2.4 Hz, 1H), 7.51 – 7.42 (m, 2H), 7.29 (d, J = 8.4 Hz, 1H), 6.97 – 6.88 (m, 2H), 6.86 – 6.80 (m, 1H), 6.76 – 6.64 (m, 4H), 4.08 (d, J = 15.6 Hz, 1H), 3.93 (d, J = 15.6 Hz, 1H), 3.82 (s, 3H), 3.77 ppm (s, 3H); ^{13}C NMR (125 MHz, DMSO-d_6): δ 181.6, 165.3, 163.9, 154.8 (d, $J_{C\text{-}F}$ = 234.5 Hz), 152.0, 151.5, 150.1, 148.6, 147.5, 146.3, 143.8, 141.6, 125.0, 124.9, 123.8, 119.0, 117.6 (d, $J_{C\text{-}F}$ = 8.8 Hz), 117.4 (d, $J_{C\text{-}F}$ = 22.5 Hz), 116.8 (d, $J_{C\text{-}F}$ = 7.9 Hz), 116.2 (d, $J_{C\text{-}F}$ = 24.1 Hz), 113.8, 112.8, 112.1, 111.7, 110.9, 109.3, 96.0, 55.6, 55.2, 22.7 ppm; ^{19}F NMR (376 MHz, DMSO-d_6): δ -126.2 ppm; MS (APCI): m/z 540.0 ([M+H]$^+$, 100). Anal. cacld (%) for $C_{30}H_{22}FN_3O_6$: C, 66.79; H, 4.11; N, 7.79. Found (%): C, 66.93; H, 4.28; N, 7.91.

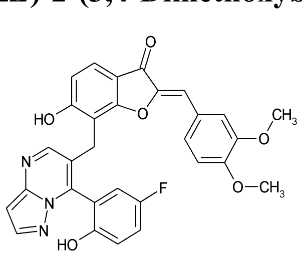

(2*Z*)-7-{[7-(5-Chloro-2-hydroxyphenyl)pyrazolo[1,5-*a*]pyrimidin-6-yl]methyl}-2-(3,4-dimethoxybenzylidene)-6-hydroxy-1-benzofuran-3(2*H*)-one (9c).

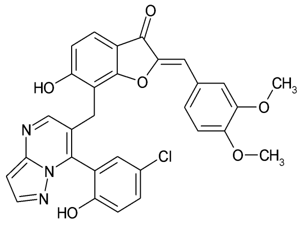

Yellow solid; yield 406 mg (73%); mp 273-275°C; ^{1}H NMR (400 MHz, DMSO-d_6): δ 11.06 (s, 1H), 9.99 (s, 1H), 8.66 (s, 1H), 8.02 (d, J = 2.4 Hz, 1H), 7.49 – 7.43 (m, 2H), 7.26 (dd, J = 8.4, 1.9 Hz, 1H), 7.08 (dd, J = 8.8, 2.7 Hz, 1H), 6.92 (d, J = 8.4 Hz, 1H), 6.88 (d, J = 2.7 Hz, 1H), 6.75 – 6.63 (m, 4H), 4.05 (d, J = 15.4 Hz, 1H), 3.92 (d, J = 15.4 Hz, 1H), 3.81 (s, 3H), 3.76 ppm (s, 3H); ^{13}C NMR (100 MHz, DMSO-d_6): δ 181.6, 165.2, 163.8, 154.0, 152.2, 150.1, 148.6, 147.5, 146.3, 143.8, 141.5, 130.6, 129.3, 125.0, 124.9, 123.8, 122.2, 119.1, 118.5, 117.5, 113.7, 112.8, 112.1, 111.7, 110.9, 109.4, 96.1, 55.6, 55.2, 22.8 ppm; MS (APCI): *m/z* 556.2 ([M+H]$^+$, 100). Anal. cacld (%) for $C_{30}H_{22}ClN_3O_6$: C, 64.81; H, 3.99; N, 7.56. Found (%): C, 64.96; H, 3.79; N, 7.68.

(2*Z*)-7-{[7-(5-Fluoro-2-hydroxyphenyl)pyrazolo[1,5-*a*]pyrimidin-6-yl]methyl}-6-hydroxy-2-(3,4,5-trimethoxybenzylidene)-1-benzofuran-3(2*H*)-one (9d).

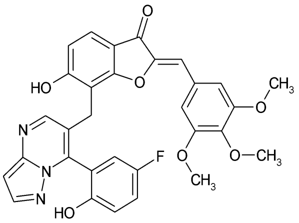

Isolated as solvate with *i*-PrOH after recrystallization from *i*-PrOH. Yellow solid; yield 279 mg (49%); mp 238-240°C; °C; ^{1}H NMR (400 MHz, DMSO-d_6): δ 11.07 (s, 1H), 9.56 (s, 1H), 8.66 (s, 1H), 8.00 (d, J = 2.3 Hz, 1H), 7.47 (d, J = 8.4 Hz, 1H), 7.23 (s, 2H), 6.92 – 6.81 (m, 1H), 6.74 – 6.57 (m, 5H), 4.04 (d, J = 15.1 Hz, 1H), 3.90 (d, J = 15.1 Hz, 1H), 3.80 (s, 6H), 3.73 ppm (s, 3H); ^{13}C NMR (125 MHz, DMSO-d_6): δ 181.7, 165.3, 164.1, 154.8 (d, J_{C-F} = 234.9 Hz), 152.8, 152.3, 151.3 (d, J_{C-F} = 1.8 Hz), 147.4, 146.9, 143.7, 141.7, 138.8, 127.6, 123.9, 118.9, 117.5 (d, J_{C-F} = 8.5 Hz), 117.3 (d, J_{C-F} = 22.7 Hz), 116.7 (d, J_{C-F} = 8.1 Hz), 116.1 (d, J_{C-F} = 24.1 Hz), 112.4, 112.2, 110.6, 109.6, 108.5, 95.9, 60.1, 55.7, 22.6 ppm; ^{19}F NMR (376 MHz, DMSO-d_6): δ -126.3 ppm; MS (APCI): *m/z* 570.2 ([M+H]$^+$, 100). Anal. cacld (%) for $C_{31}H_{24}FN_3O_7{\cdot}0.5C_3H_8O$: C, 65.10; H, 4.71; N, 7.01. Found (%): C, 65.21; H, 4.43; N, 7.14.

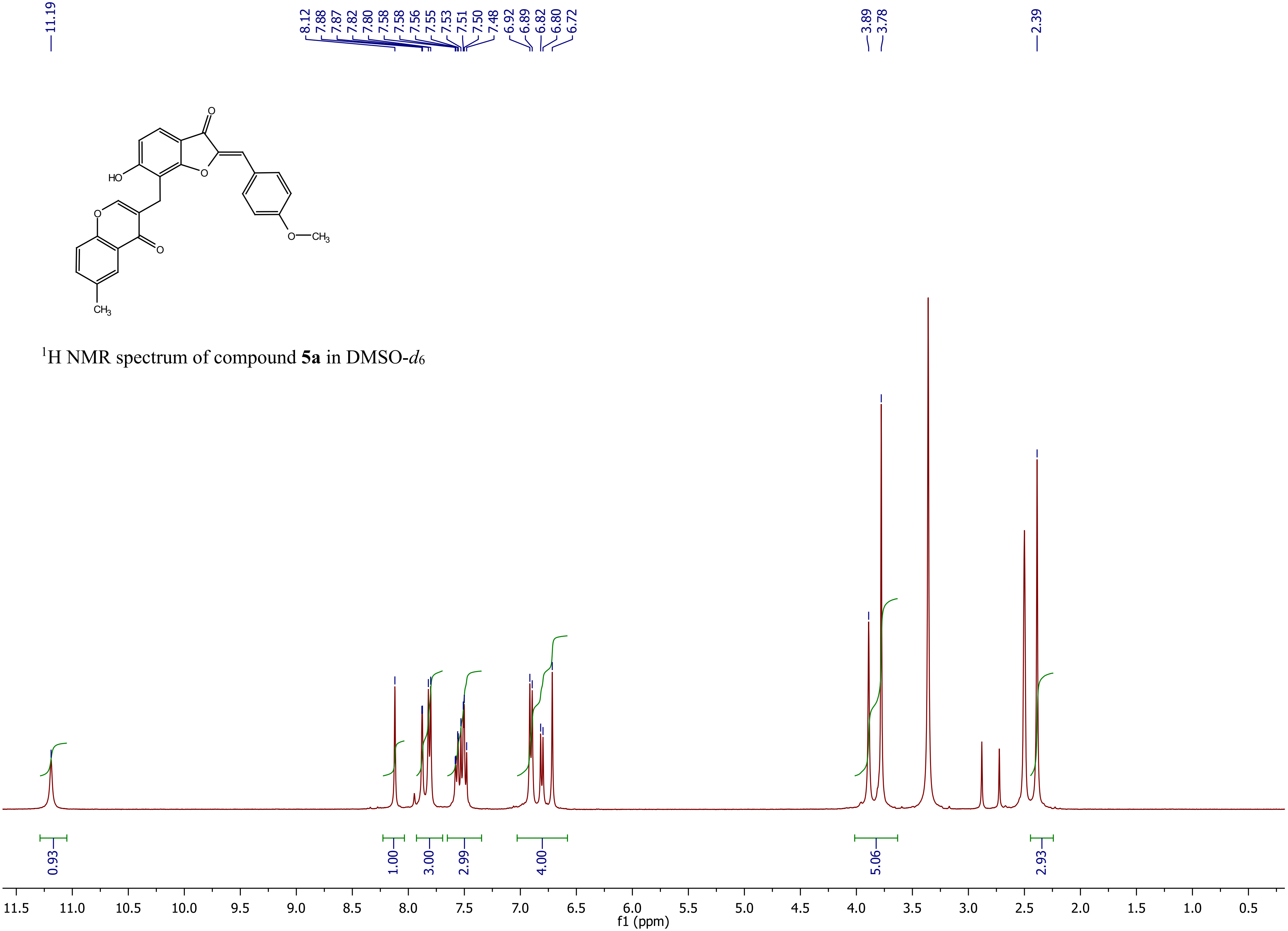

^{1}H NMR spectrum of compound **5a** in DMSO-d_6

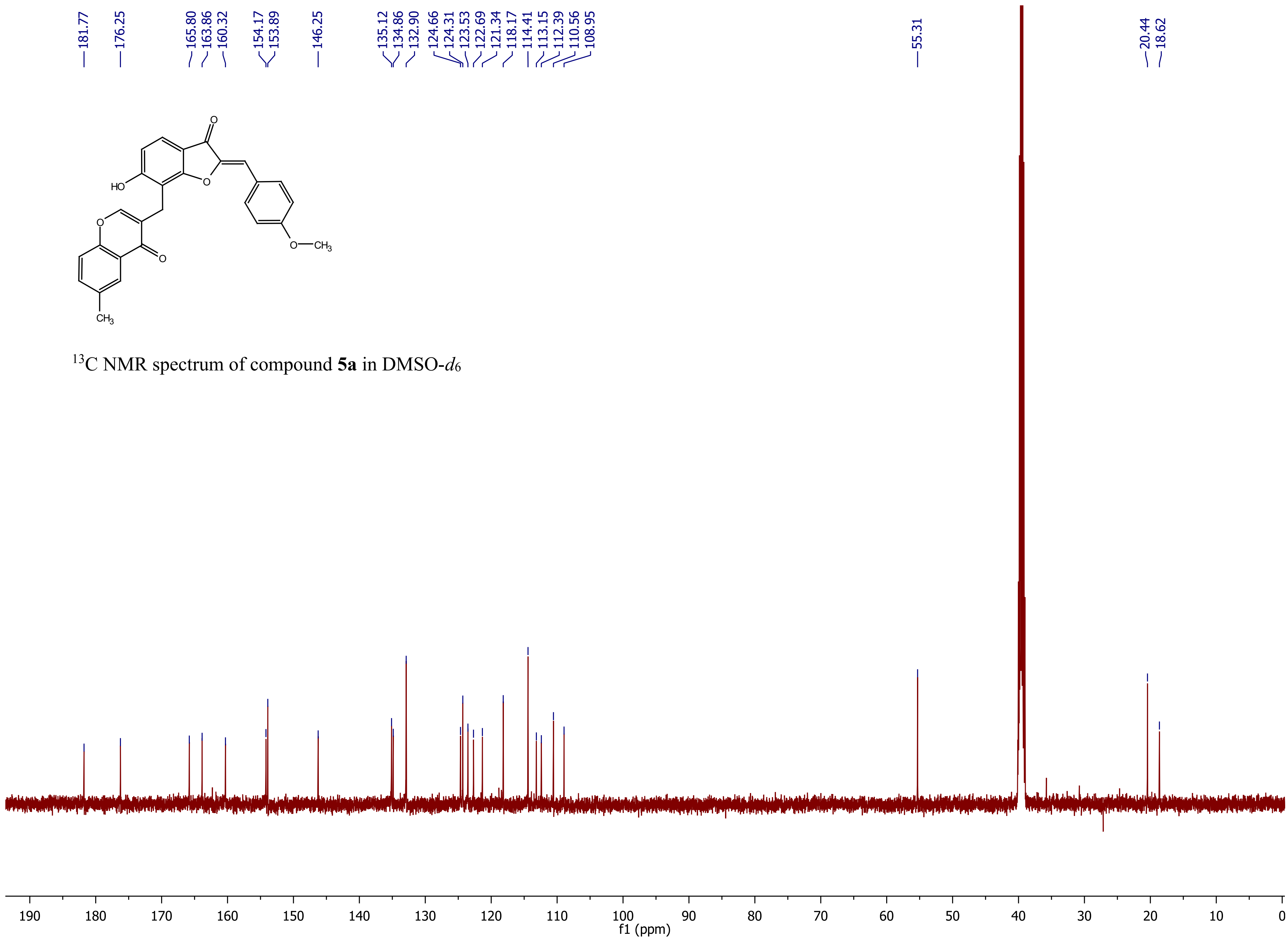

^{13}C NMR spectrum of compound **5a** in DMSO-d_6

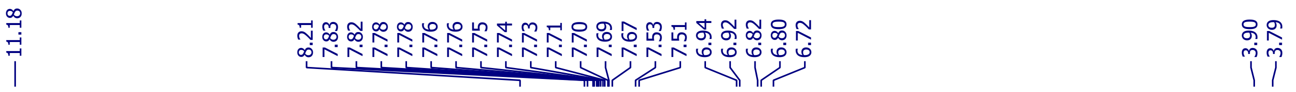

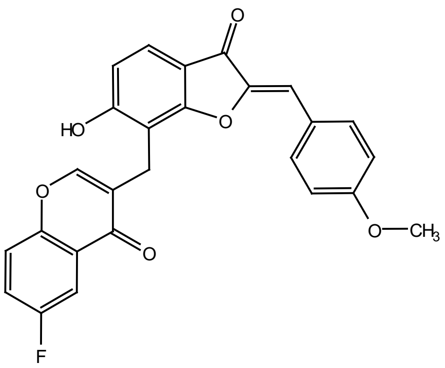

^{1}H NMR spectrum of compound **5b** in DMSO-d_6

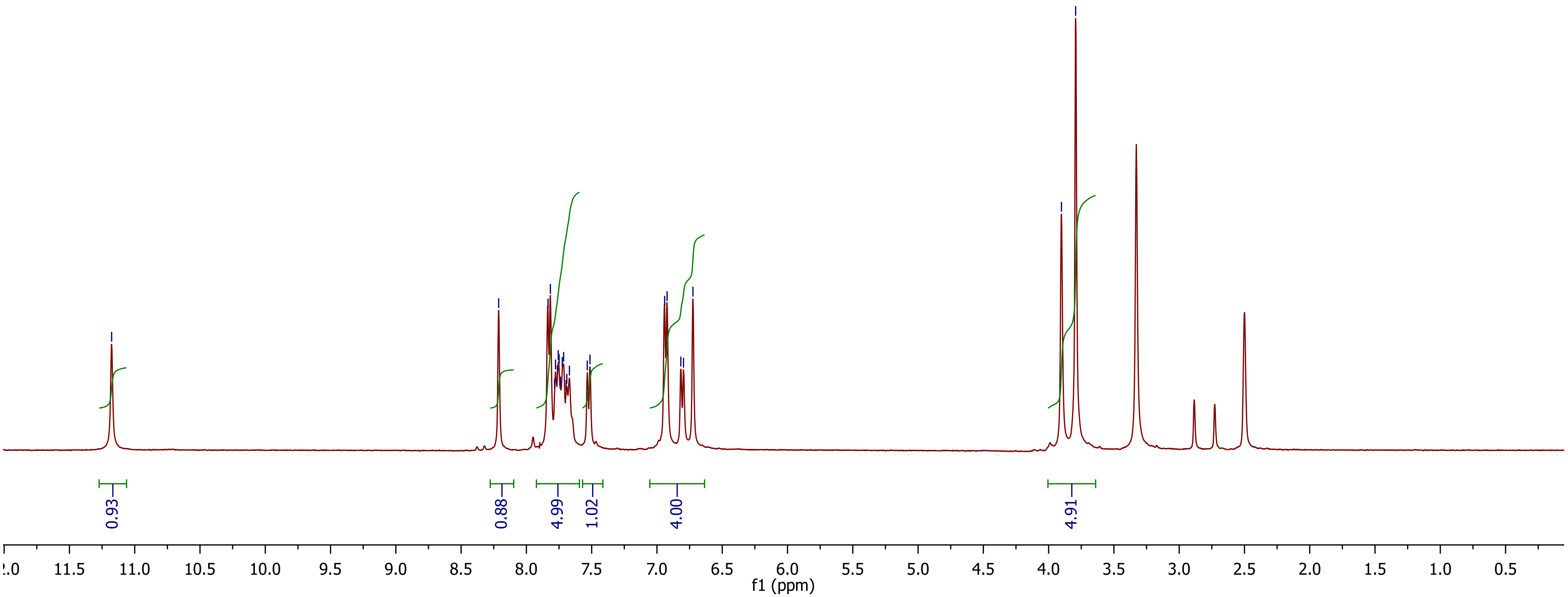

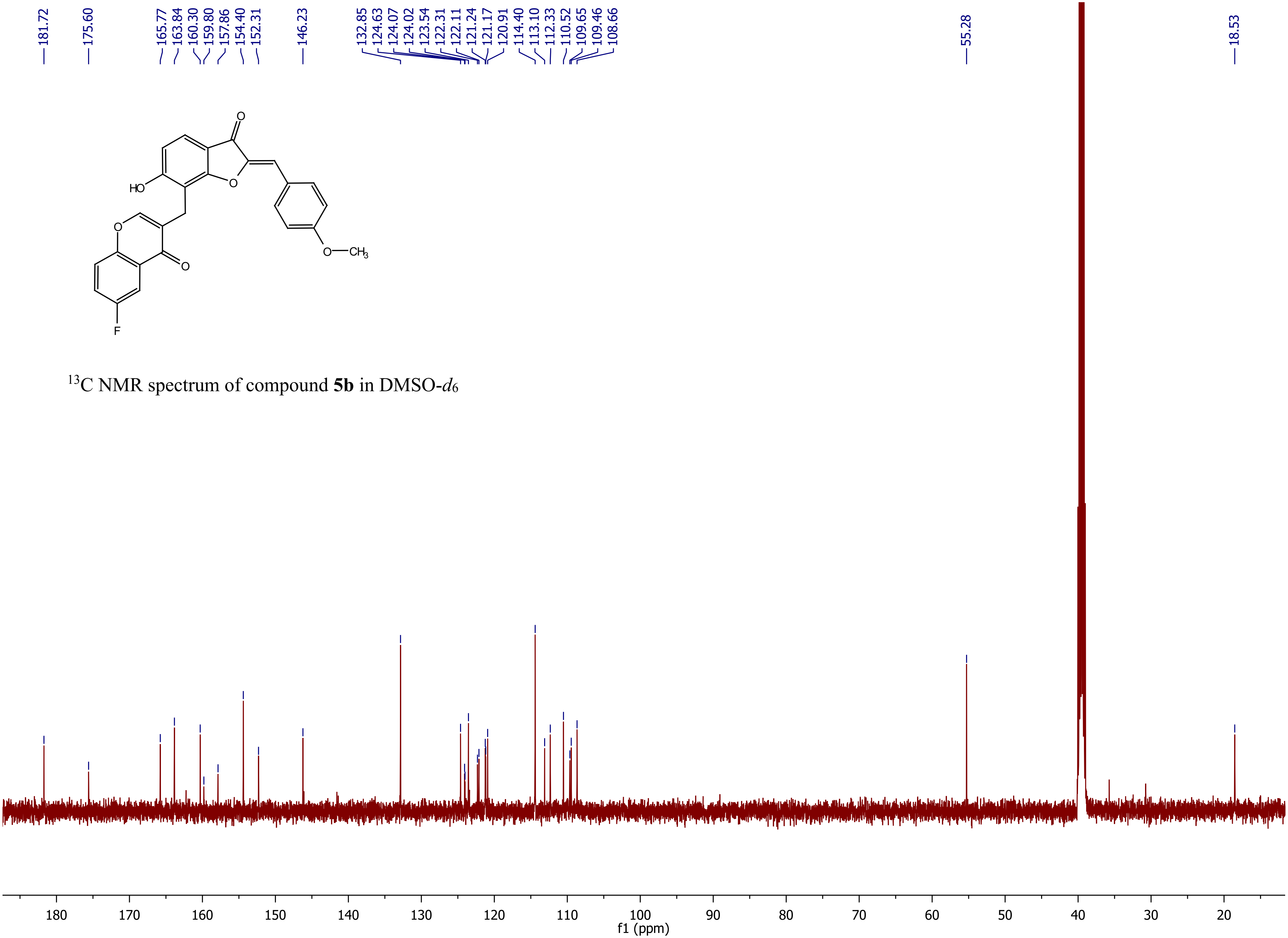

^{13}C NMR spectrum of compound **5b** in DMSO-d_6

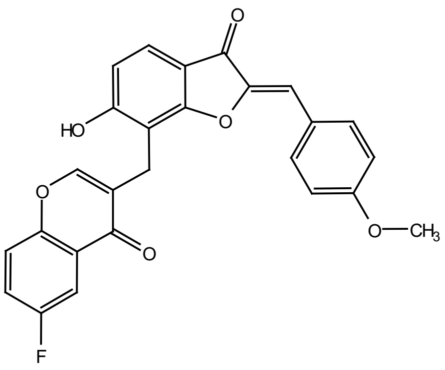

^{19}F NMR spectrum of compound **5b** in DMSO-*d6*

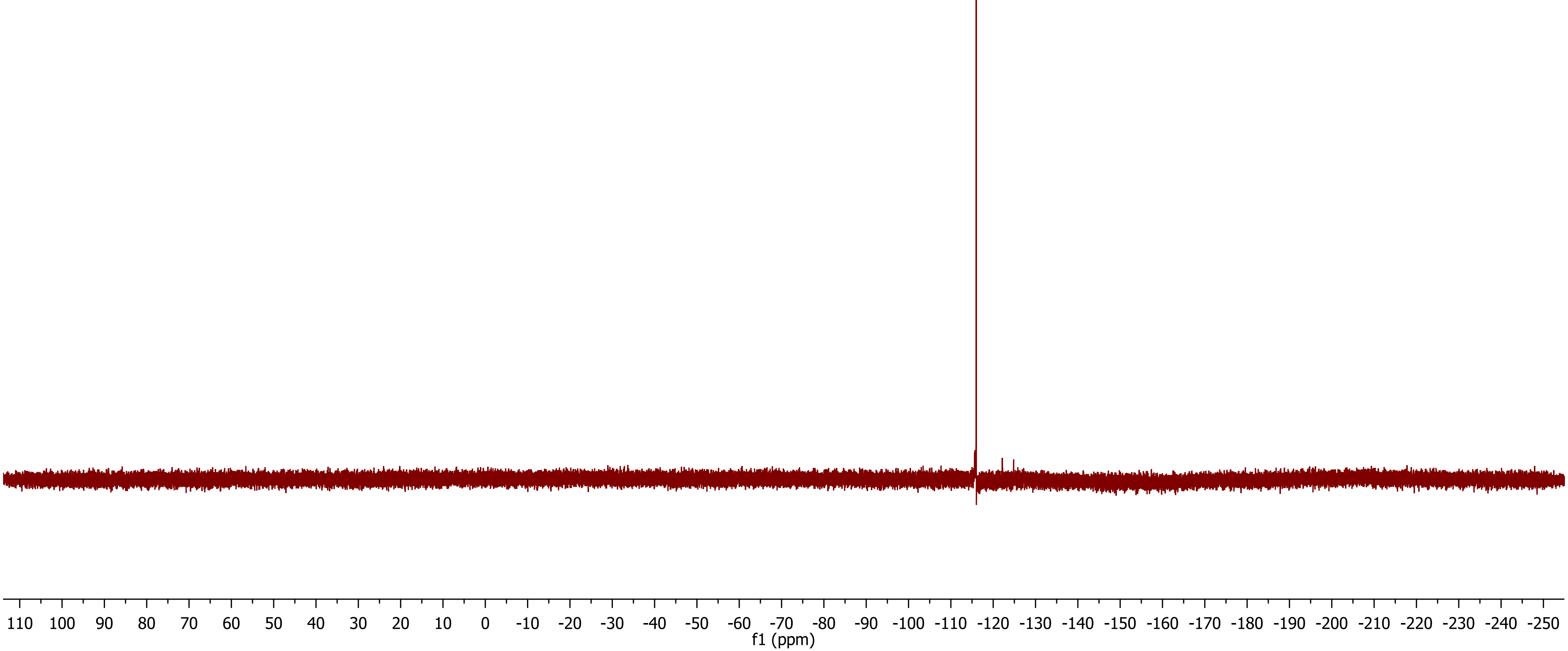

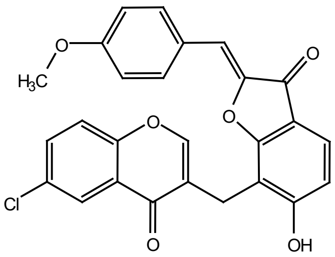

^{1}H NMR spectrum of compound **5c** in DMSO-*d*6

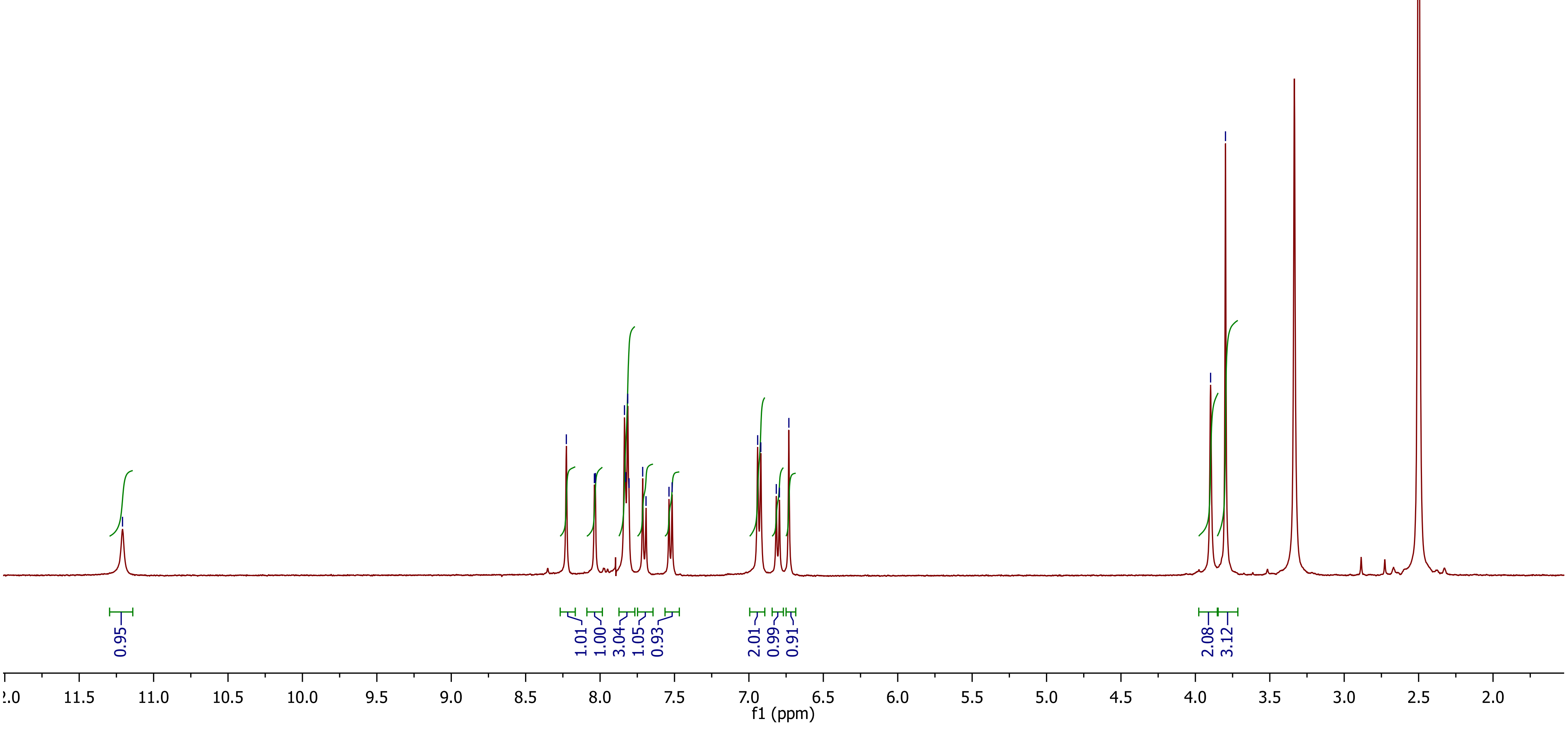

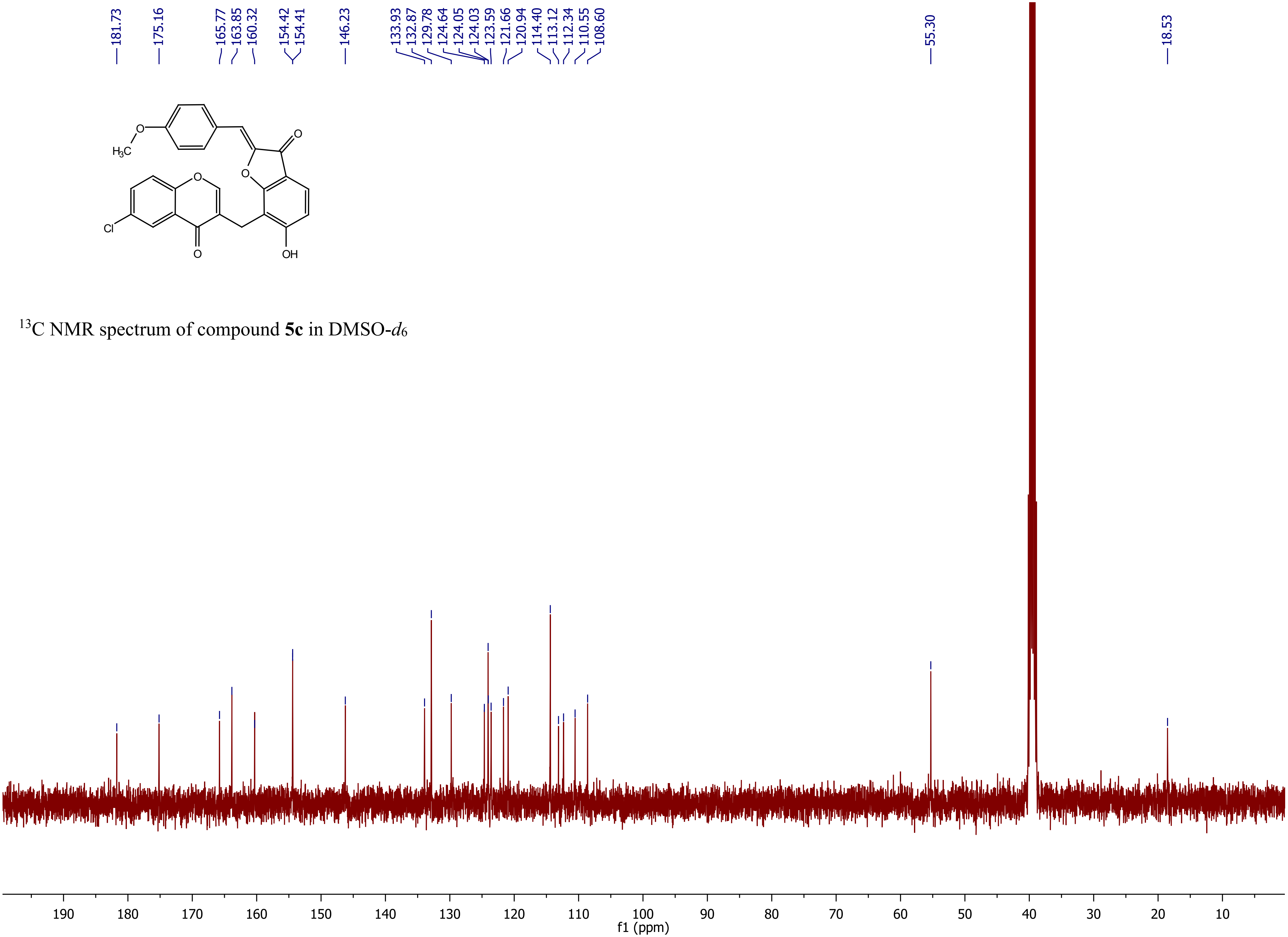

^{13}C NMR spectrum of compound **5c** in DMSO-d_6

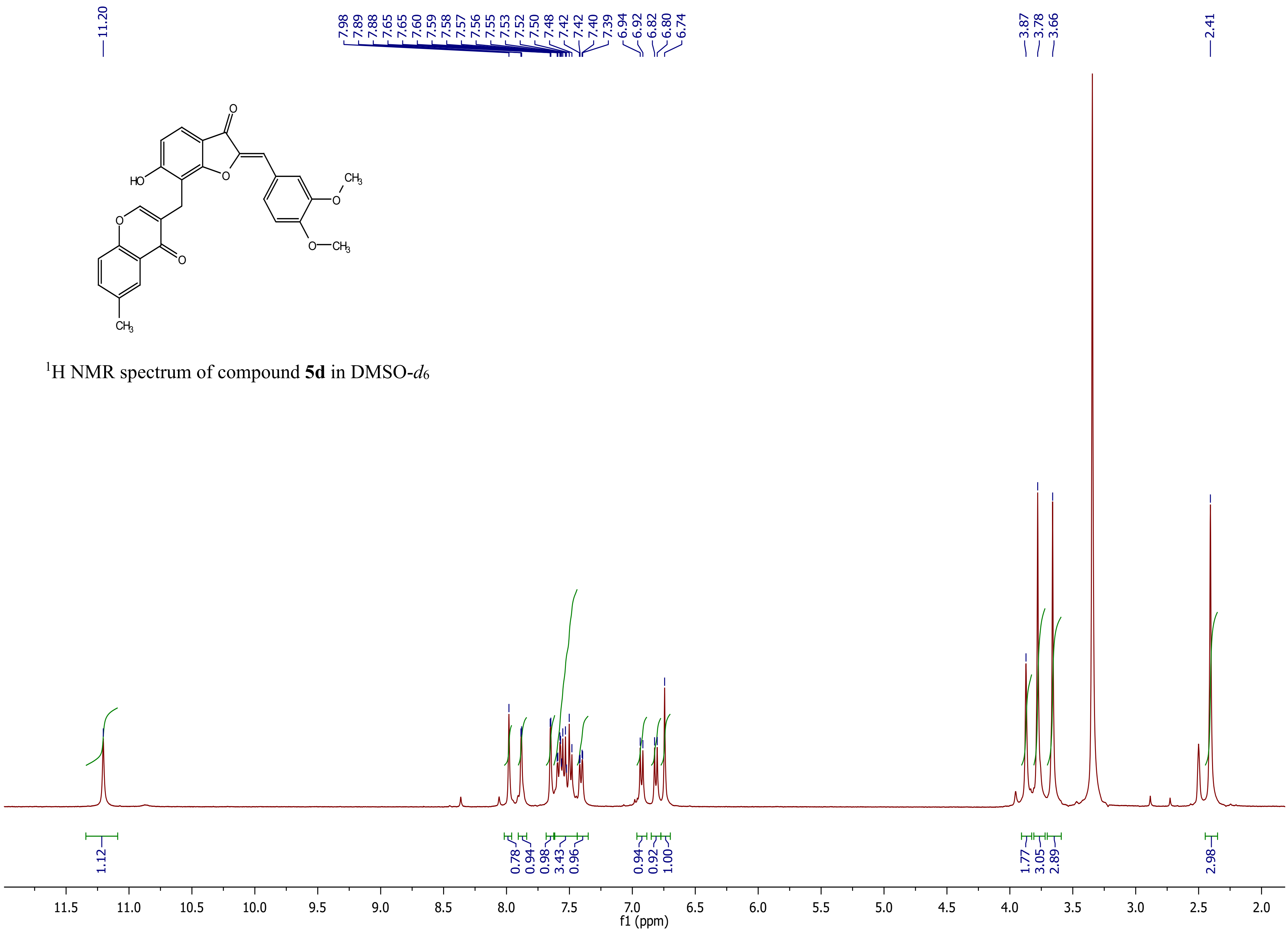

^{1}H NMR spectrum of compound **5d** in DMSO-d_6

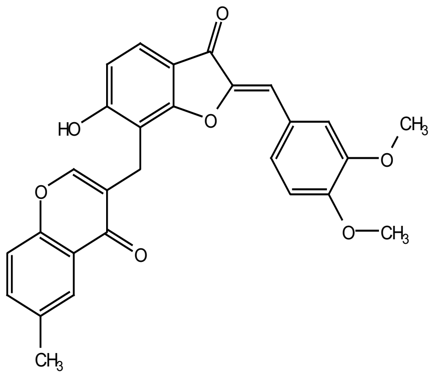

^{13}C NMR spectrum of compound **5d** in DMSO-d_6

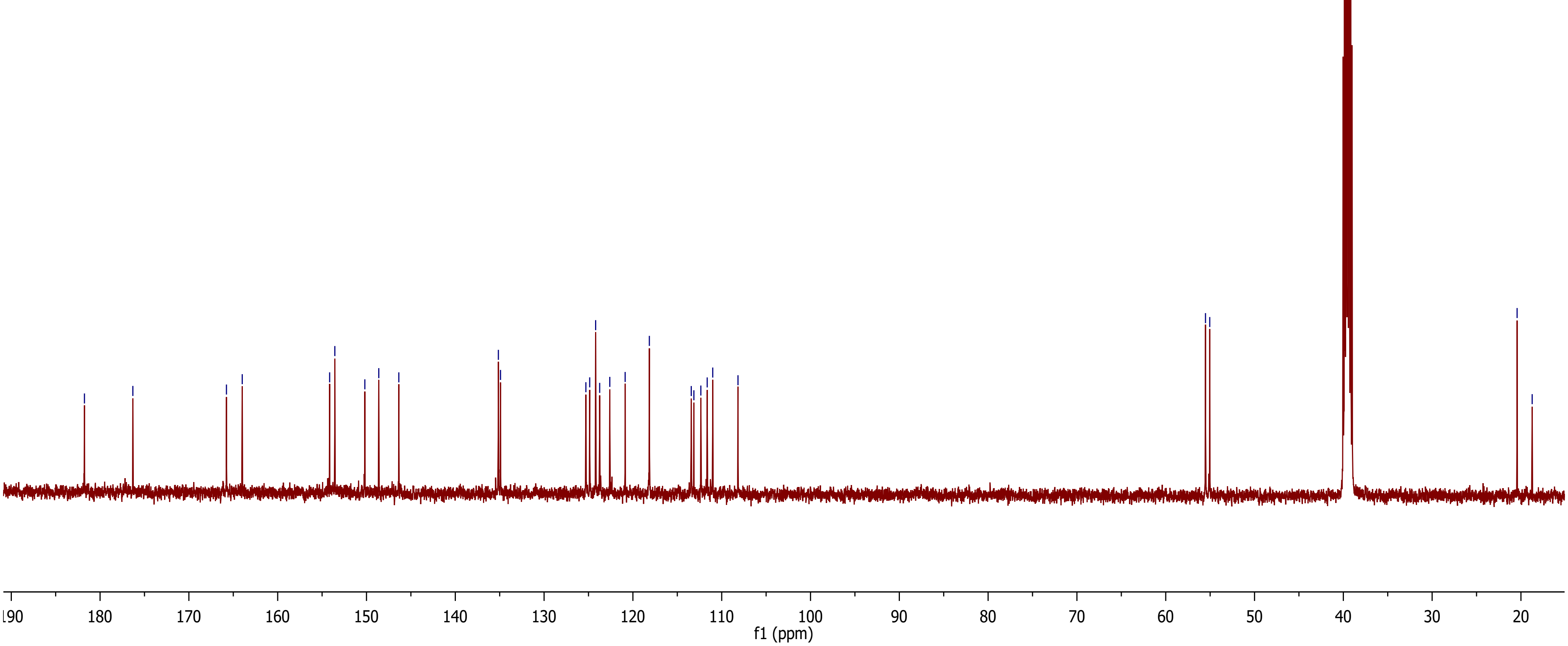

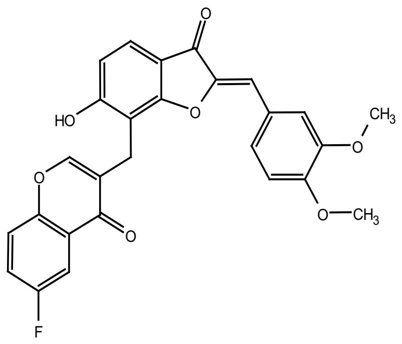

^{1}H NMR spectrum of compound **5e** in DMSO-d_6

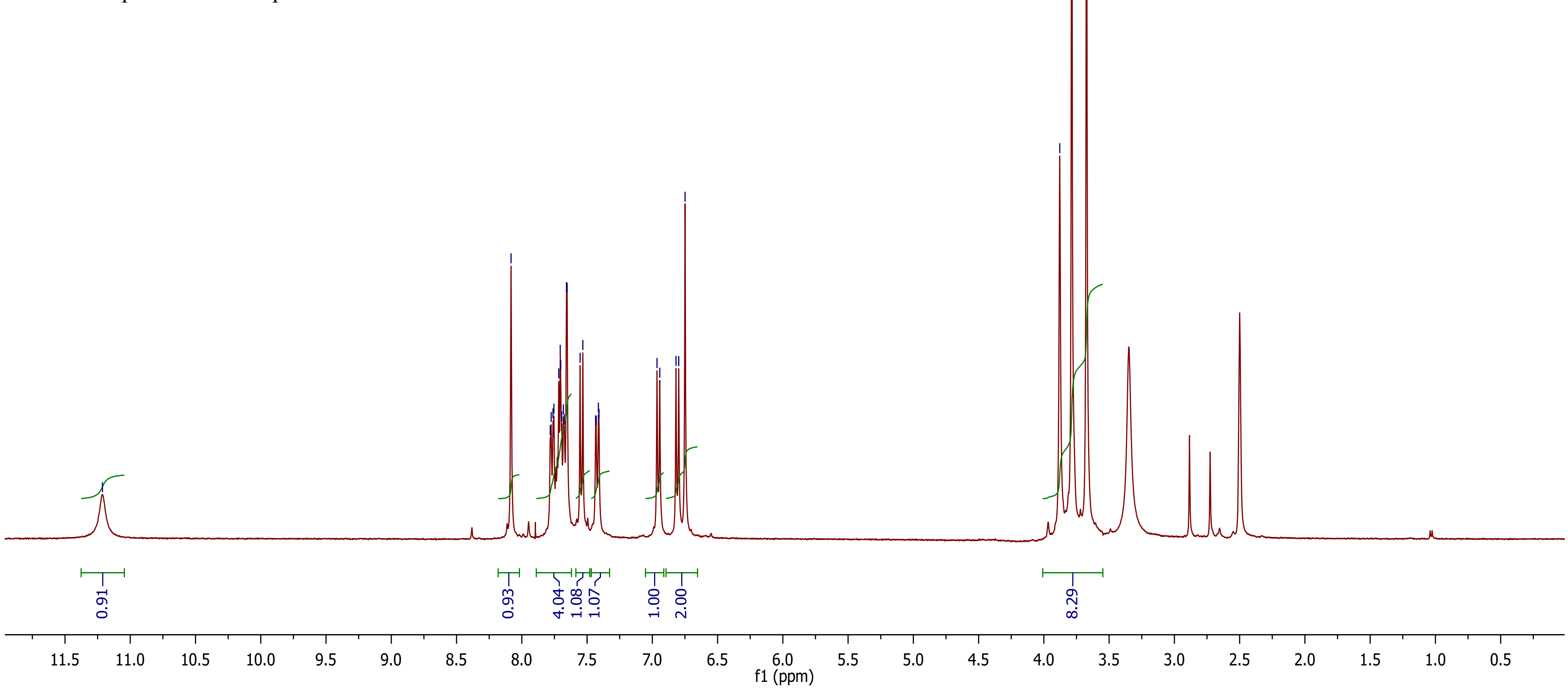

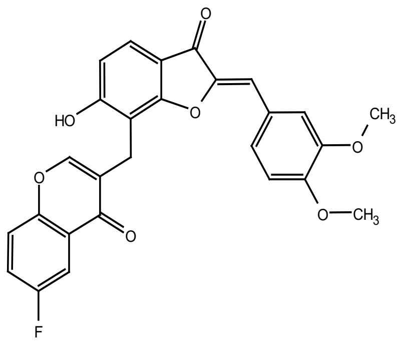

^{13}C NMR spectrum of compound **5e** in DMSO-d_6

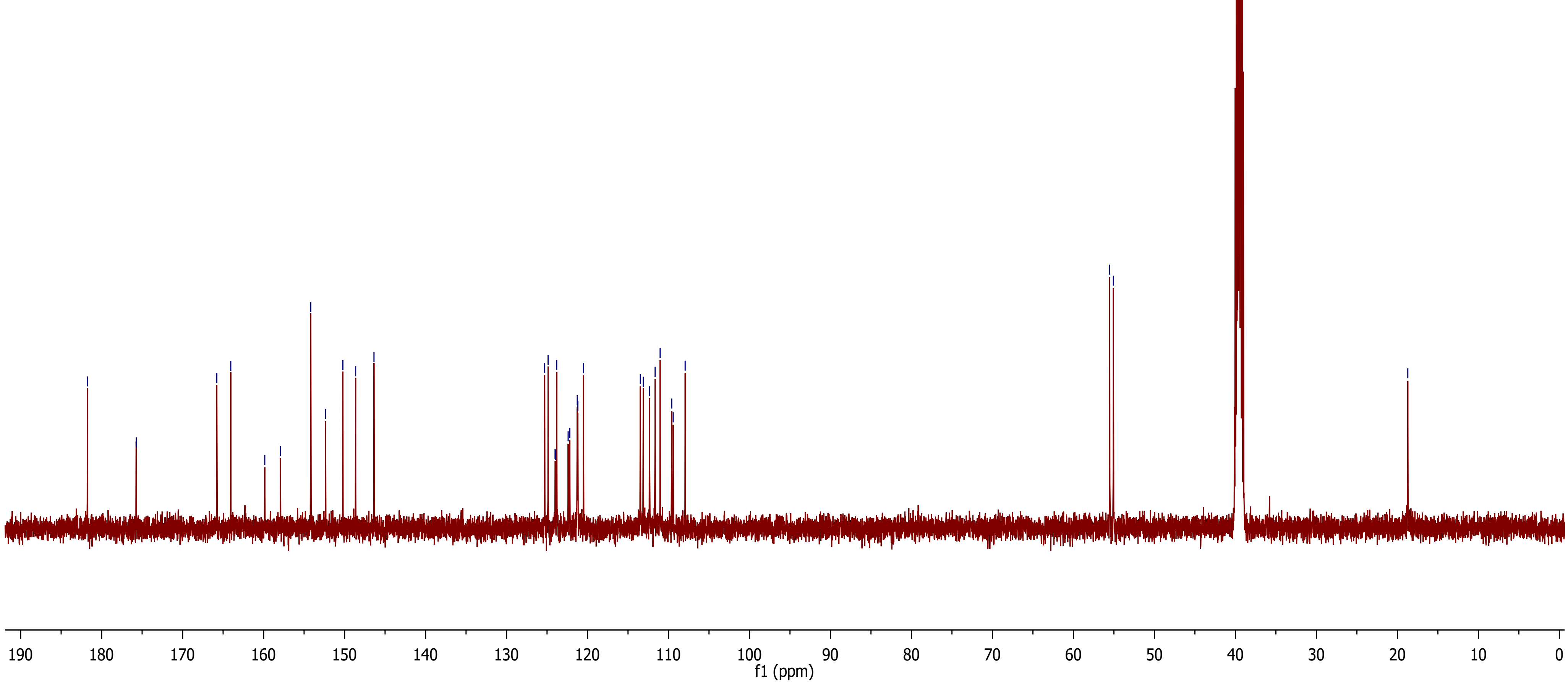

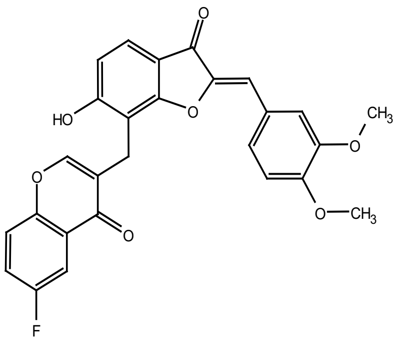

^{19}F NMR spectrum of compound **5e** in DMSO-d_6

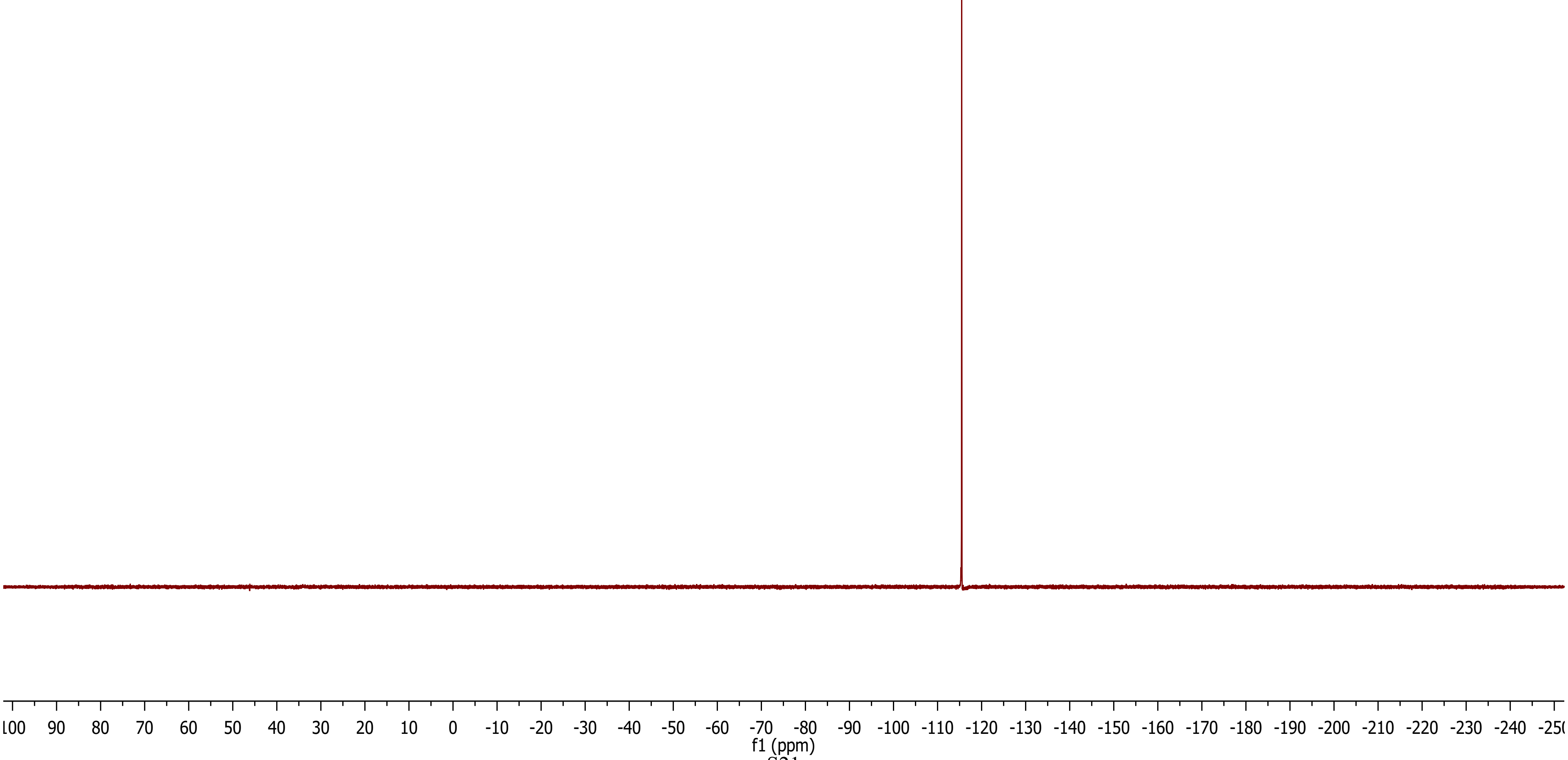

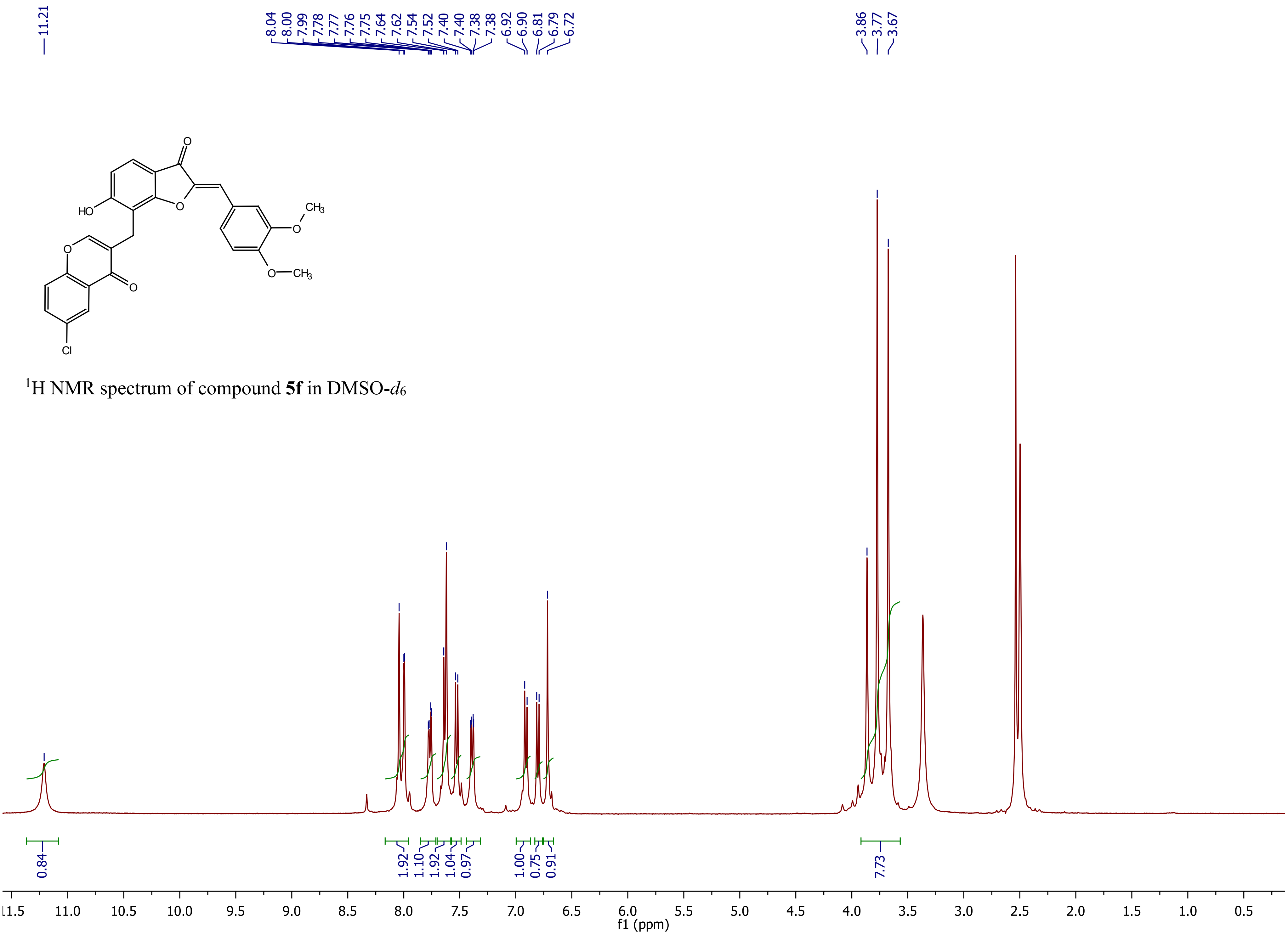

^{1}H NMR spectrum of compound **5f** in DMSO-d_6

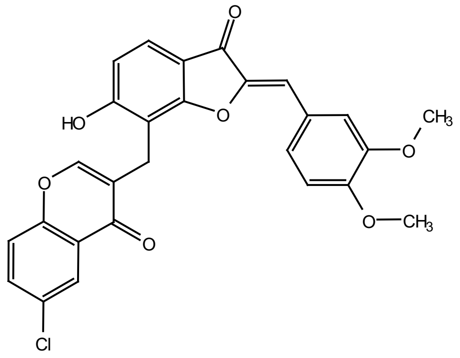

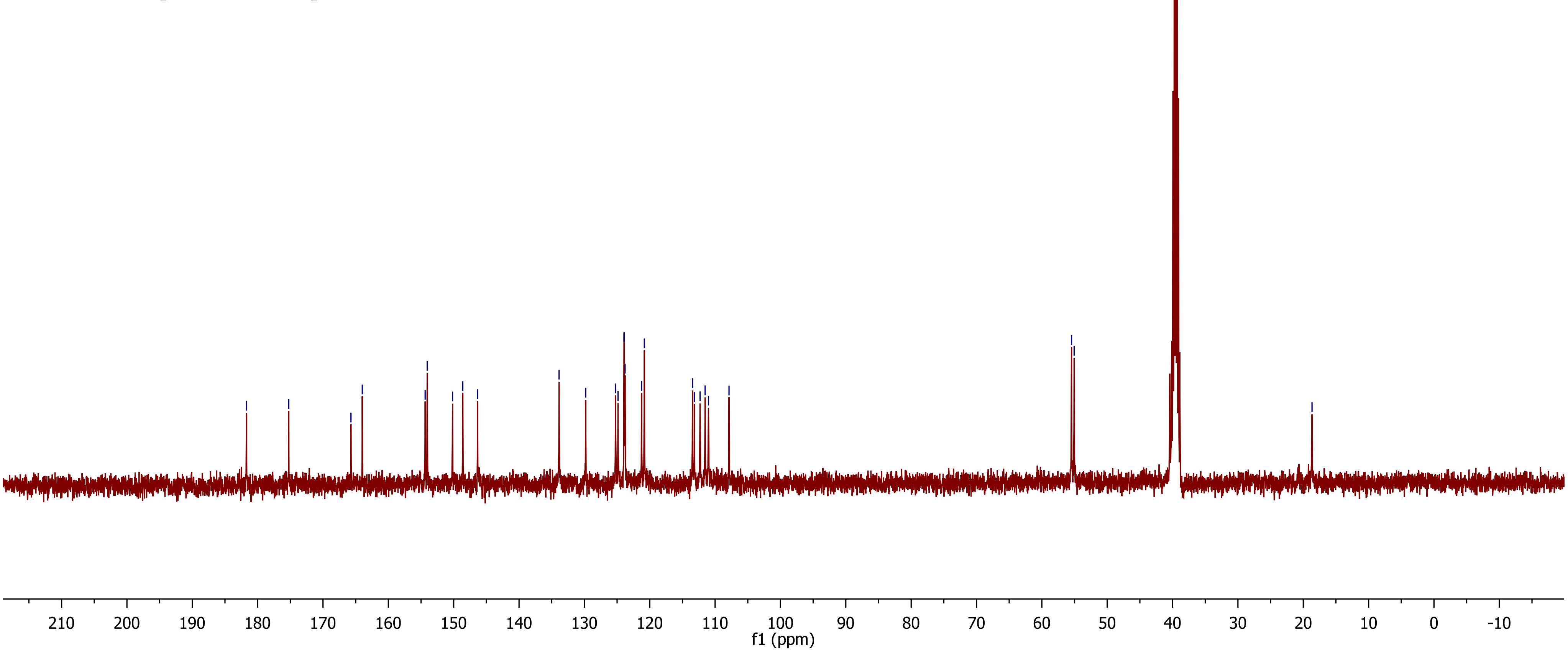

^{13}C NMR spectrum of compound **5f** in DMSO-d_6

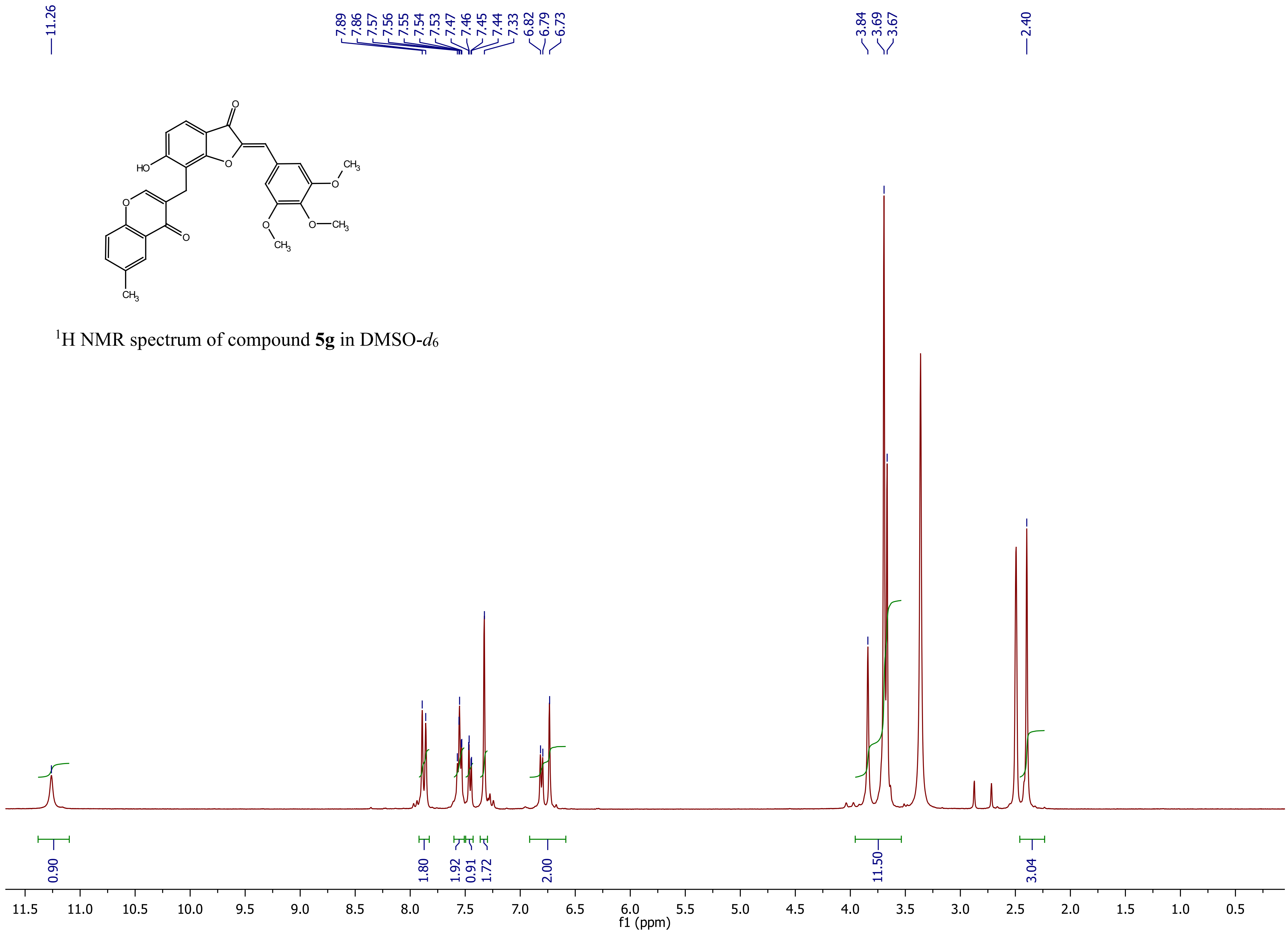

^{1}H NMR spectrum of compound **5g** in DMSO-d_6

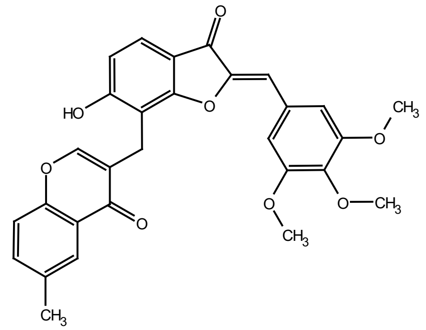

^{13}C NMR spectrum of compound **5g** in DMSO-d_6

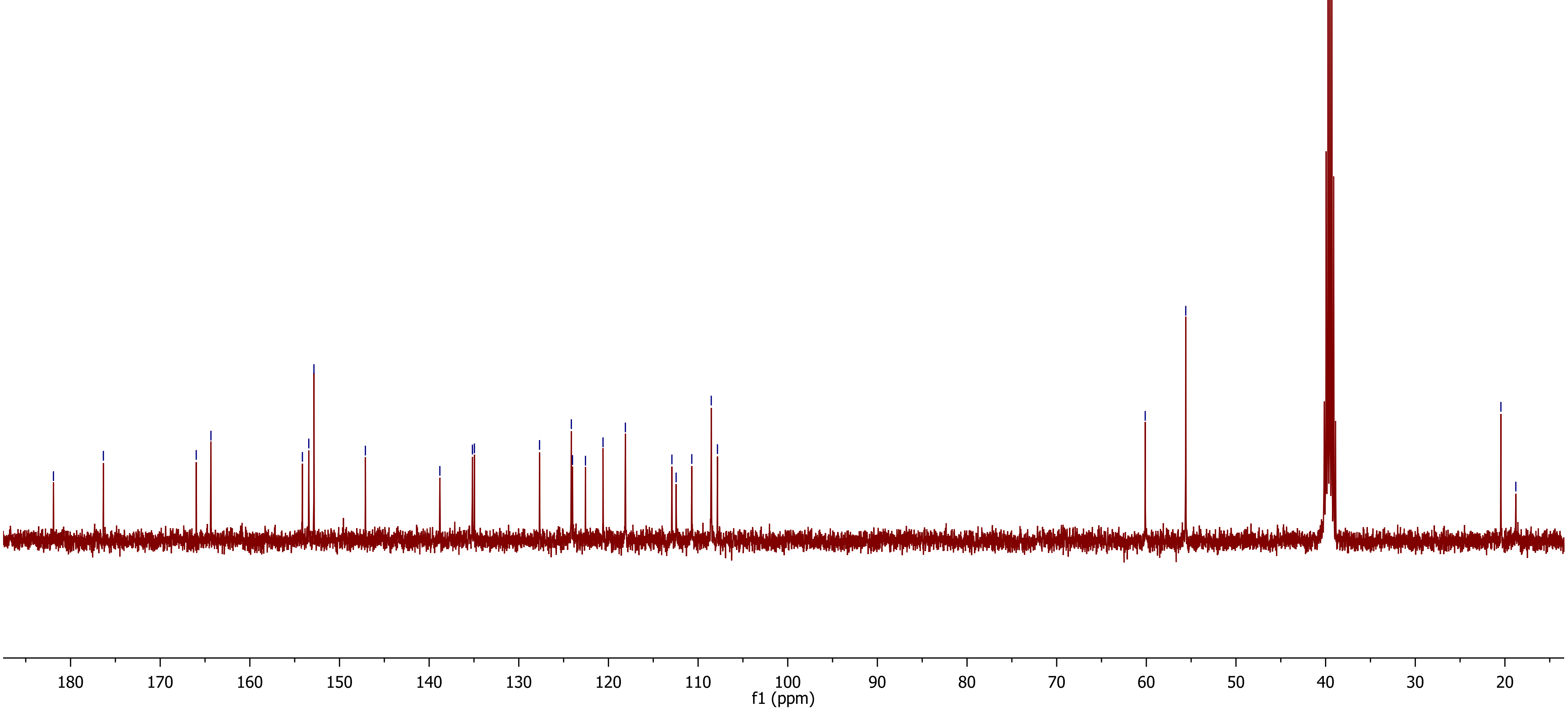

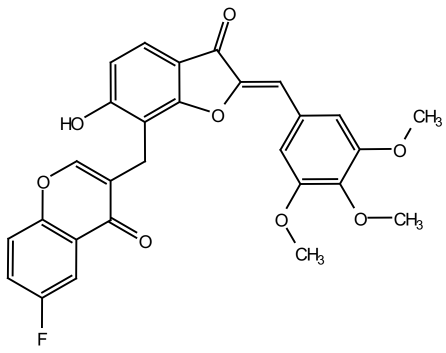

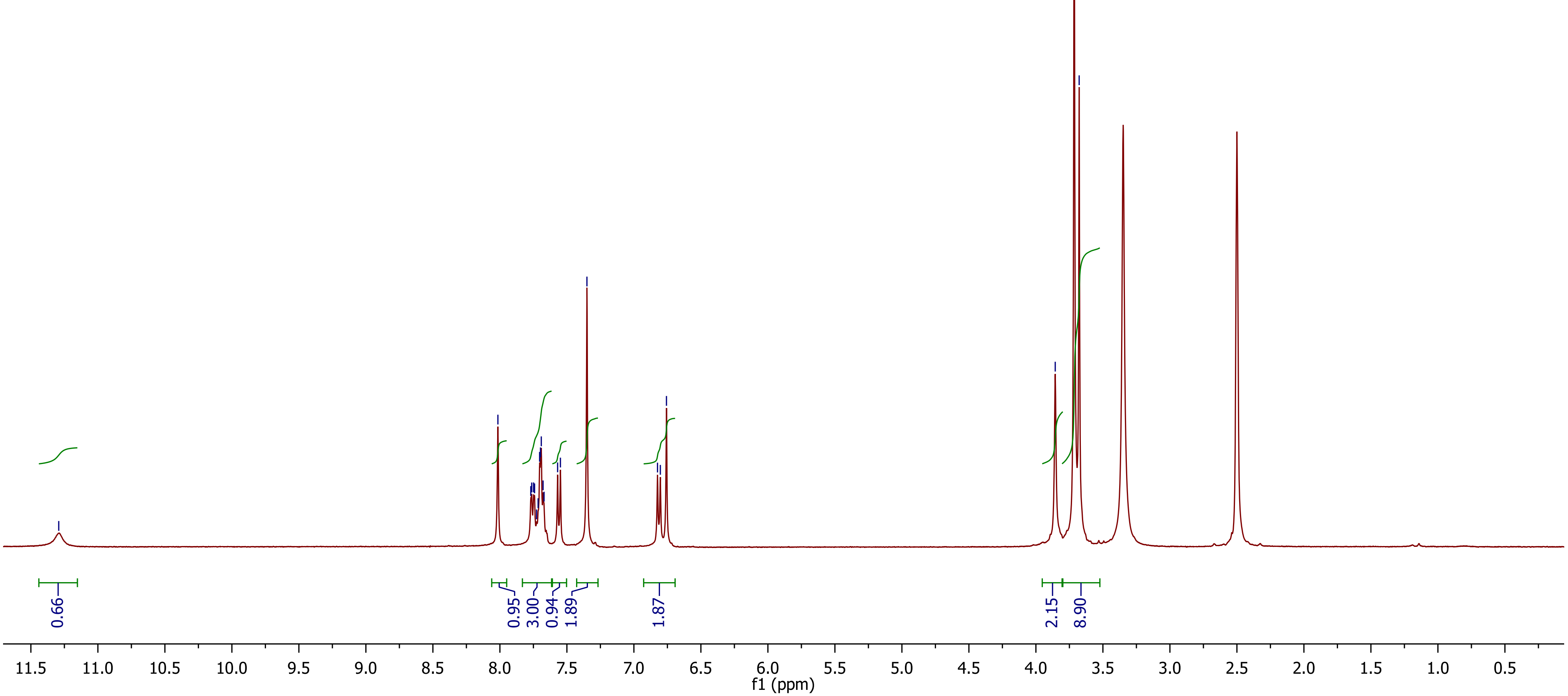

^{1}H NMR spectrum of compound **5h** in DMSO-d_6

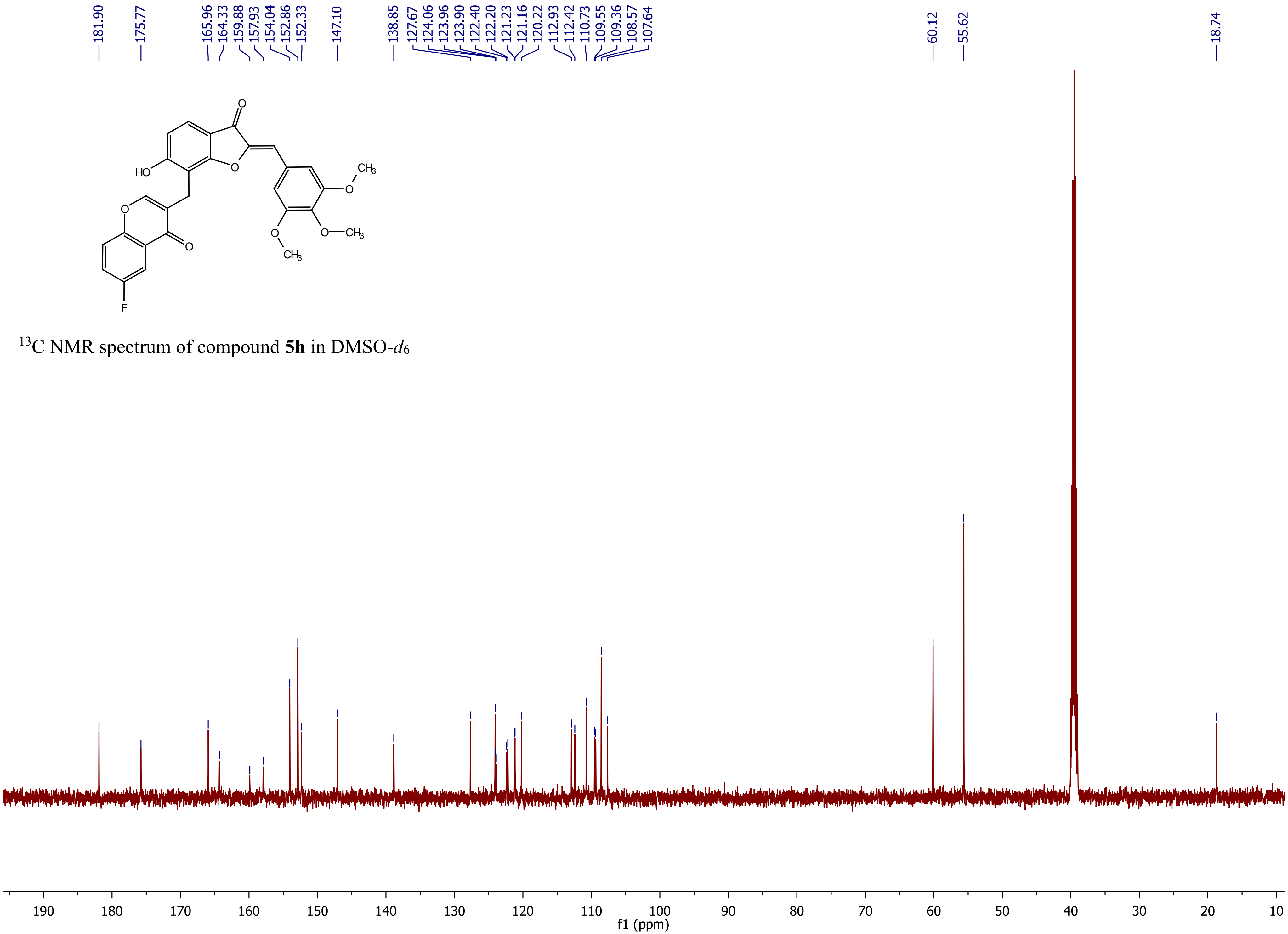

^{13}C NMR spectrum of compound **5h** in DMSO-d_6

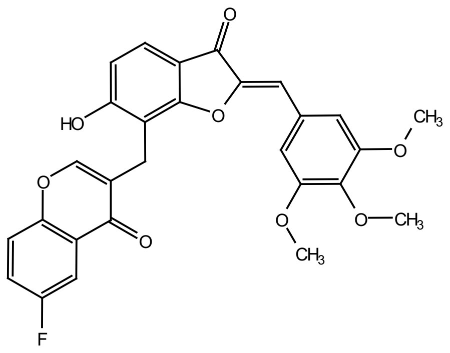

^{19}F NMR spectrum of compound **5h** in DMSO-d_6

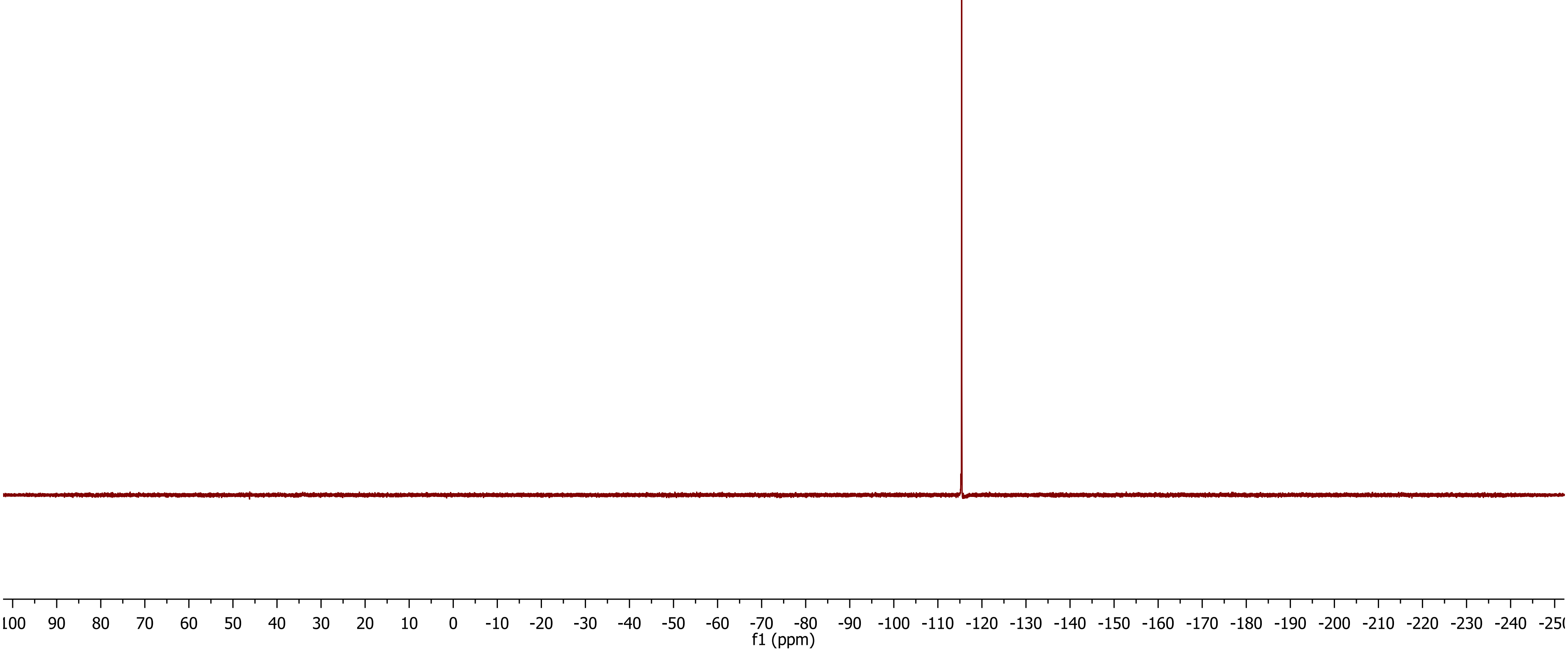

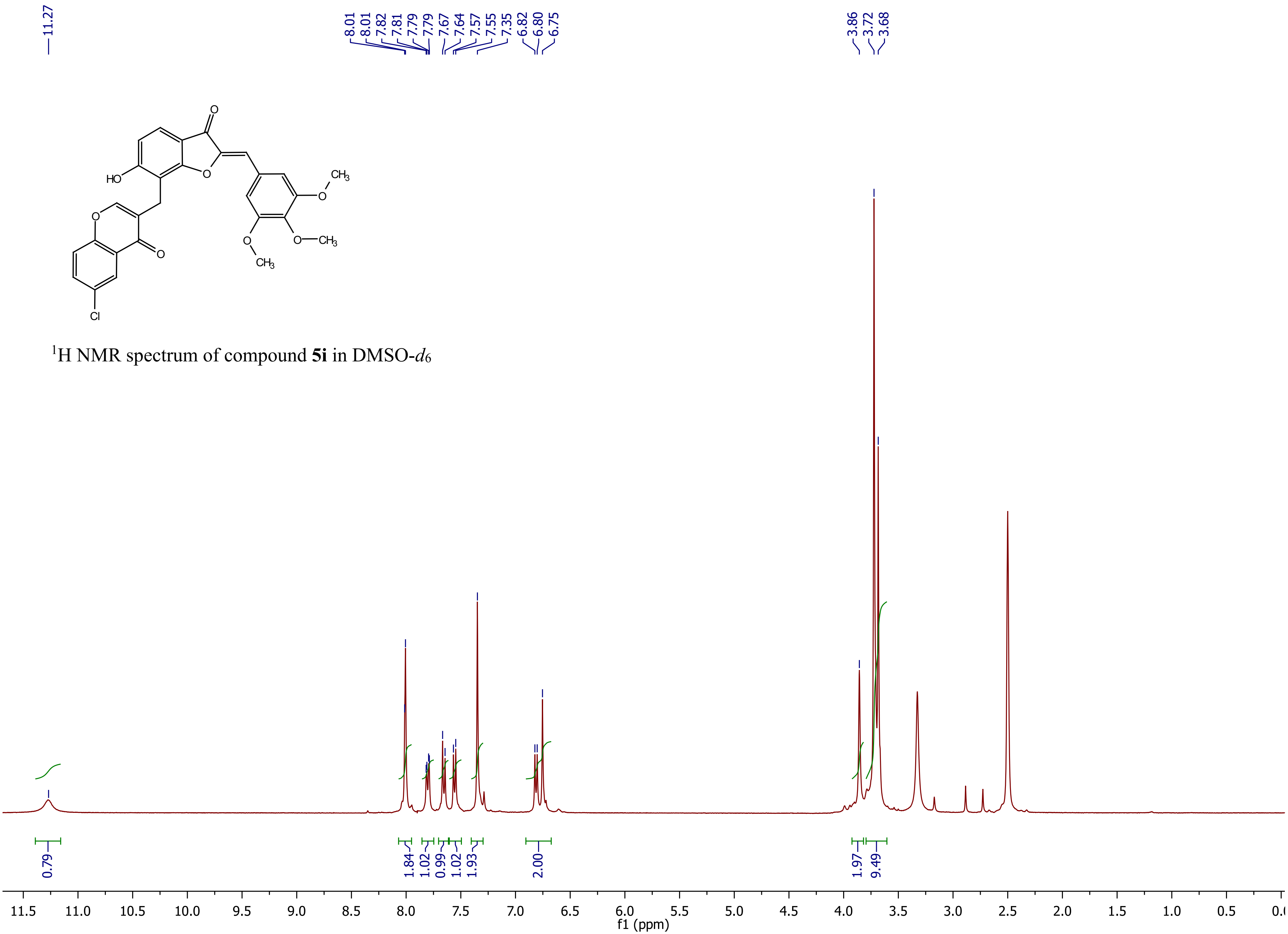

^{1}H NMR spectrum of compound **5i** in DMSO-d_6

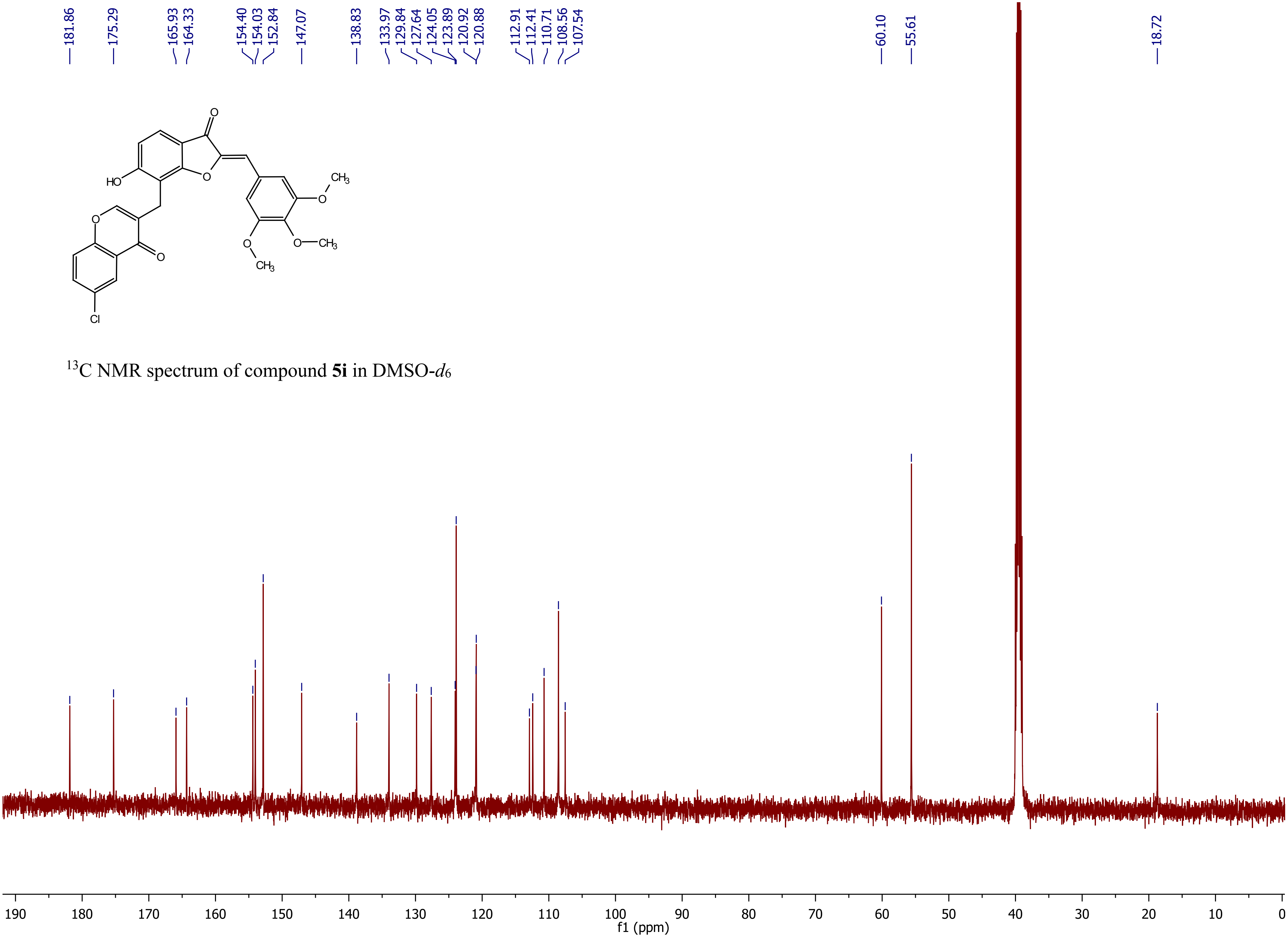

^{13}C NMR spectrum of compound **5i** in DMSO-d_6

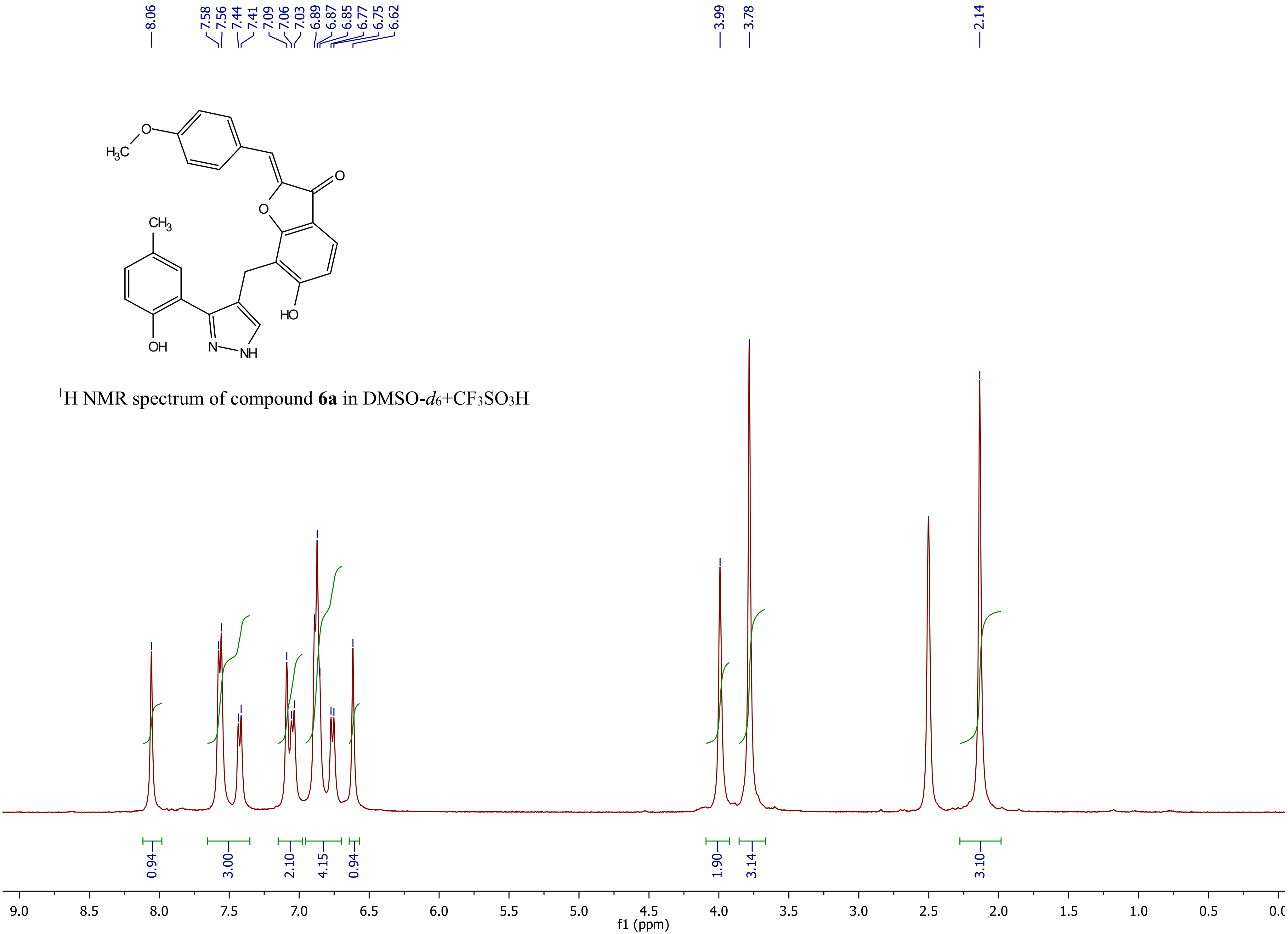

^{1}H NMR spectrum of compound **6a** in DMSO-d_6+CF_3SO_3H

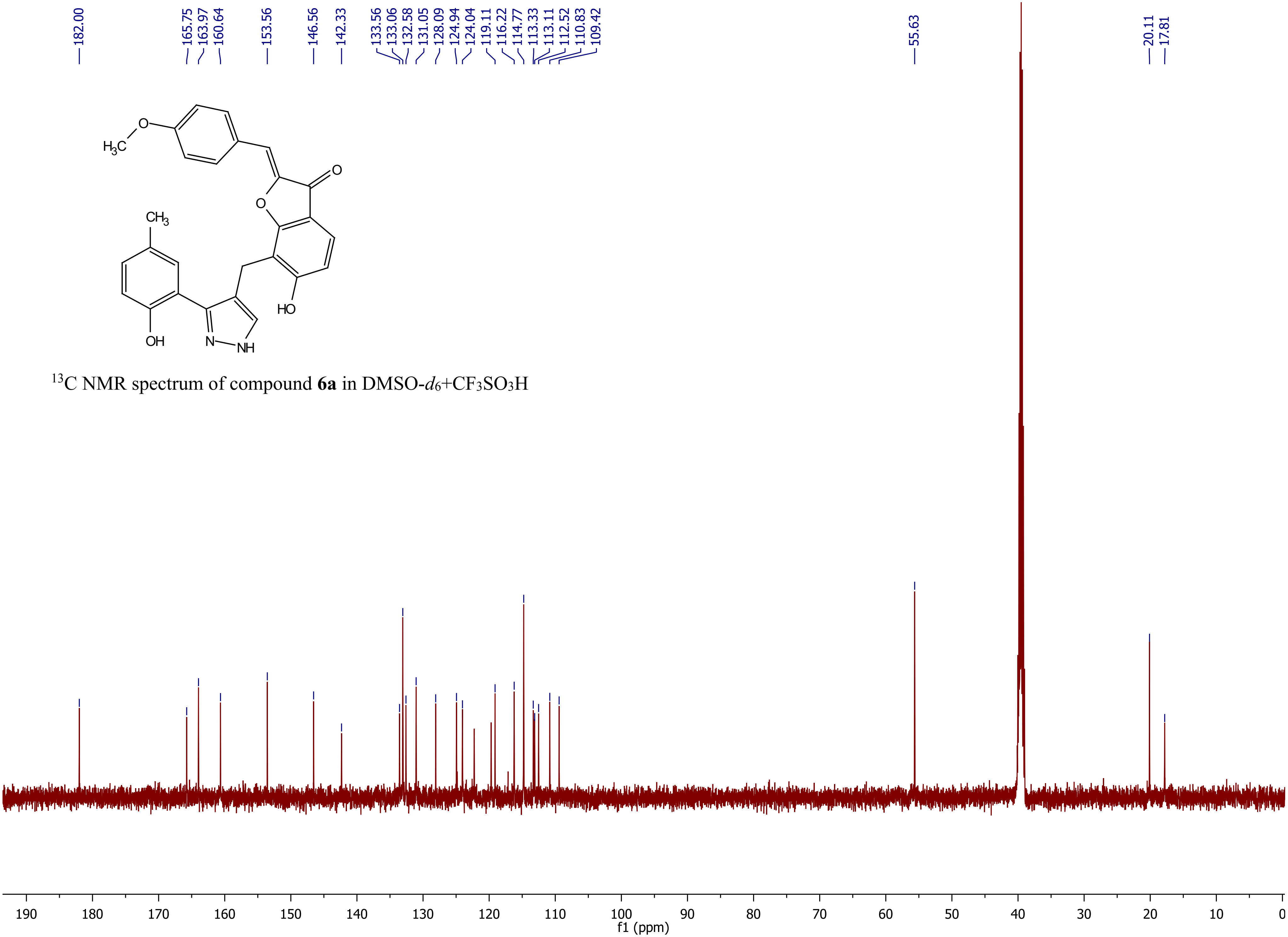

^{13}C NMR spectrum of compound **6a** in DMSO-d_6+CF_3SO_3H

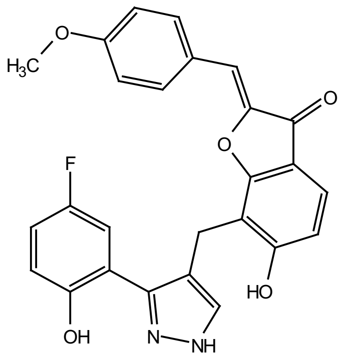

^{1}H NMR spectrum of compound **6b** in DMSO-d_6+CF_3SO_3H

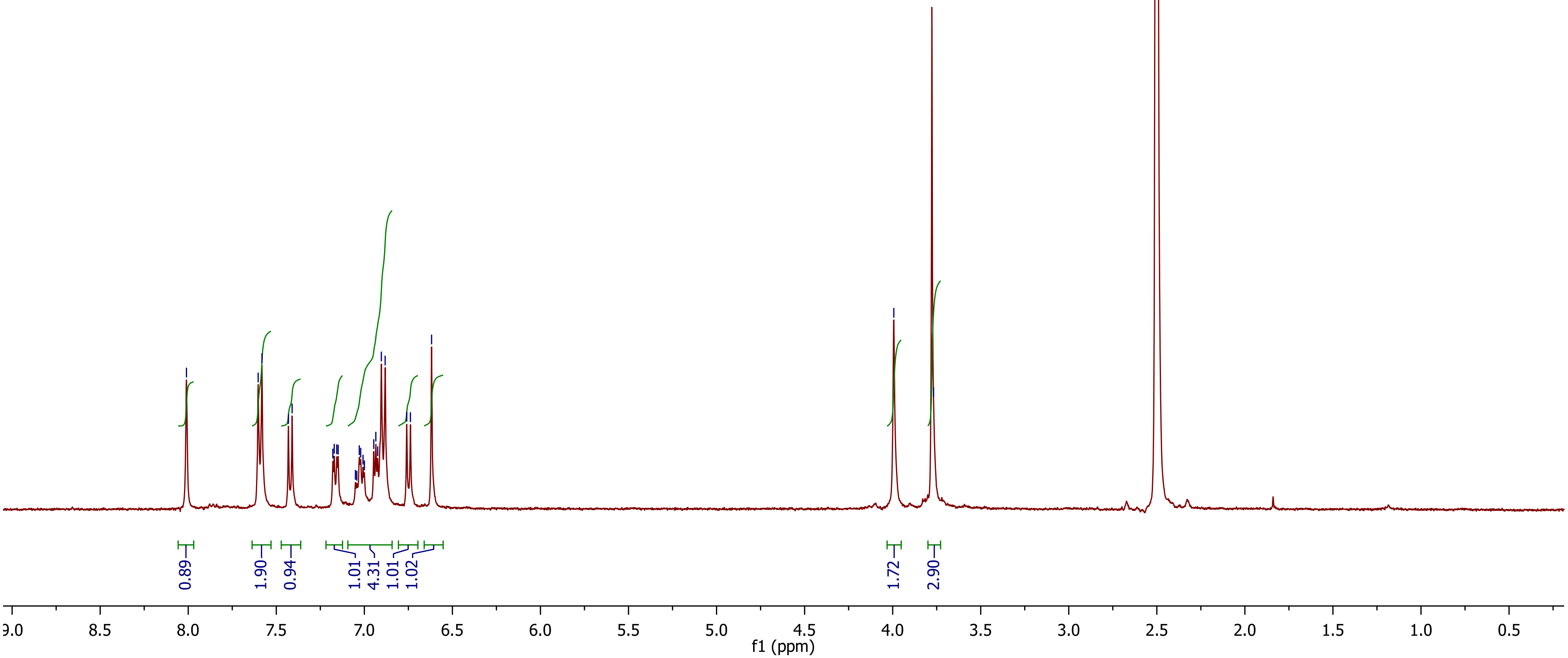

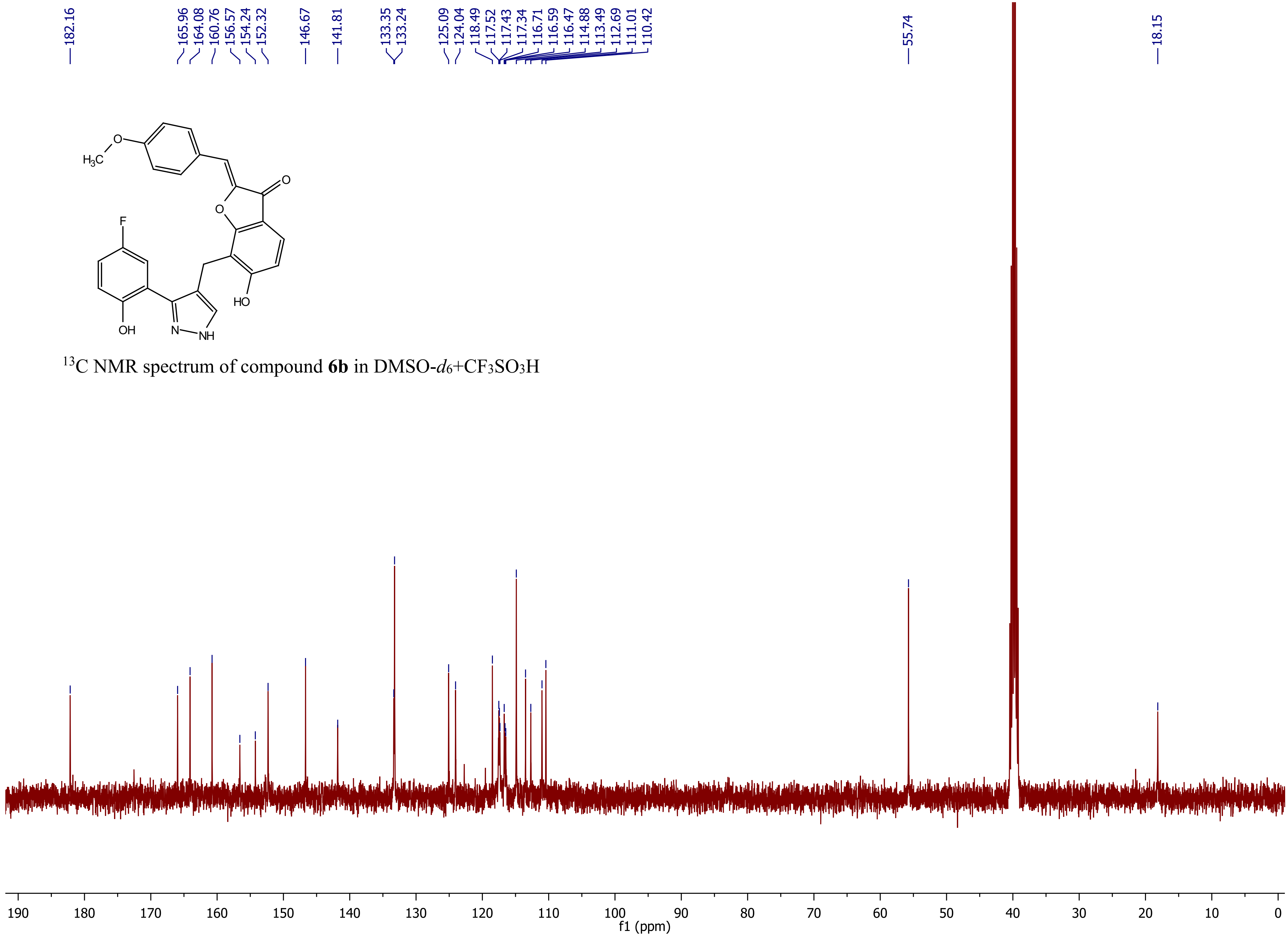

^{13}C NMR spectrum of compound **6b** in DMSO-d_6+CF_3SO_3H

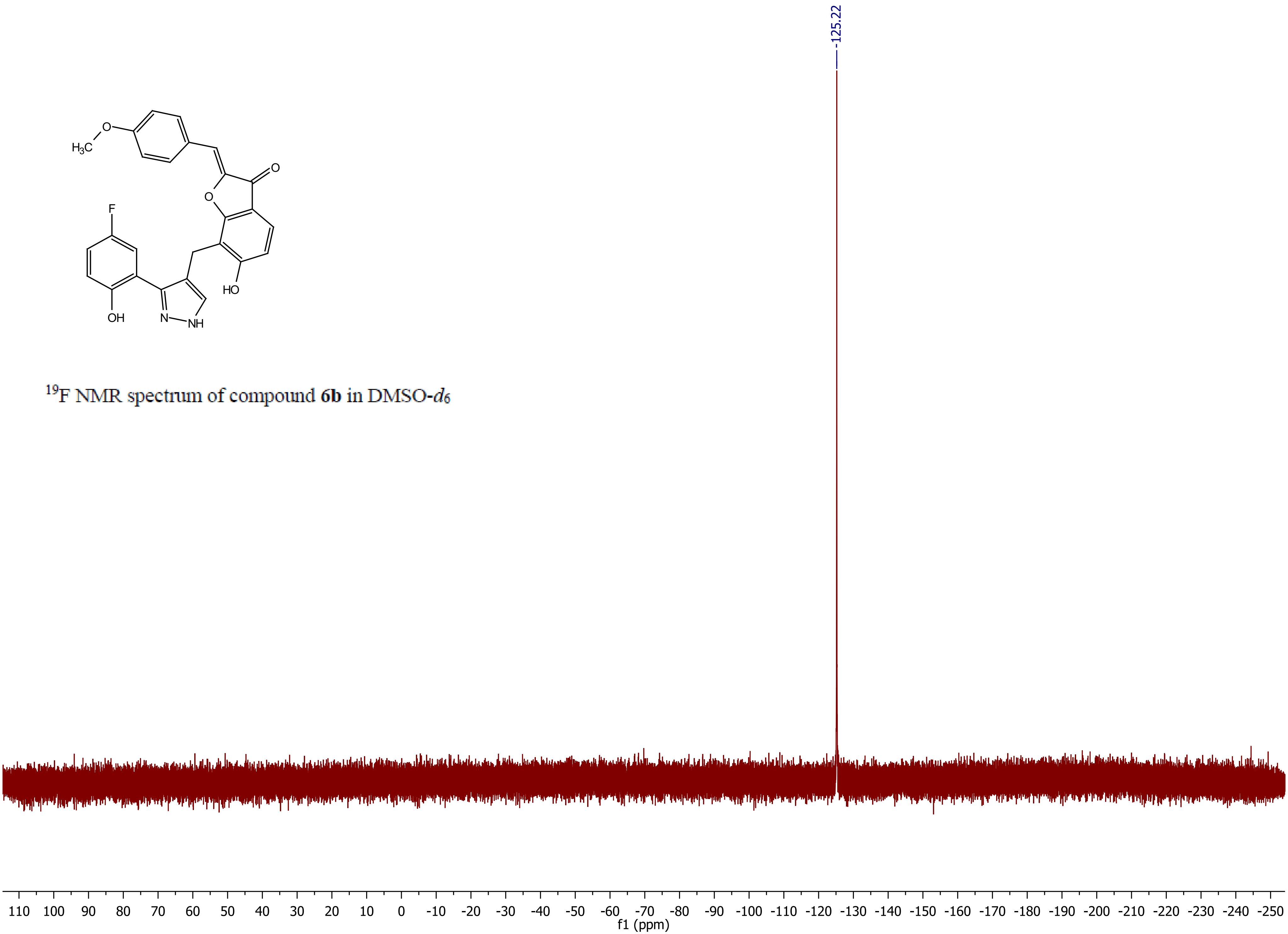

^{19}F NMR spectrum of compound **6b** in DMSO-d_6

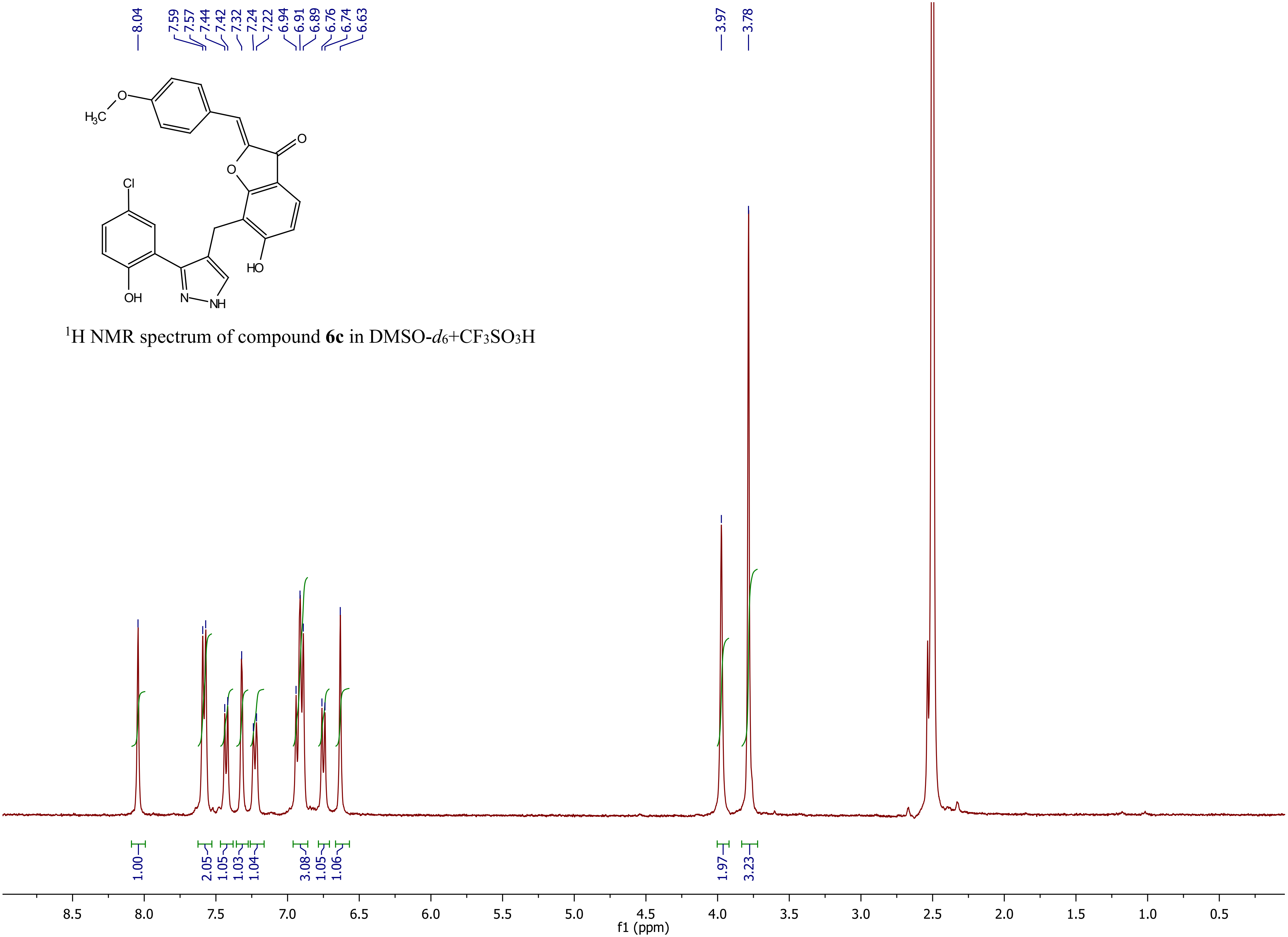

^{1}H NMR spectrum of compound **6c** in DMSO-d_6+CF_3SO_3H

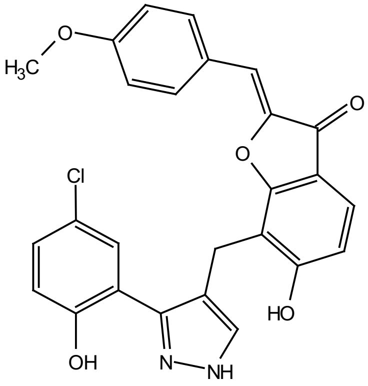

^{13}C NMR spectrum of compound **6c** in DMSO-d_6+CF_3SO_3H

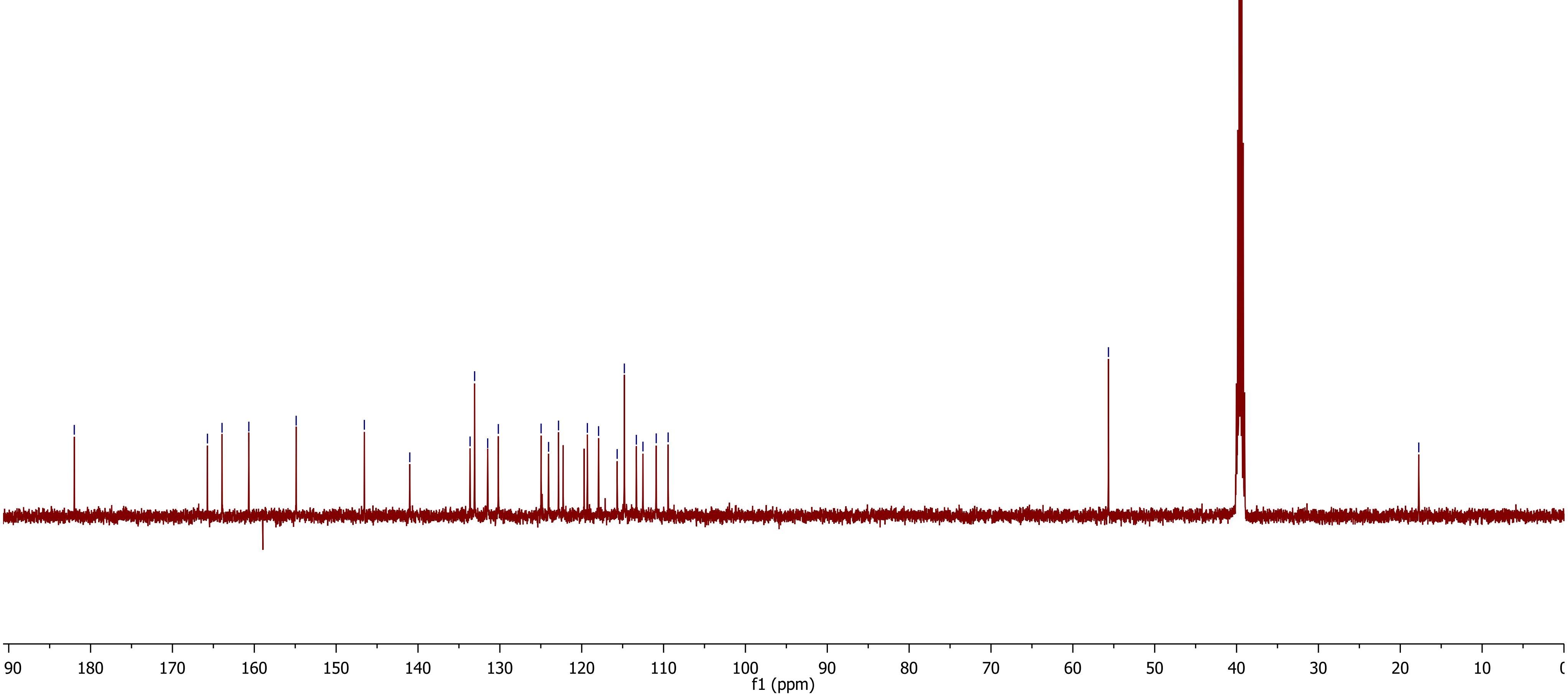

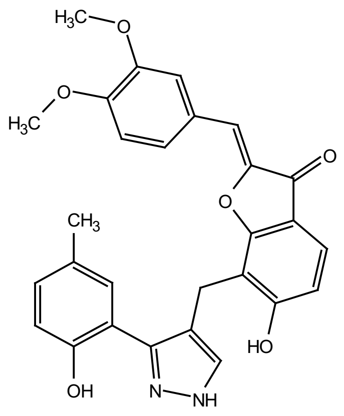

^{1}H NMR spectrum of compound **6d** in DMSO-d_6+CF_3SO_3H

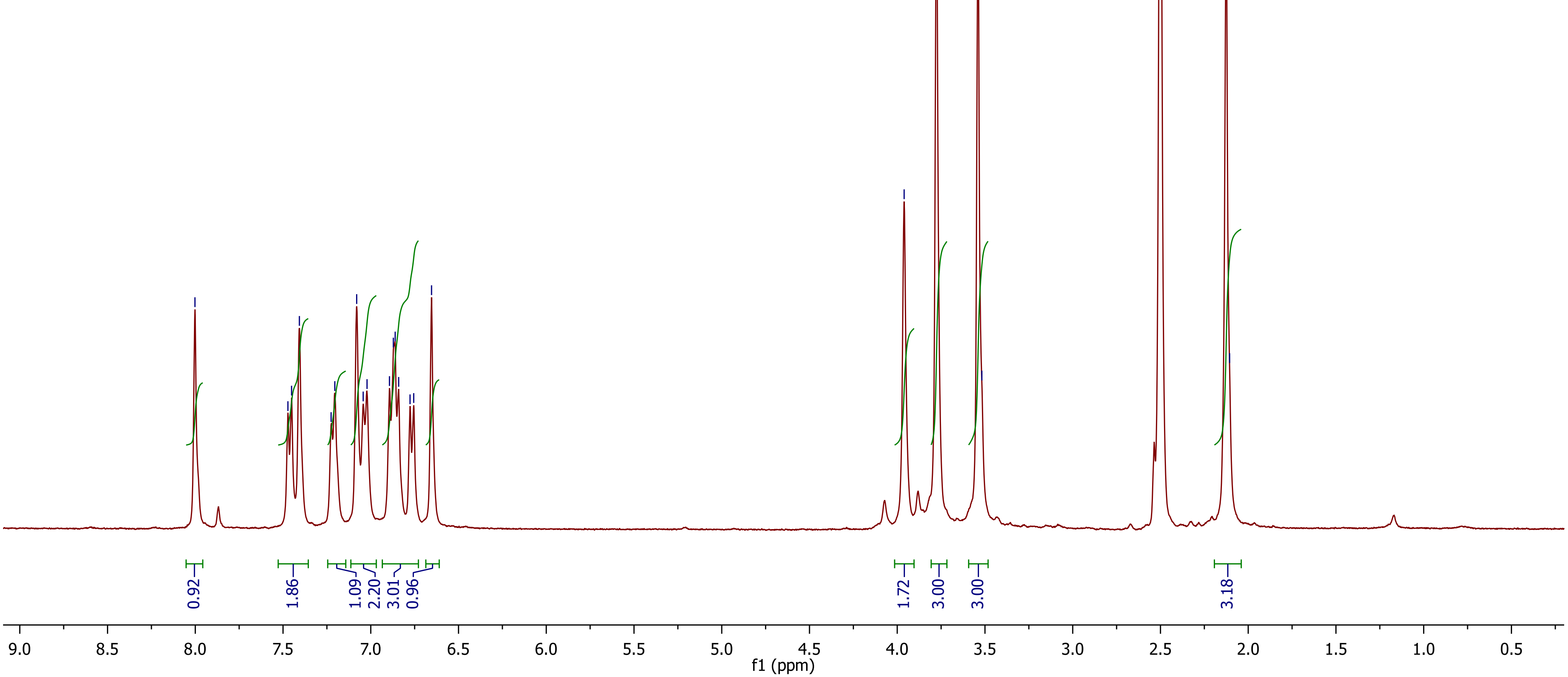

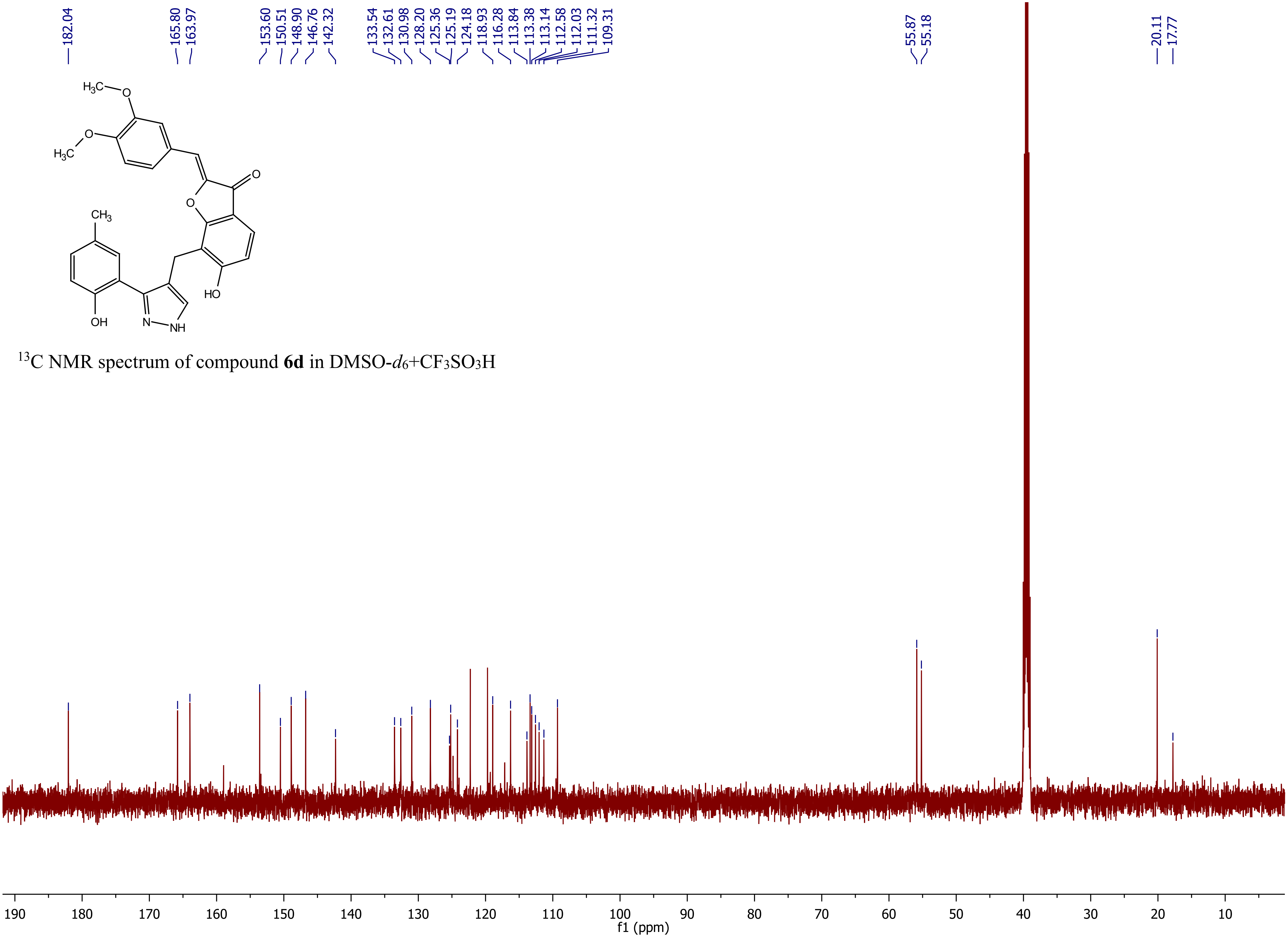

^{13}C NMR spectrum of compound **6d** in DMSO-d_6+CF_3SO_3H

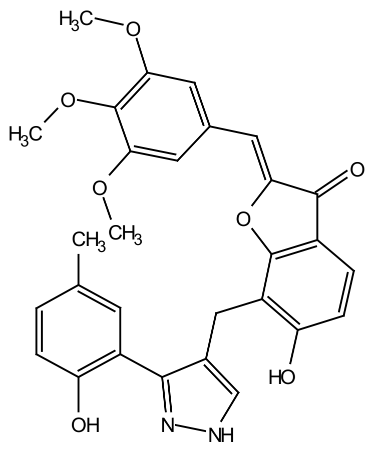

^{1}H NMR spectrum of compound **6e** in DMSO-d_6+CF_3SO_3H

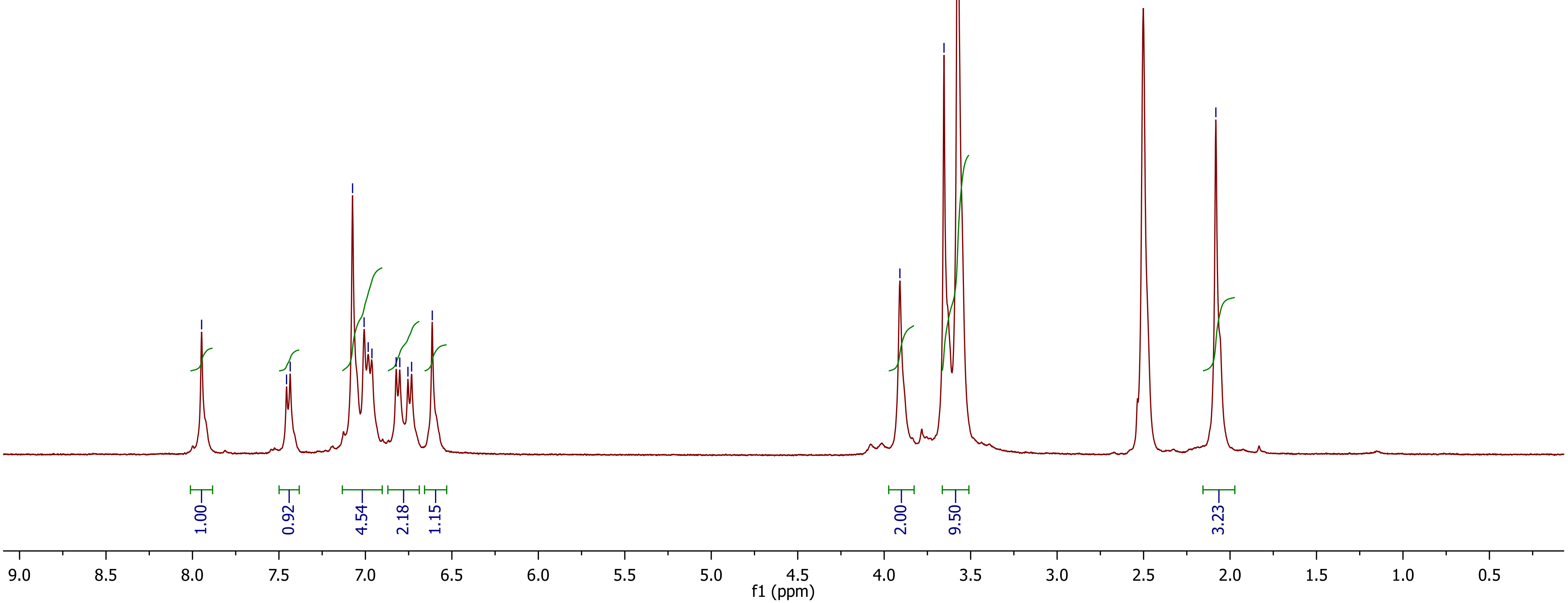

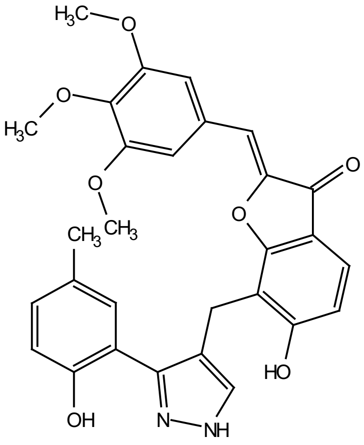

^{13}C NMR spectrum of compound **6e** in DMSO-d_6+CF_3SO_3H

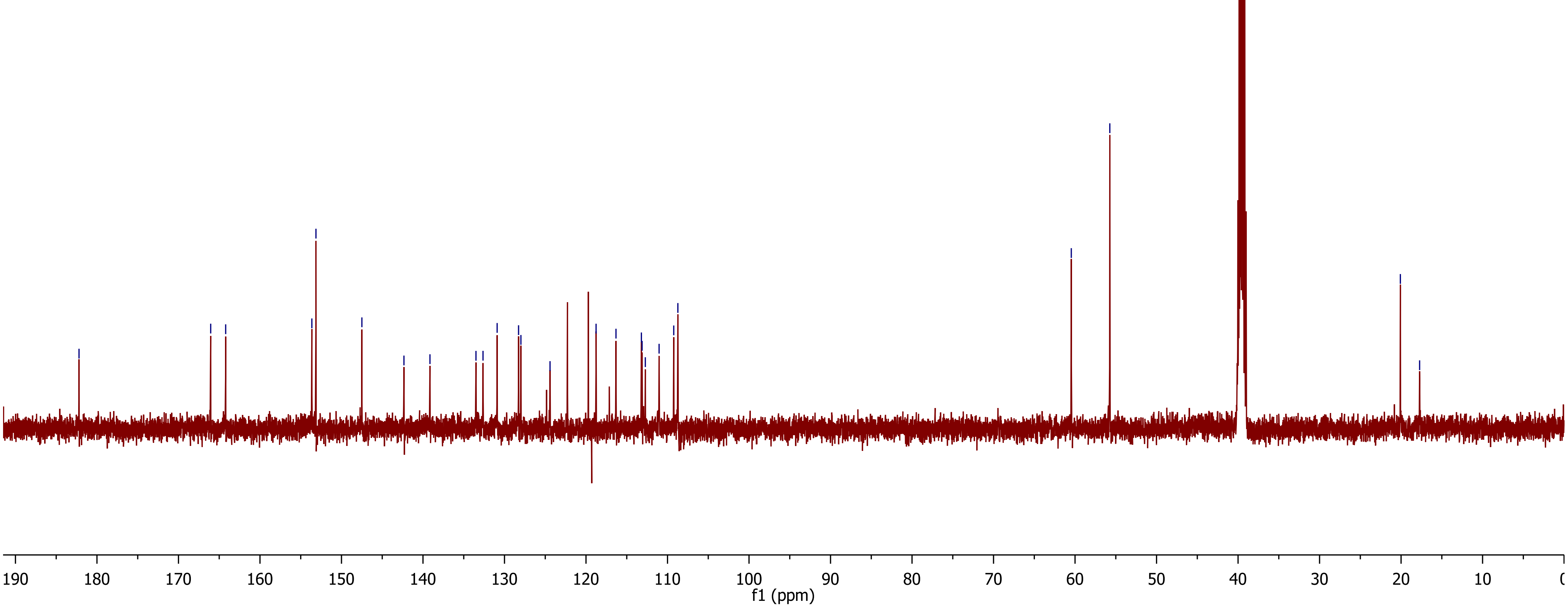

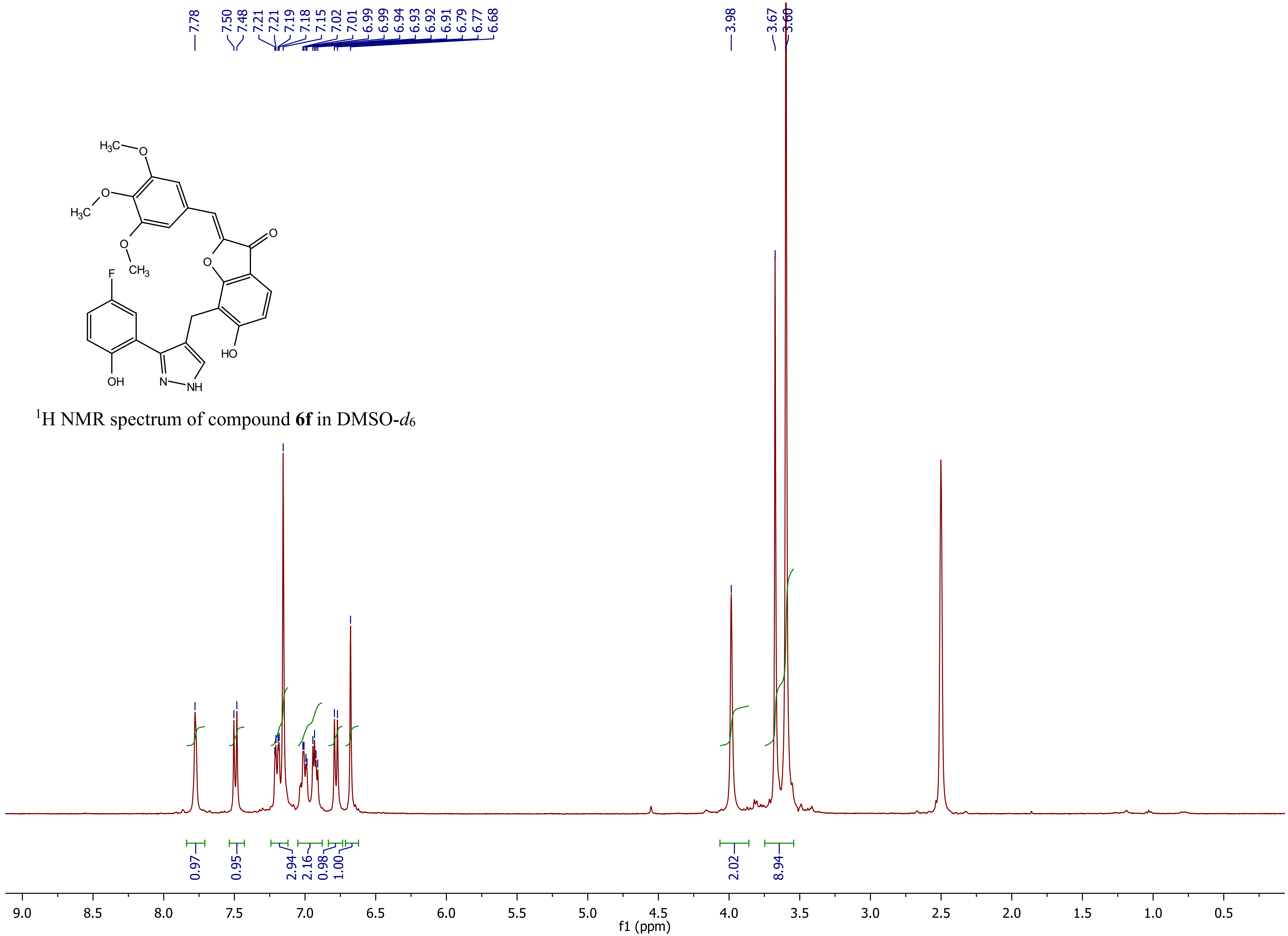

^{1}H NMR spectrum of compound **6f** in DMSO-d_6

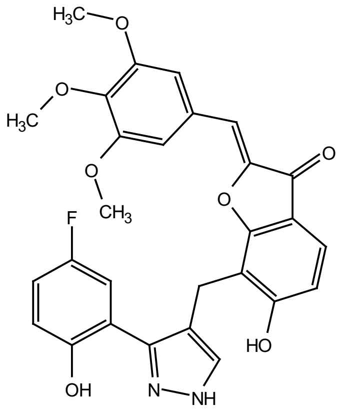

^{13}C NMR spectrum of compound **6f** in DMSO-d_6

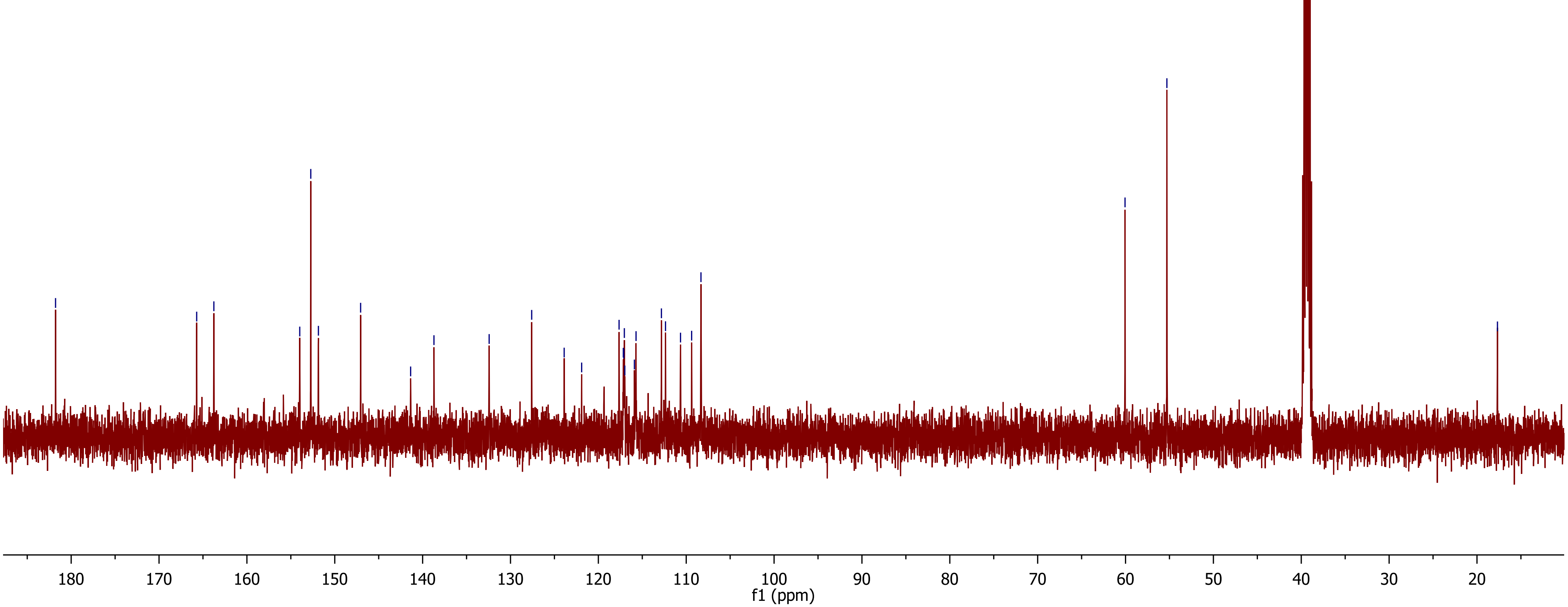

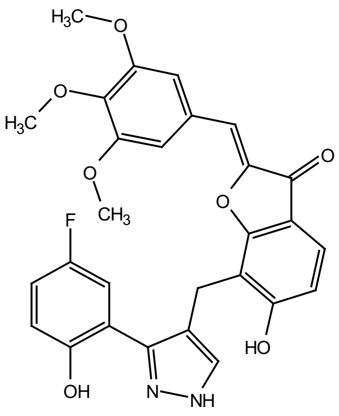

^{19}F NMR spectrum of compound **6f** in DMSO-*d*6

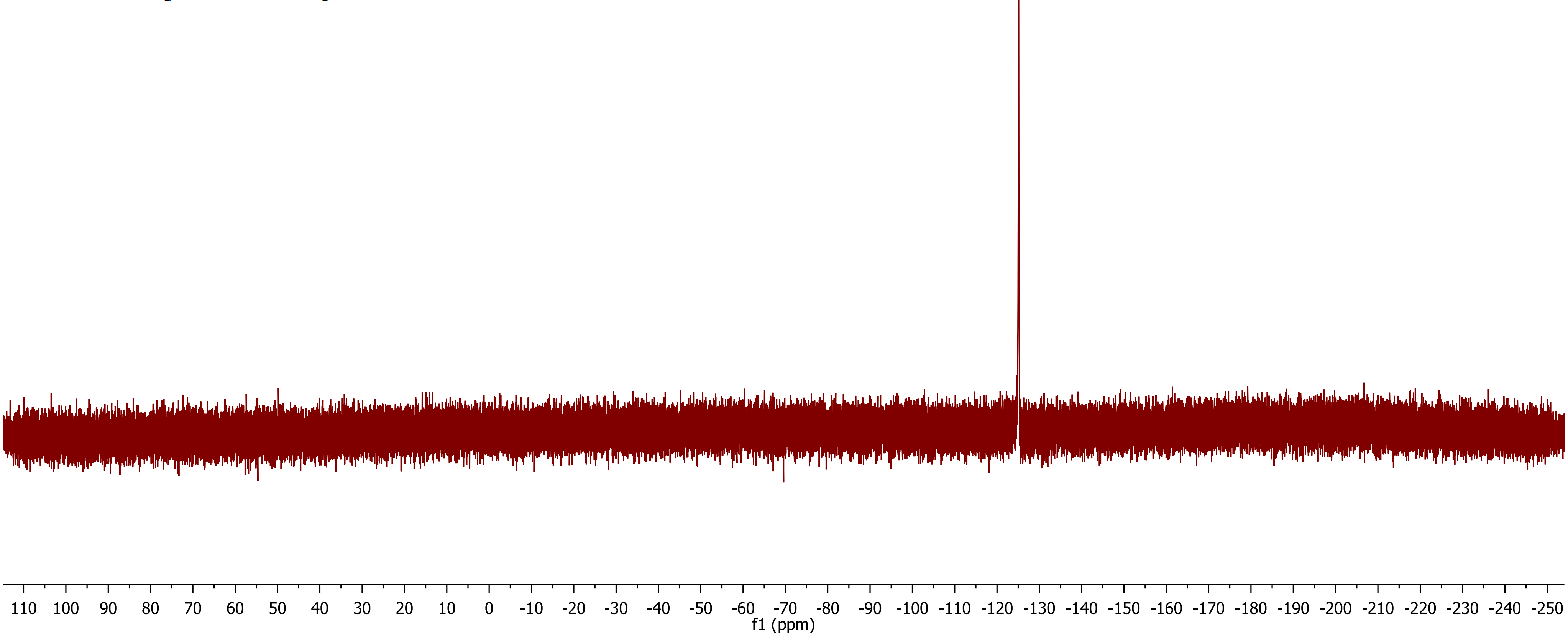

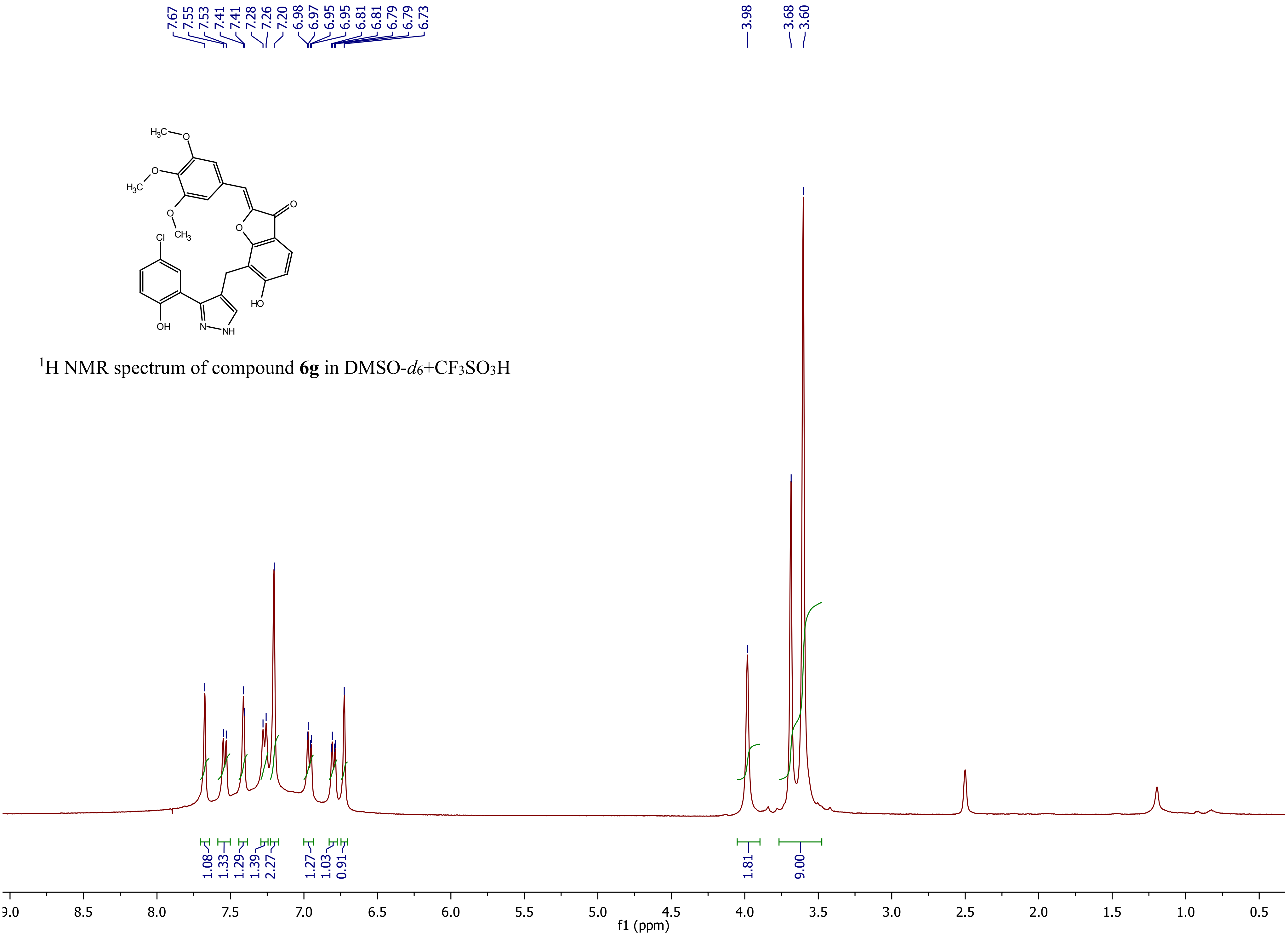

^{1}H NMR spectrum of compound **6g** in DMSO-d_6+CF_3SO_3H

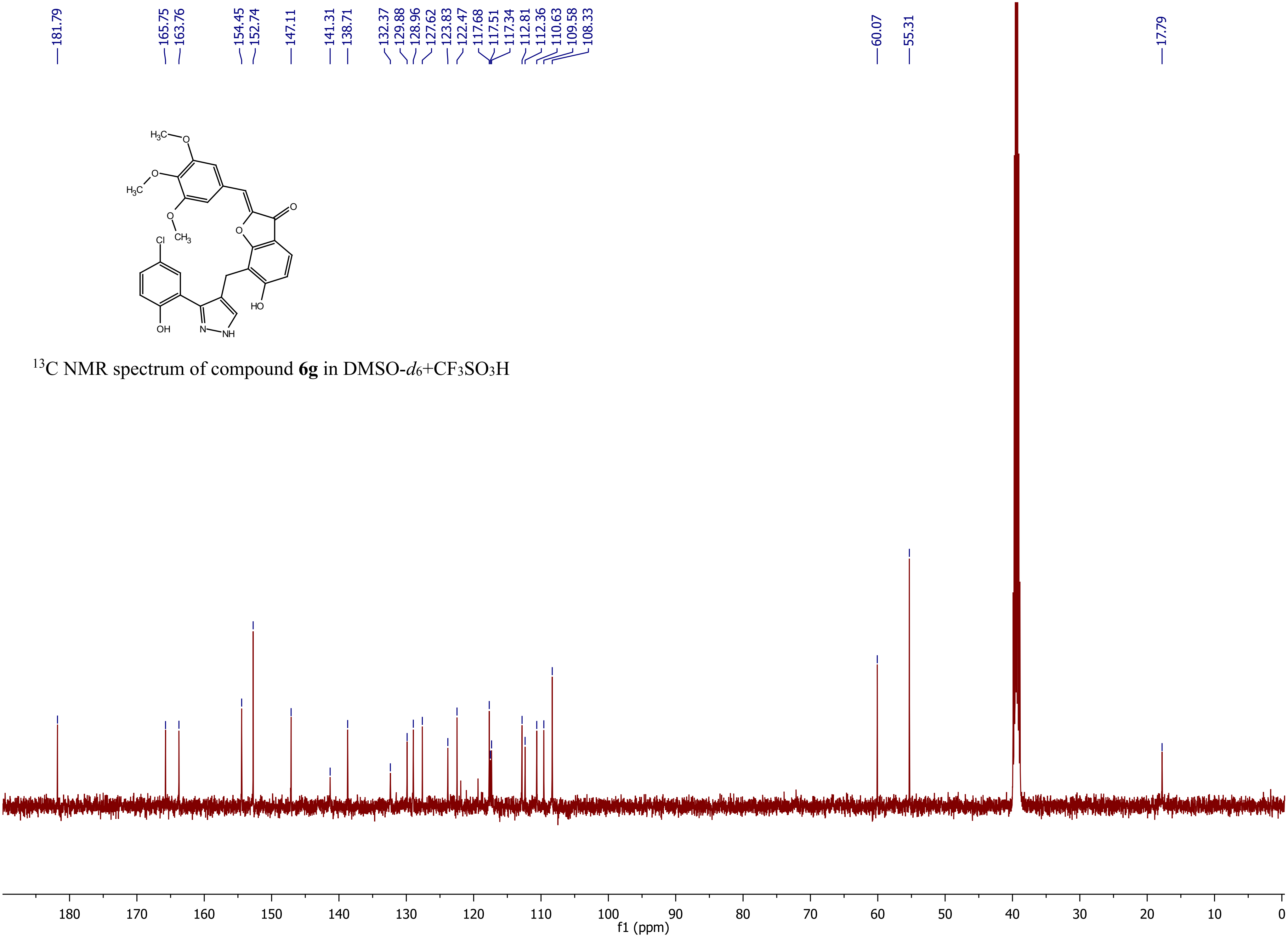

^{13}C NMR spectrum of compound **6g** in DMSO-d_6+CF_3SO_3H

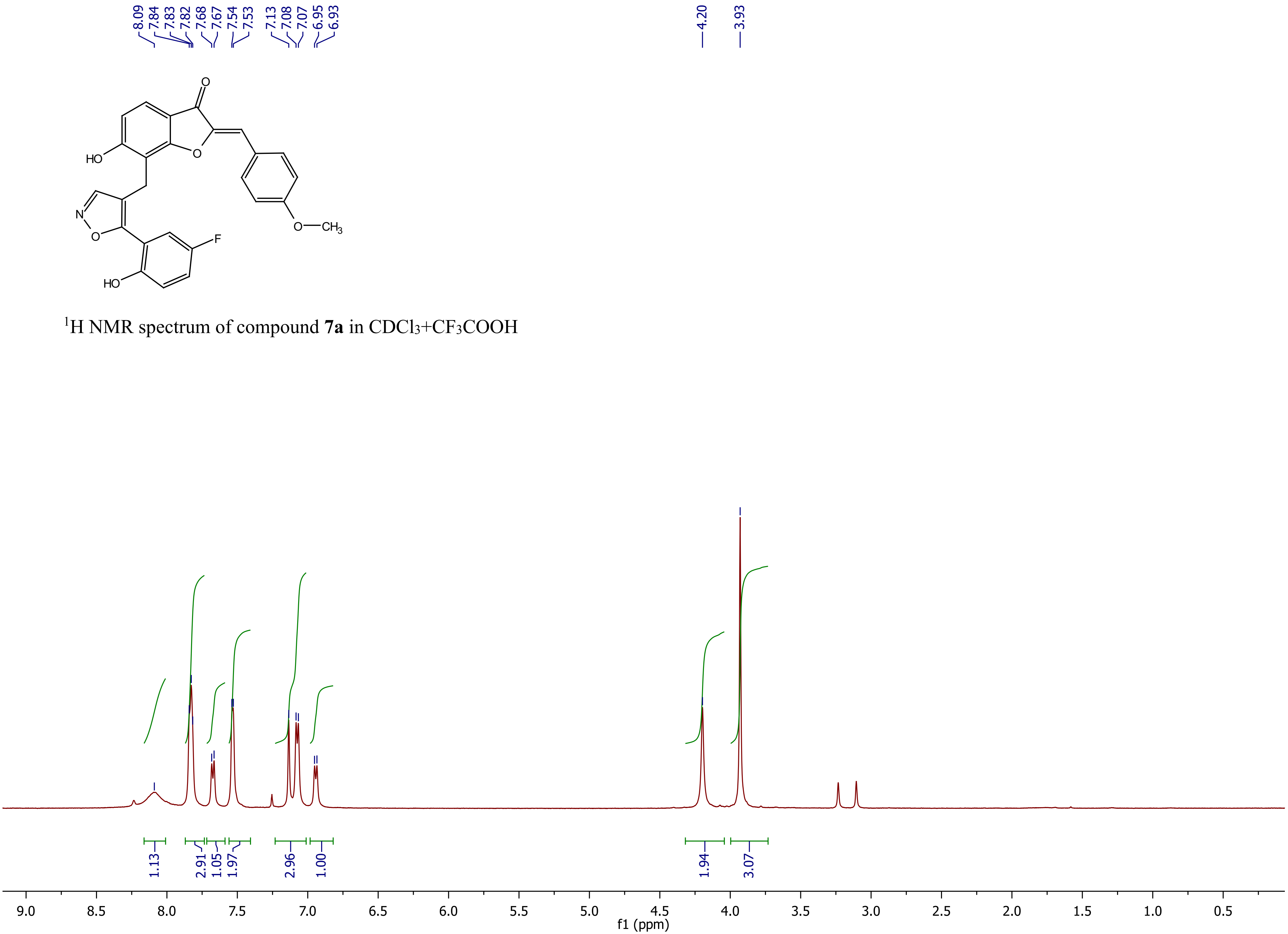

^{1}H NMR spectrum of compound **7a** in $CDCl_3$+CF_3COOH

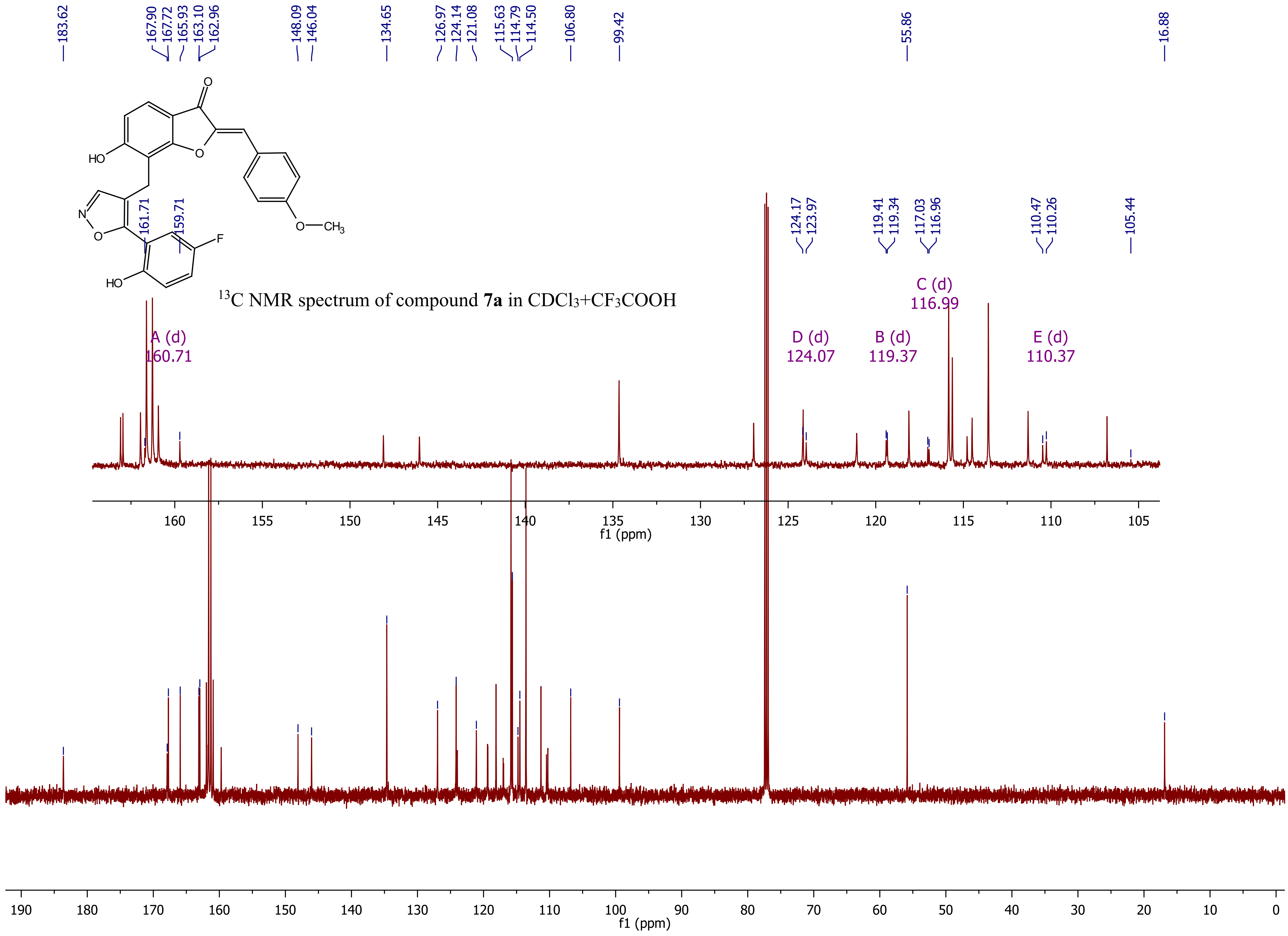

183.62
167.90
167.72
165.93
163.10
162.96
148.09
146.04
134.65
126.97
124.14
121.08
115.63
114.79
114.50
106.80
99.42
55.86
16.88
HO
O
O
N
O
HO
F
O—CH3
161.71
159.71
124.17
123.97
119.41
119.34
117.03
116.96
110.47
110.26
105.44
^{13}C NMR spectrum of compound **7a** in CDCl3+CF3COOH
A (d)
160.71
D (d)
124.07
B (d)
119.37
C (d)
116.99
E (d)
110.37
f1 (ppm)
f1 (ppm)

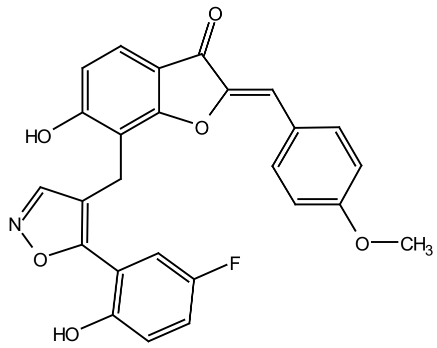

^{19}F NMR spectrum of compound **7a** in DMSO-d_6

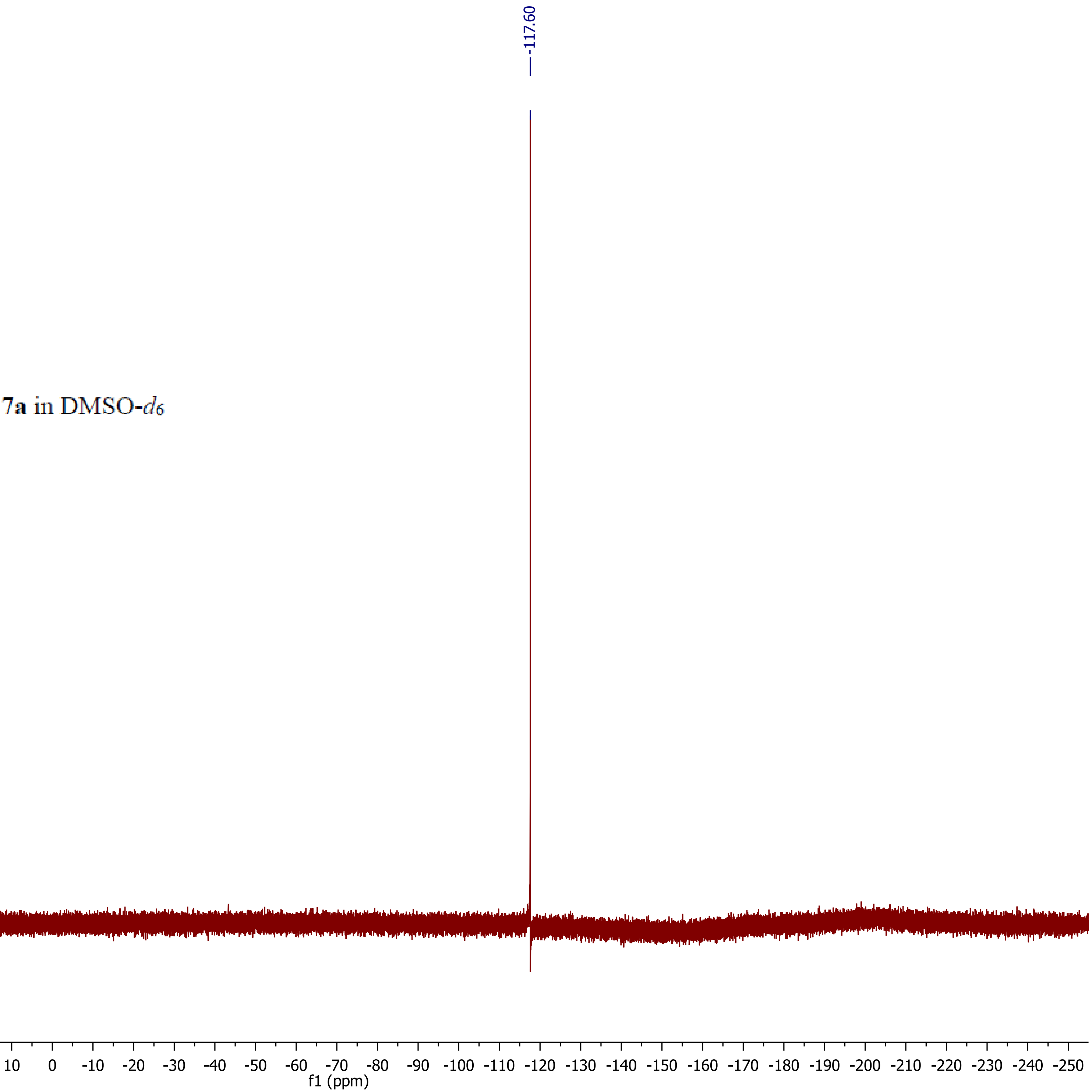

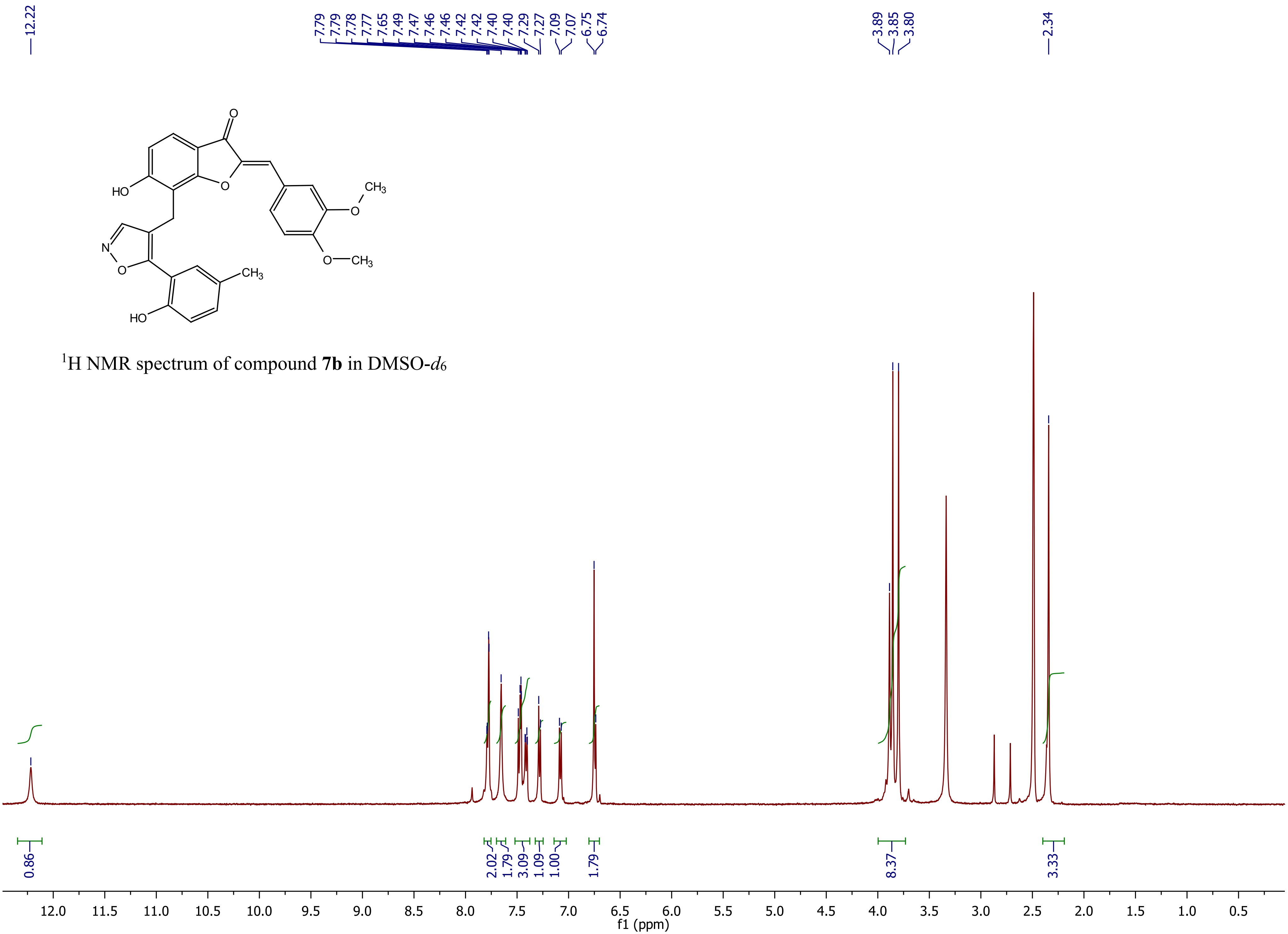

^{1}H NMR spectrum of compound **7b** in DMSO-d_6

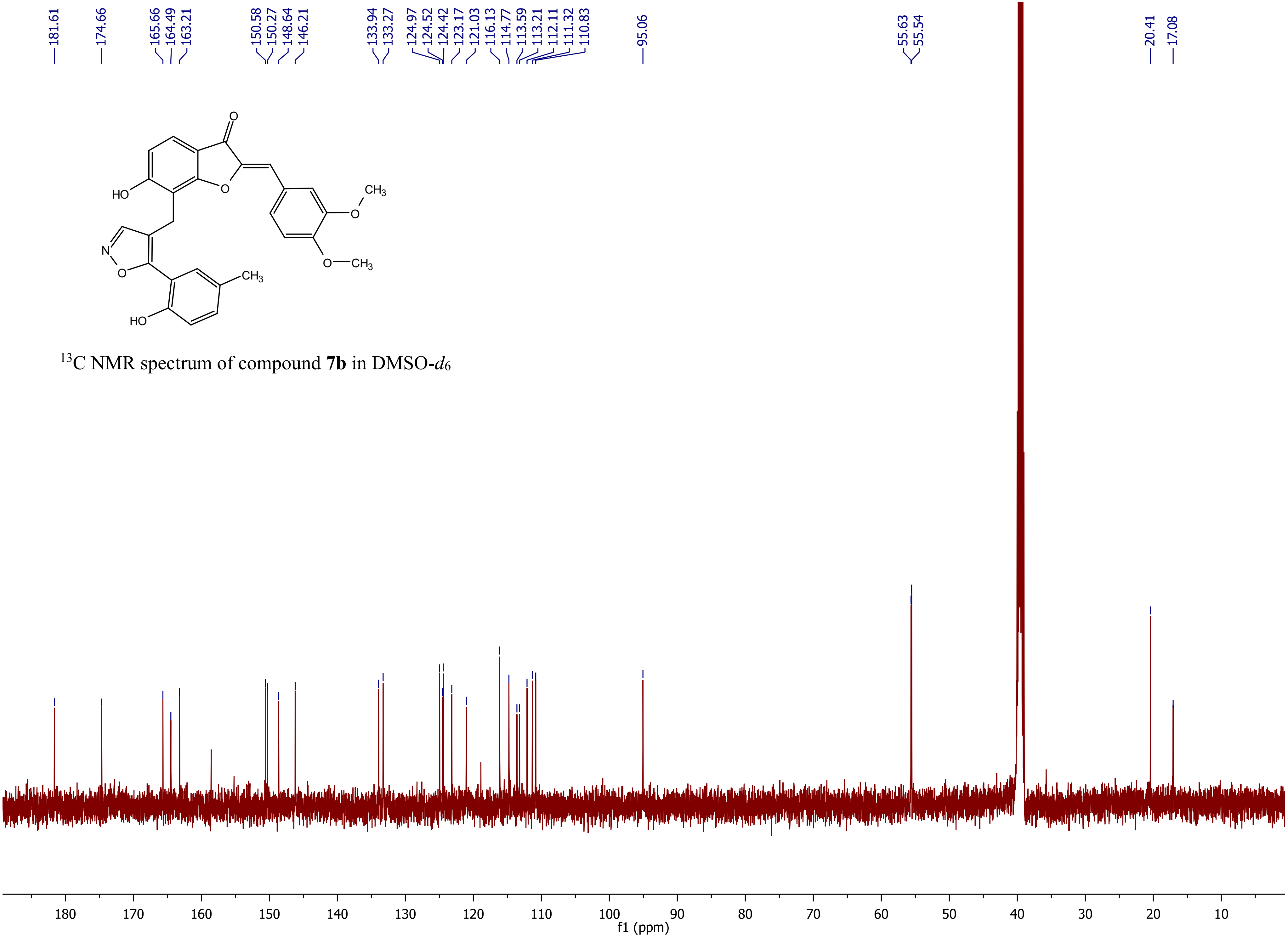

^{13}C NMR spectrum of compound **7b** in DMSO-d_6

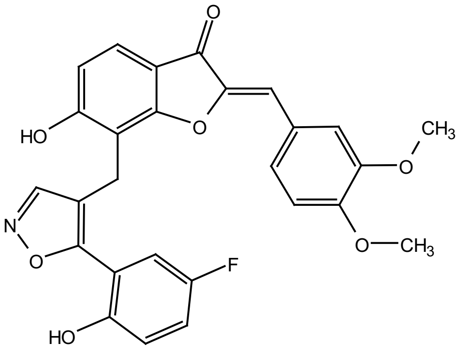

^{1}H NMR spectrum of compound **7c** in $CDCl_3$+CF_3COOH

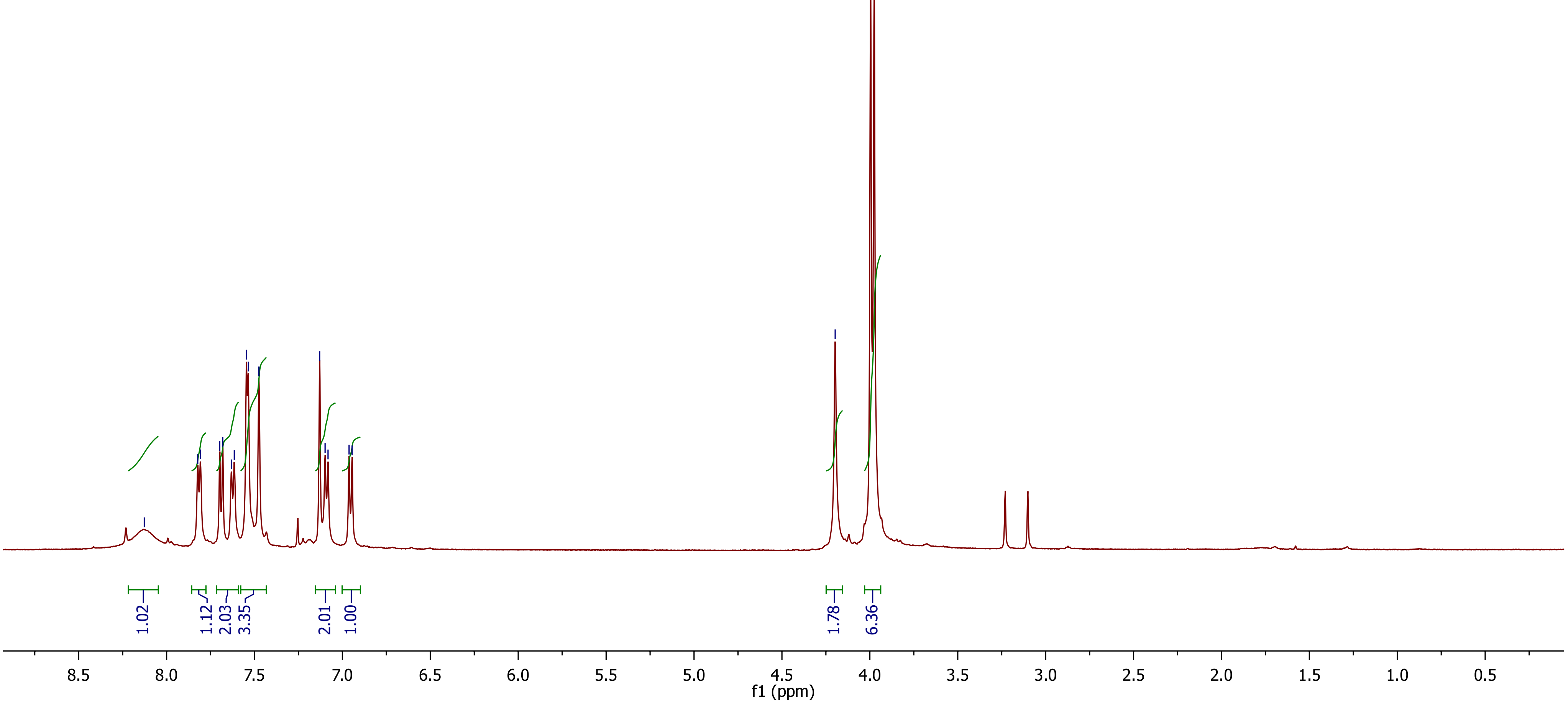

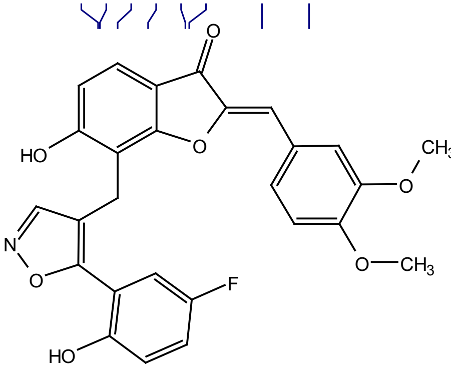

^{13}C NMR spectrum of compound **7c** in $CDCl_3$+CF_3COOH

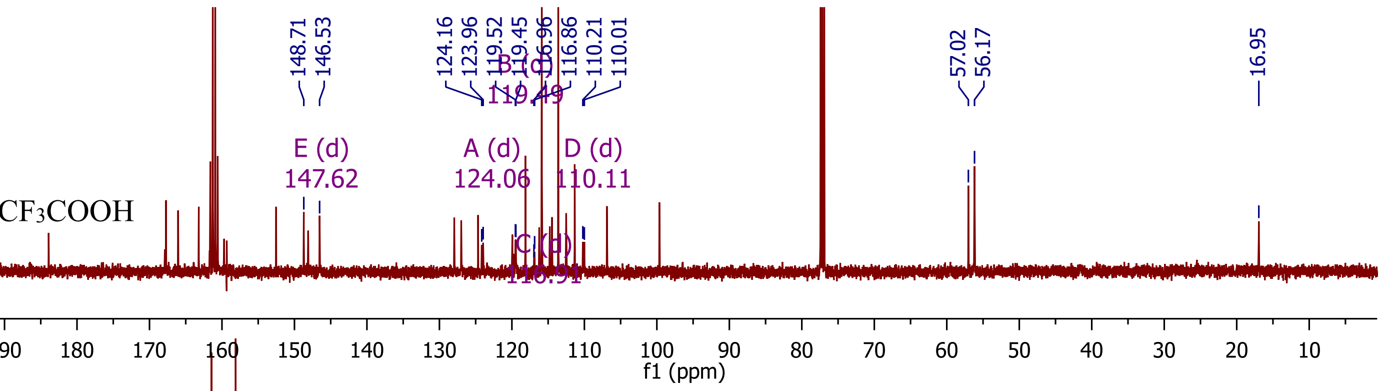

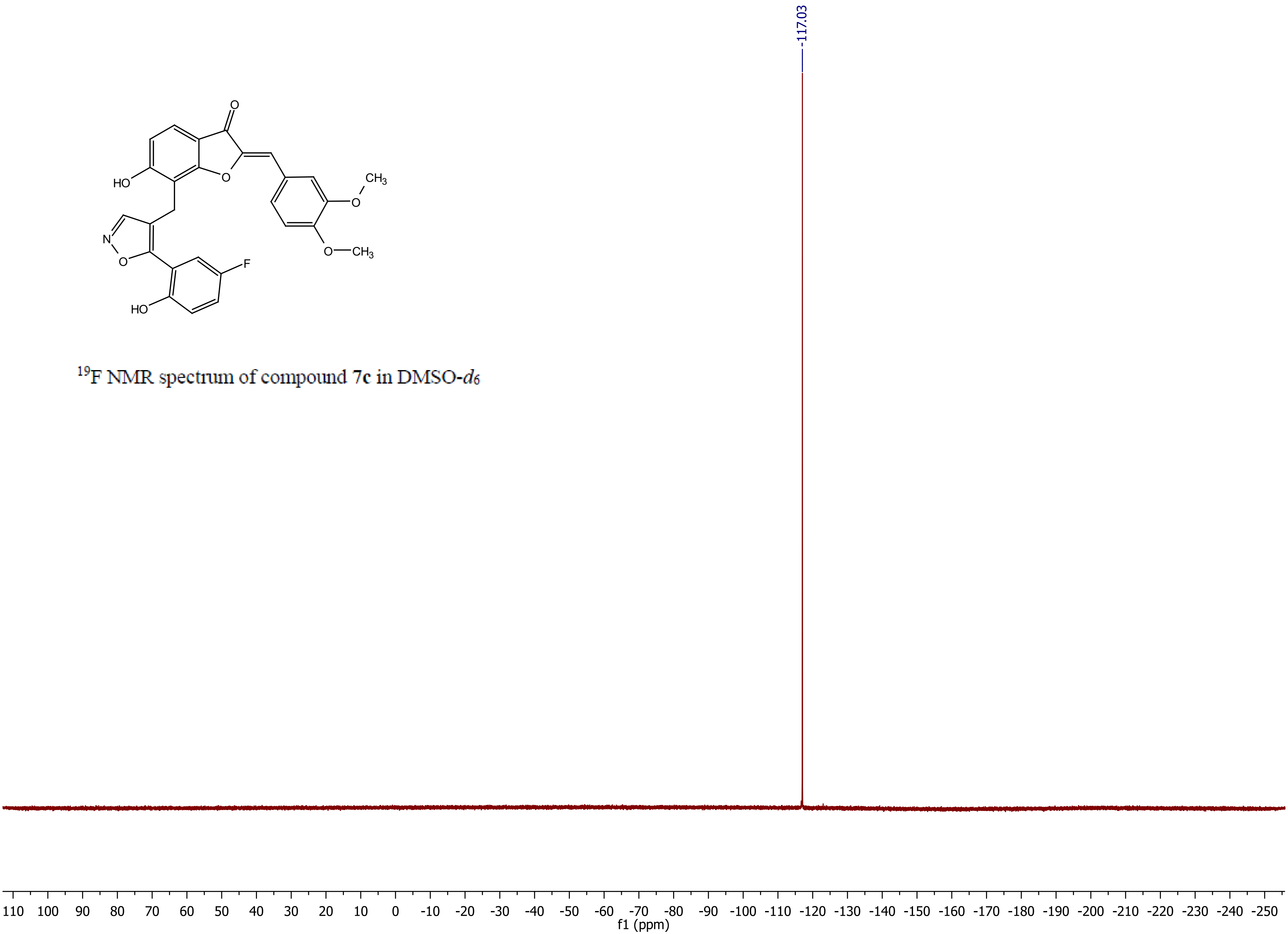

^{19}F NMR spectrum of compound **7c** in DMSO-d_6

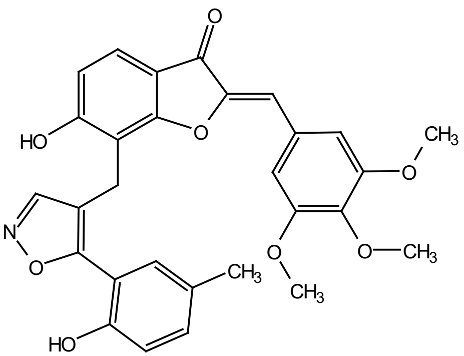

^{1}H NMR spectrum of compound **7d** in $CDCl_3$+CF_3COOH

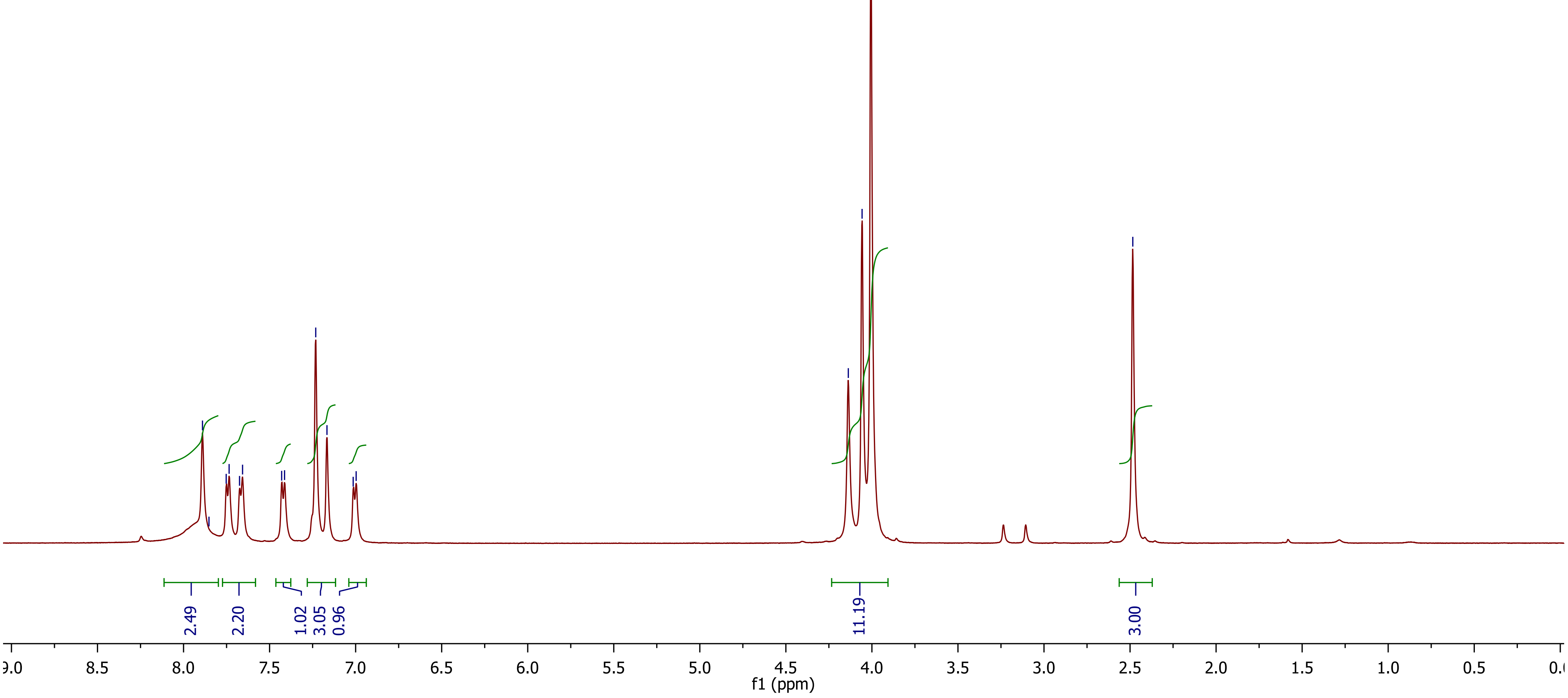

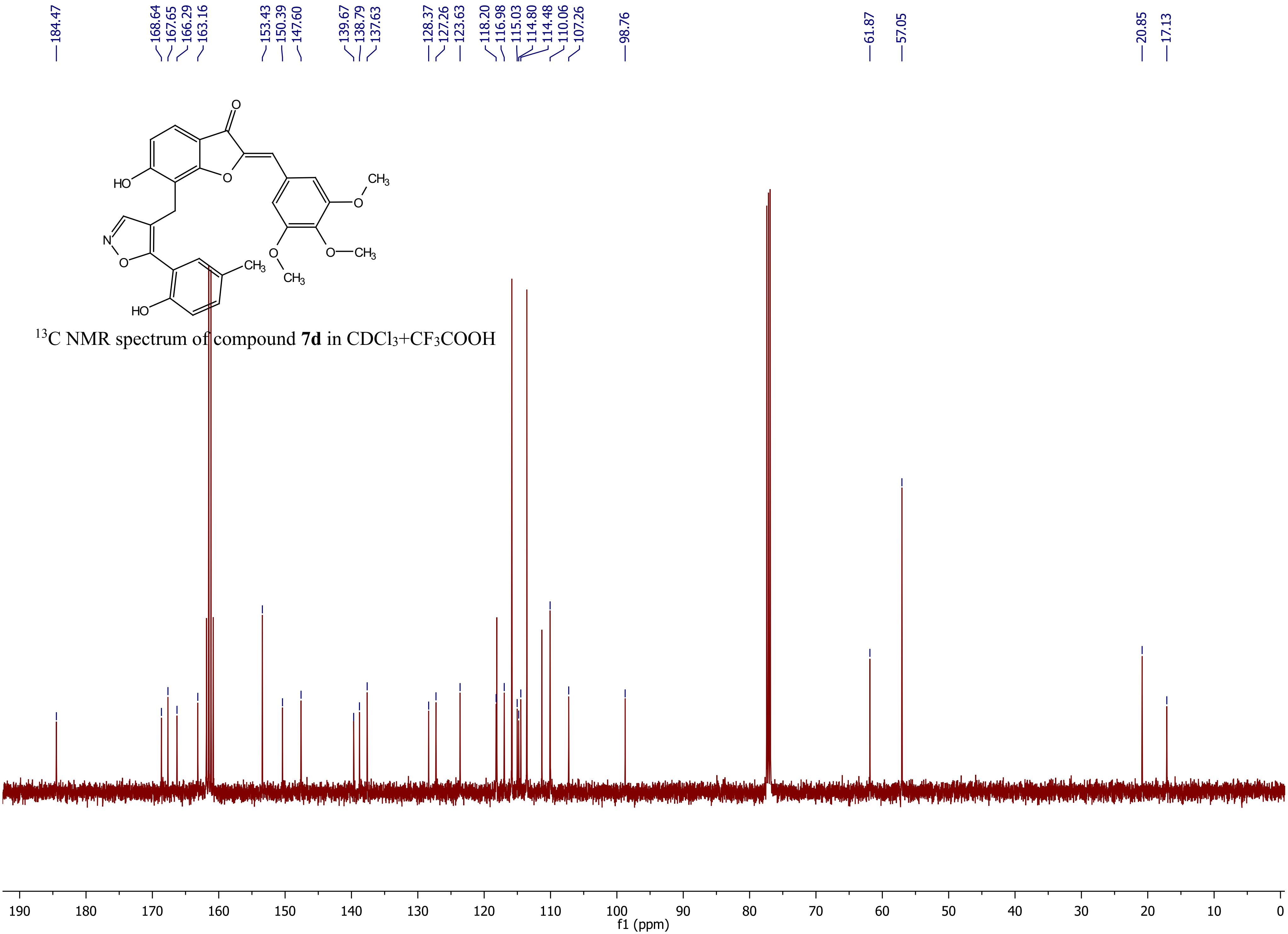

^{13}C NMR spectrum of compound **7d** in $CDCl_3$+CF_3COOH

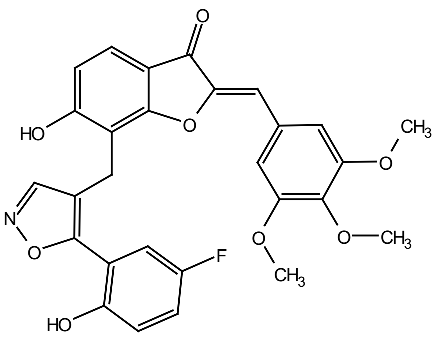

^{1}H NMR spectrum of compound **7e** in $CDCl_3$+CF_3COOH

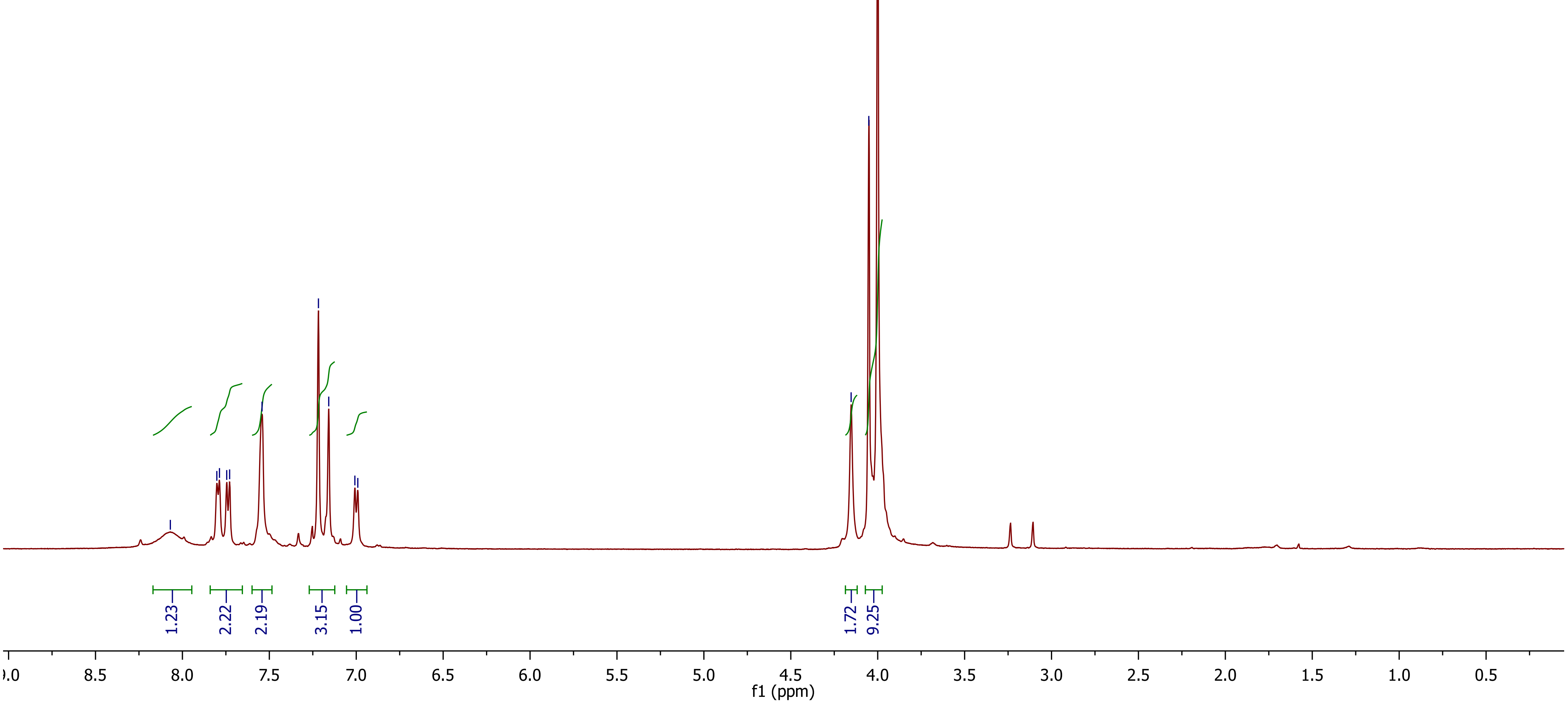

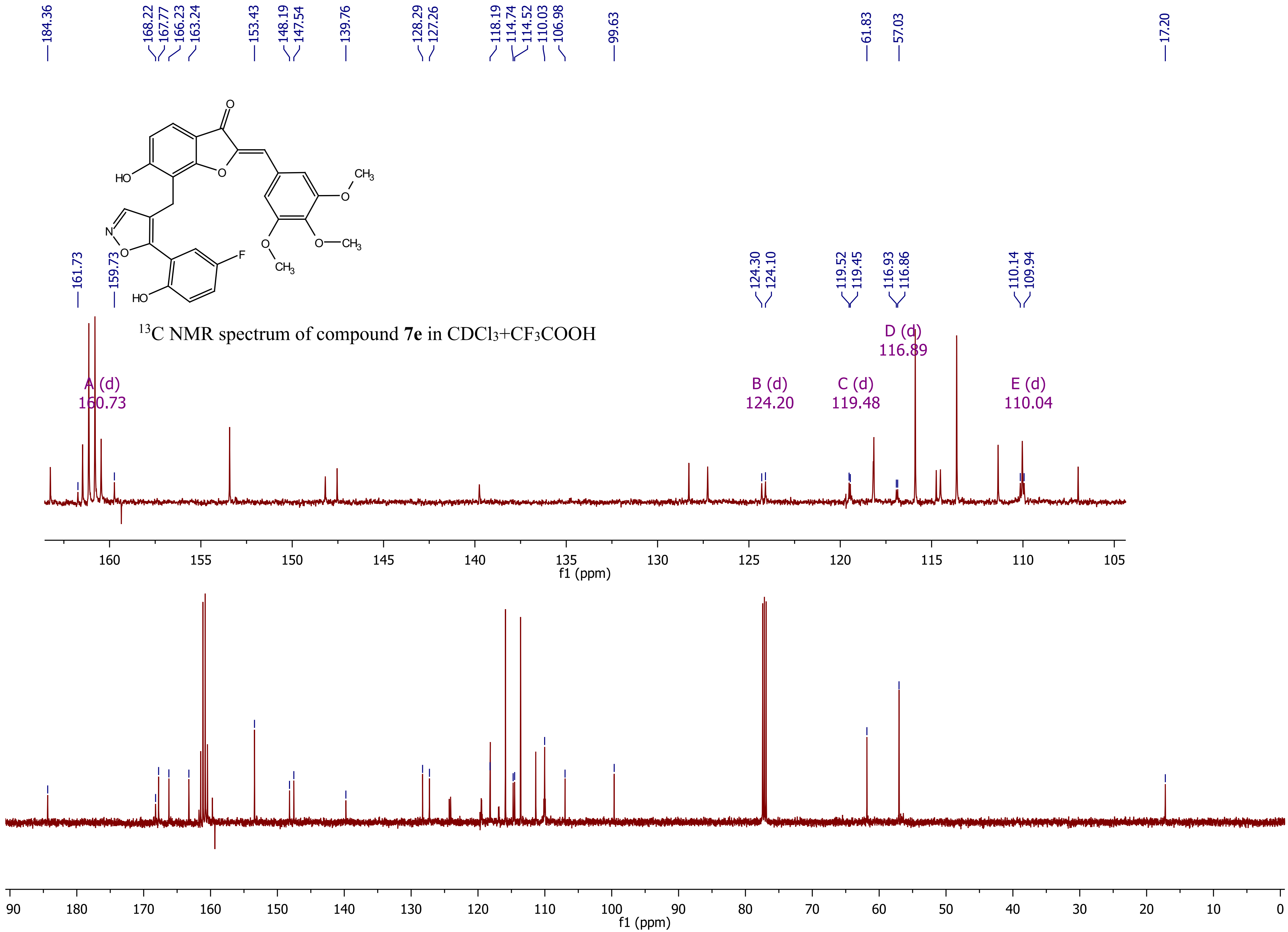

^{13}C NMR spectrum of compound **7e** in $CDCl_3$+CF_3COOH

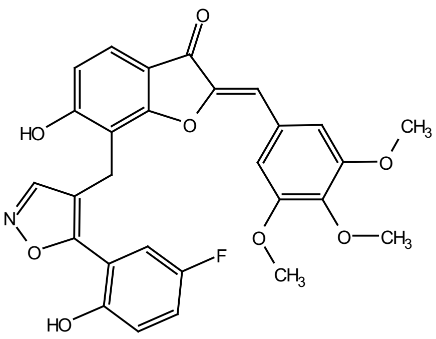

^{19}F NMR spectrum of compound **7e** in DMSO-d_6

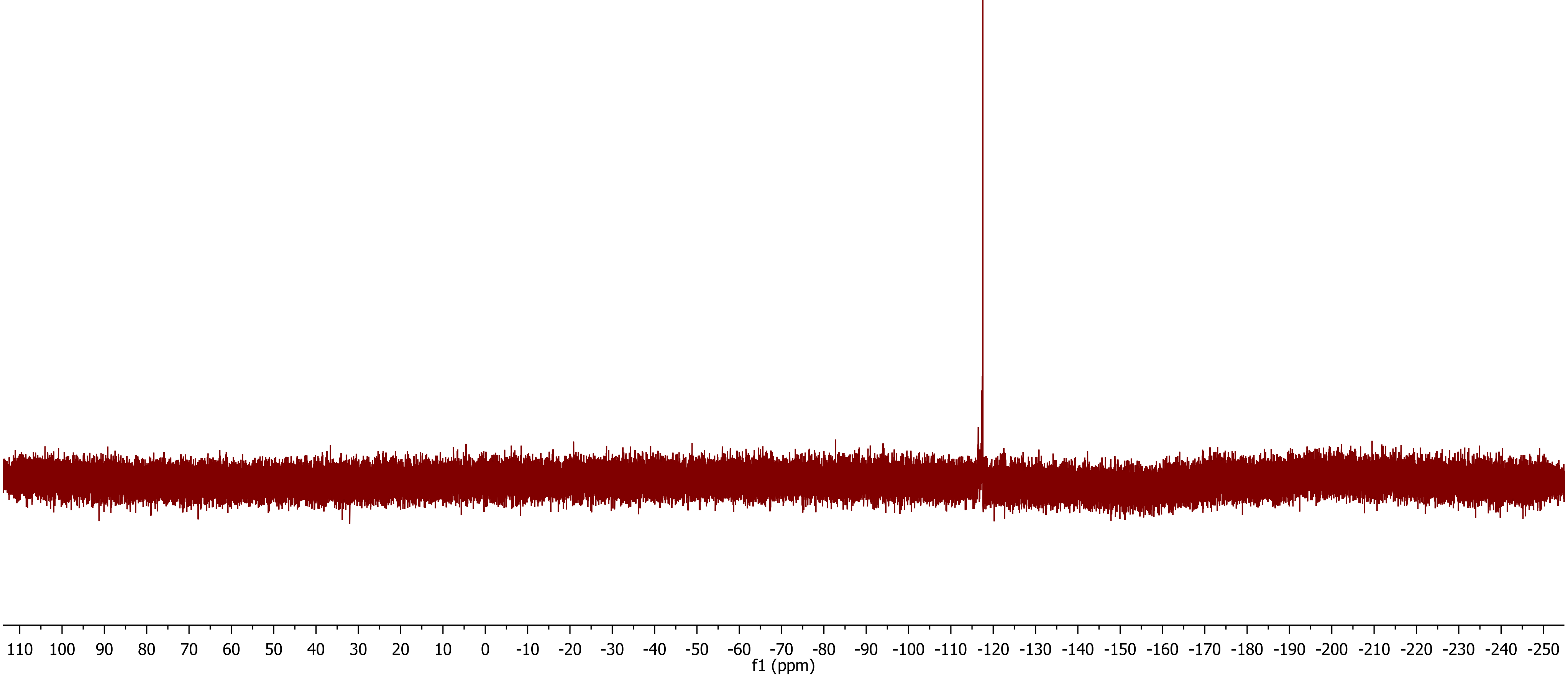

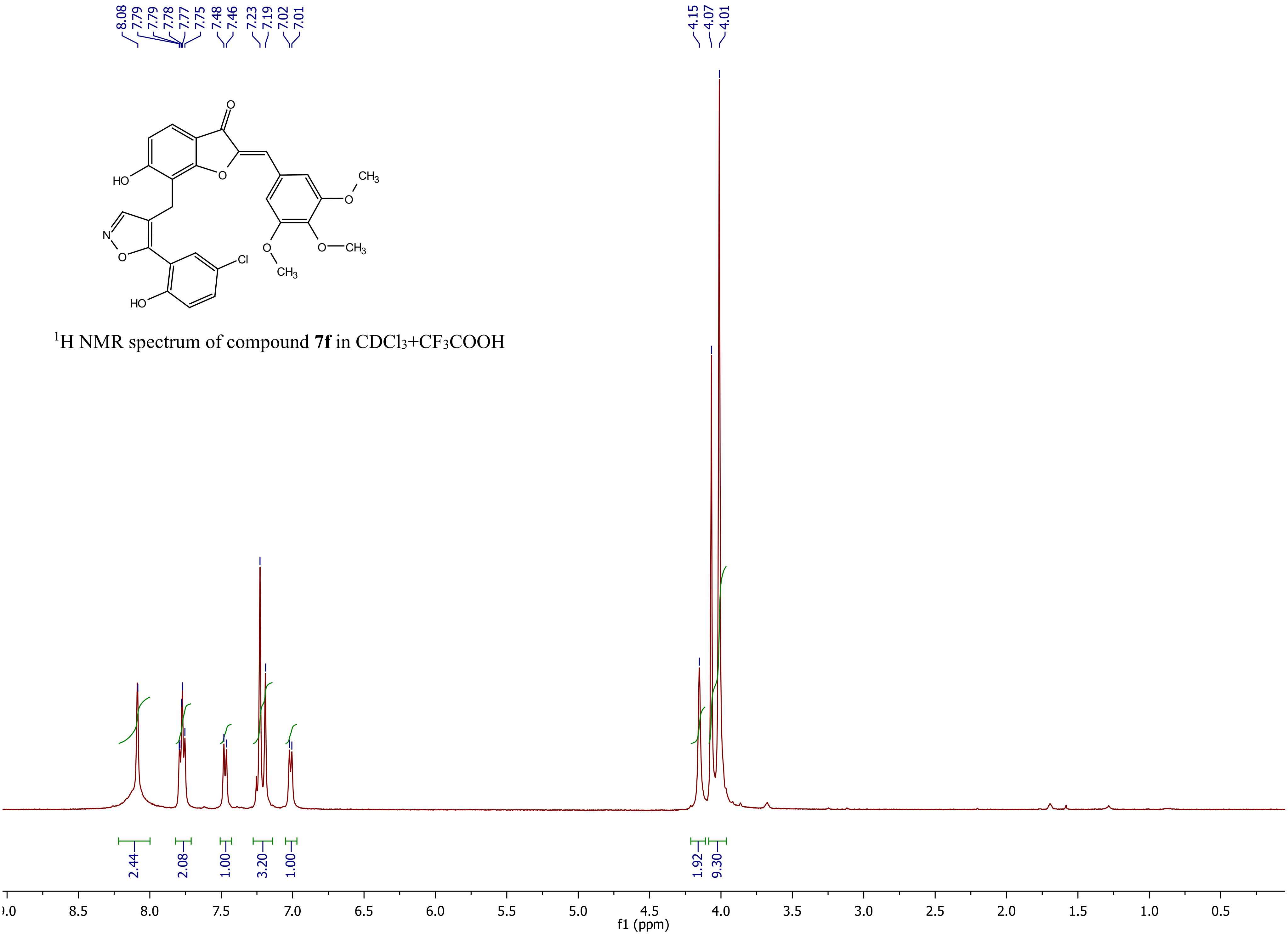

^{1}H NMR spectrum of compound **7f** in $CDCl_3$+CF_3COOH

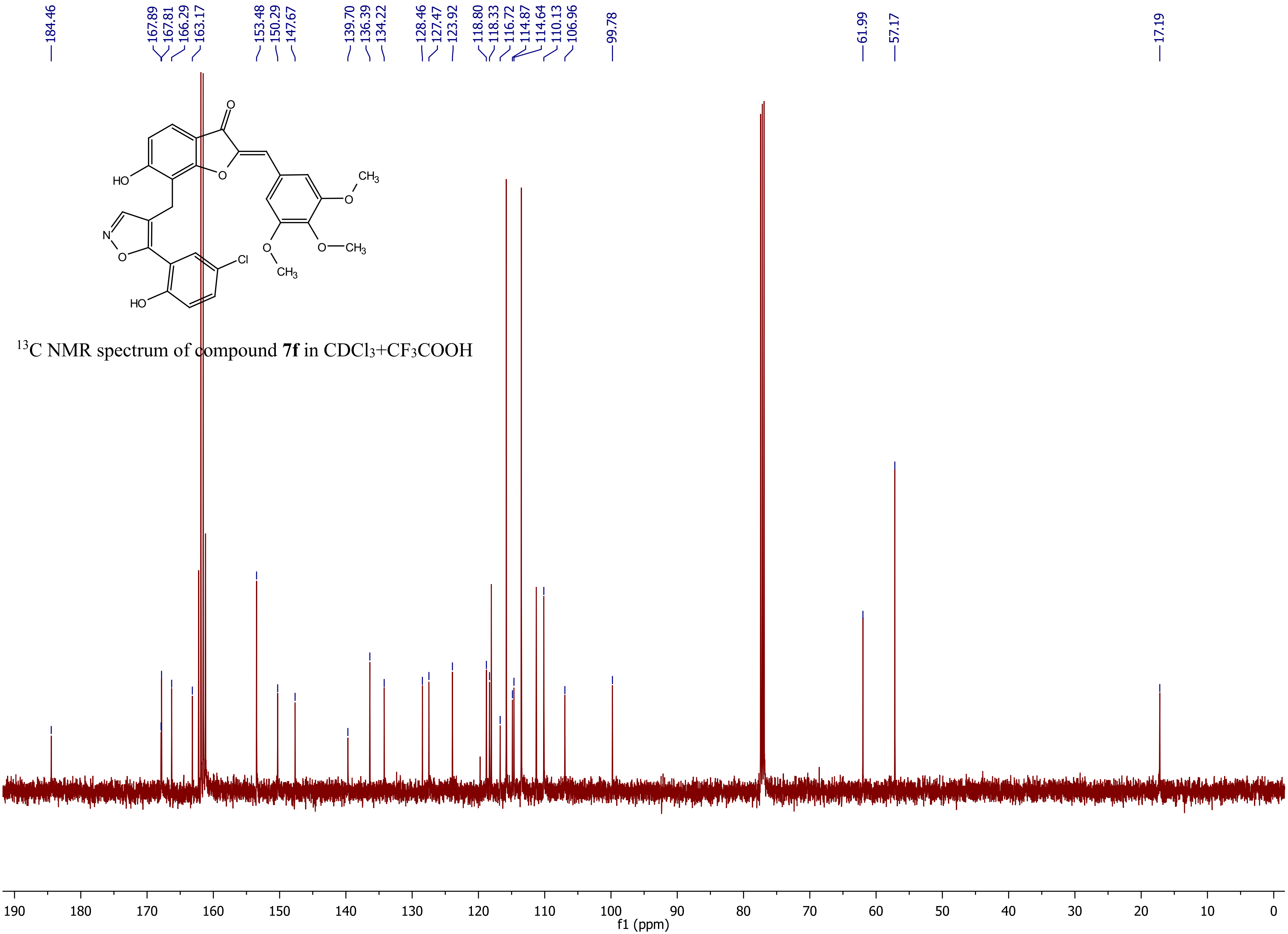

^{13}C NMR spectrum of compound **7f** in $CDCl_3$+CF_3COOH

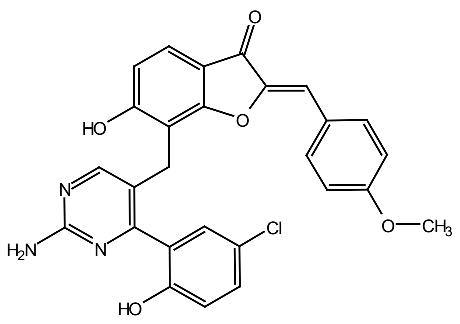

^{1}H NMR spectrum of compound **8a** in DMSO-d_6

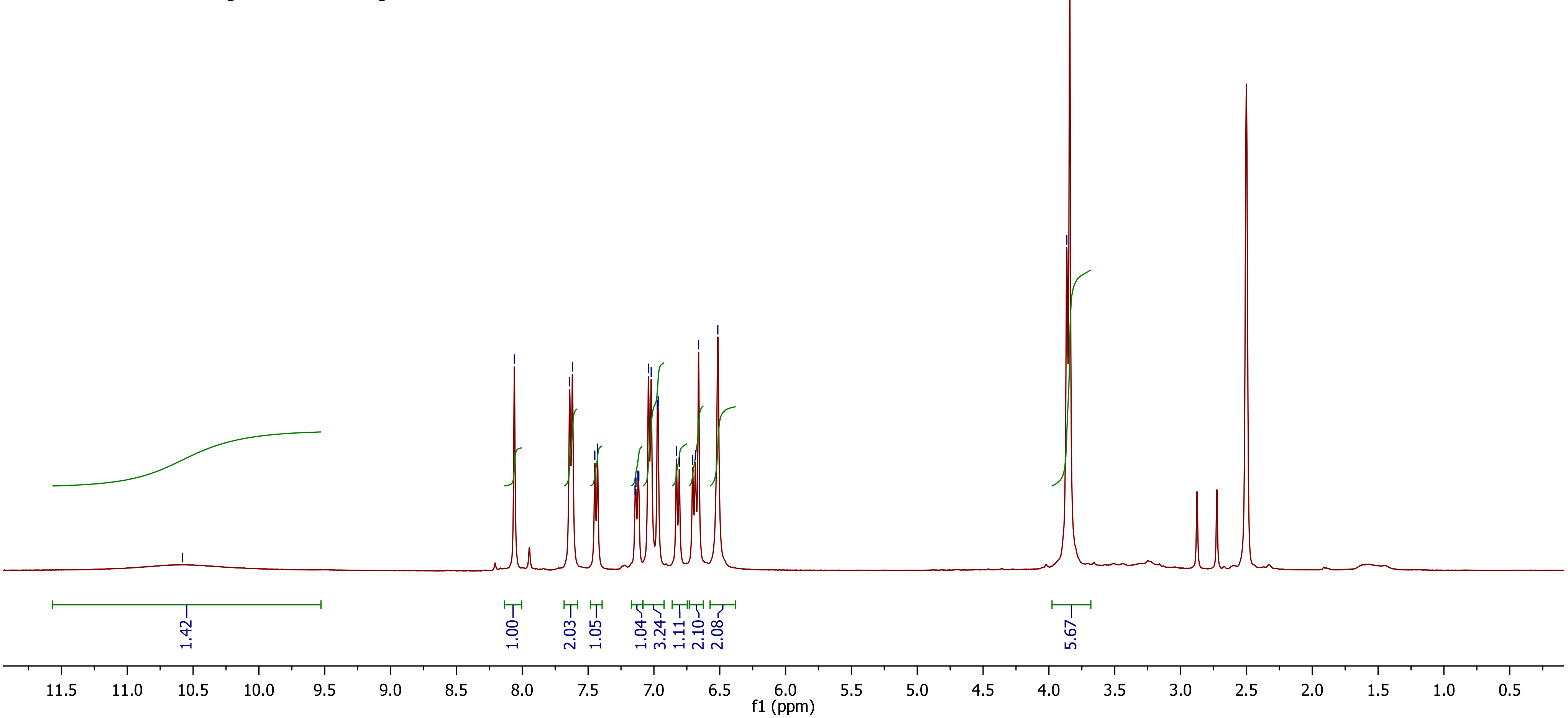

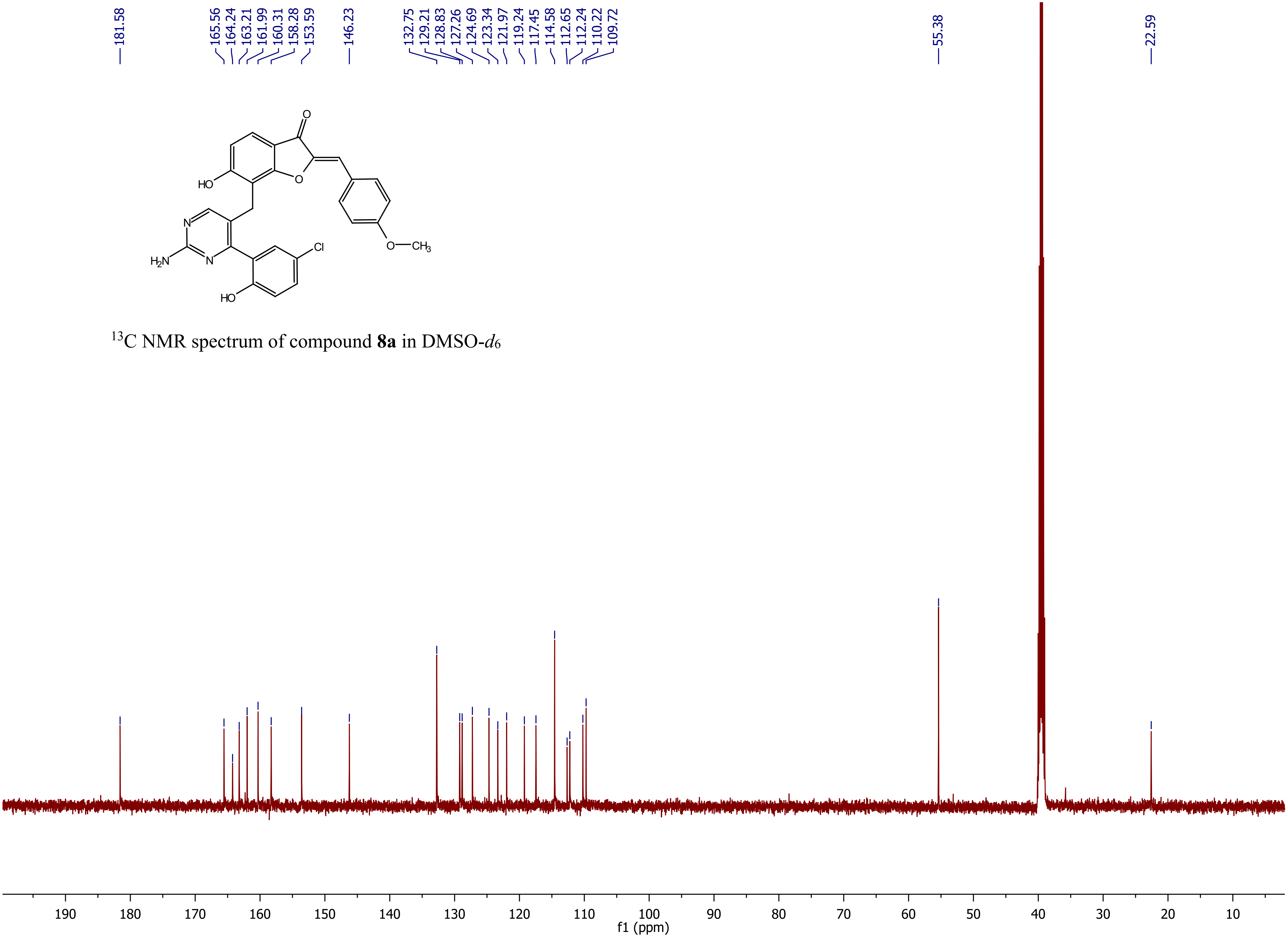

^{13}C NMR spectrum of compound **8a** in DMSO-d_6

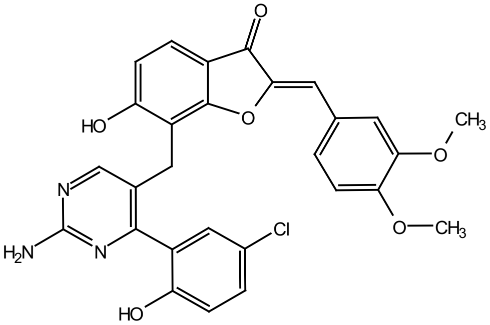

^{1}H NMR spectrum of compound **8b** in DMSO-d_6

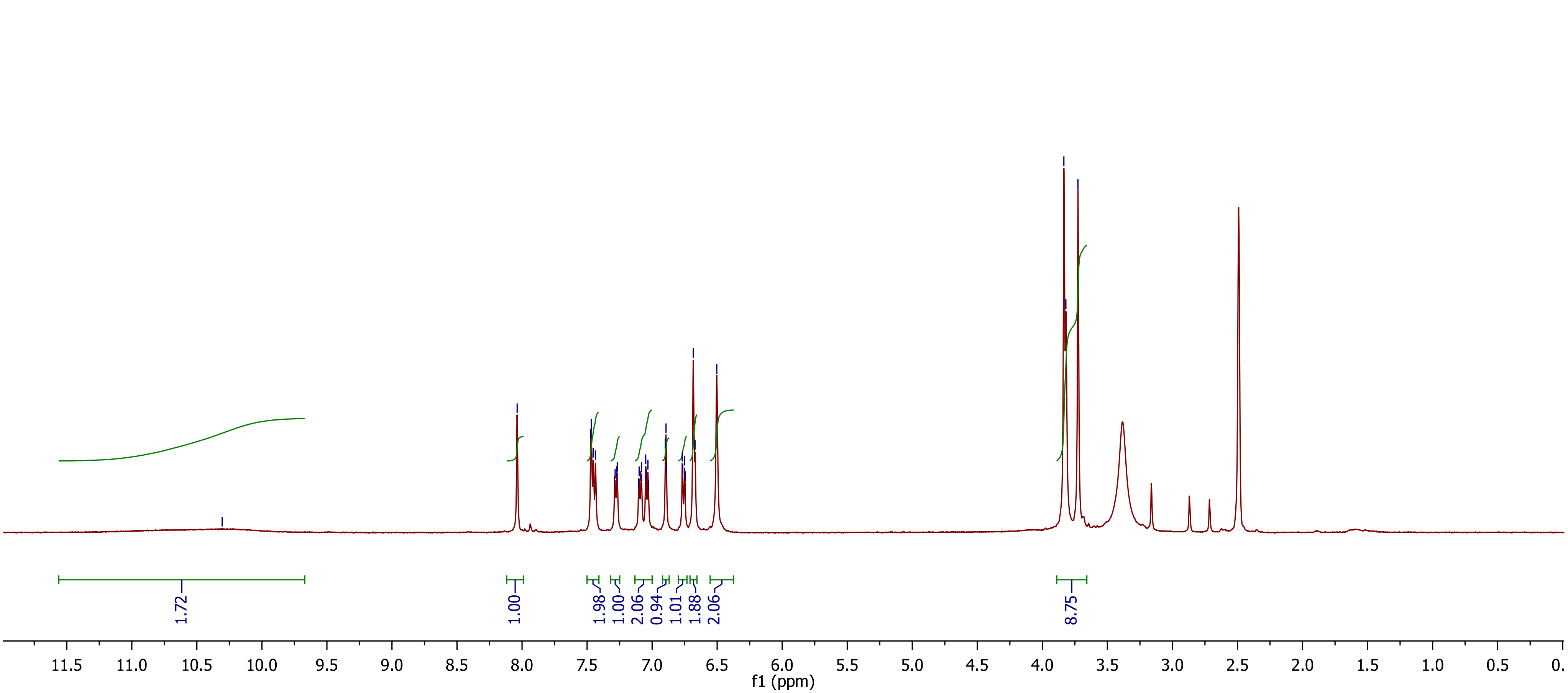

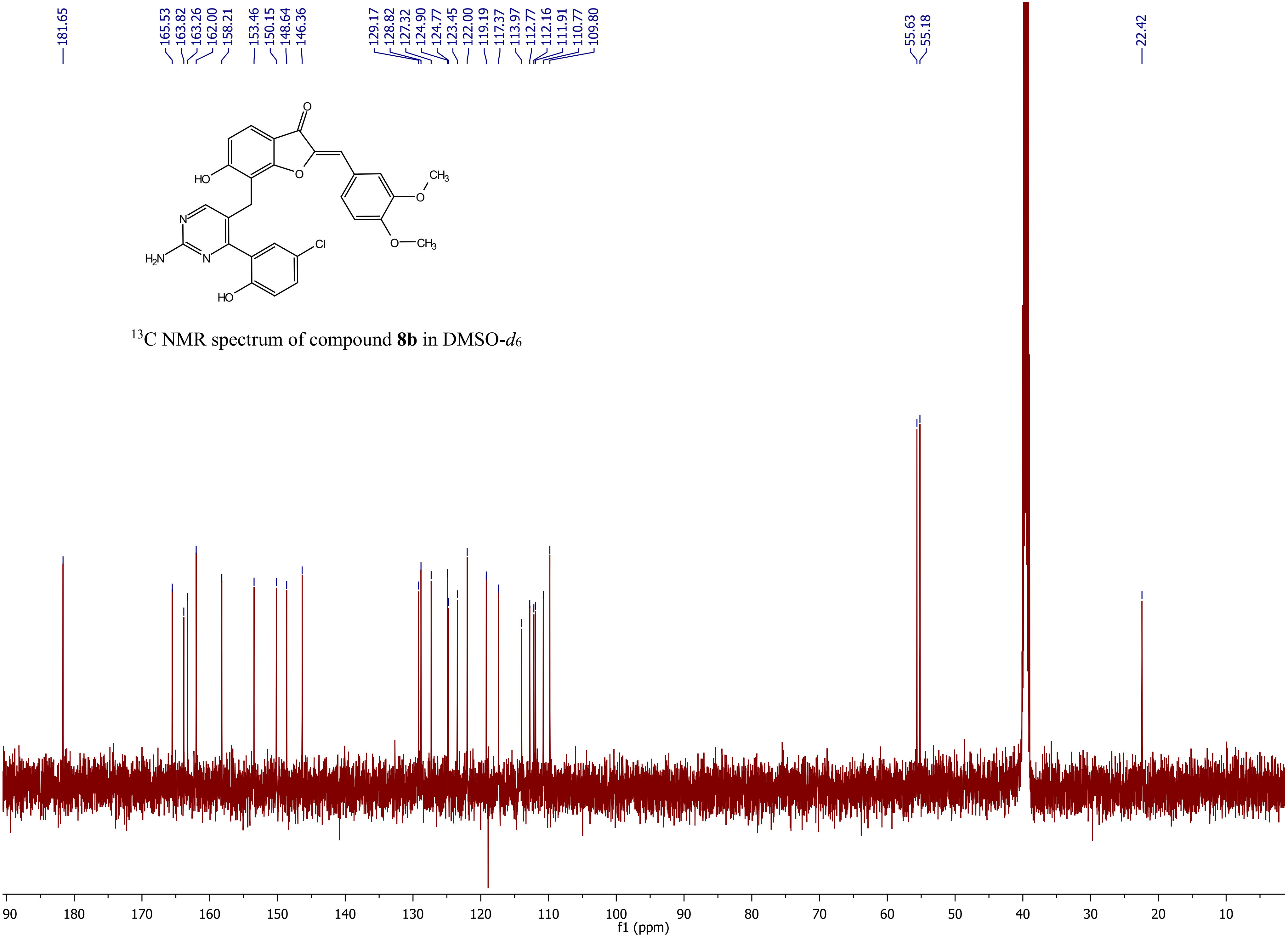

^{13}C NMR spectrum of compound **8b** in DMSO-d_6

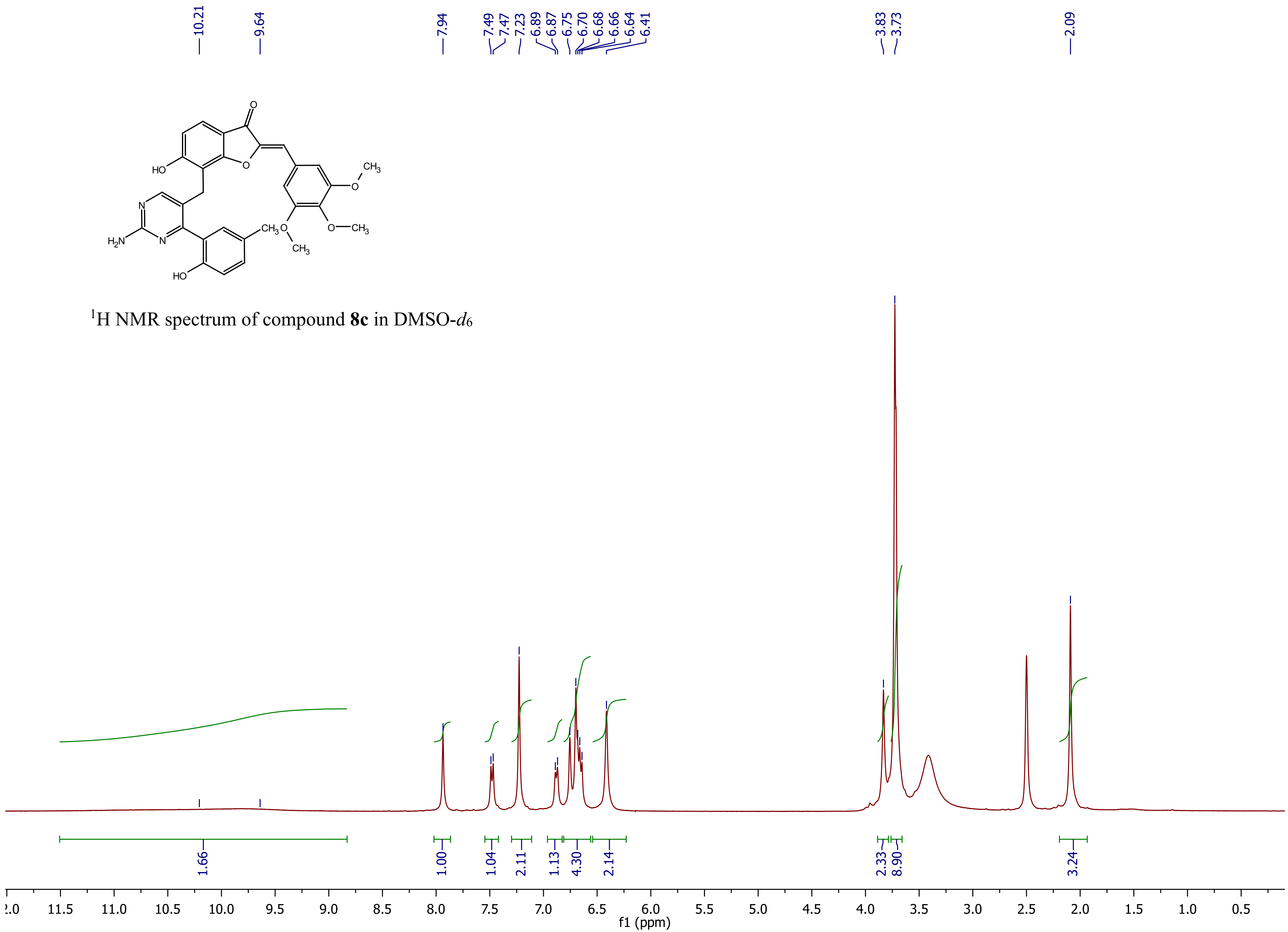

^{1}H NMR spectrum of compound **8c** in DMSO-d_6

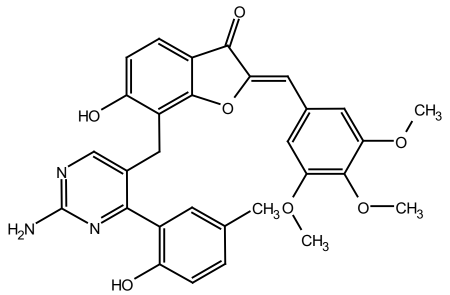

^{13}C NMR spectrum of compound **8c** in DMSO-d_6

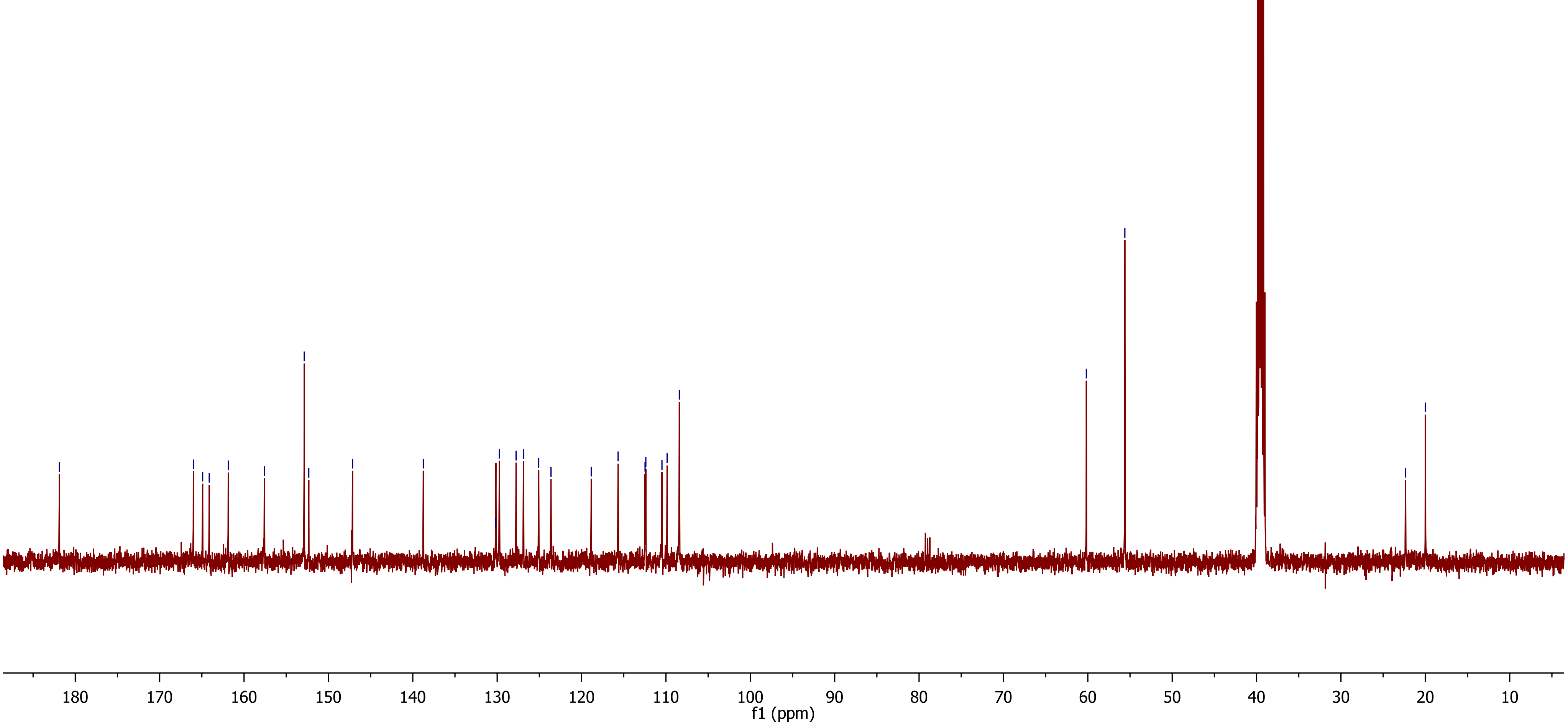

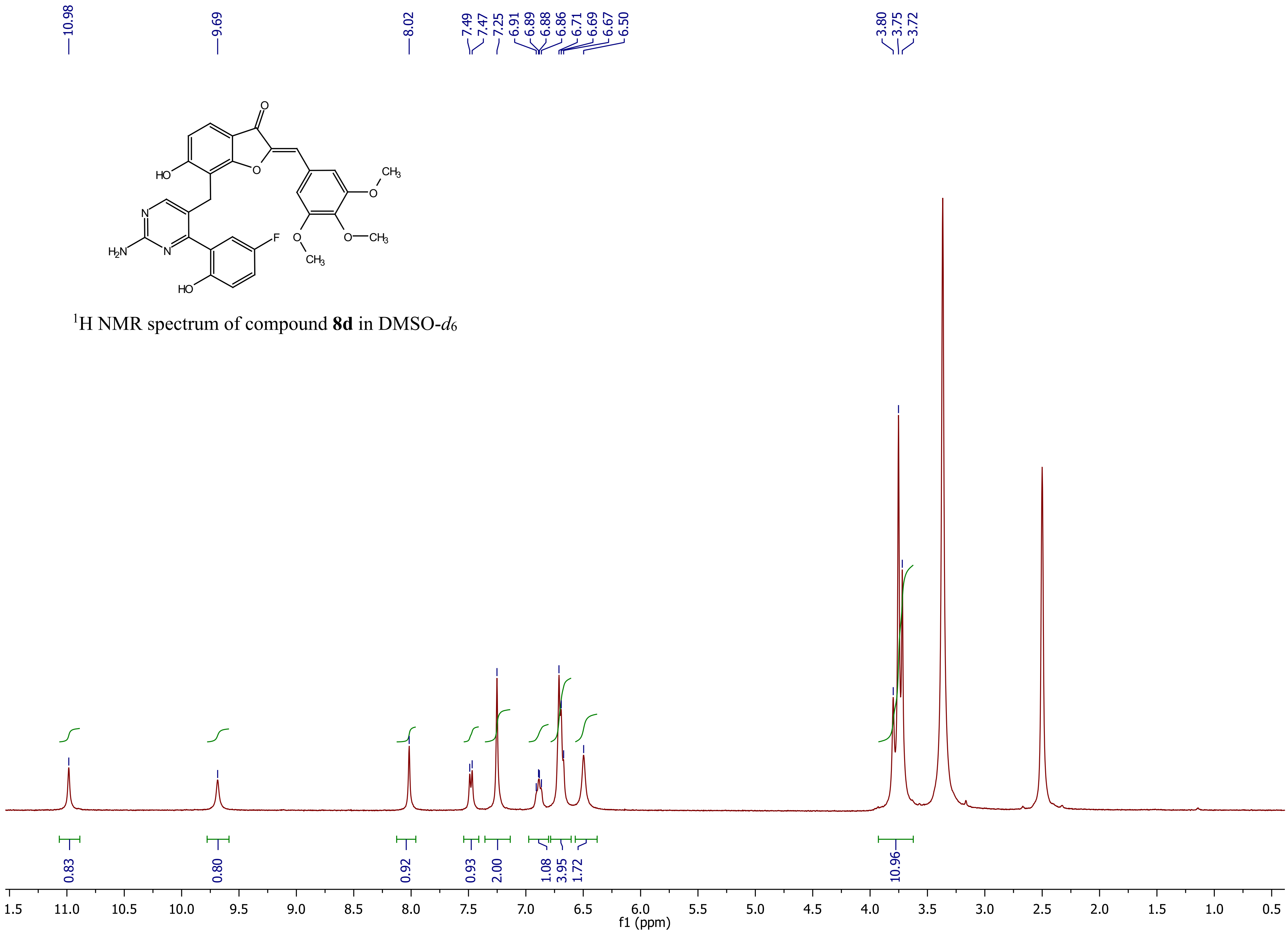

^{1}H NMR spectrum of compound **8d** in DMSO-d_6

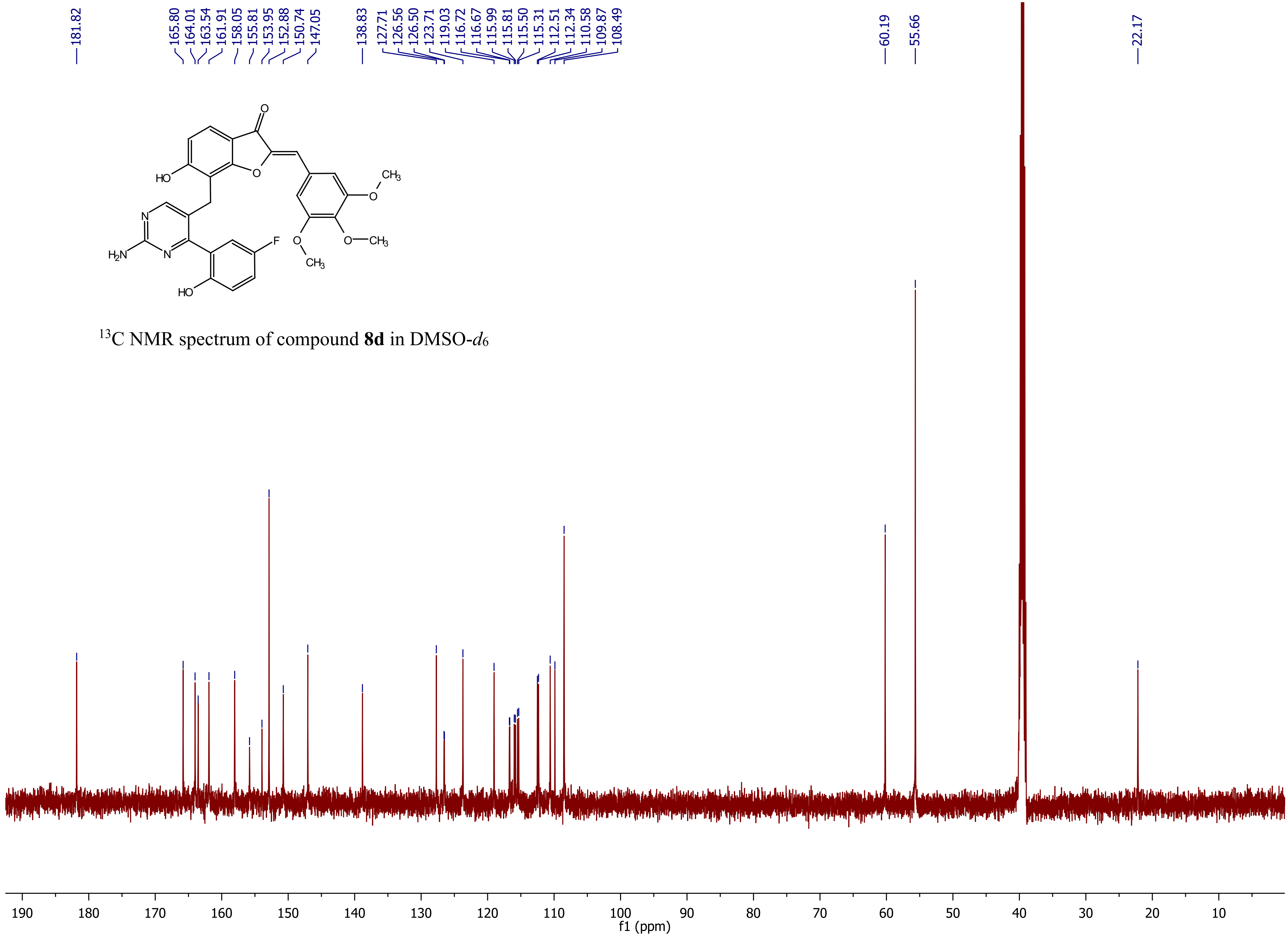

^{13}C NMR spectrum of compound **8d** in DMSO-*d*$_6$

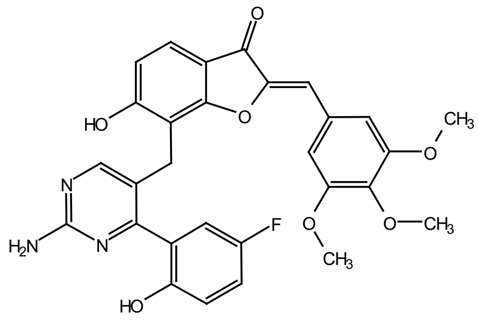

^{19}F NMR spectrum of compound **8d** in DMSO-*d6*

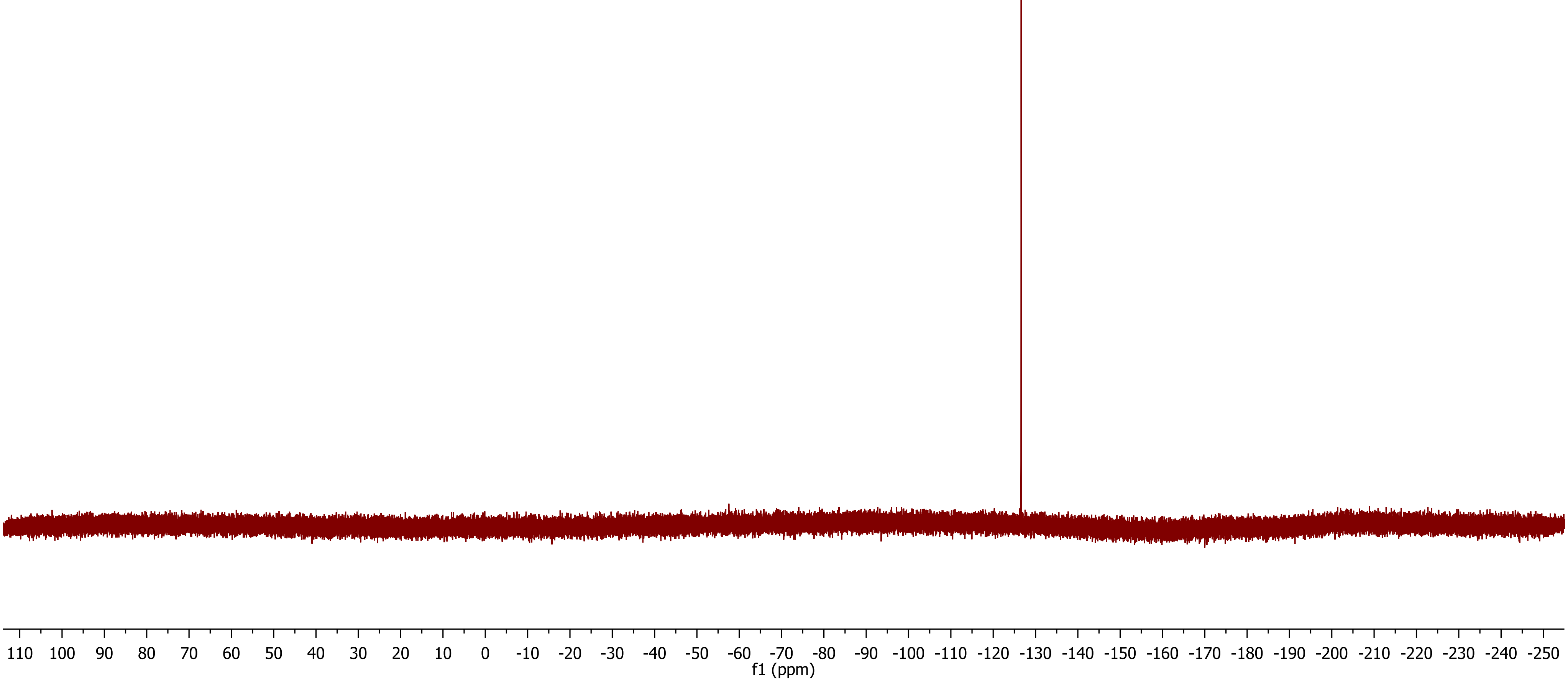

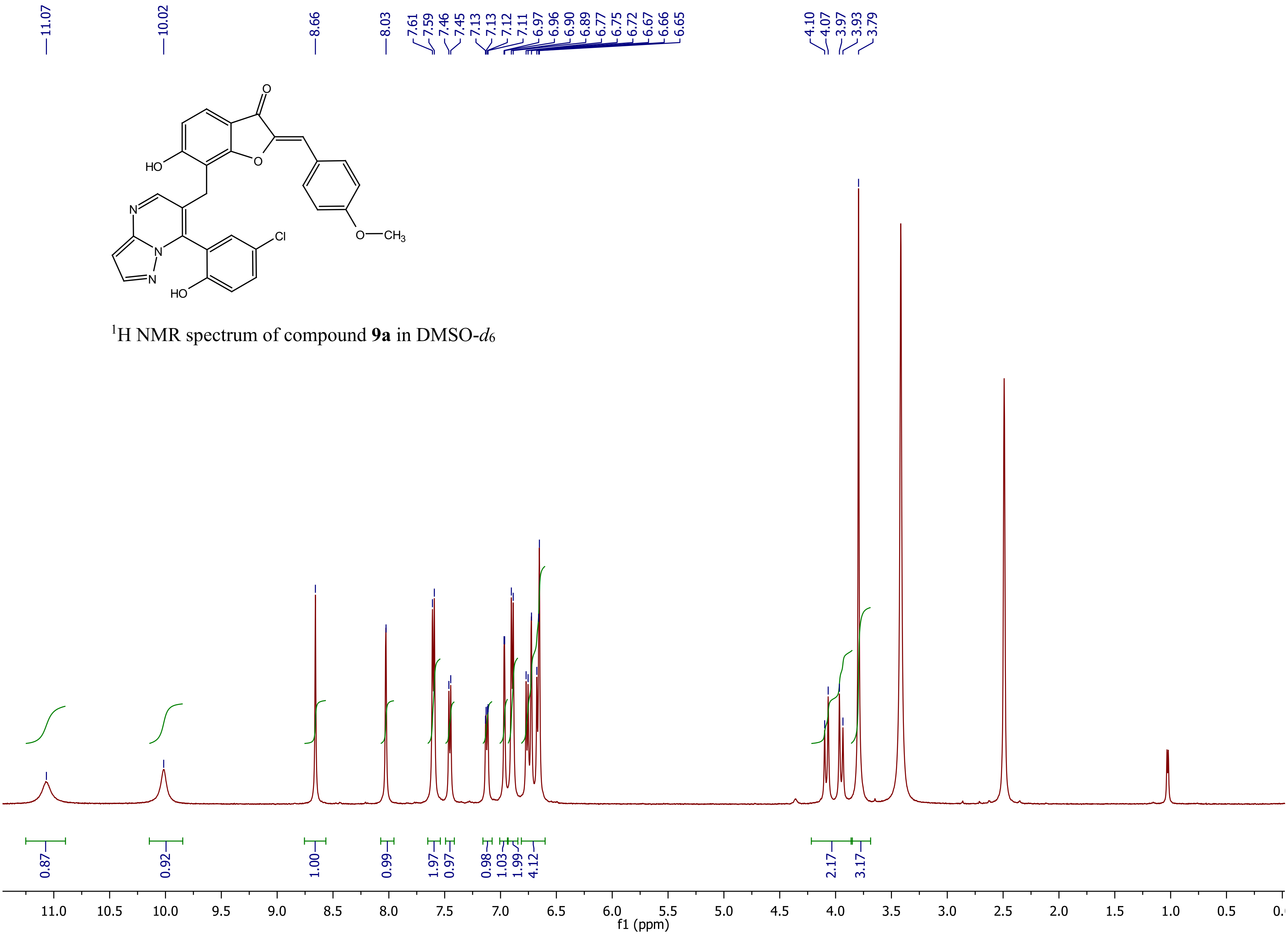

^{1}H NMR spectrum of compound **9a** in DMSO-d_6

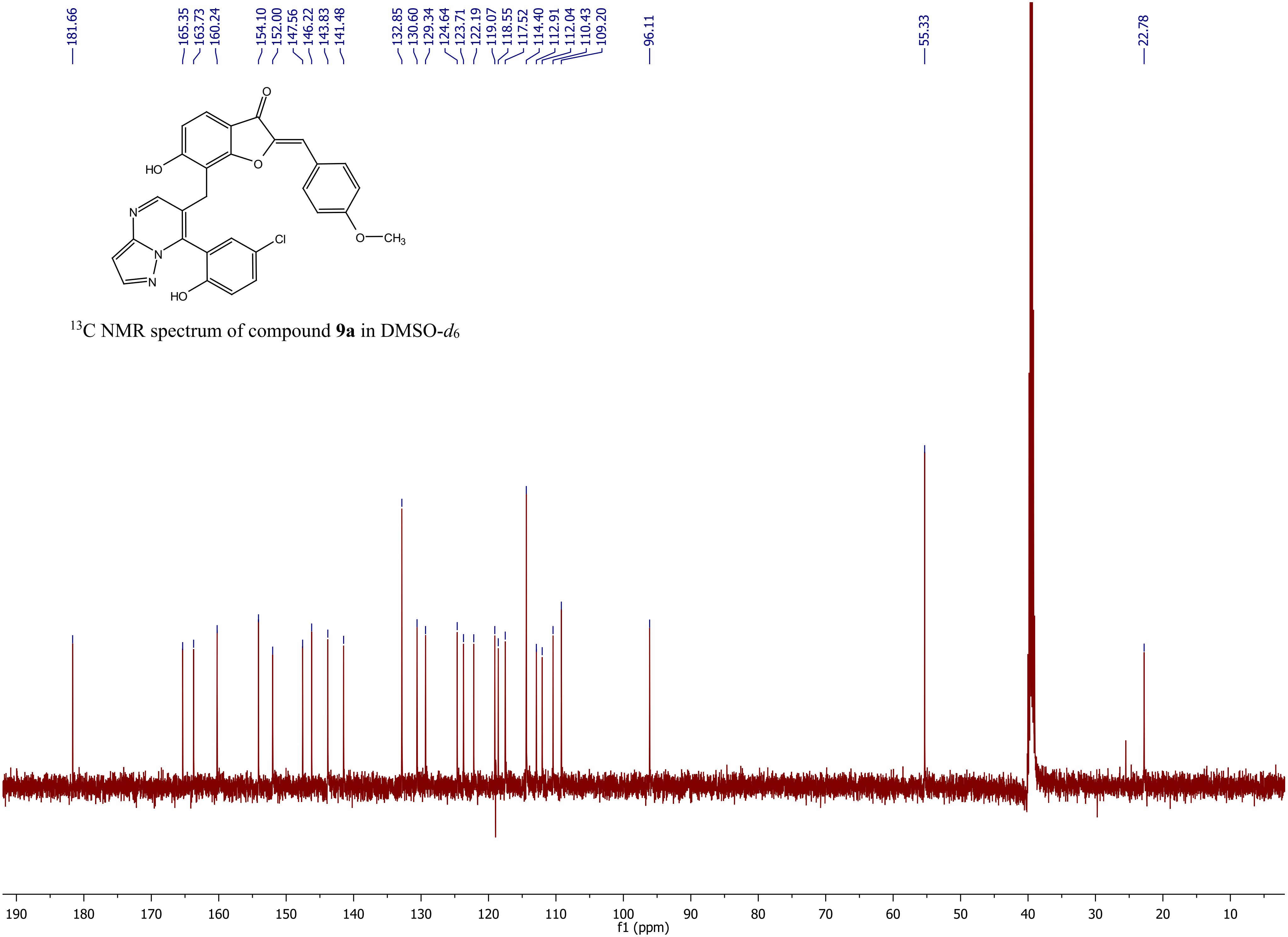

^{13}C NMR spectrum of compound **9a** in DMSO-d_6

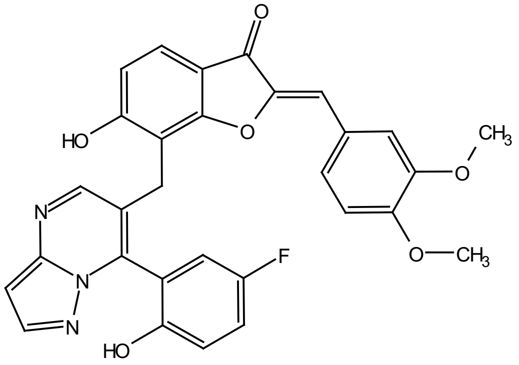

^{1}H NMR spectrum of compound **9b** in DMSO-d_6

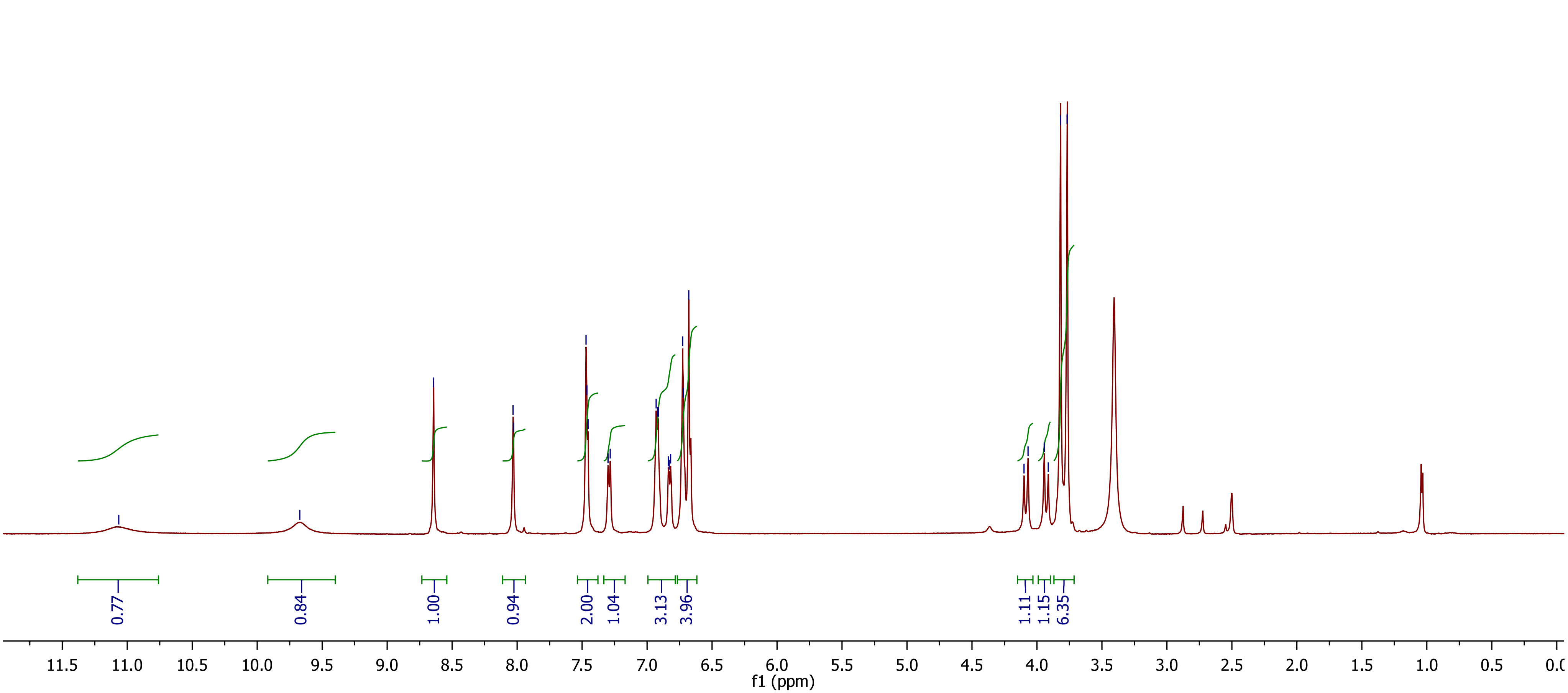

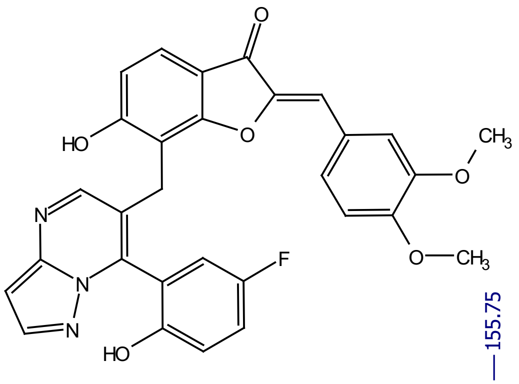

^{13}C NMR spectrum of compound **9b** in DMSO-d_6

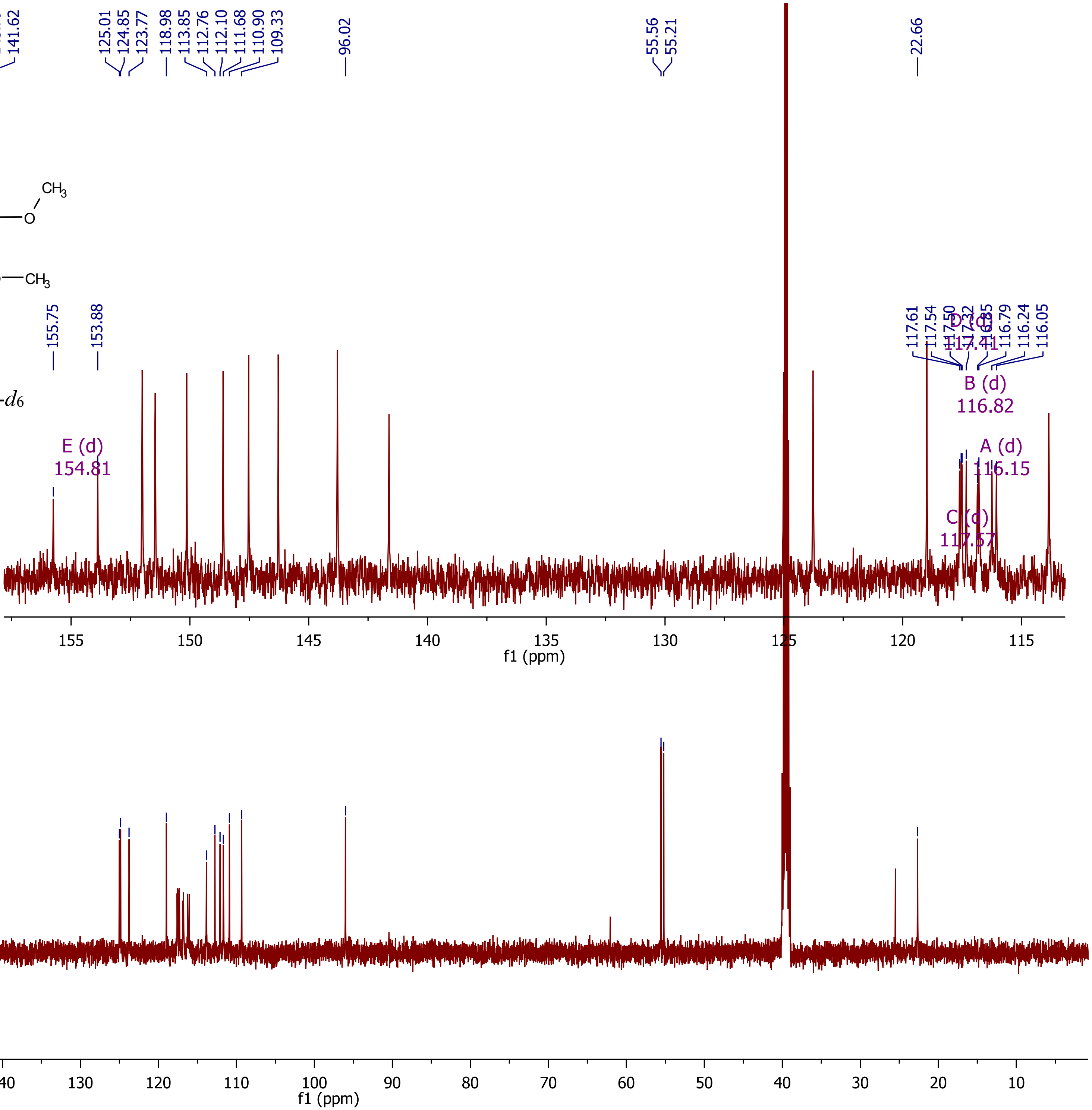

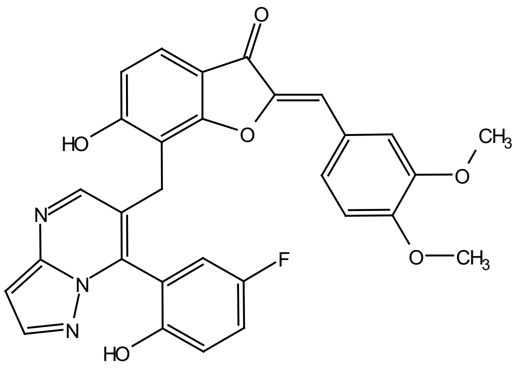

^{19}F NMR spectrum of compound **9b** in DMSO-d_6

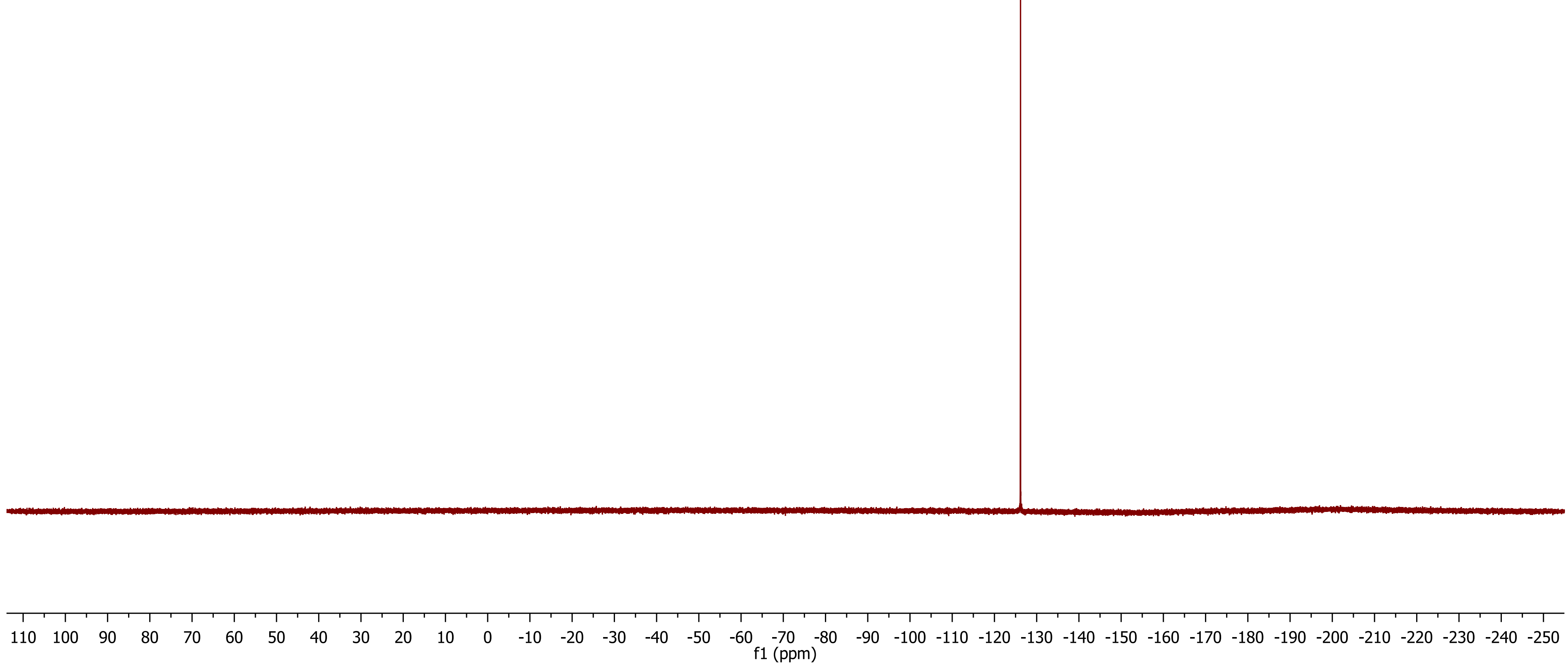

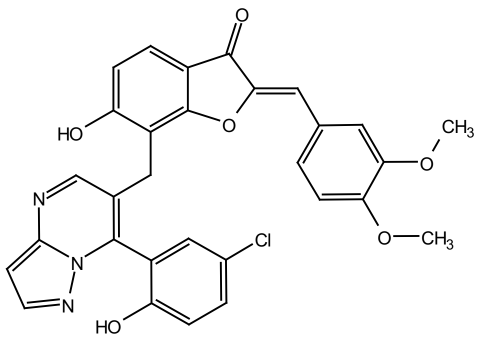

^{1}H NMR spectrum of compound **9c** in DMSO-d_6

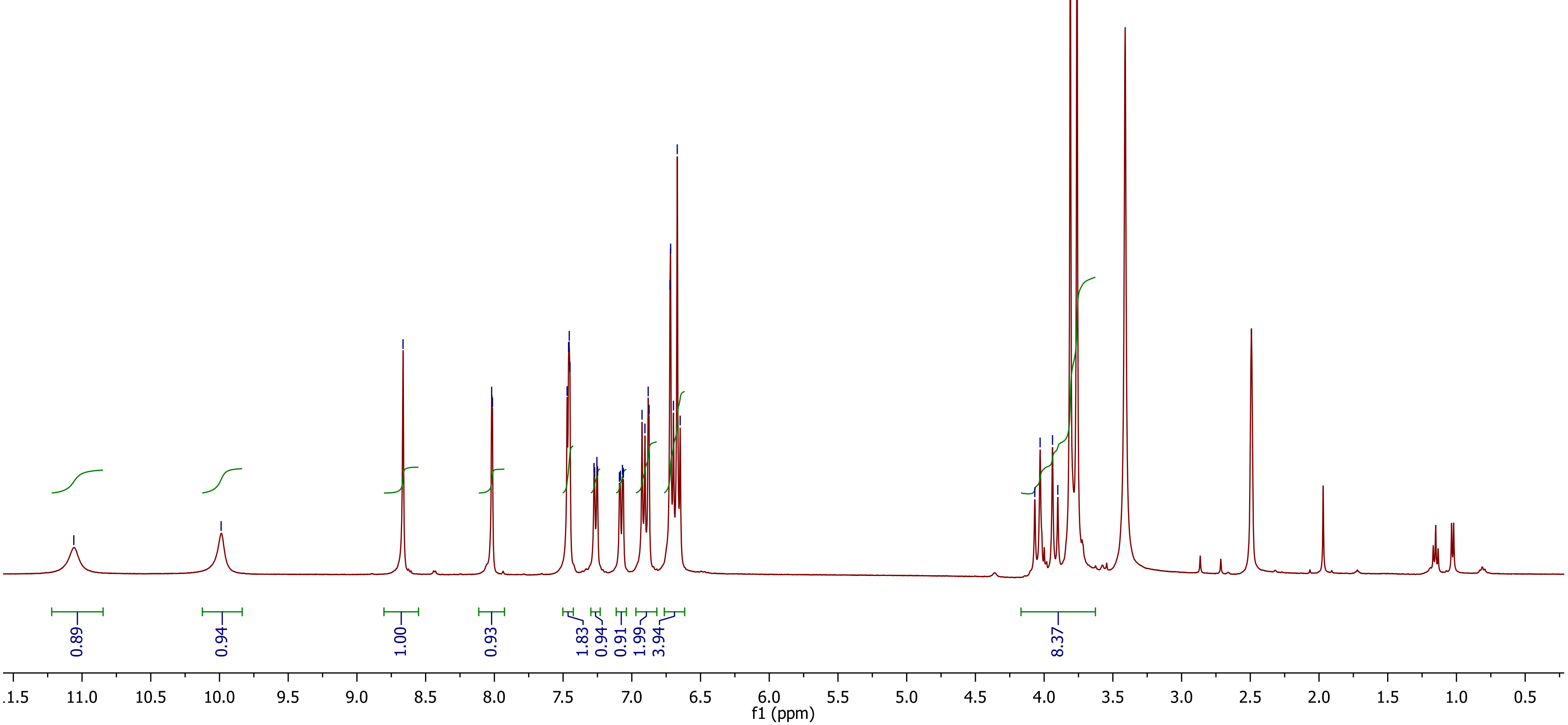

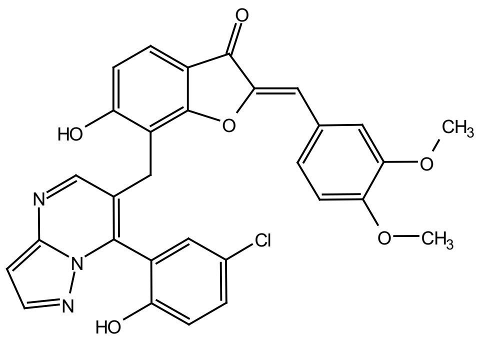

^{13}C NMR spectrum of compound **9c** in DMSO-d_6

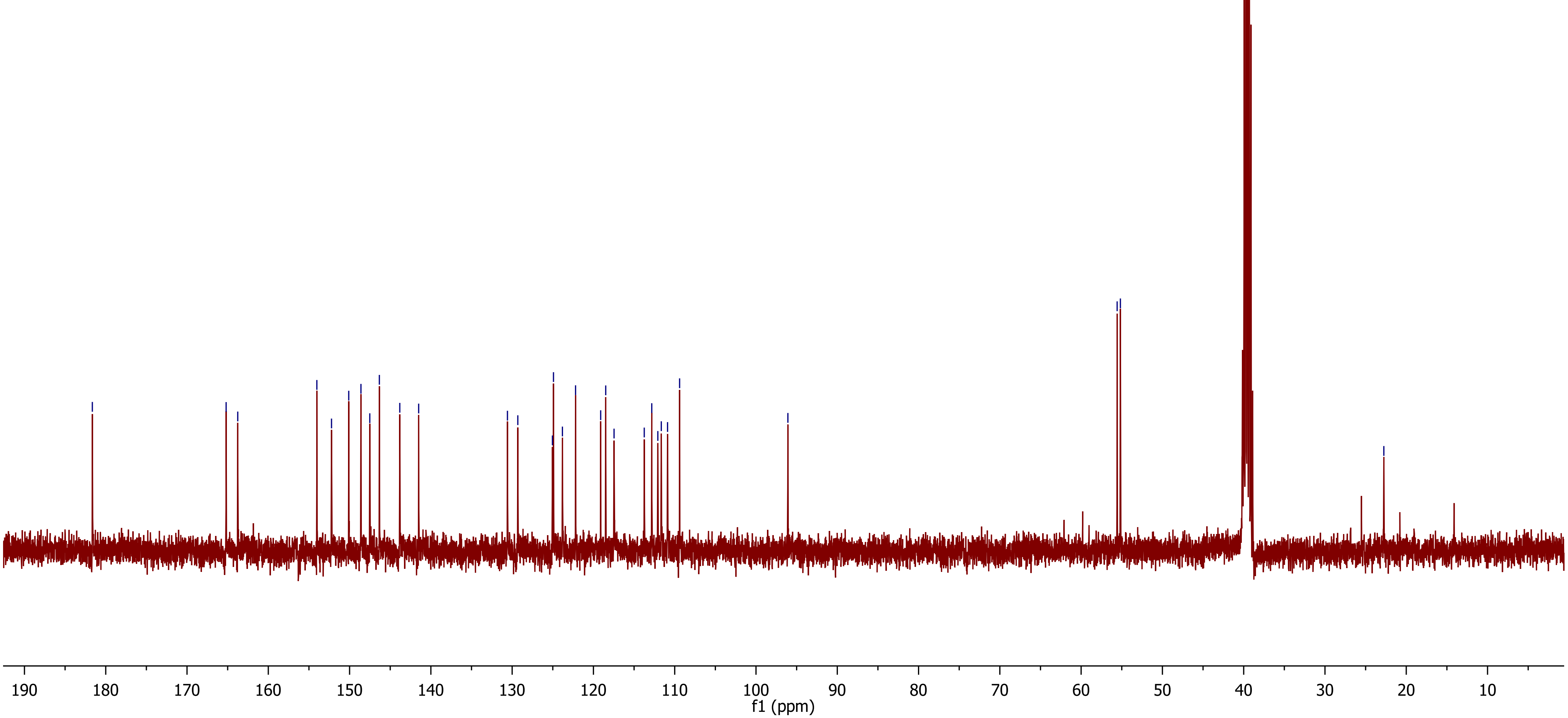

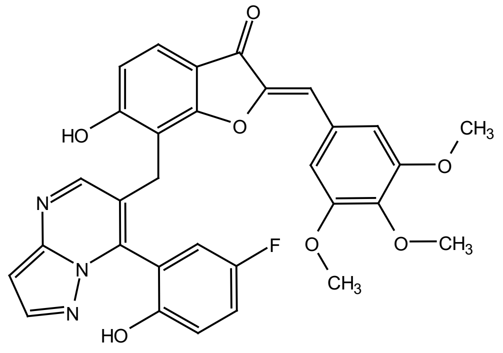

^{1}H NMR spectrum of compound **9d** in DMSO-d_6

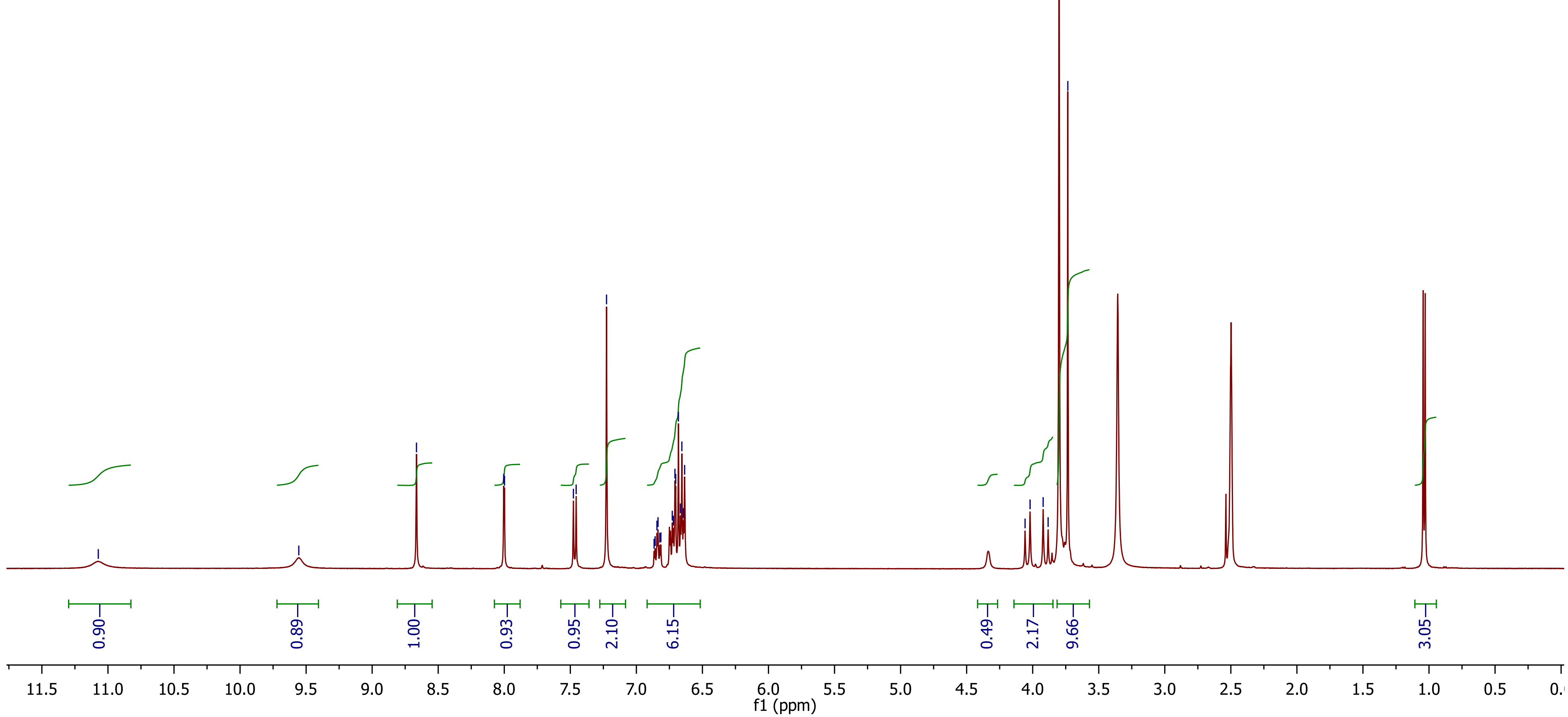

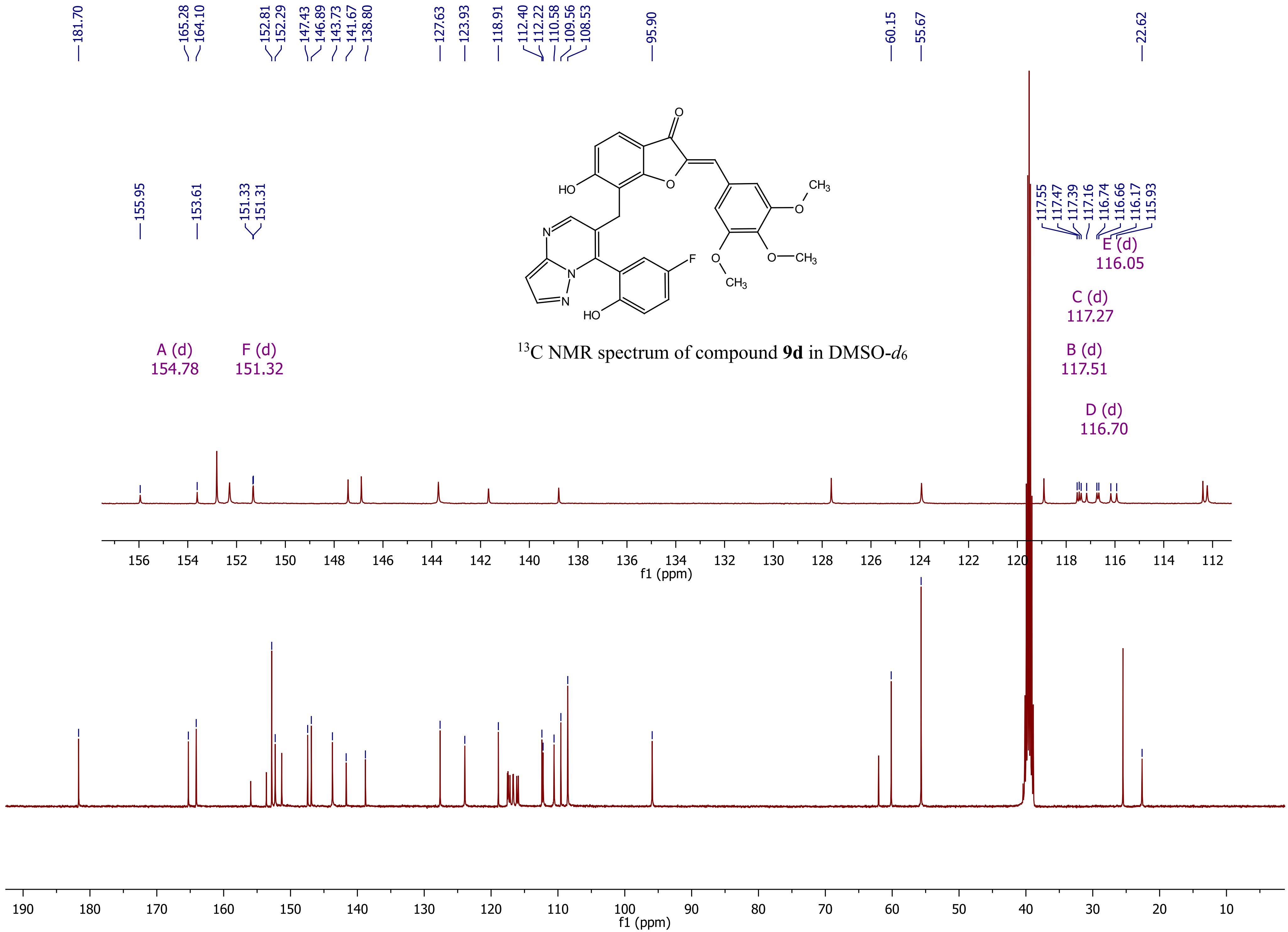

^{13}C NMR spectrum of compound **9d** in DMSO-d_6

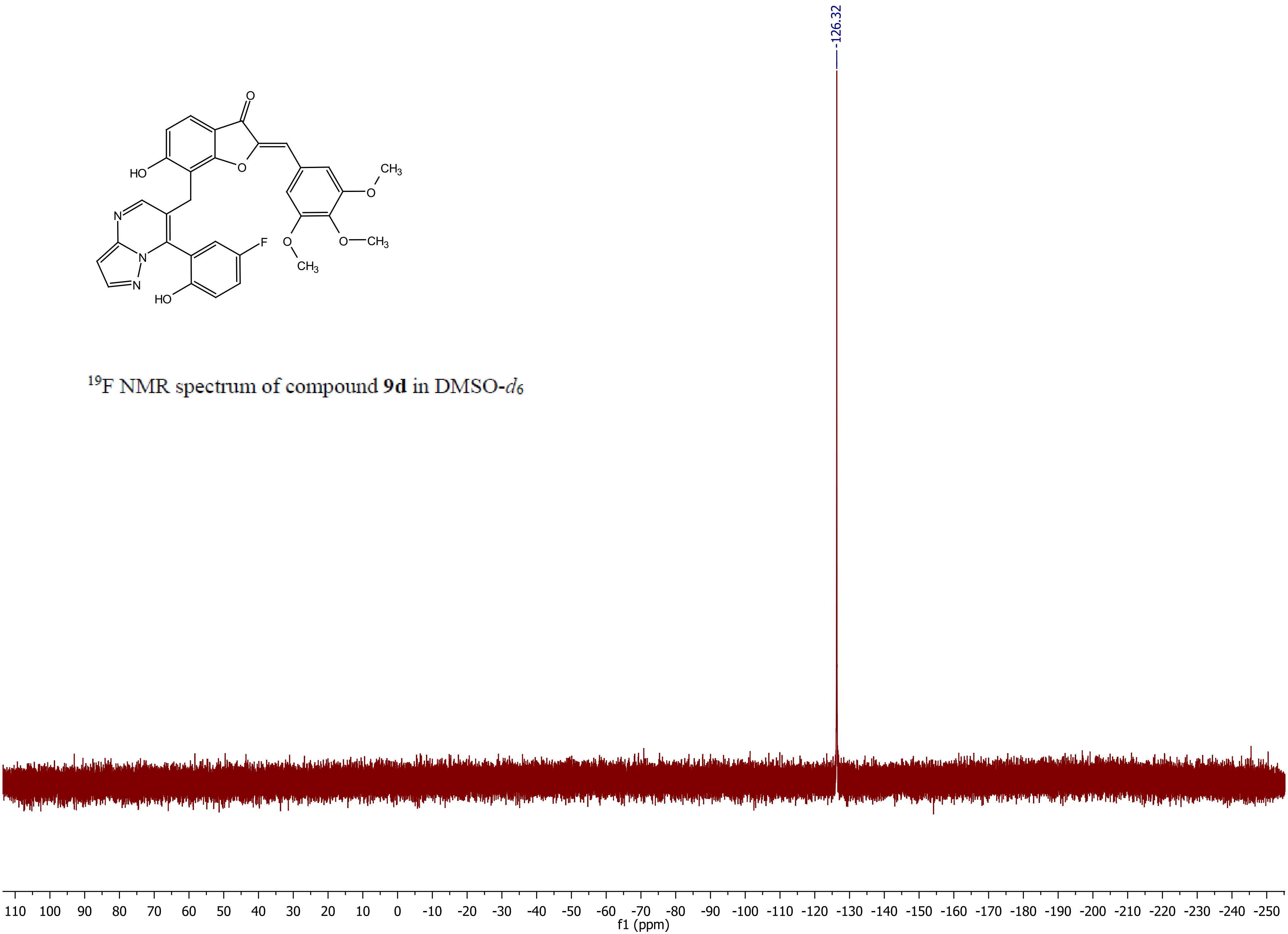

^{19}F NMR spectrum of compound **9d** in DMSO-*d*$_6$

MaxPeak: 100.00%
Ret_Time: 1.622 min

IBOX14033

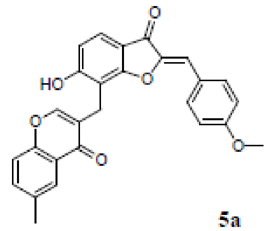

5a

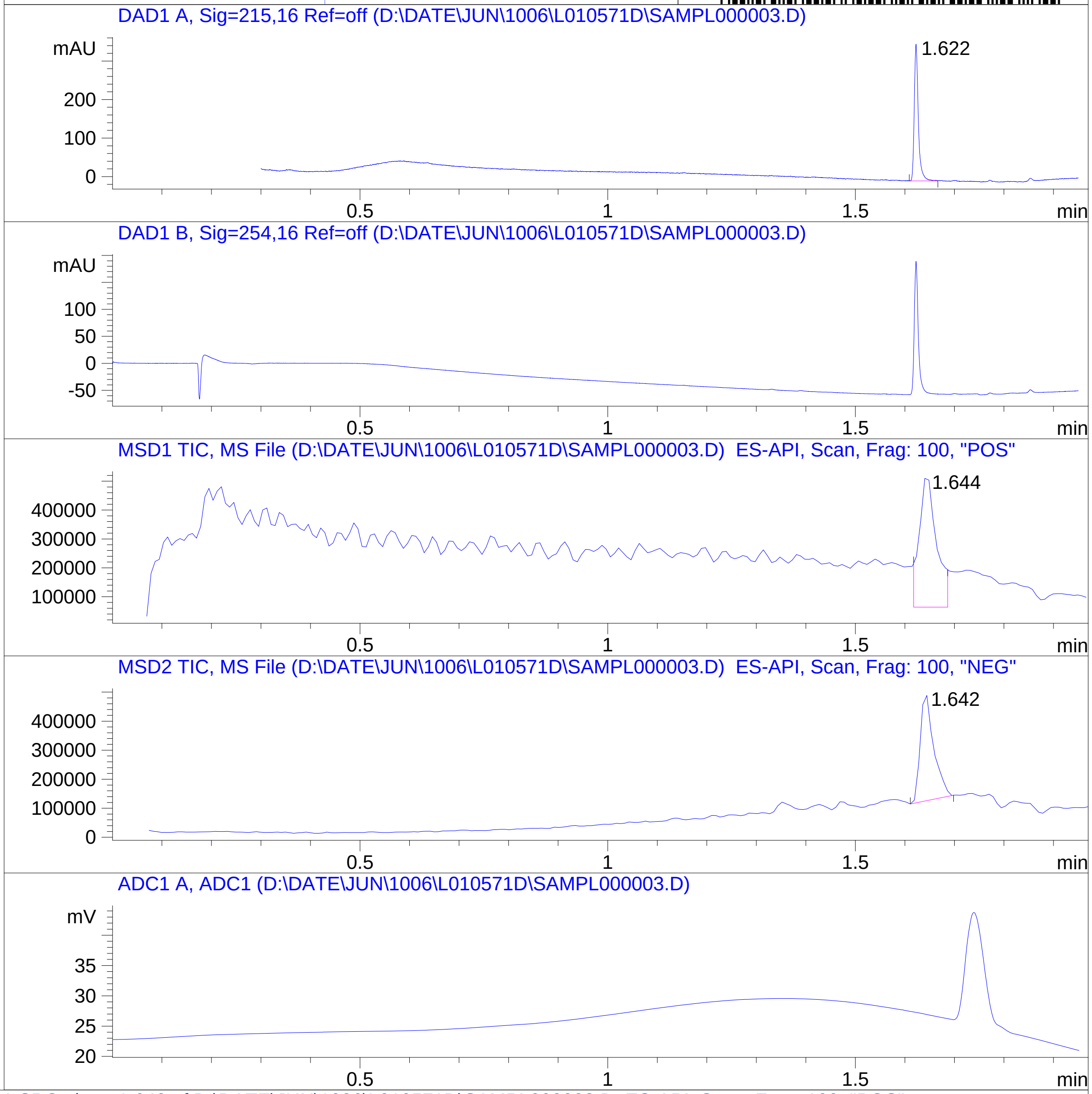

Mol Wt 0

Exact Mass

#	Time	Area%
1	1.622	100.00

RT 1.644

RT 1.642

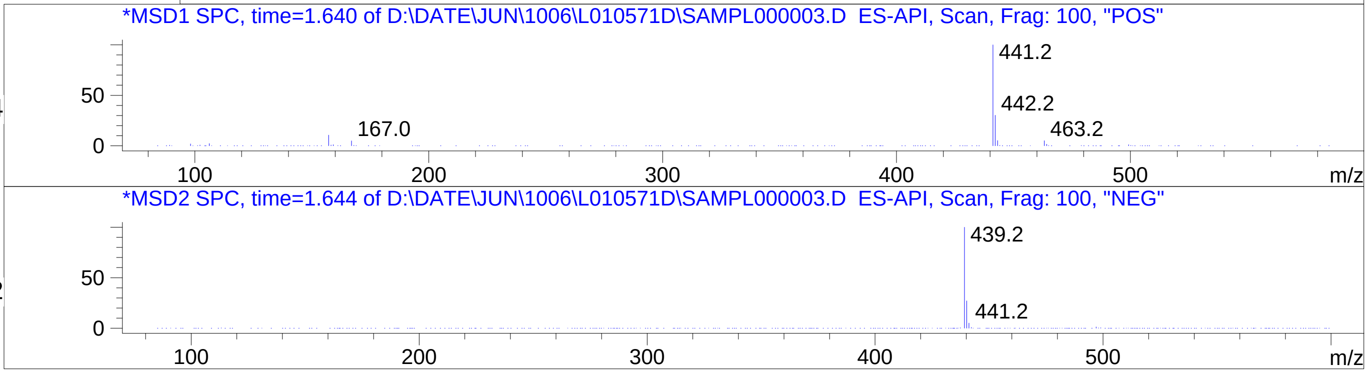

```
MaxPeak:  100.00%
Ret_Time: 1.560 min
```

IBOX14826

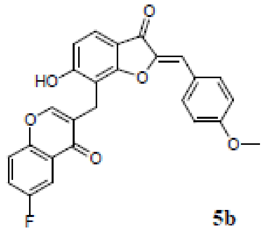

5b

Mol Wt 0

Exact Mass

#	Time	Area%
1	1.560	100.00

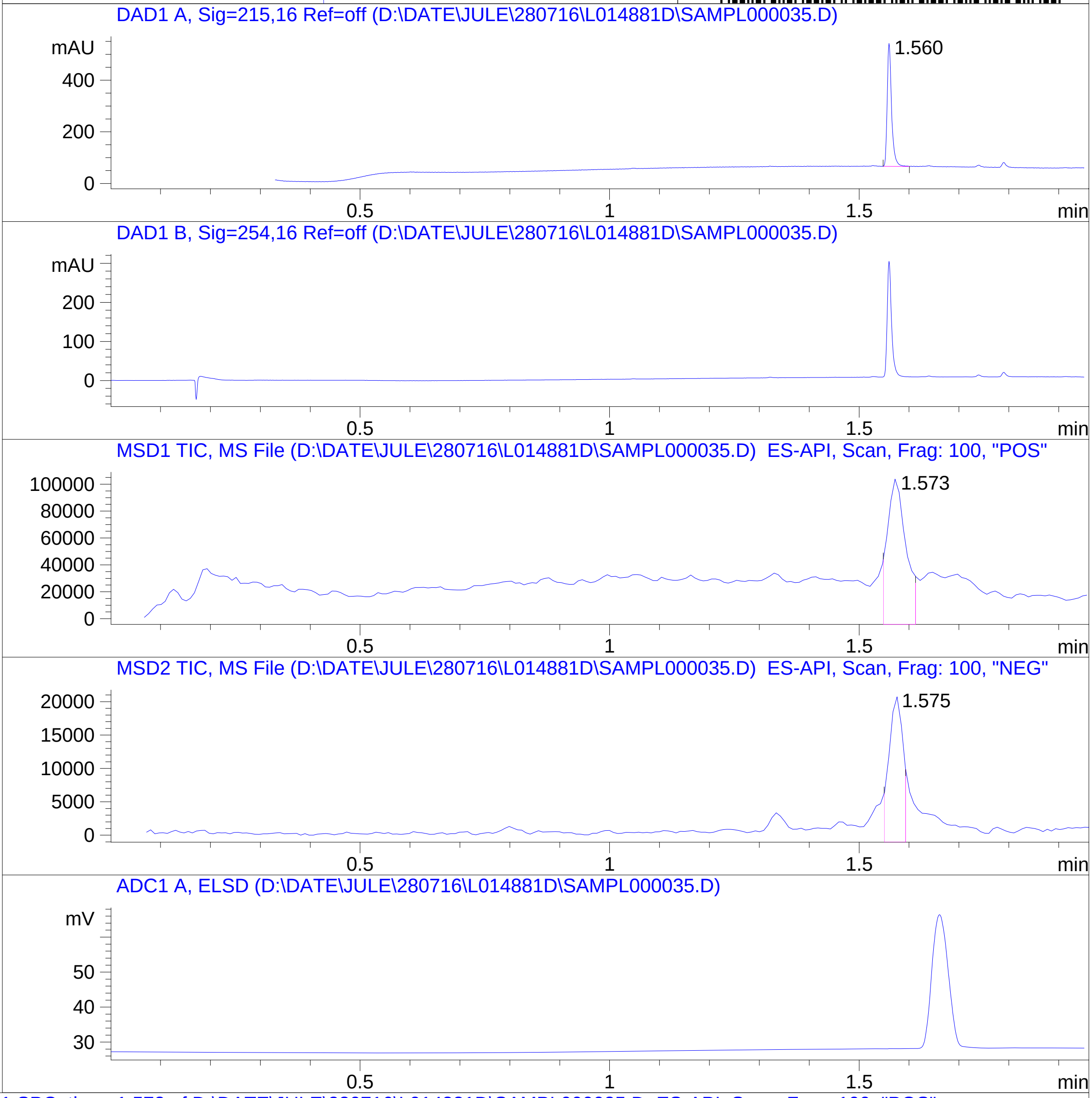

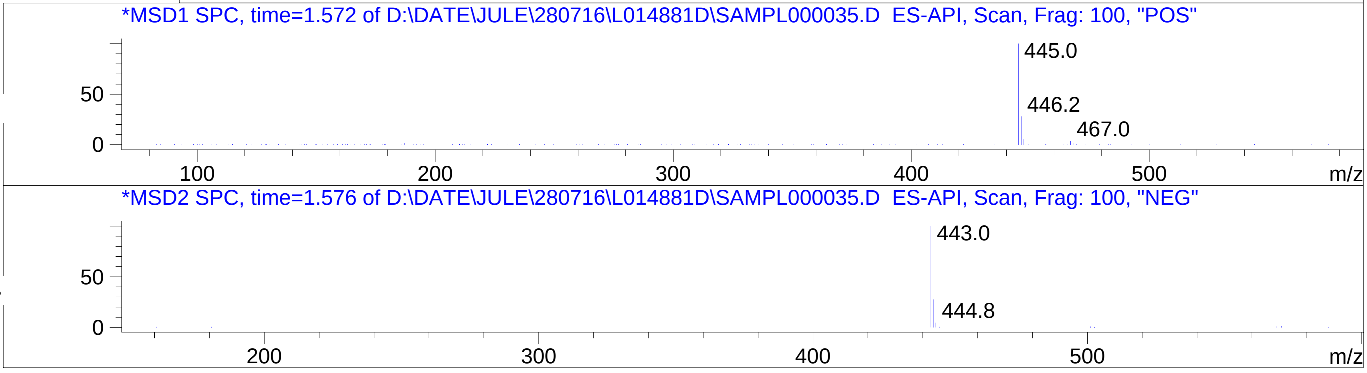

Inj.Date 7/28/2016 T P2-C-09 -12- Acq. Method C:\CHEM32\-> ->

MaxPeak: 100.00%
Ret_Time: 1.553 min

IBOX21764

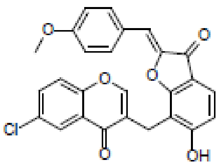

5c

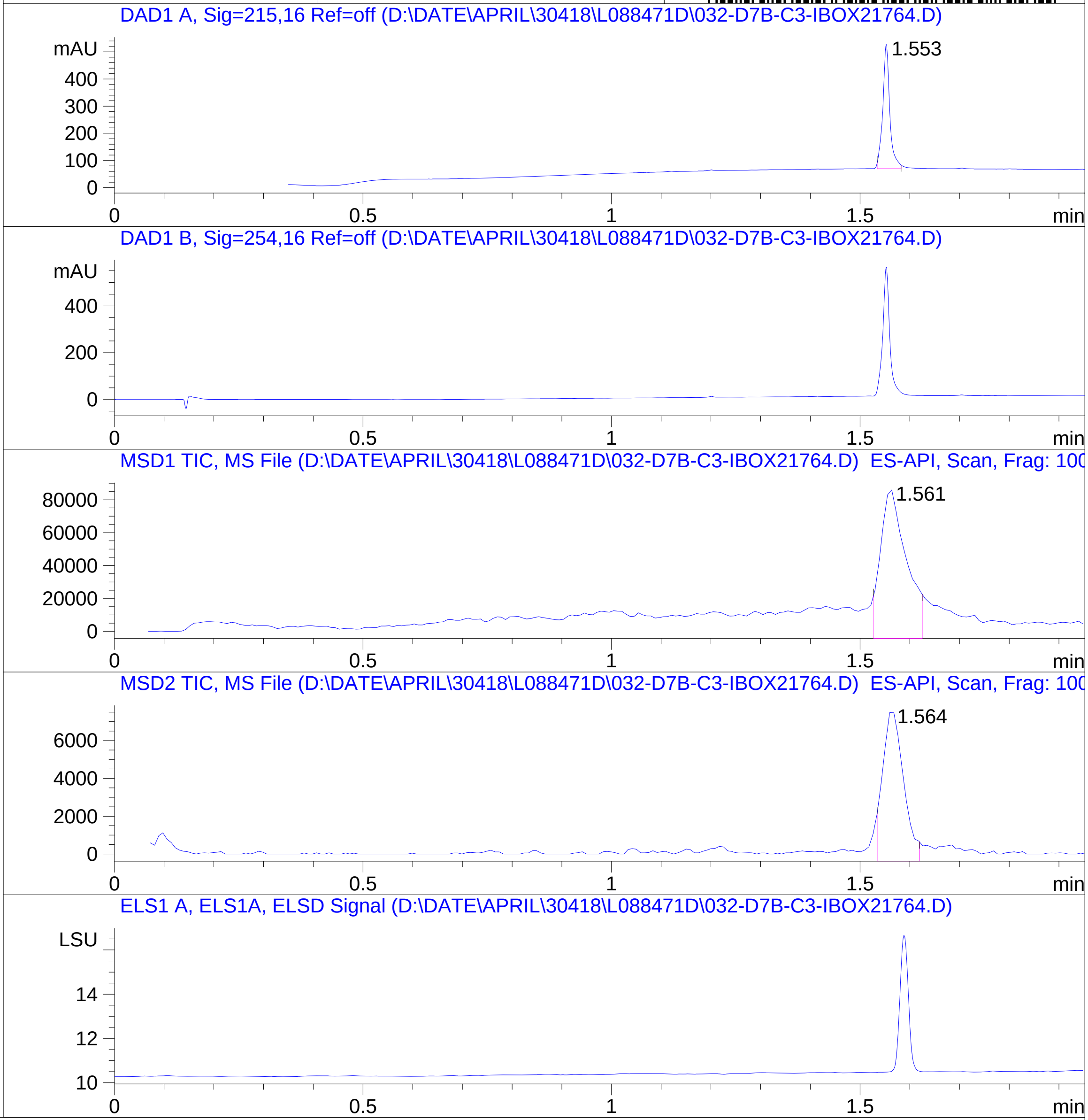

Mol Wt 0

Exact Mass

#	Time	Area%
1	1.553	100.00

RT 1.561

RT 1.564

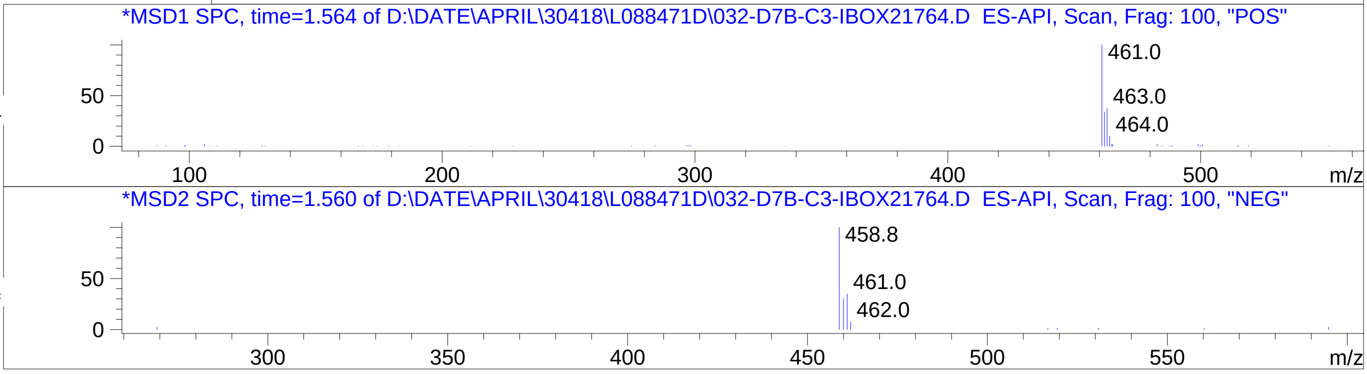

MaxPeak: 76.85%
Ret_Time: 1.547 min

IBOX14030

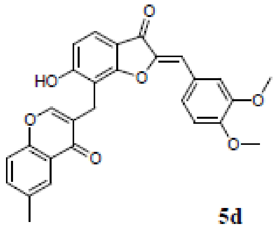

5d

Mol Wt 0

Exact Mass

#	Time	Area%
1	0.326	10.46
2	1.547	76.85
3	1.810	12.69

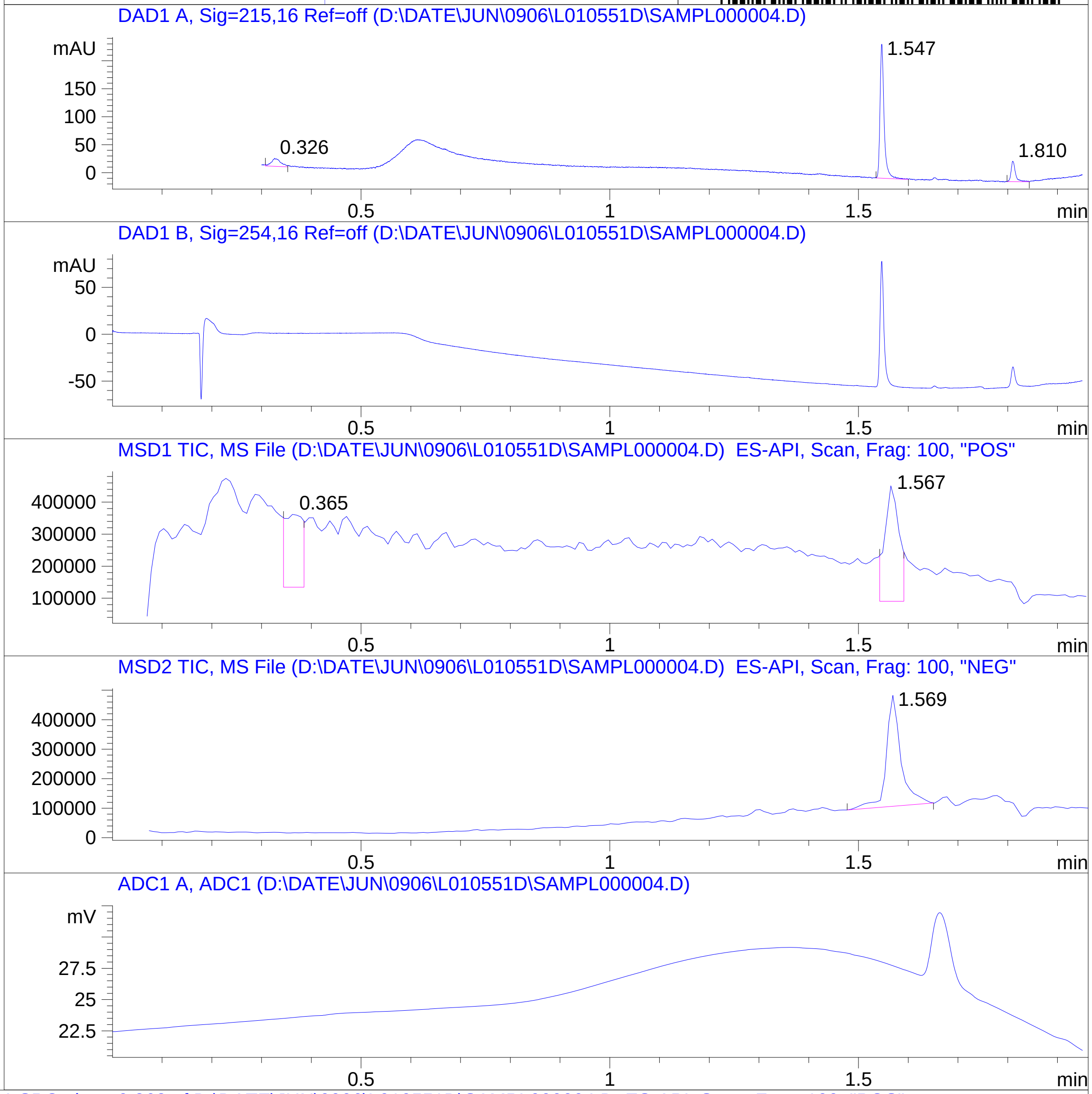

RT 0.365

RT 1.567

RT 1.569

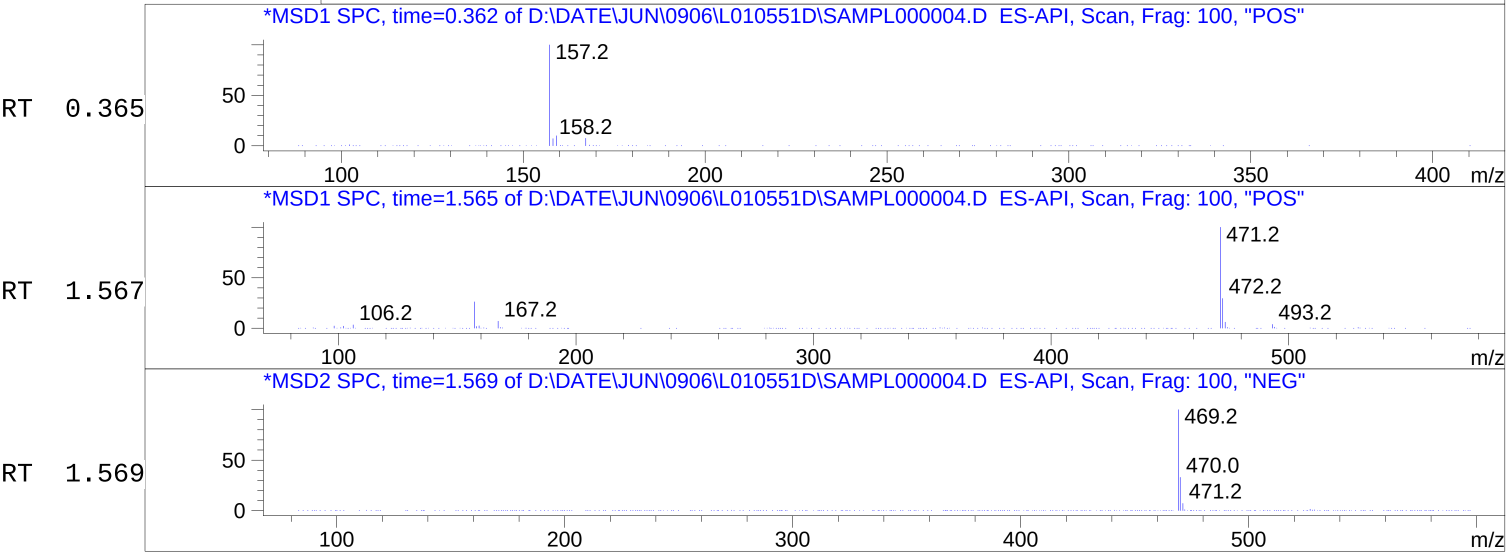

Inj.Date 6/9/2016 A P2-A-04 - 4 - Acq. Method C:\CHEM32\-> ->

MaxPeak: 92.37%
Ret_Time: 1.412 min

IBOX21908

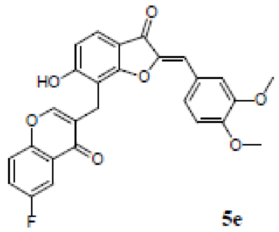

5e

Mol Wt 0

Exact Mass

#	Time	Area%
1	1.177	1.09
2	1.412	92.37
3	1.671	6.54

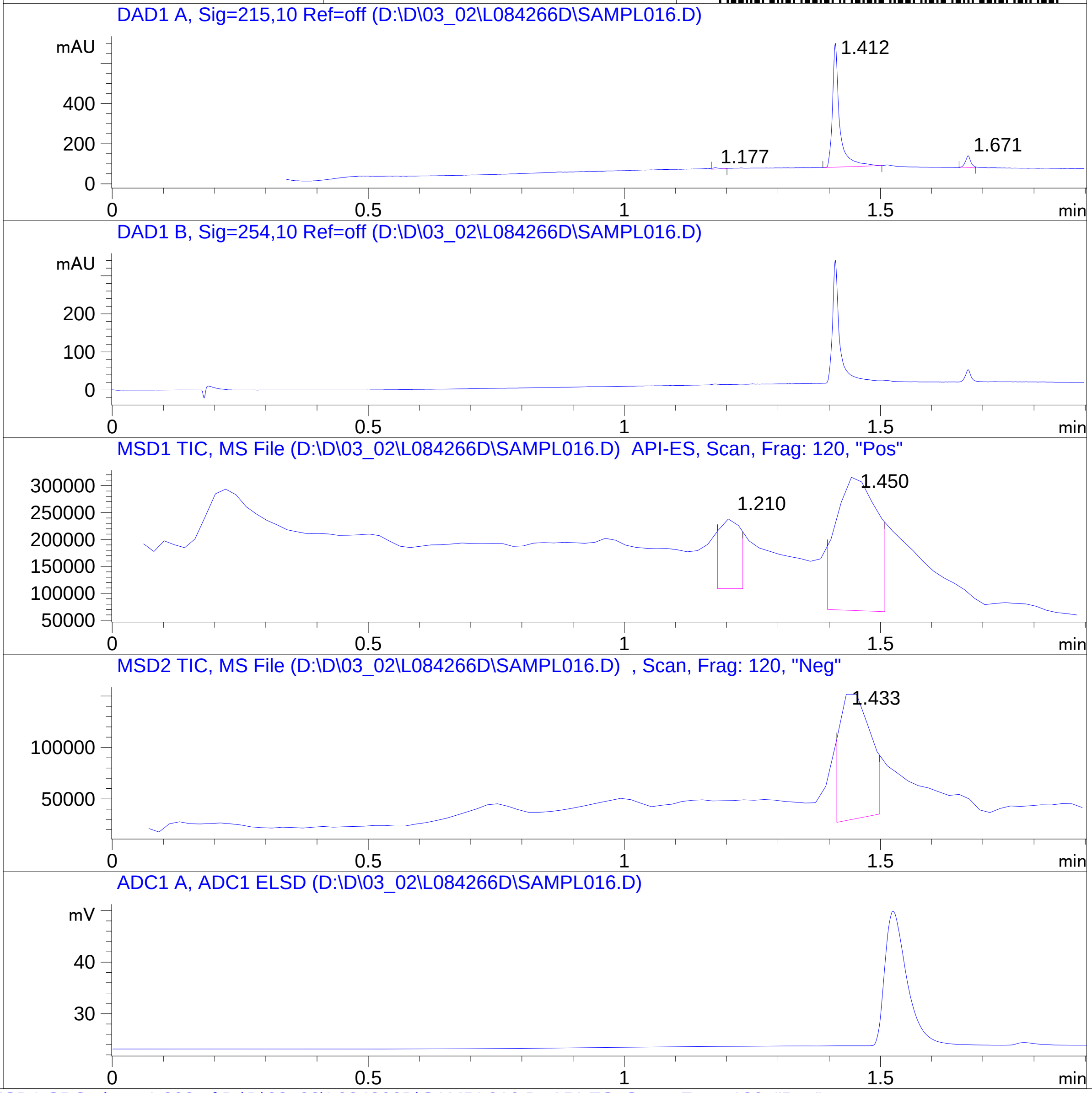

RT 1.210

RT 1.450

RT 1.433

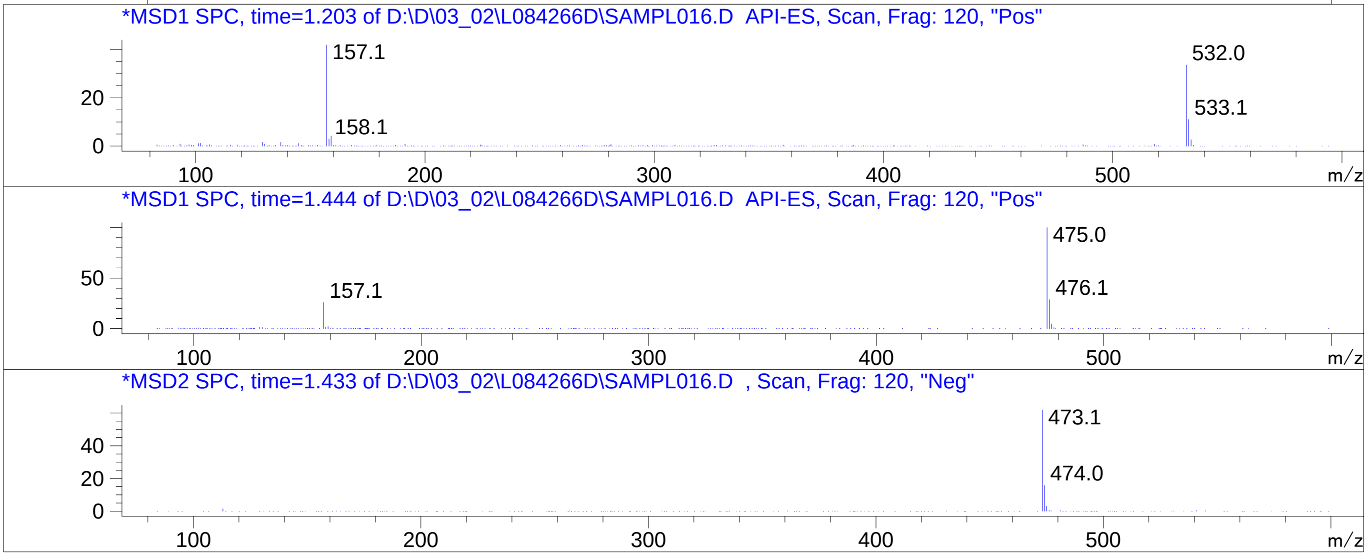

Inj.Date 3/3/2018 N P2-B-04 S85 VL Acq. Method C:\HPCHEM\-> ->

MaxPeak: 82.03%
Ret_Time: 1.552 min

IBOX14820

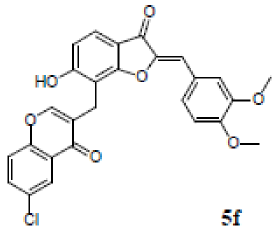

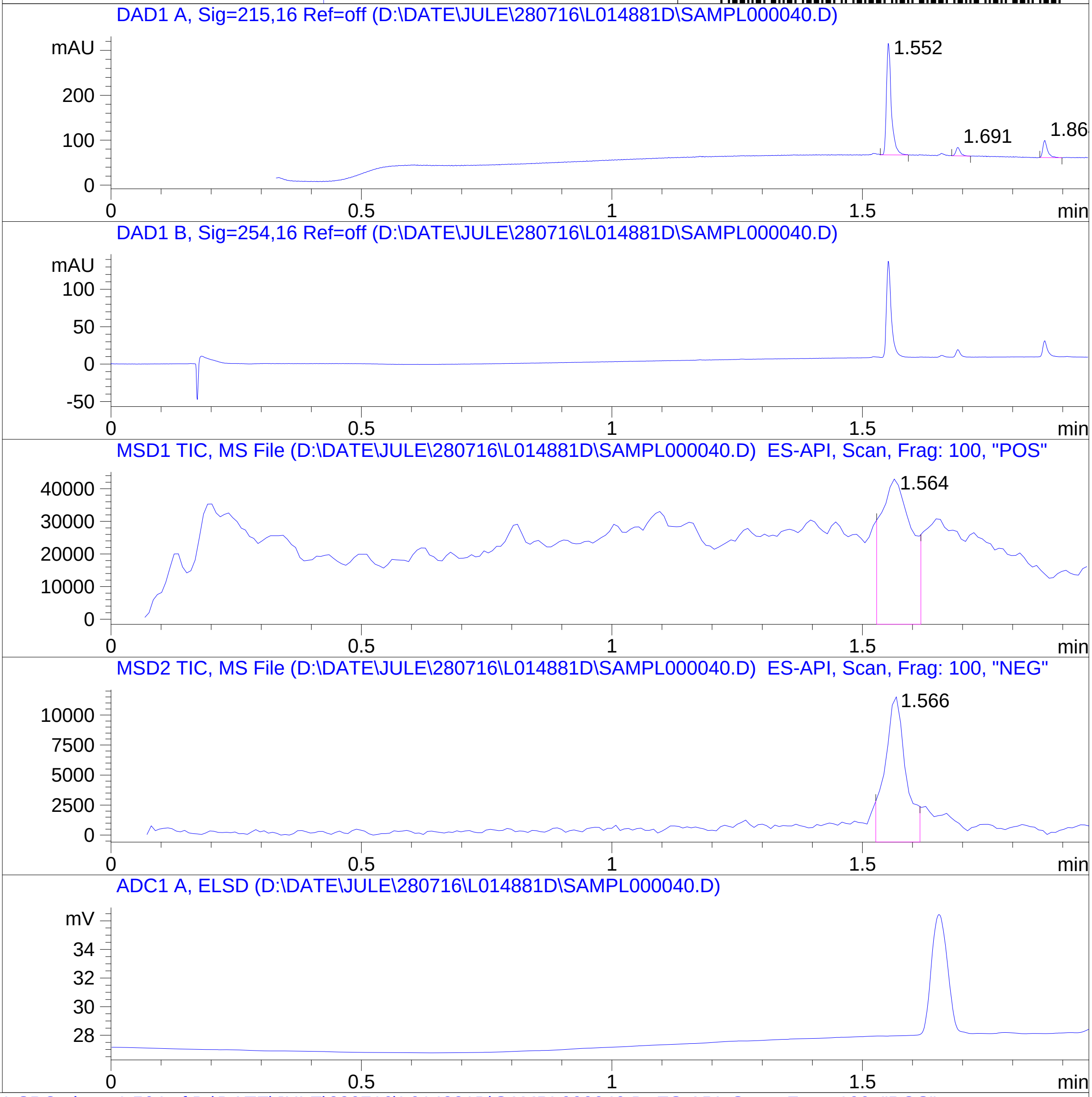

Mol Wt 0

Exact Mass

#	Time	Area%
1	1.552	82.03
2	1.691	5.91
3	1.864	12.06

RT 1.564

RT 1.566

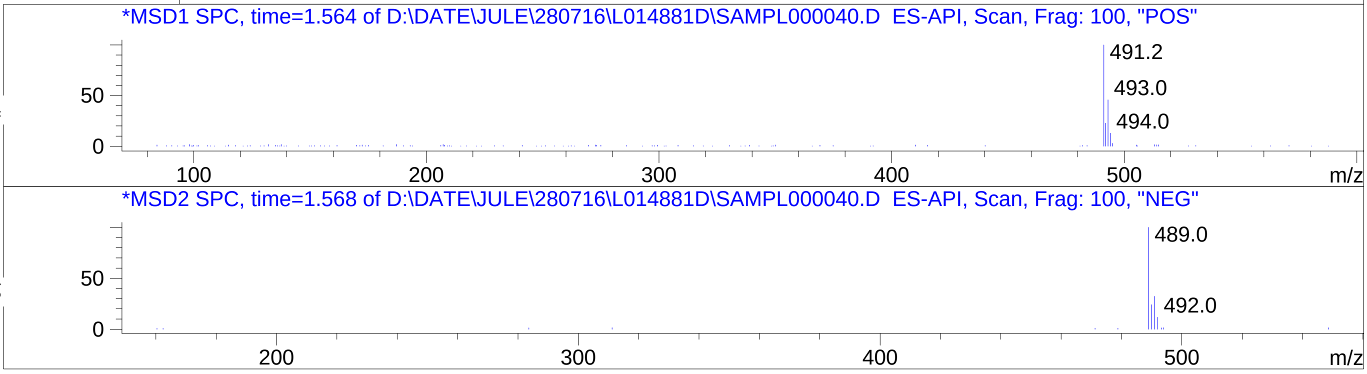

Inj.Date 7/28/2016 T P2-D-05 -12- Acq. Method C:\CHEM32\-> ->

MaxPeak: 90.67%
Ret_Time: 1.446 min

IBOX14129

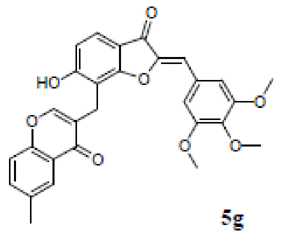

5g

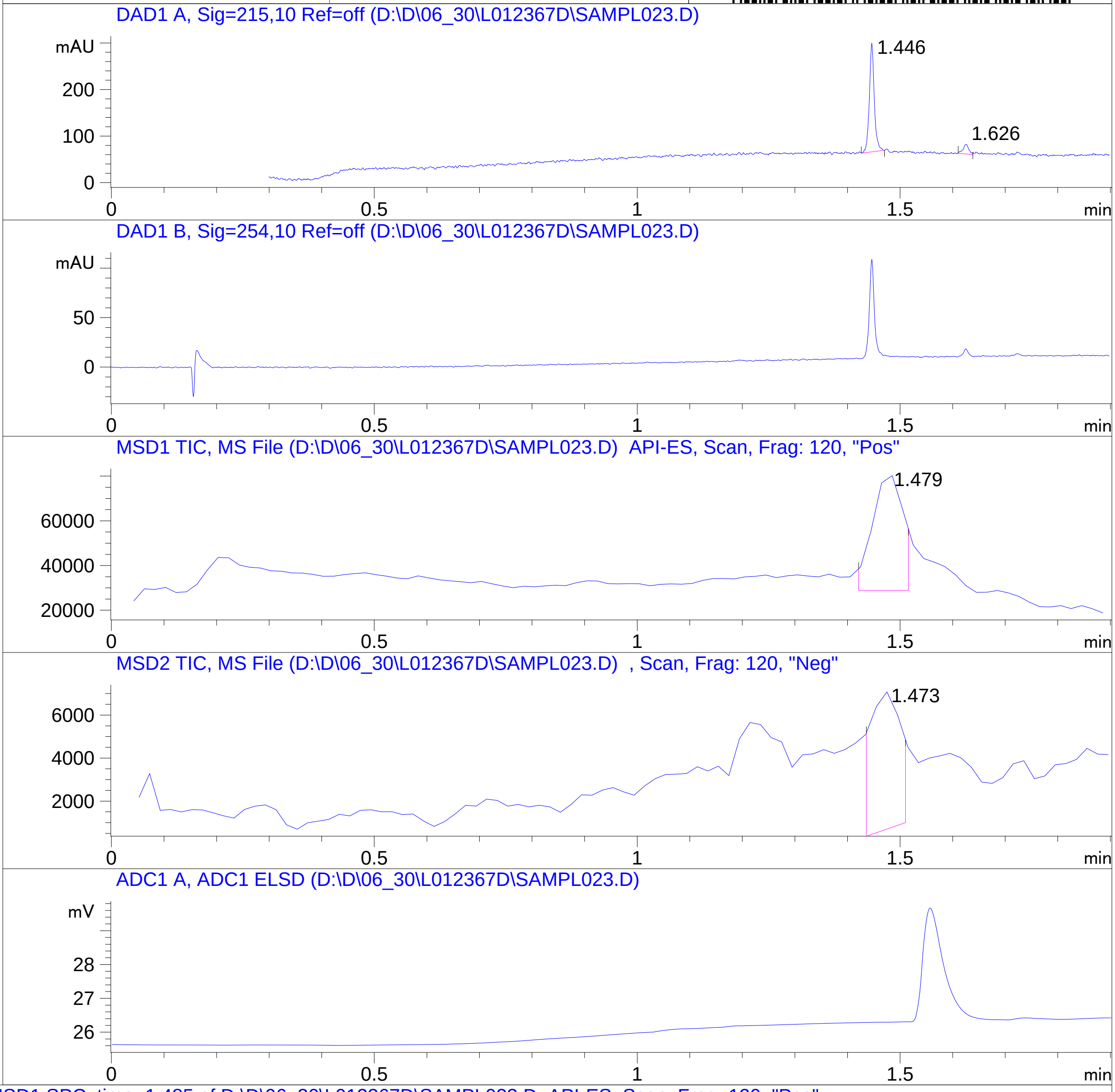

Mol Wt 0
Exact Mass

#	Time	Area%
1	1.446	90.67
2	1.626	9.33

RT 1.479

RT 1.473

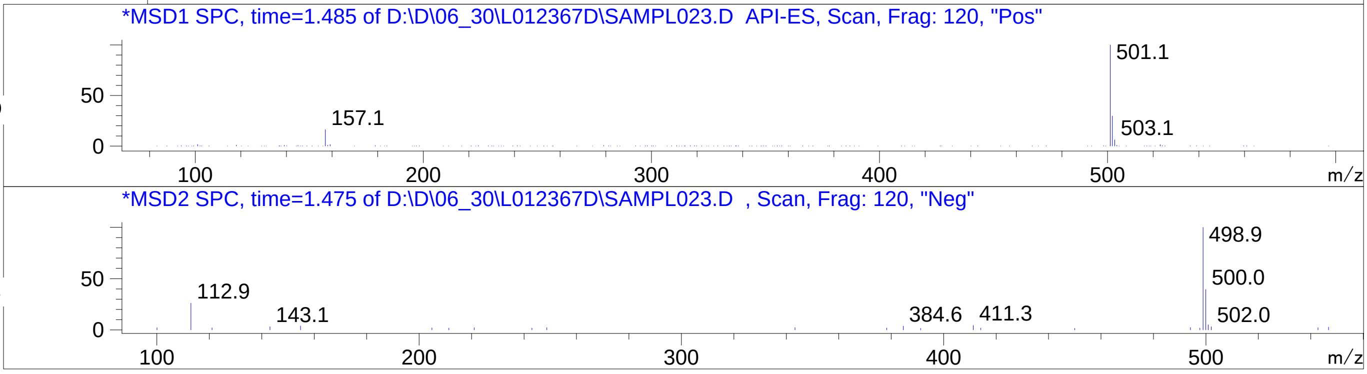

Inj.Date 6/30/2016 N P2-B-07 S87 VL Acq. Method C:\HPCHEM\-> ->

MaxPeak: 100.00%
Ret_Time: 1.397 min

IBOX22332

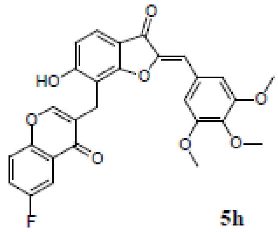

5h

Mol Wt 0

Exact Mass

#	Time	Area%
1	1.397	100.00

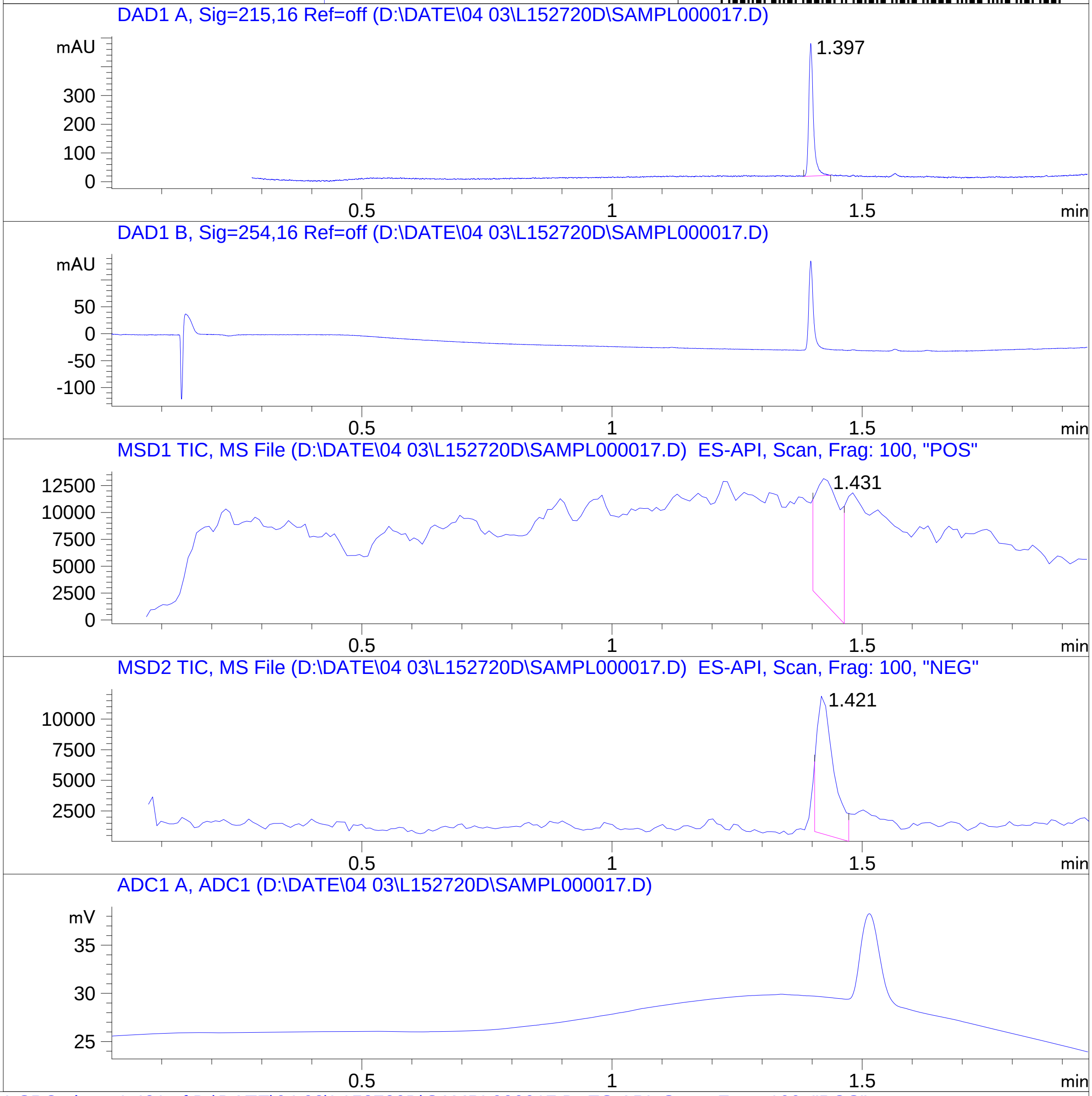

RT 1.431

RT 1.421

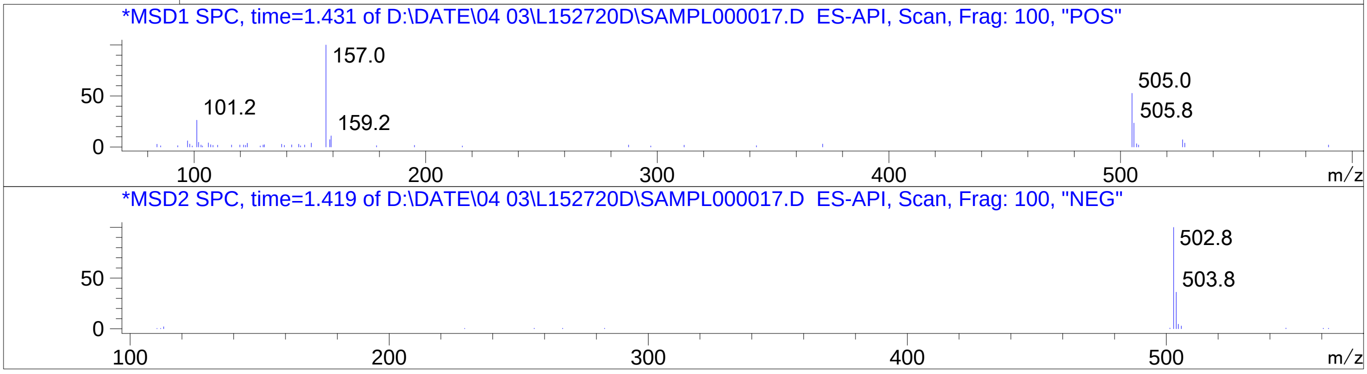

MaxPeak: 86.24%
Ret_Time: 1.574 min

IBOX14821

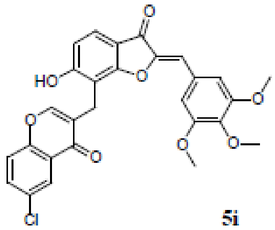

5i

Mol Wt 0

Exact Mass

#	Time	Area%
1	1.574	86.24
2	1.749	11.20
3	1.886	2.55

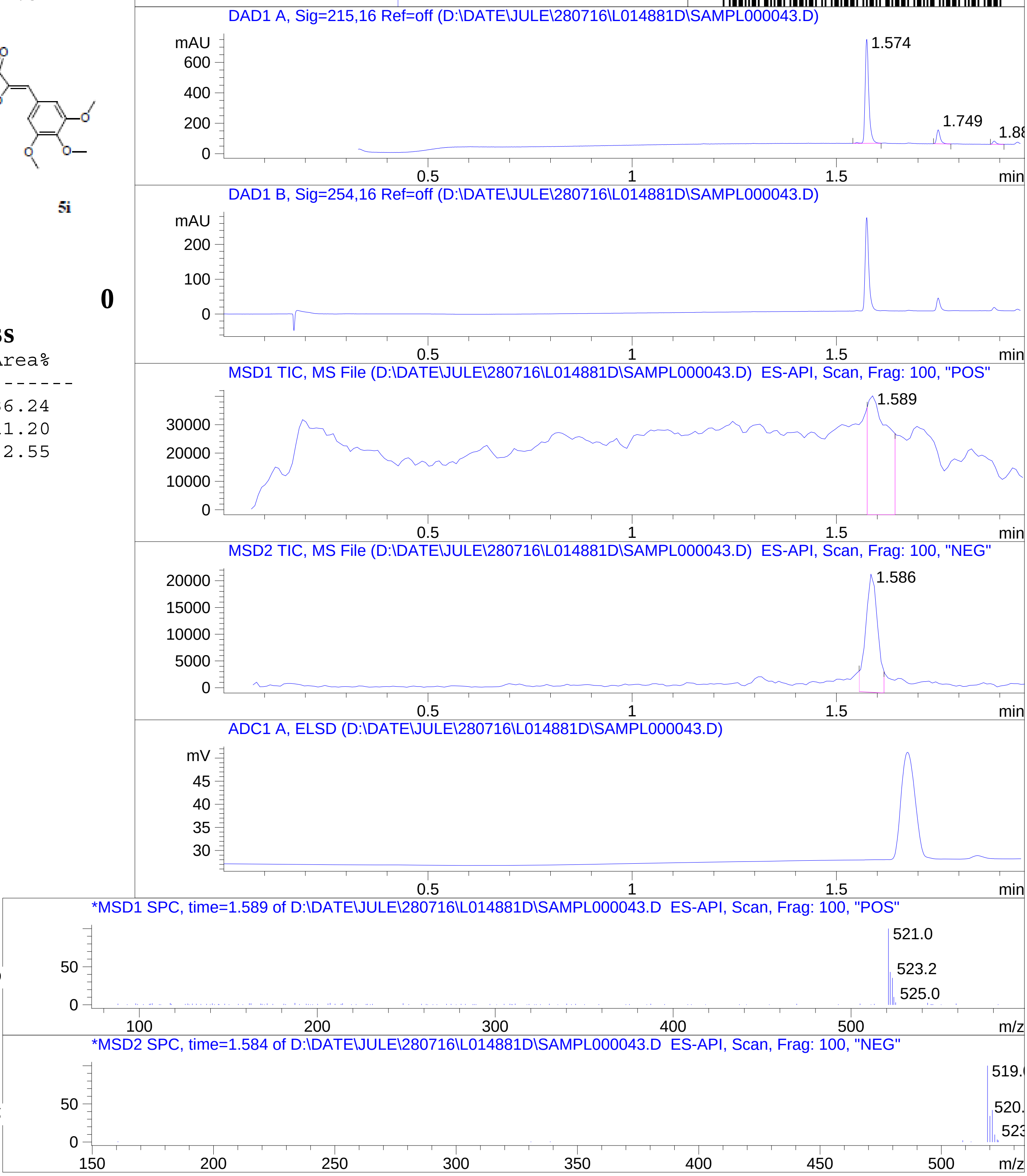

RT 1.589

RT 1.586

Inj.Date 7/28/2016 T P2-D-08 -12- Acq. Method C:\CHEM32\-> ->

MaxPeak: 100.00%
Ret_Time: 1.252 min

IBOX14631

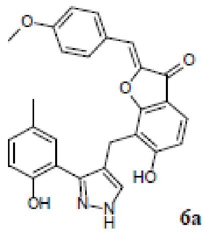

6a

Mol Wt 0

Exact Mass

#	Time	Area%
1	1.252	100.00

RT 1.268

RT 1.271

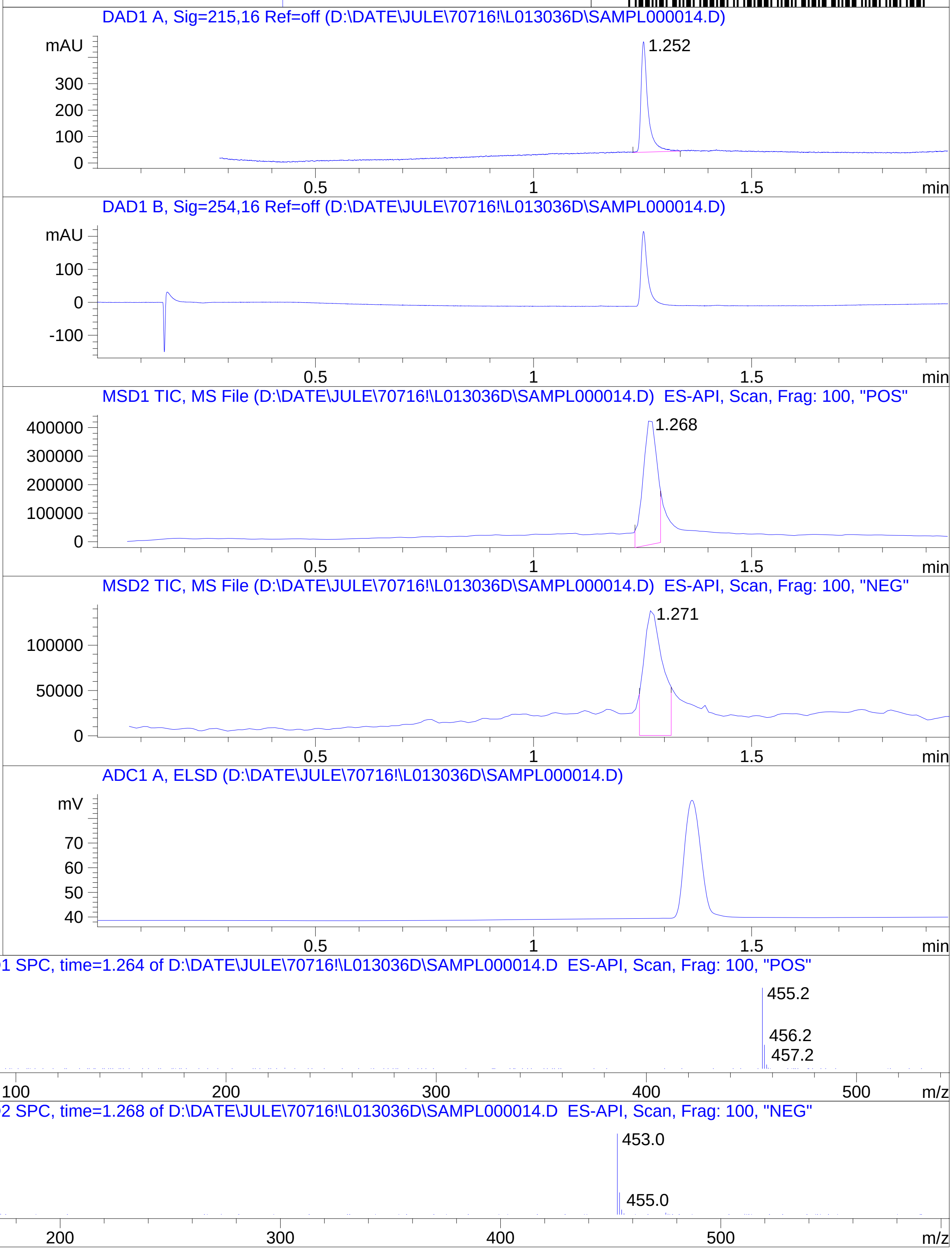

MaxPeak: 96.14%
Ret_Time: 1.333 min

IBOX14938

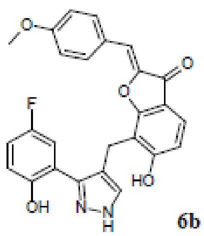

6b

Mol Wt **0**

Exact Mass

#	Time	Area%
1	1.333	96.14
2	1.431	2.58
3	1.548	1.28

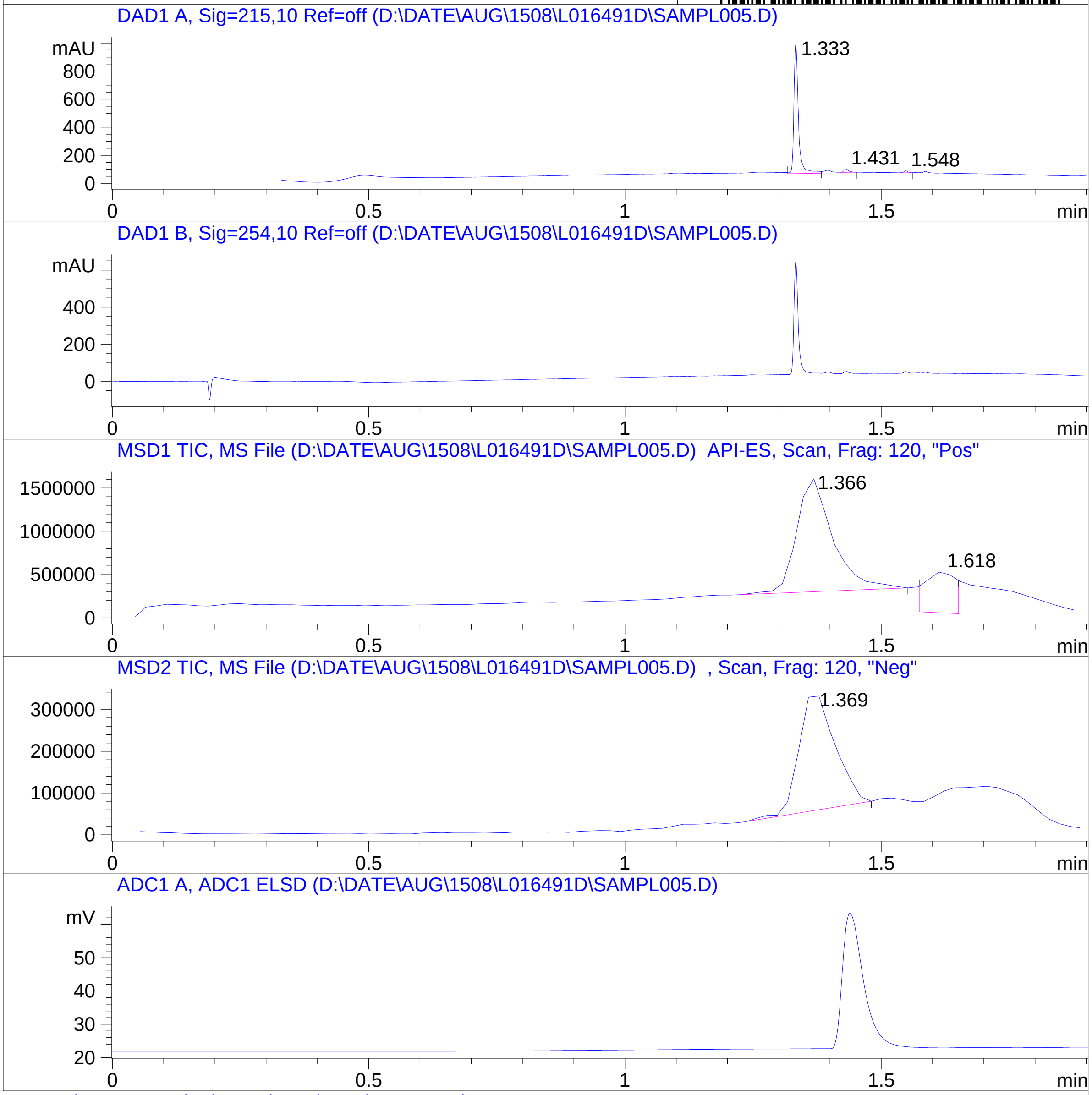

RT 1.366

RT 1.618

RT 1.369

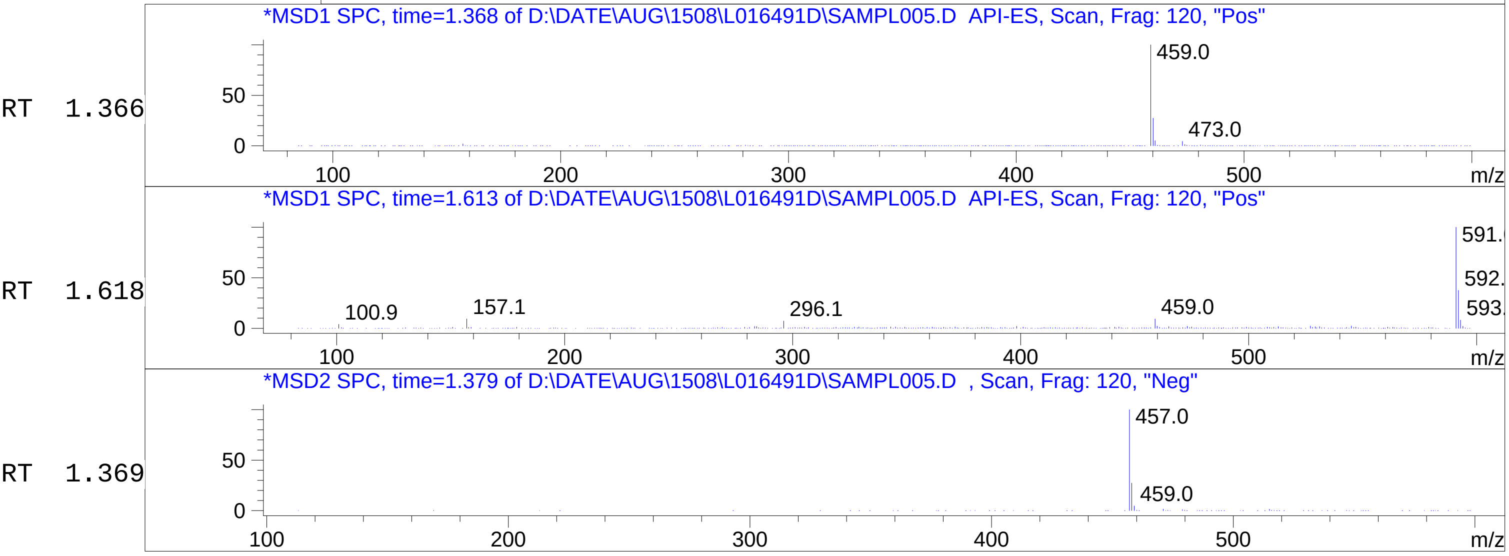

Inj.Date 8/16/2016 A P2-A-05 -SL- Acq. Method C:\HPCHEM\-> ->

MaxPeak: 100.00%
Ret_Time: 1.361 min

IBOX14102

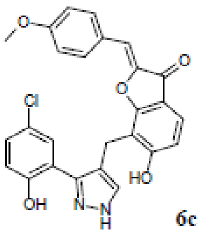

6c

Mol Wt 0
Exact Mass

#	Time	Area%
1	1.361	100.00

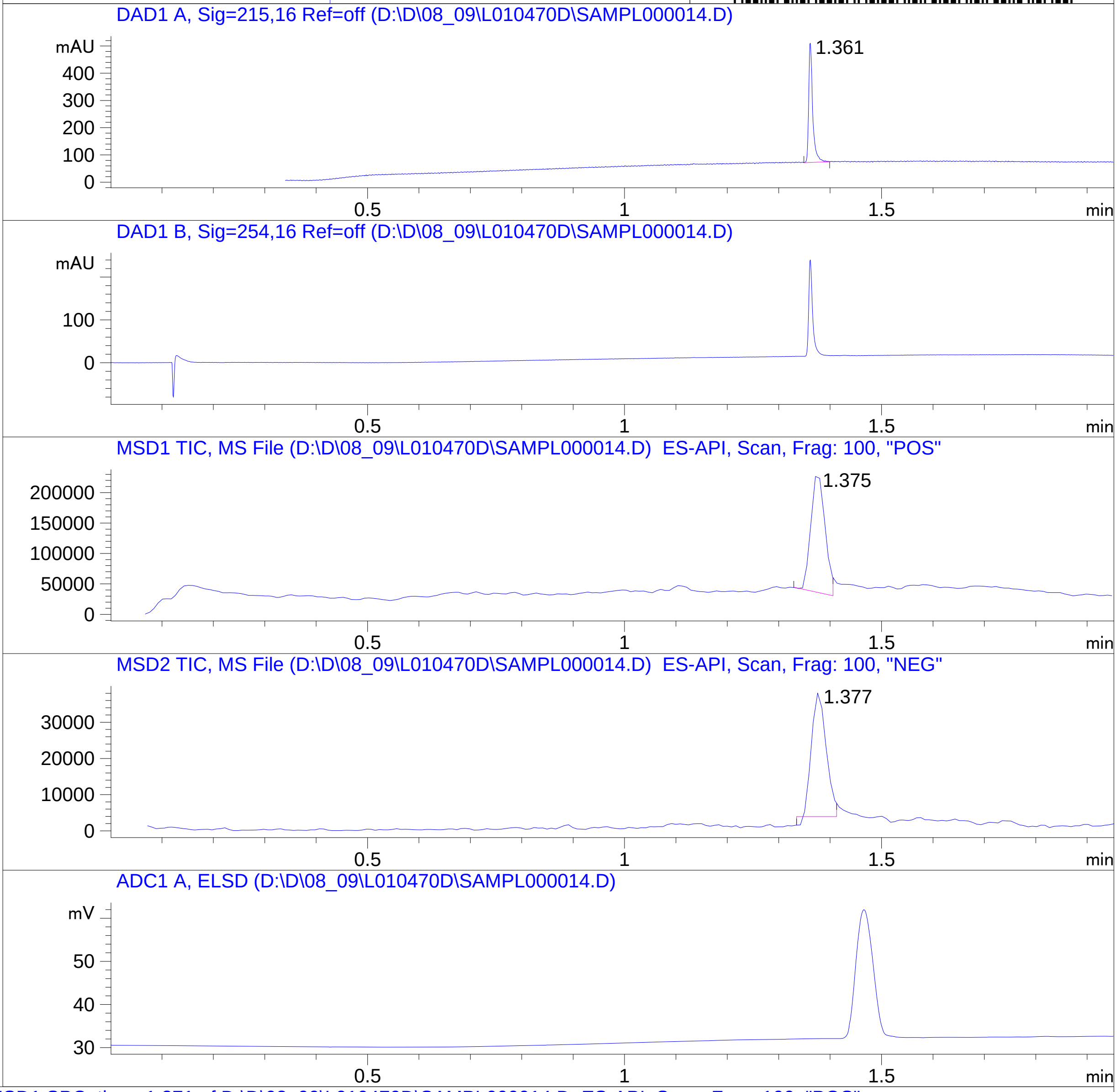

RT 1.375

RT 1.377

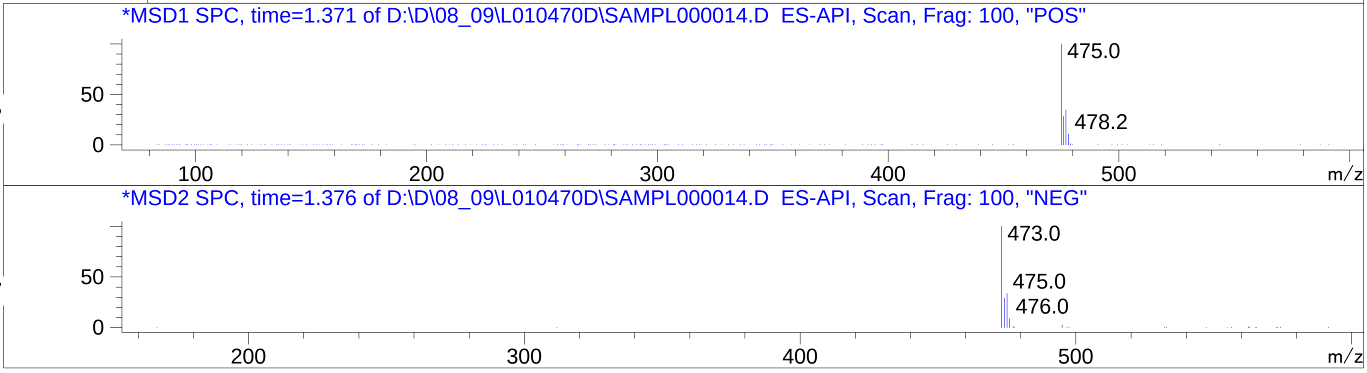

MaxPeak: 94.98%
Ret_Time: 1.254 min

IBOX14119

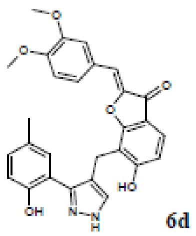

6d

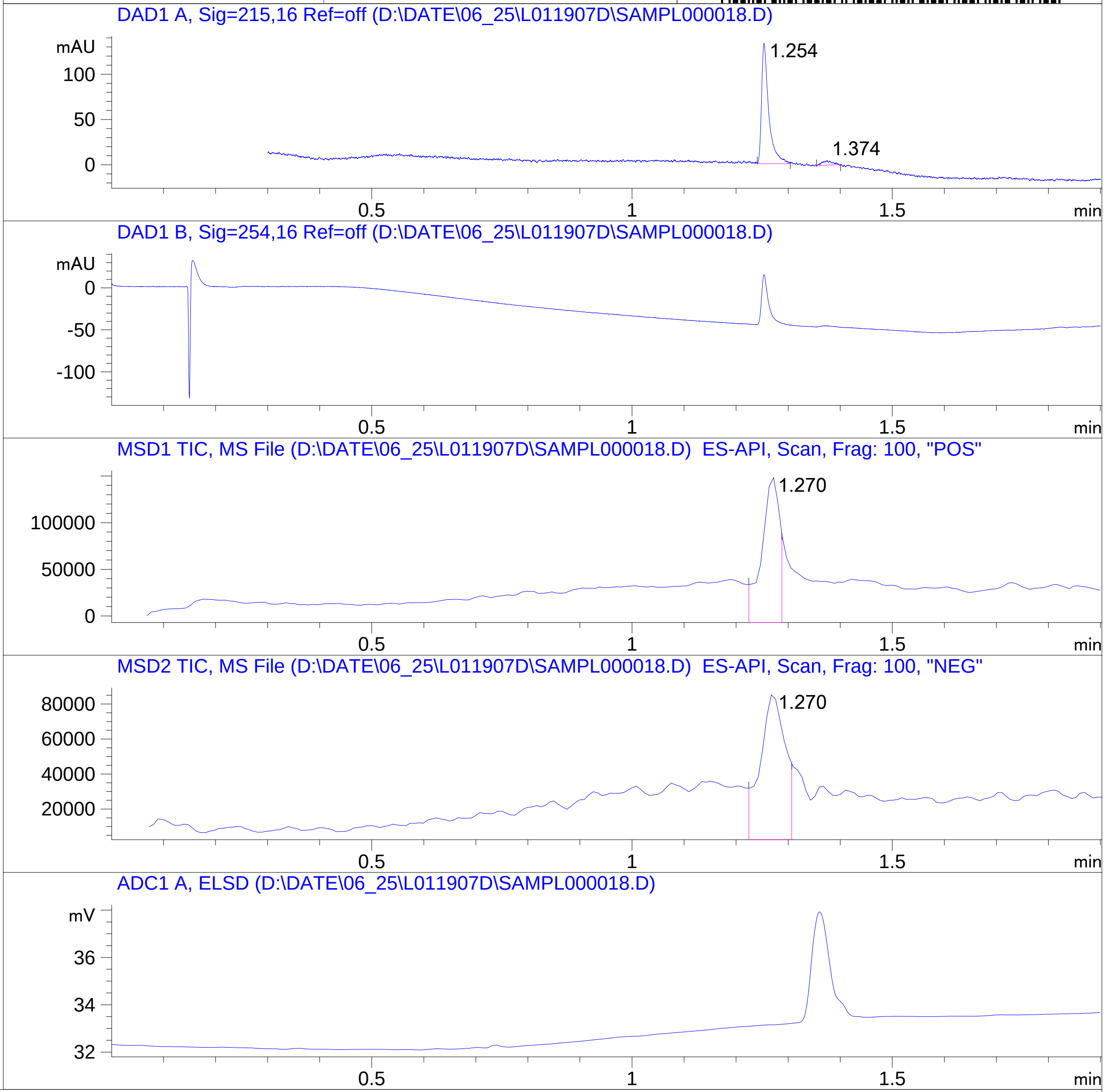

Mol Wt 0

Exact Mass

#	Time	Area%
1	1.254	94.98
2	1.374	5.02

RT 1.270

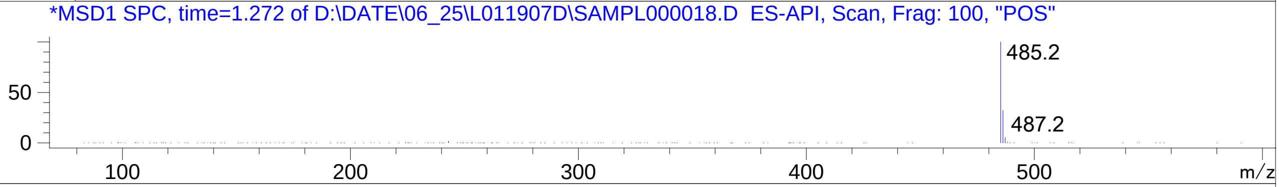

RT 1.270

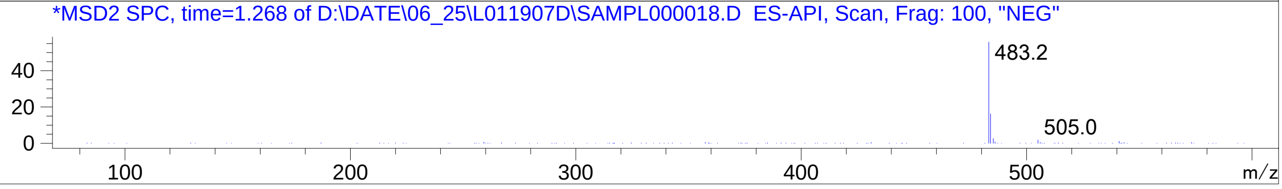

Inj.Date 6/24/2016 E P2-B-09 S93 -5- Acq. Method C:\CHEM32\-> ->

MaxPeak: 100.00%
Ret_Time: 1.240 min

IBOX14630

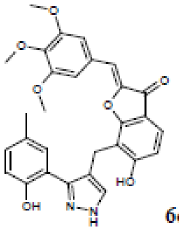

6e

Mol Wt 0

Exact Mass

#	Time	Area%
1	1.240	100.00

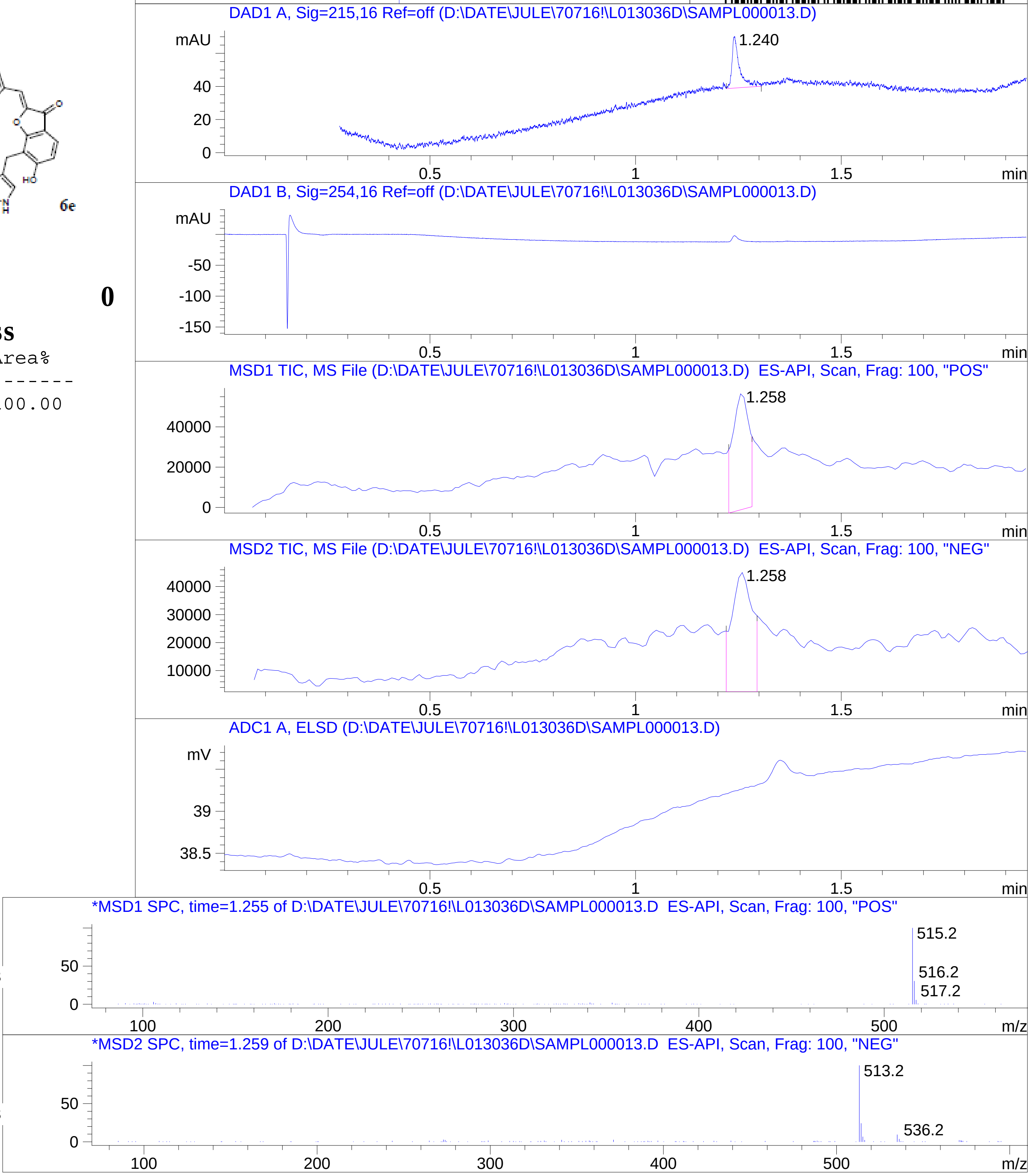

MaxPeak: 100.00%
Ret_Time: 1.320 min

IBOX14678

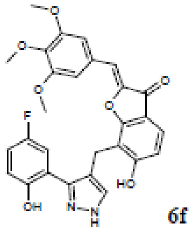

6f

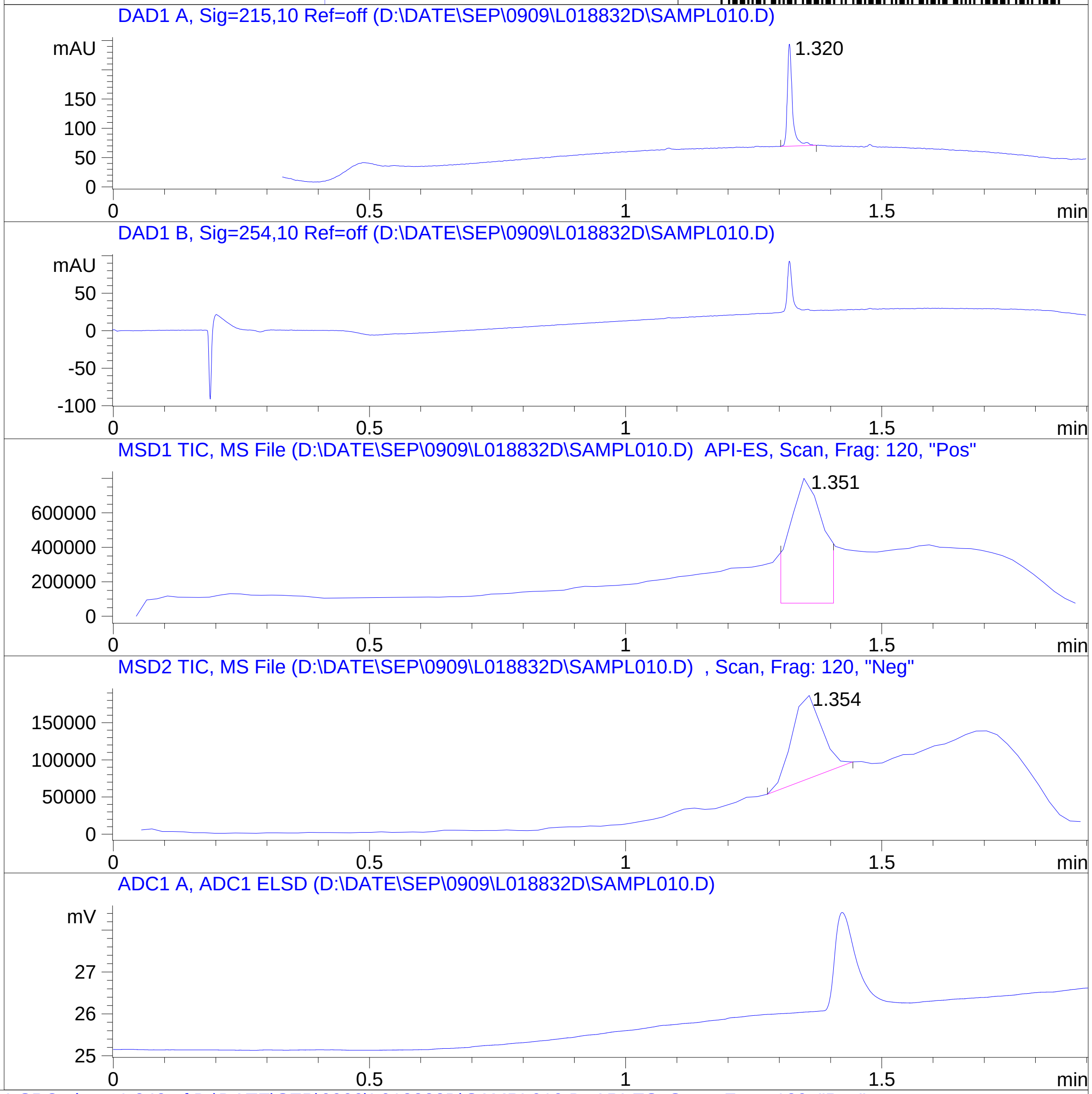

Mol Wt 0

Exact Mass

#	Time	Area%
1	1.320	100.00

RT 1.351

RT 1.354

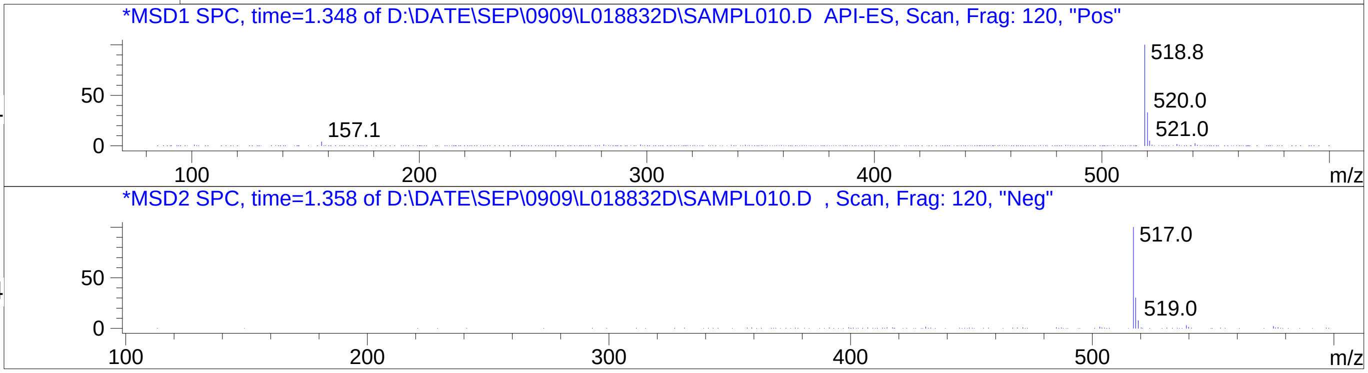

Inj.Date 9/10/2016 A P2-A-09 -SL- Acq. Method C:\HPCHEM\-> ->

MaxPeak: 100.00%
Ret_Time: 1.402 min

IBOX14949

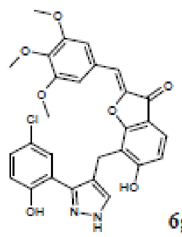

6g

Mol Wt 0

Exact Mass

```
#  Time    Area%
------------------
1  1.402   100.00
```

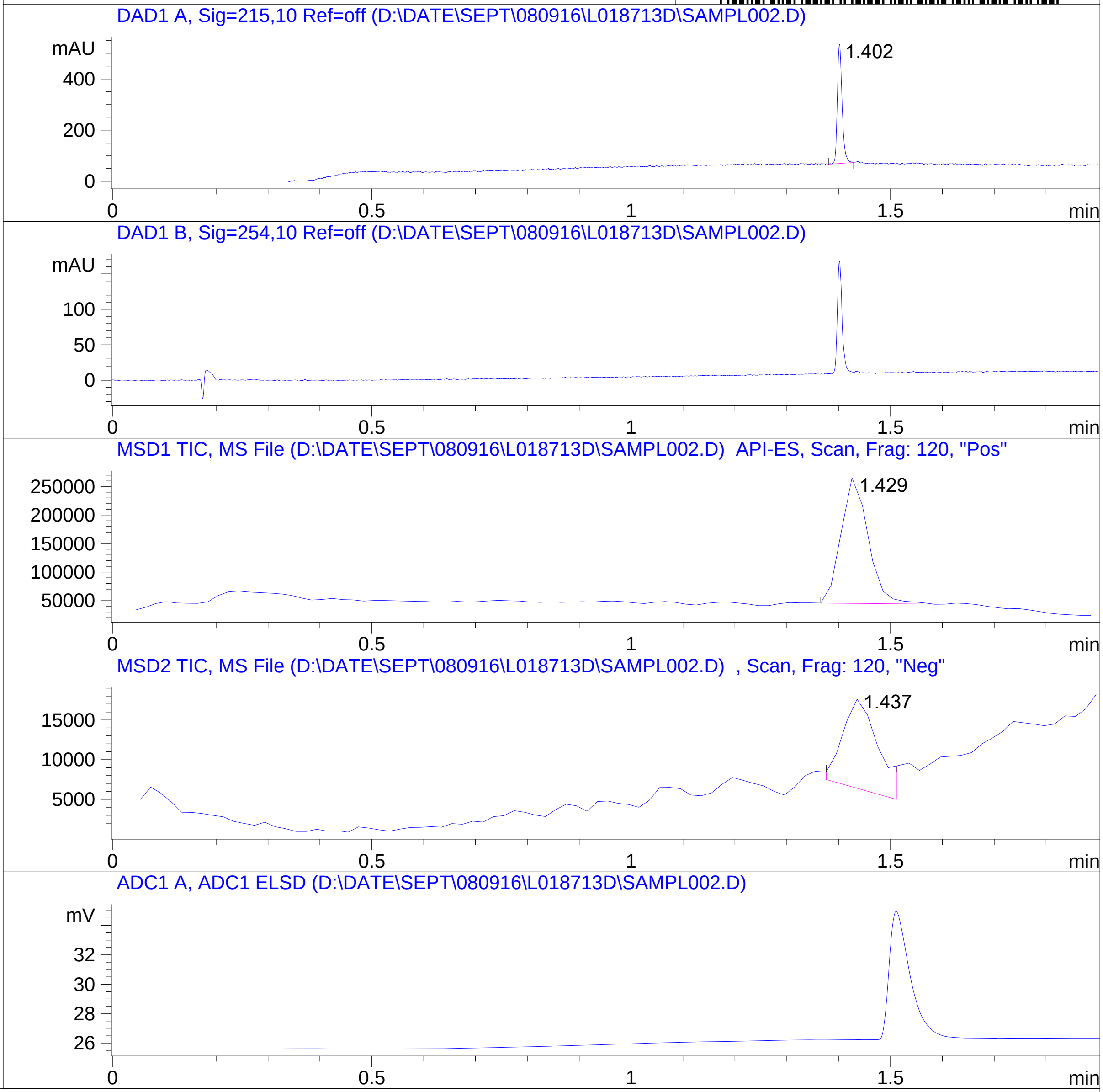

RT 1.429

RT 1.437

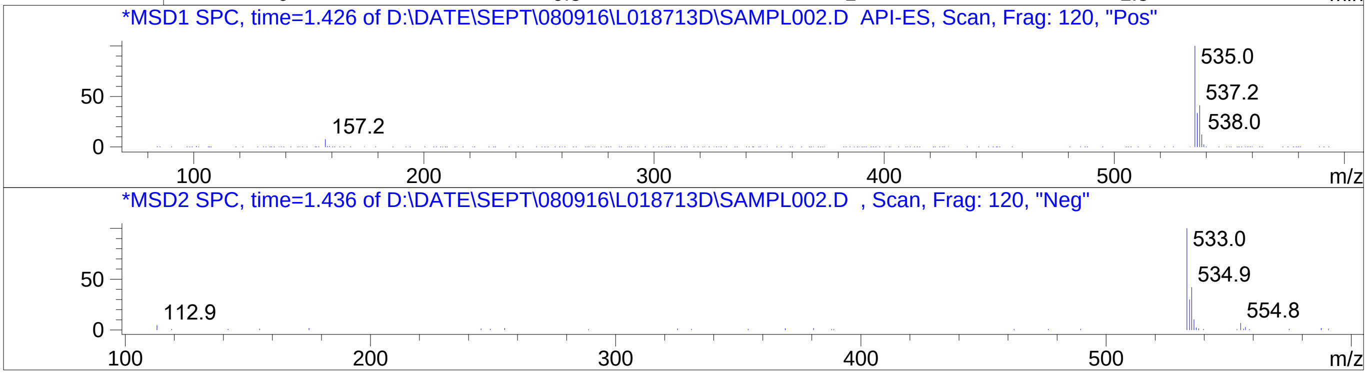

Inj.Date 9/9/2016 Y P2-A-02 S96 VL Acq. Method C:\HPCHEM\-> ->

MaxPeak: 100.00%
Ret_Time: 1.498 min

IBOX23657

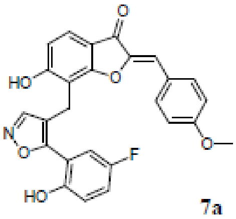

7a

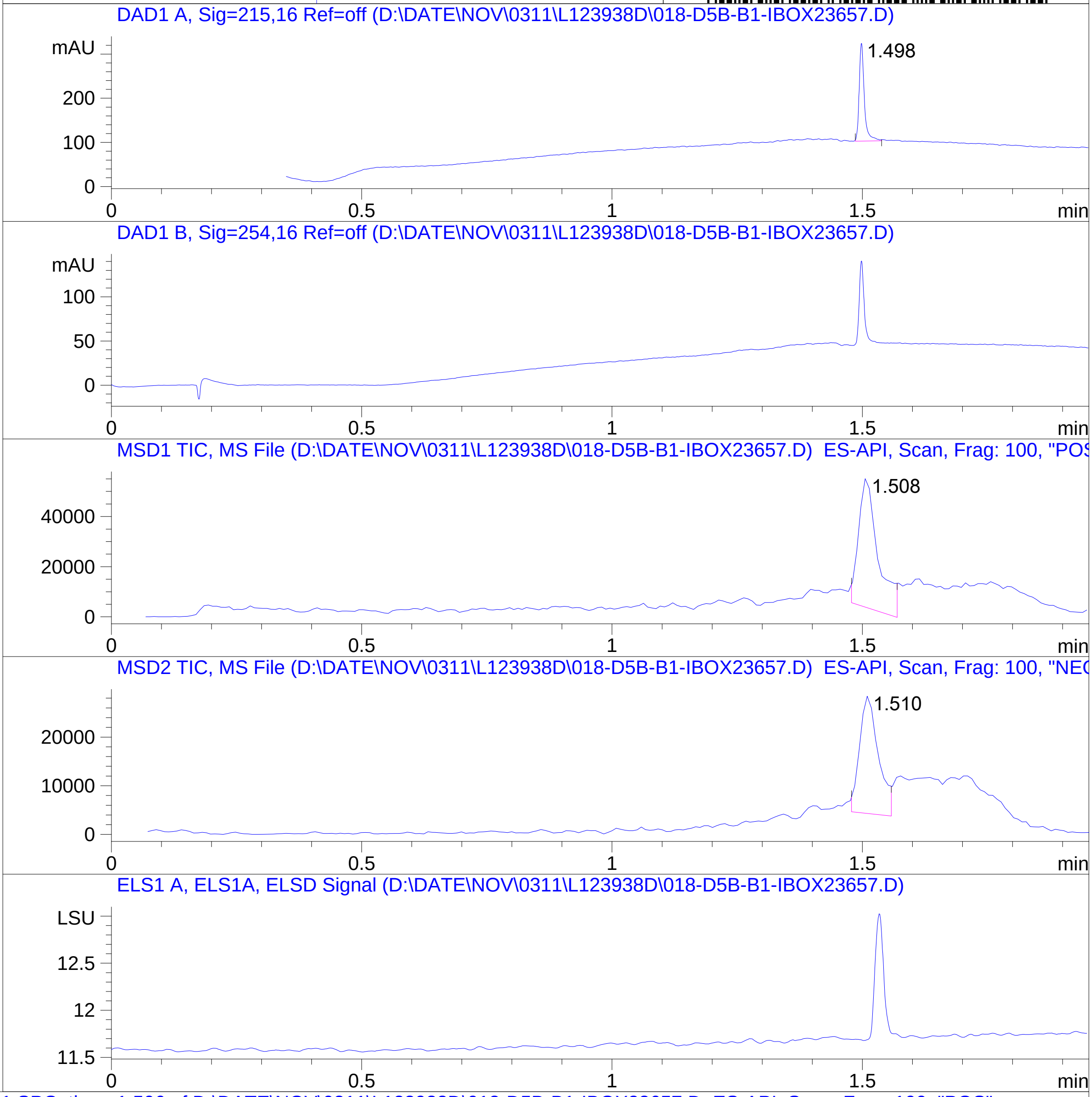

Mol Wt 0

Exact Mass

#	Time	Area%
1	1.498	100.00

RT 1.508

RT 1.510

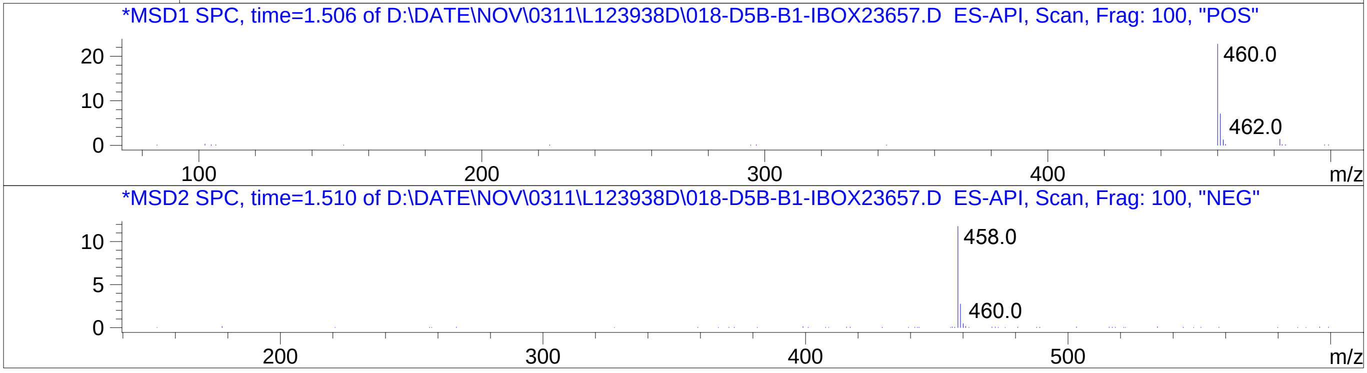

Inj.Date 11/5/2018 Y <invalid>-15- Acq. Method C:\Chem32\-> ->

MaxPeak: 90.77%
Ret_Time: 1.517 min

IBOX23659

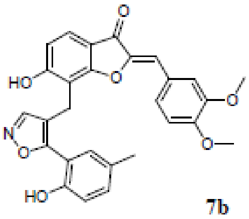

7b

Mol Wt 0

Exact Mass

#	Time	Area%
1	1.517	90.77
2	1.543	9.23

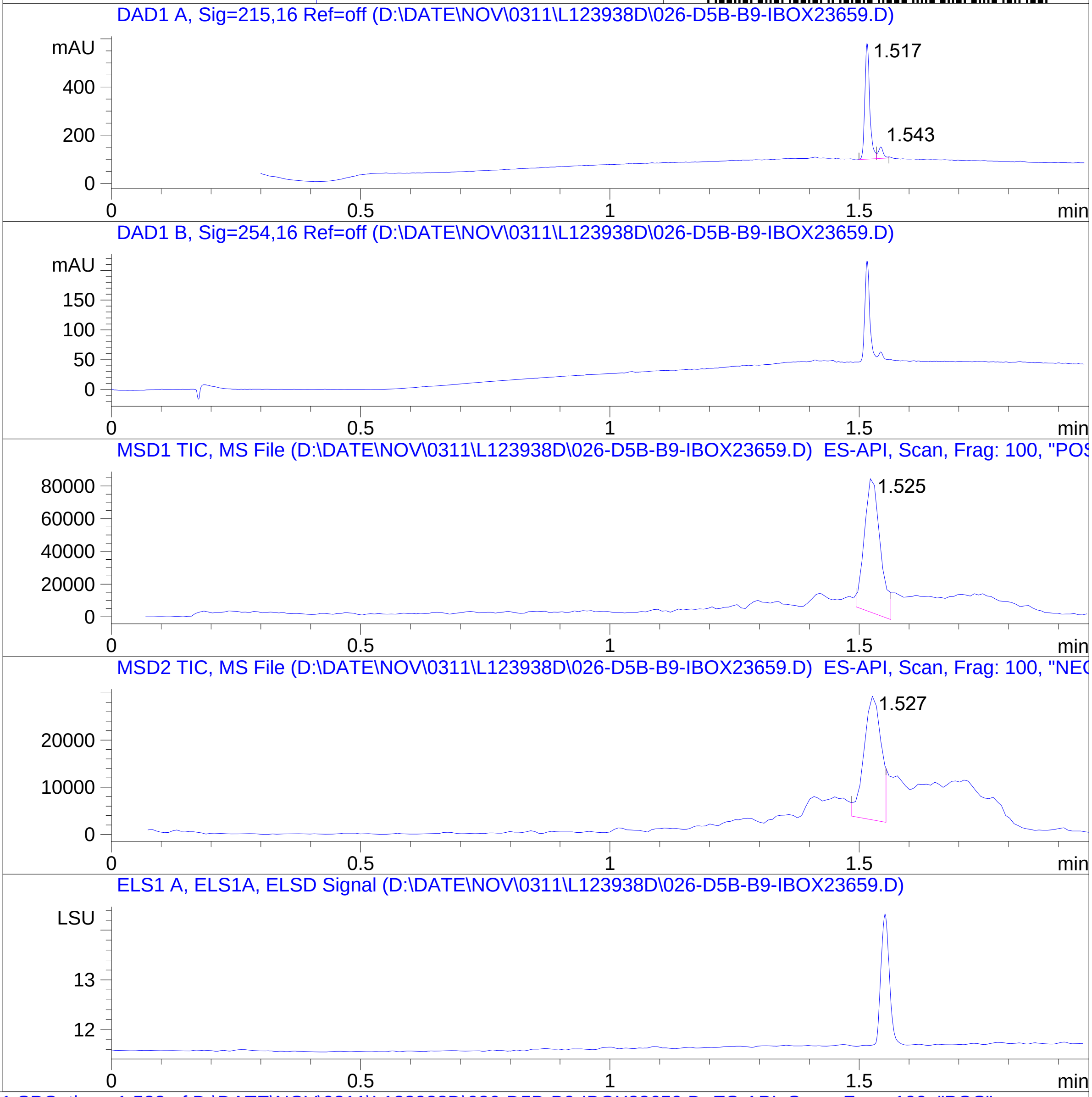

RT 1.525

RT 1.527

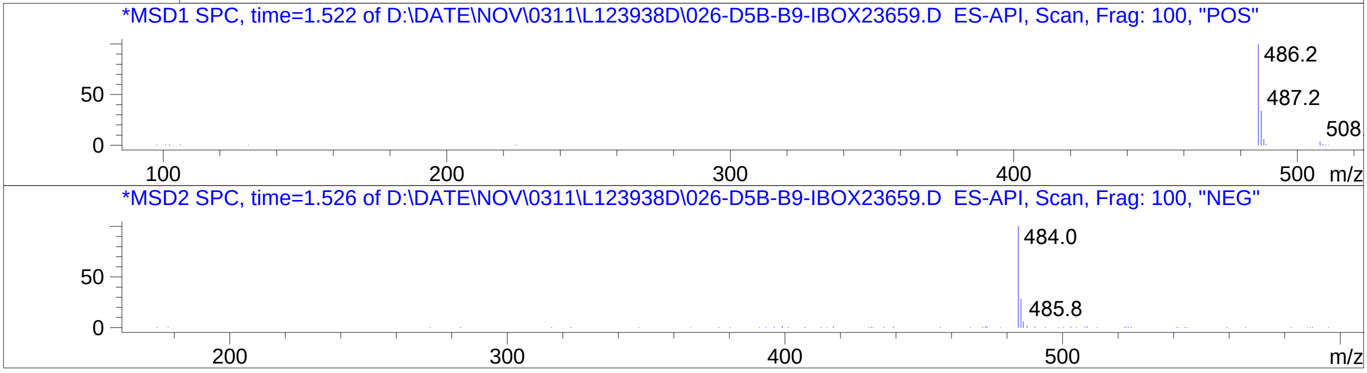

Inj.Date 11/5/2018 Y <invalid>-15- Acq. Method C:\Chem32\-> ->

MaxPeak: 97.75%
Ret_Time: 1.425 min

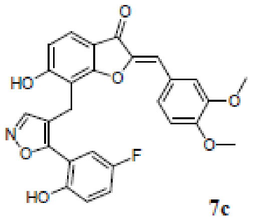

7c

IBOX23655

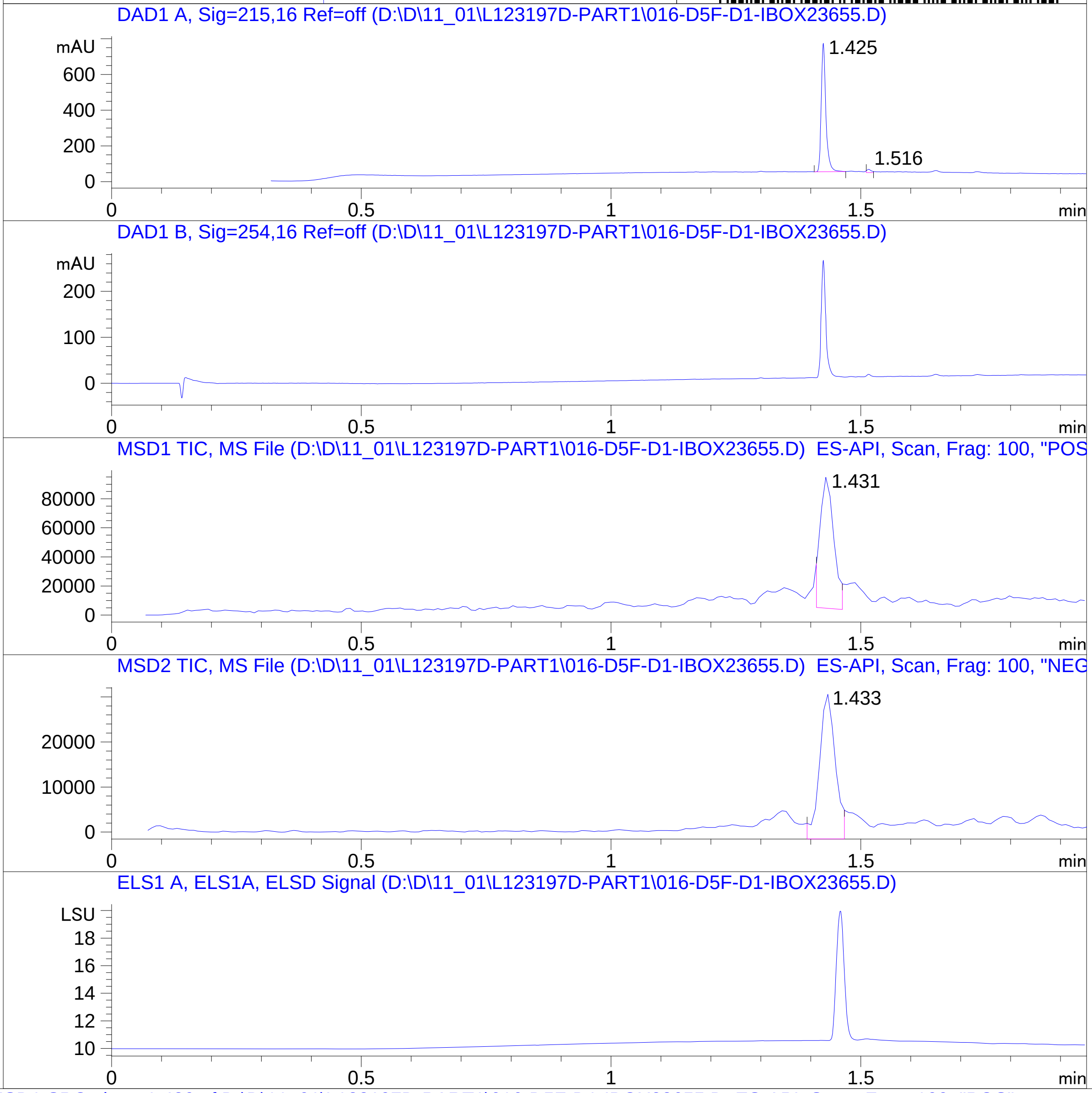

Mol Wt 0

Exact Mass

#	Time	Area%
1	1.425	97.75
2	1.516	2.25

RT 1.431

RT 1.433

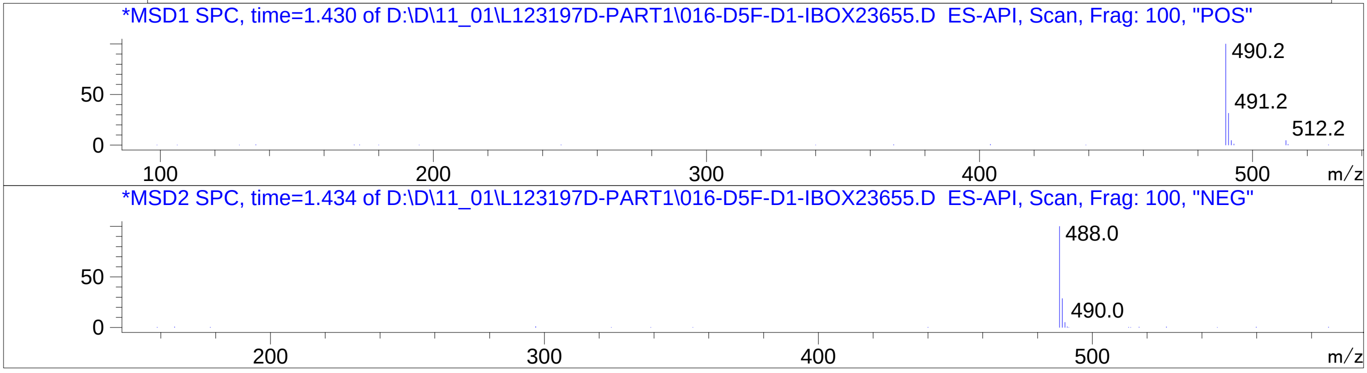

MaxPeak: 97.09%
Ret_Time: 1.529 min

IBOX23654

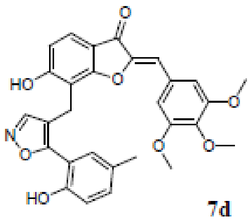

7d

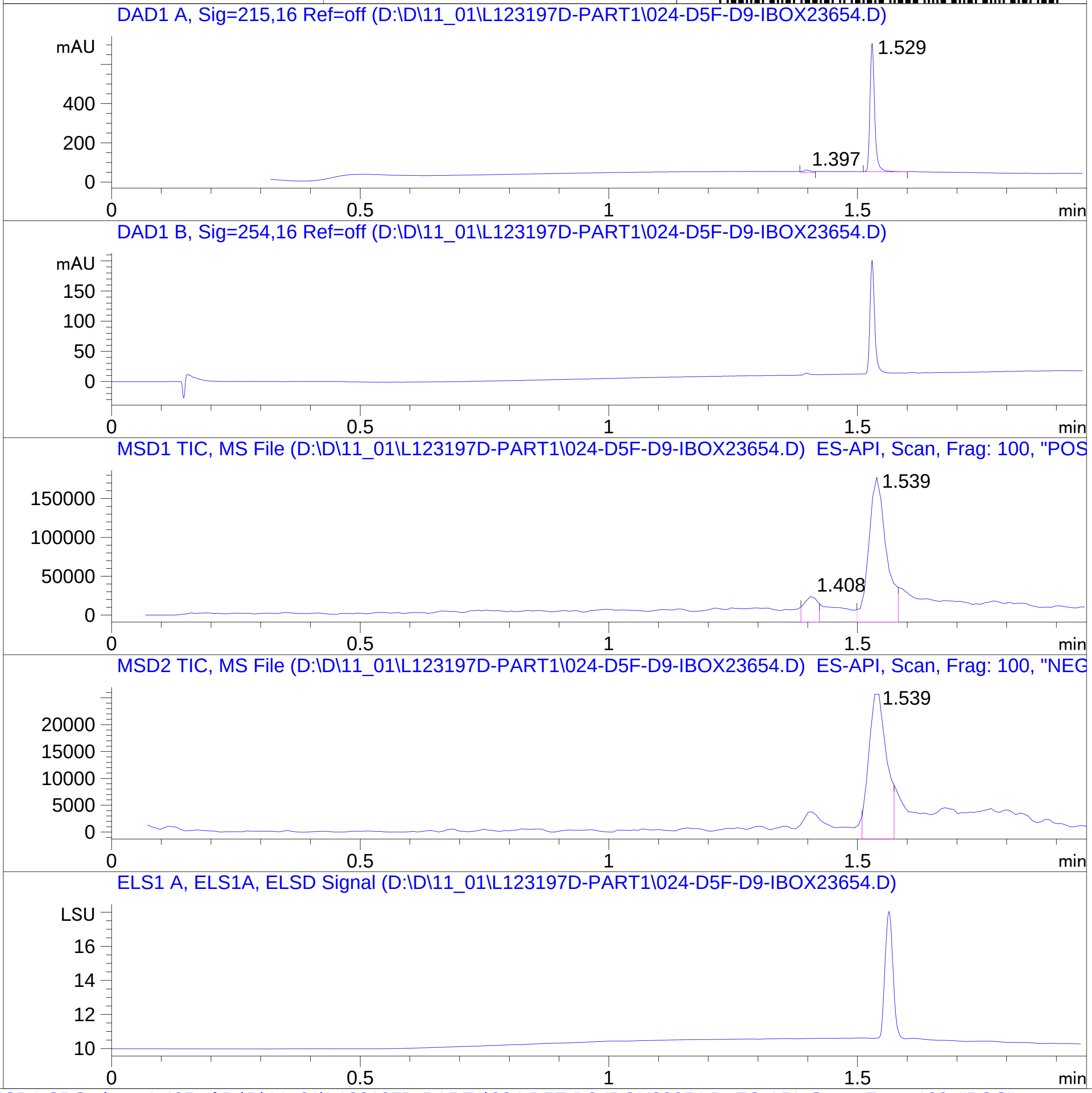

Mol Wt 0

Exact Mass

#	Time	Area%
1	1.397	2.91
2	1.529	97.09

RT 1.408

RT 1.539

RT 1.539

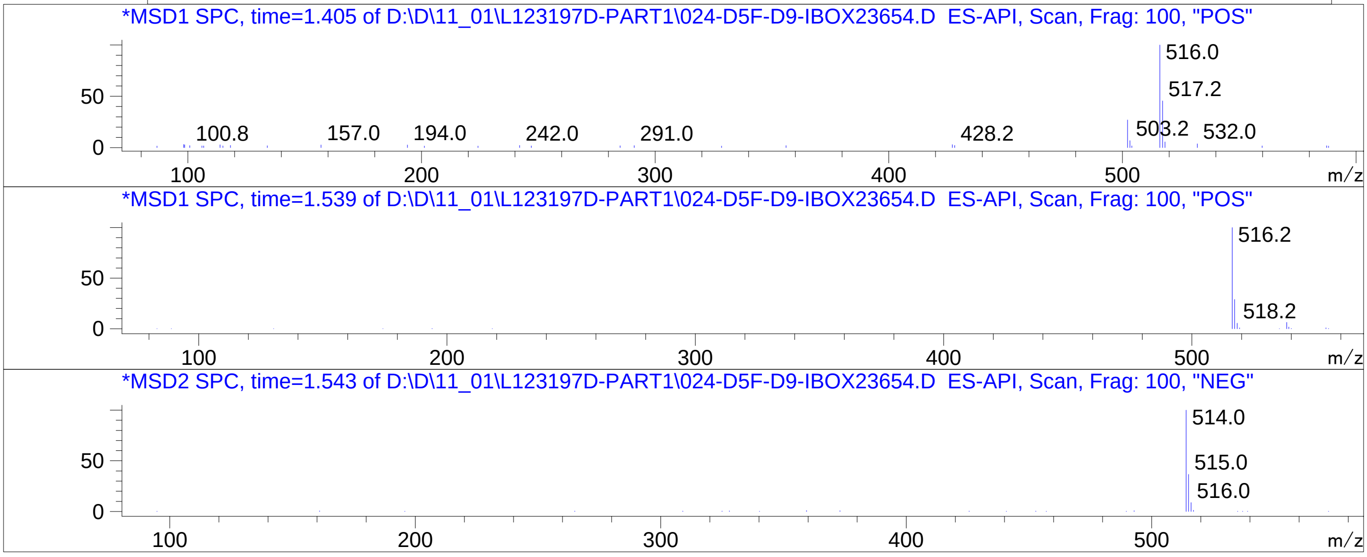

Inj.Date 10/31/2018 N <invalid> -17- Acq. Method C:\Chem32\-> ->

MaxPeak: 96.35%
Ret_Time: 1.526 min

IBOX23658

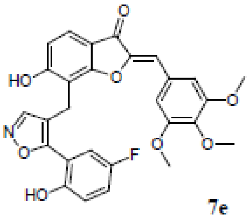

7e

Mol Wt 0

Exact Mass

#	Time	Area%
1	1.526	96.35
2	1.623	3.65

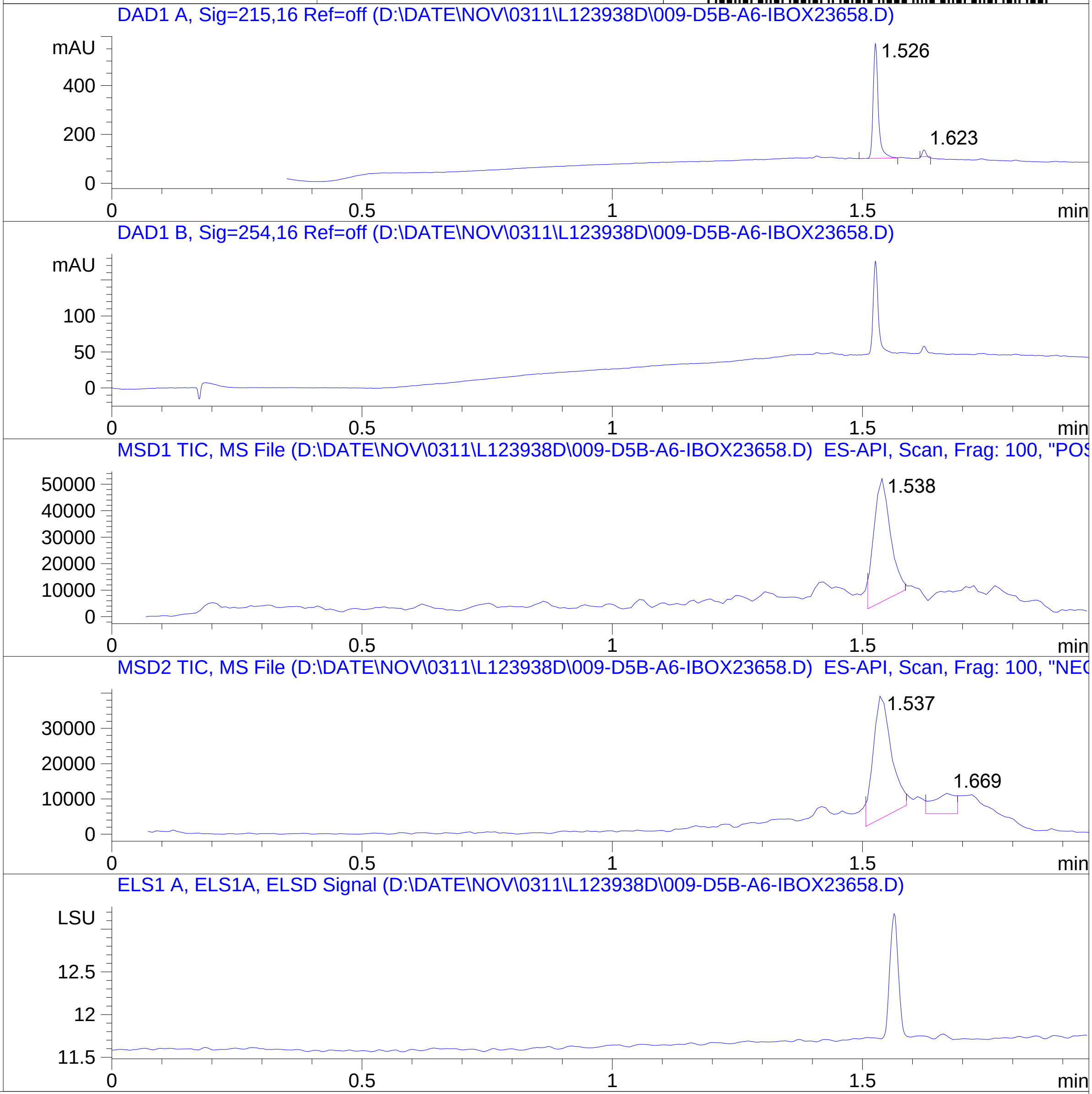

RT 1.538

RT 1.537

RT 1.669

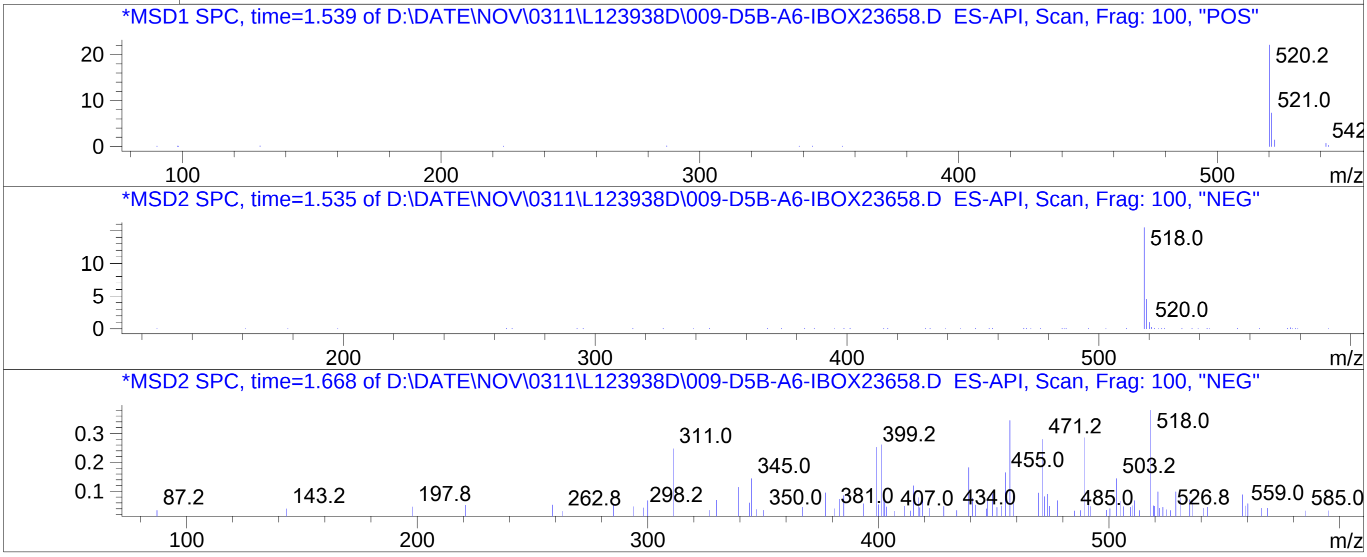

MaxPeak: 100.00%
Ret_Time: 1.567 min

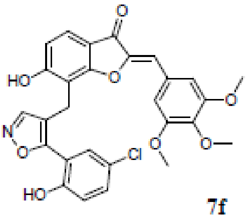

7f

Mol Wt 0

Exact Mass

#	Time	Area%
1	1.567	100.00

IBOX23652

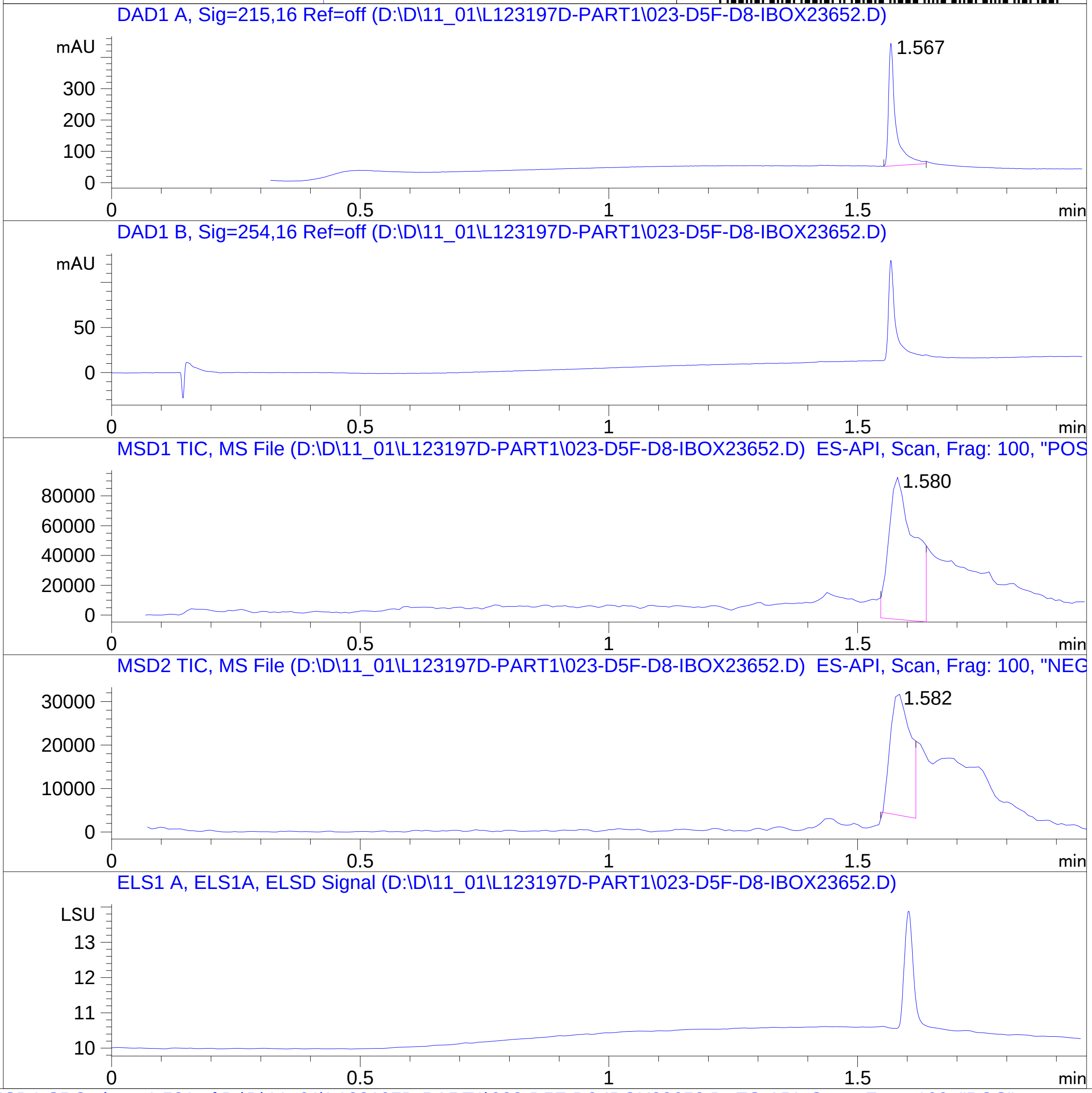

RT 1.580

RT 1.582

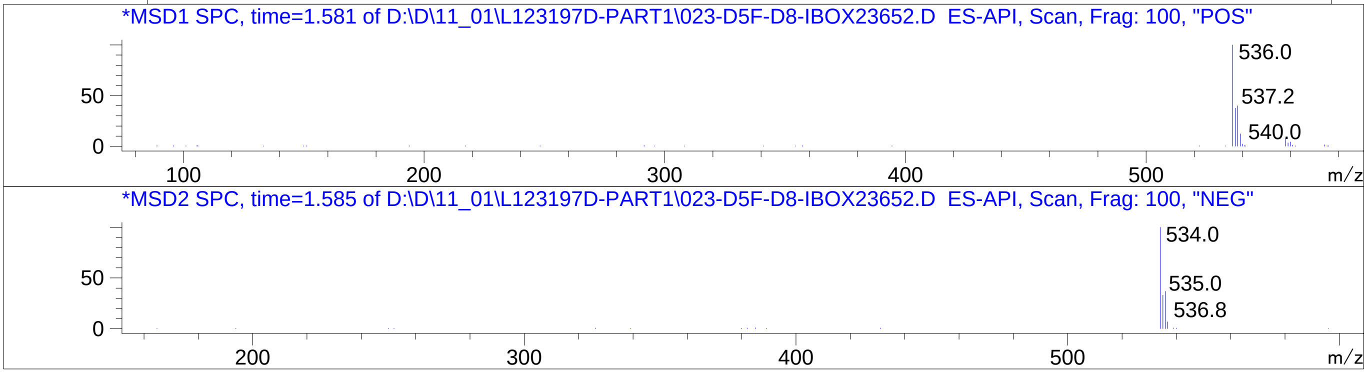

MaxPeak: 100.00%
Ret_Time: 1.267 min

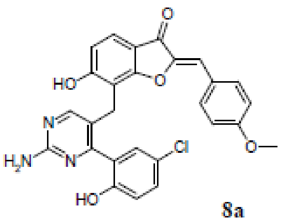

8a

IBOX23978

Mol Wt 0

Exact Mass

#	Time	Area%
1	1.267	100.00

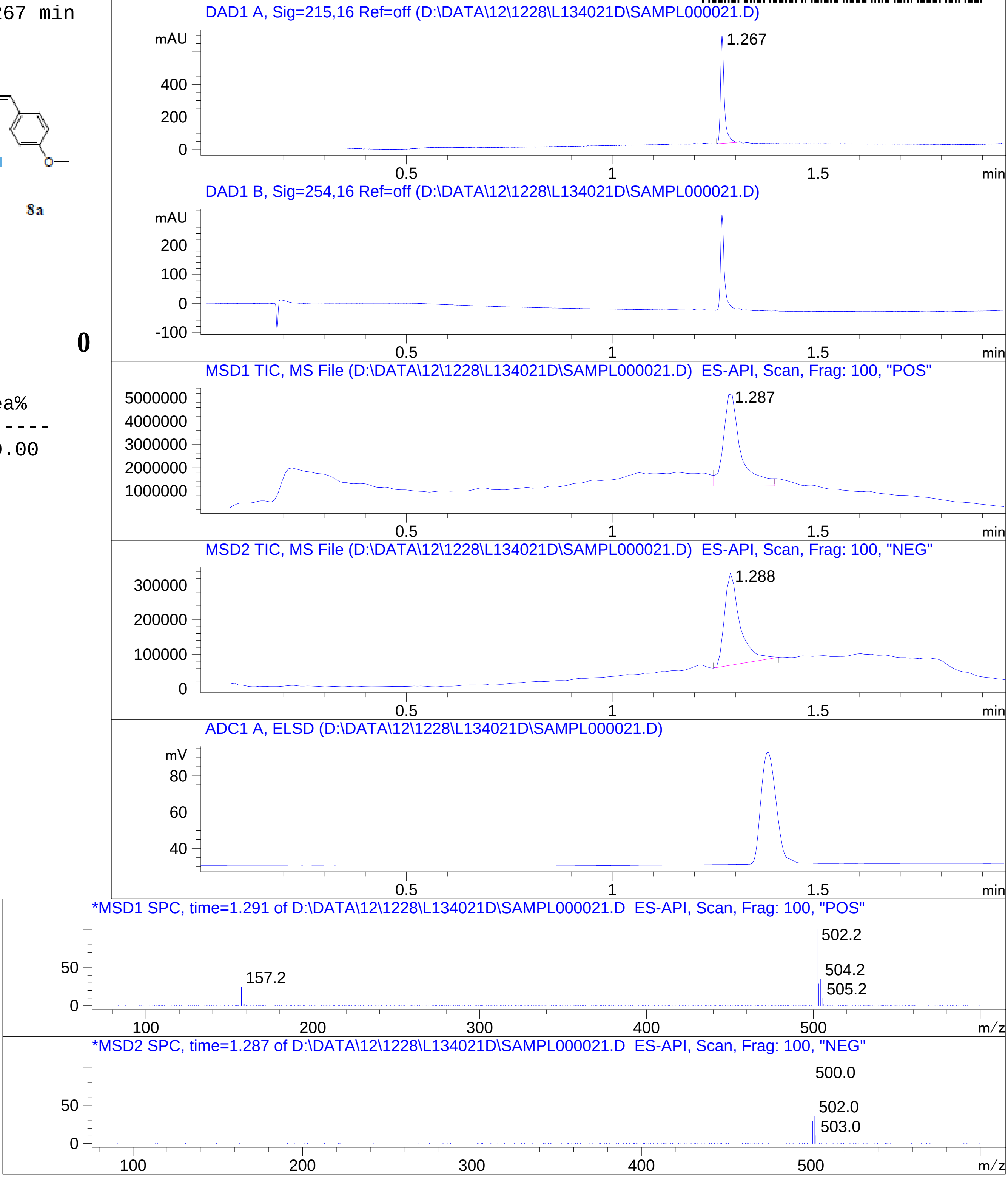

RT 1.287

RT 1.288

Inj.Date 12/28/2018 LB P2-B-06 -3- Acq. Method C:\CHEM32\-> ->

MaxPeak: 100.00%
Ret_Time: 1.111 min

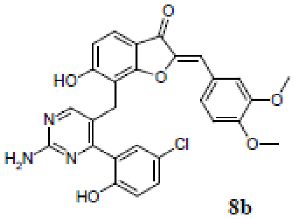

8b

Mol Wt 0

Exact Mass

#	Time	Area%
1	1.111	100.00

IBOX23979

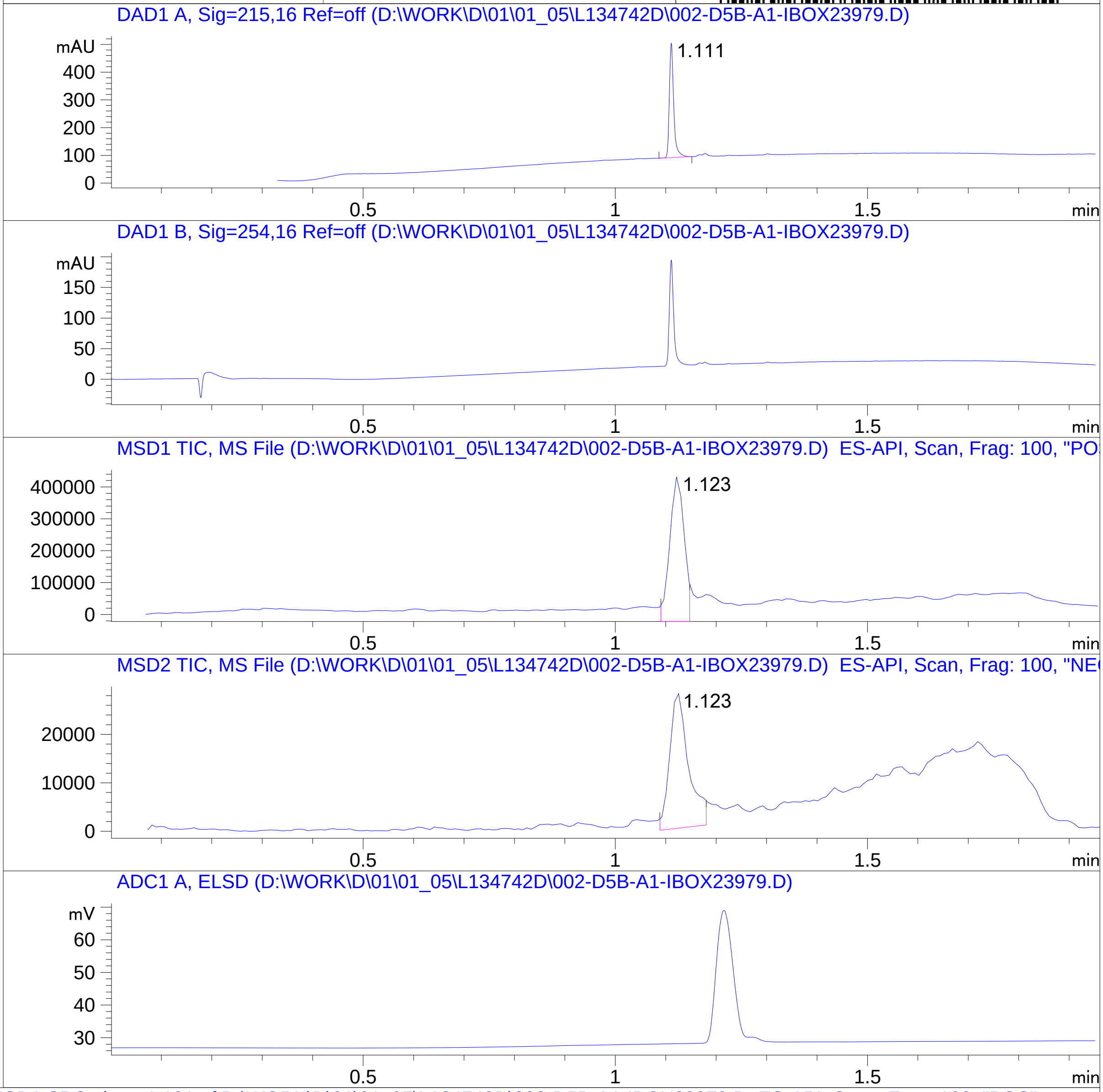

RT 1.123

RT 1.123

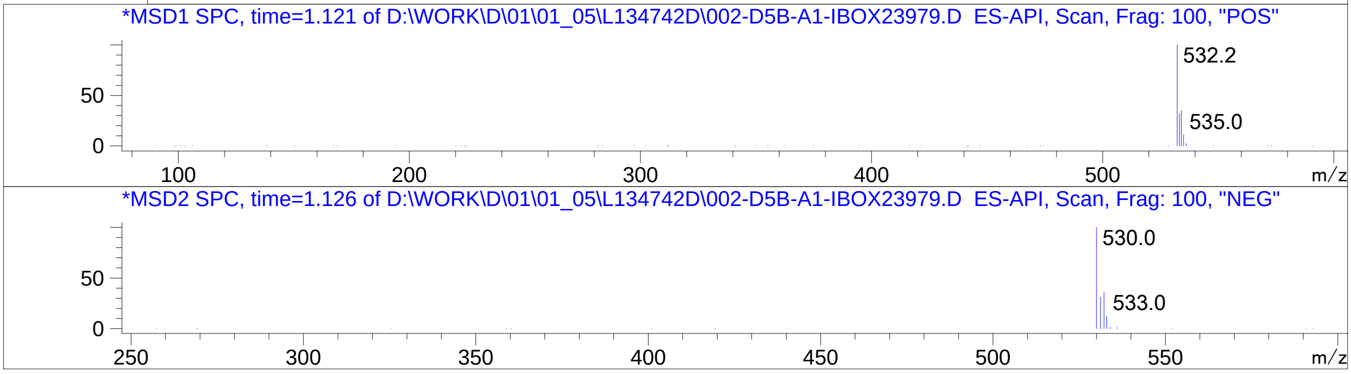

Inj.Date 1/4/2019 K <invalid> -> Acq. Method C:\Chem32\-> ->

MaxPeak: 100.00%
Ret_Time: 1.175 min

IBOX25758

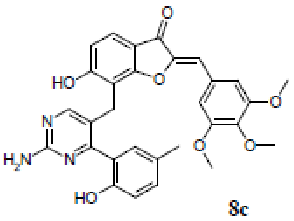

8c

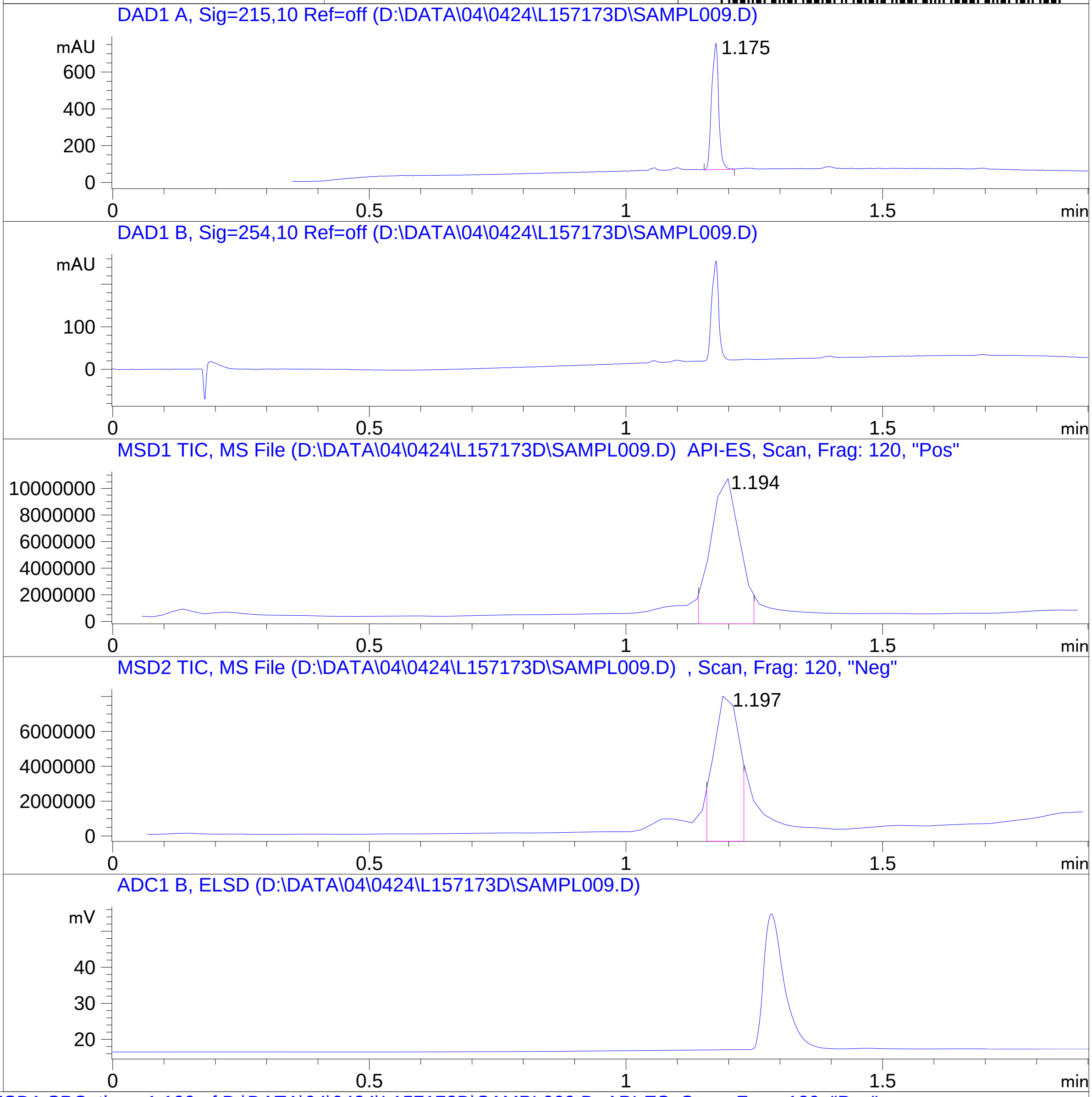

Mol Wt 0

Exact Mass

```
#  Time    Area%
------------------
1  1.175   100.00
```

RT 1.194

RT 1.197

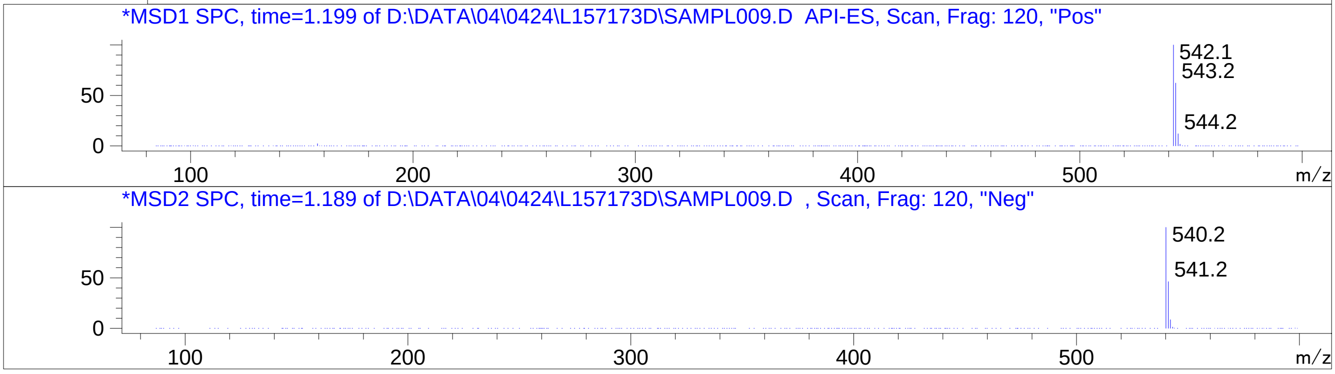

Inj.Date 4/24/2019 LB P2-A-09 S105 -SL- Acq. Method C:\HPCHEM\-> ->

MaxPeak: 100.00%
Ret_Time: 1.179 min

IBOX25759

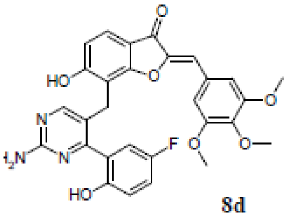

8d

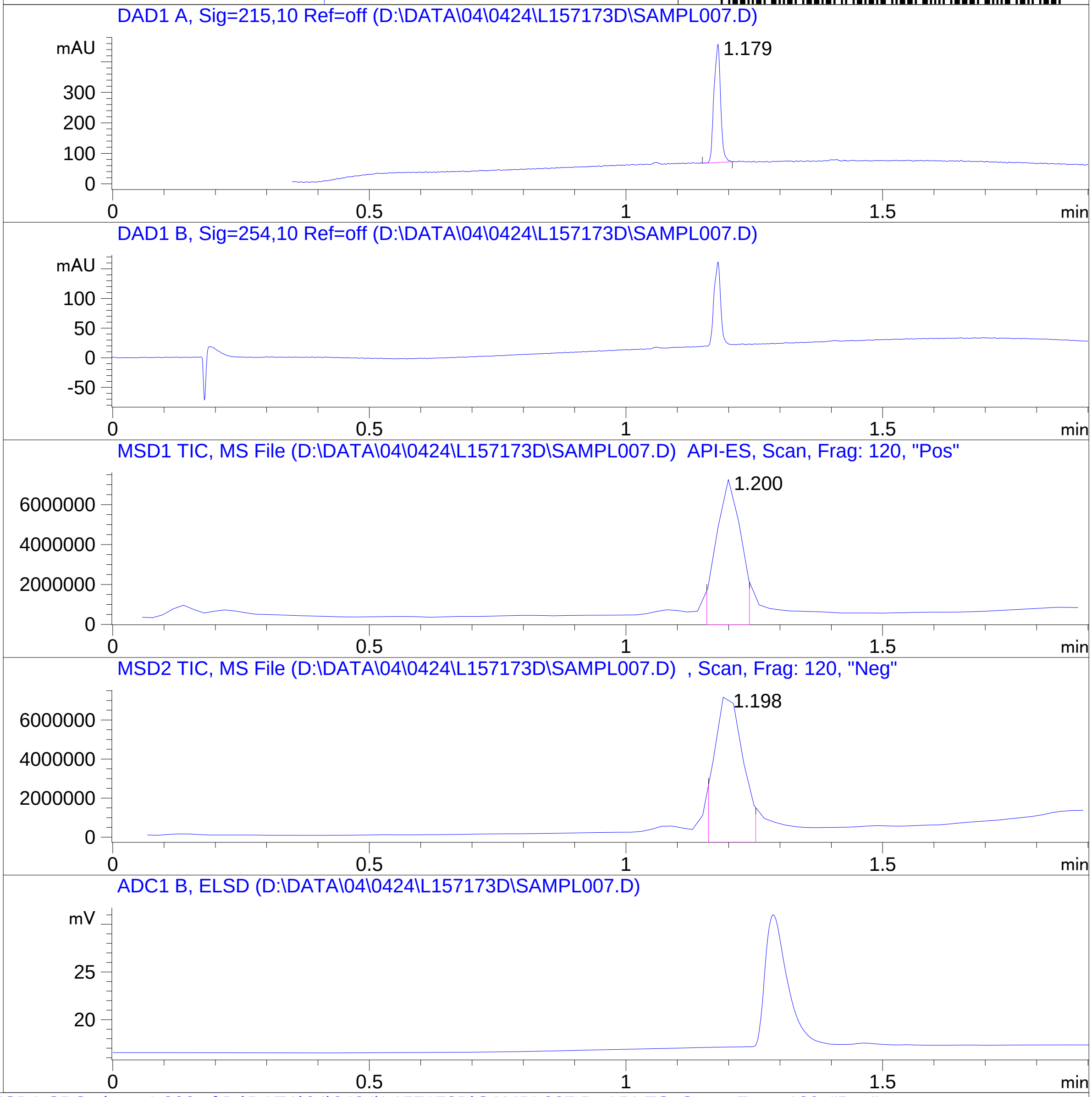

Mol Wt 0

Exact Mass

#	Time	Area%
1	1.179	100.00

RT 1.200

RT 1.198

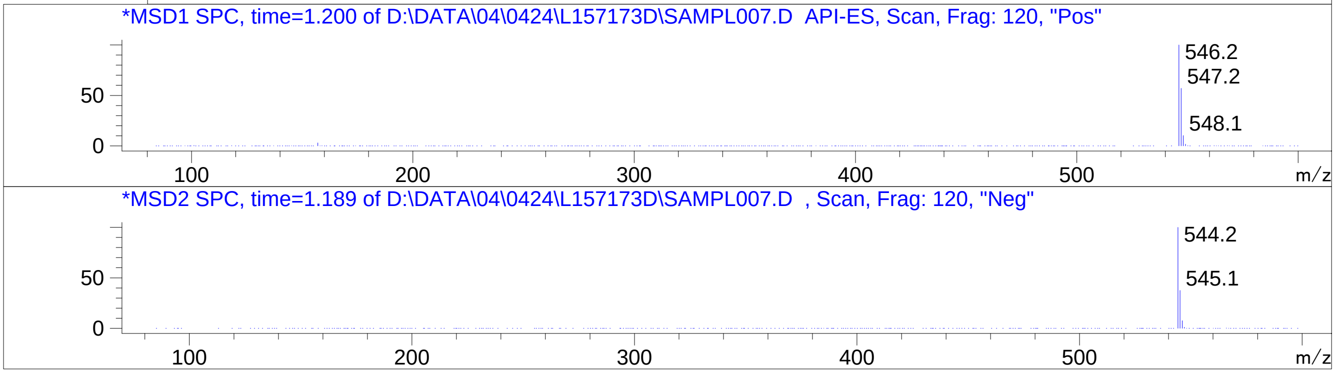

Inj.Date 4/24/2019 LB P2-A-07 S106 -SL- Acq. Method C:\HPCHEM\-> ->

IBOX23472

MaxPeak: 98.02%
Ret_Time: 1.233 min

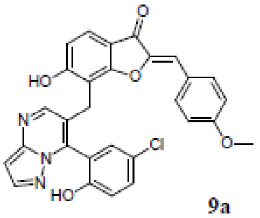

9a

Mol Wt 0

Exact Mass

#	Time	Area%
1	1.233	98.02
2	1.324	1.98

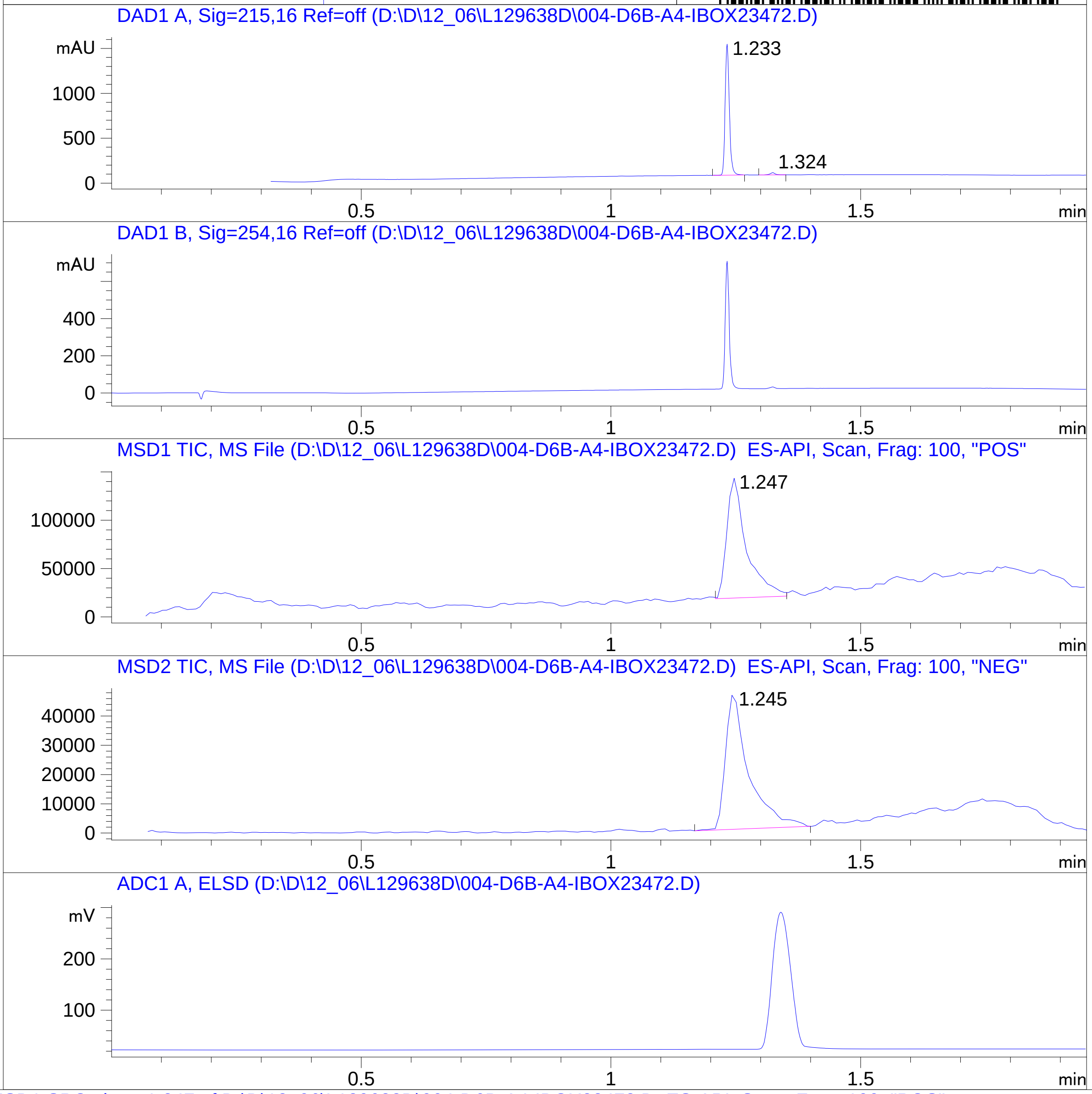

RT 1.247

RT 1.245

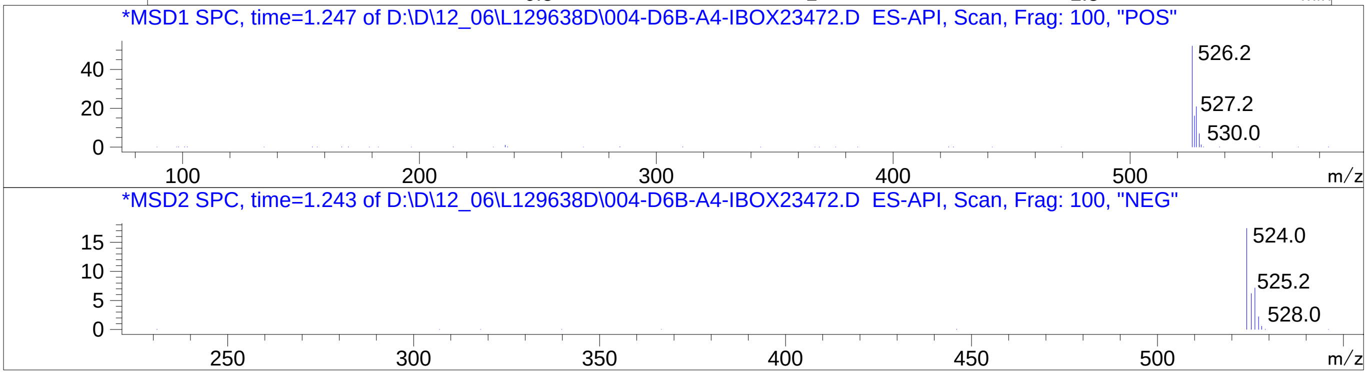

Inj.Date 12/5/2018 N <invalid> -> Acq. Method C:\Chem32\-> ->

MaxPeak: 93.79%
Ret_Time: 1.237 min

IBOX23866

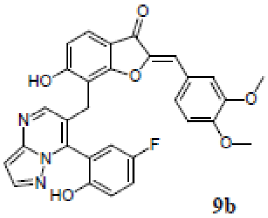

9b

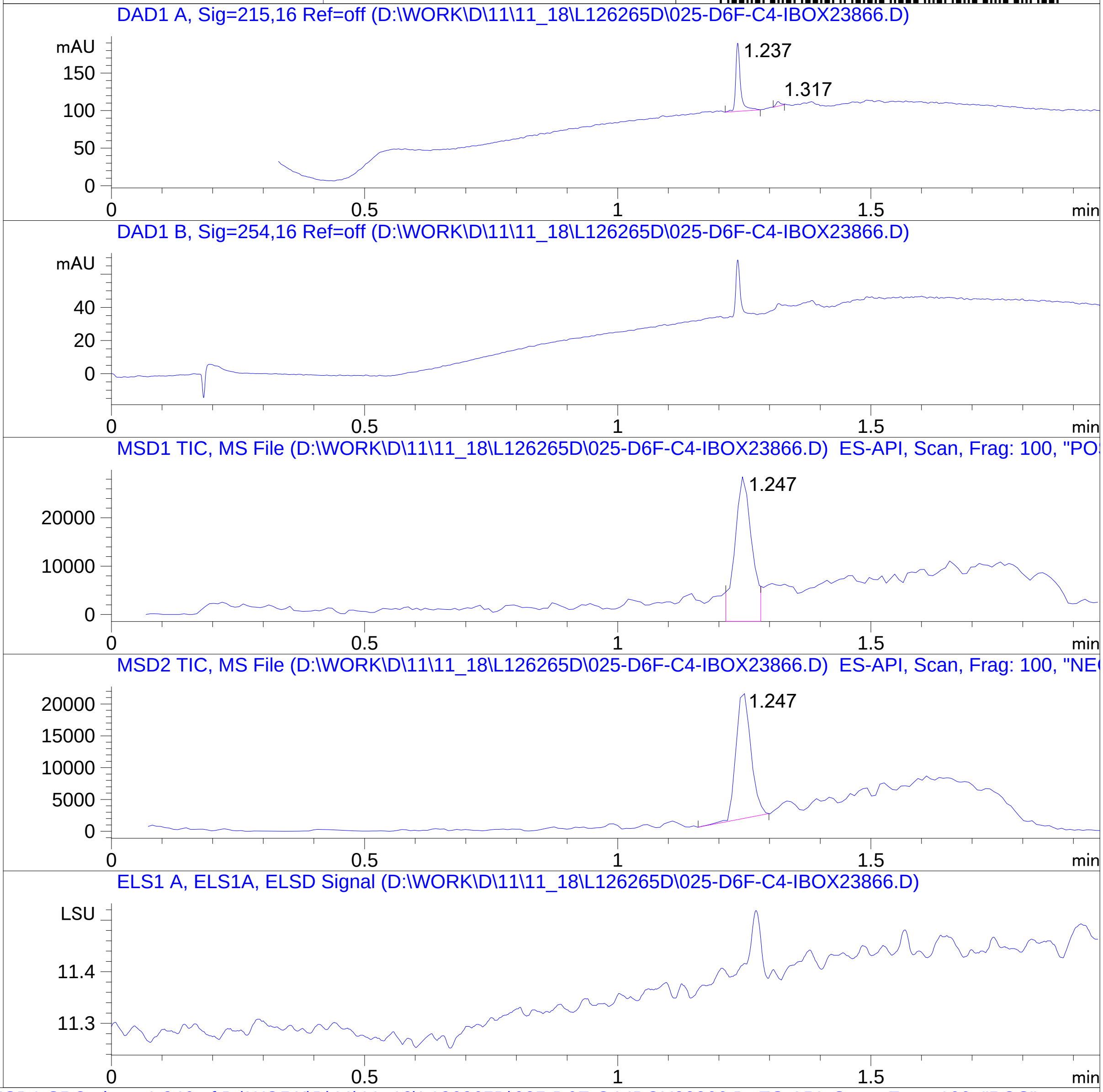

Mol Wt 0

Exact Mass

#	Time	Area%
1	1.237	93.79
2	1.317	6.21

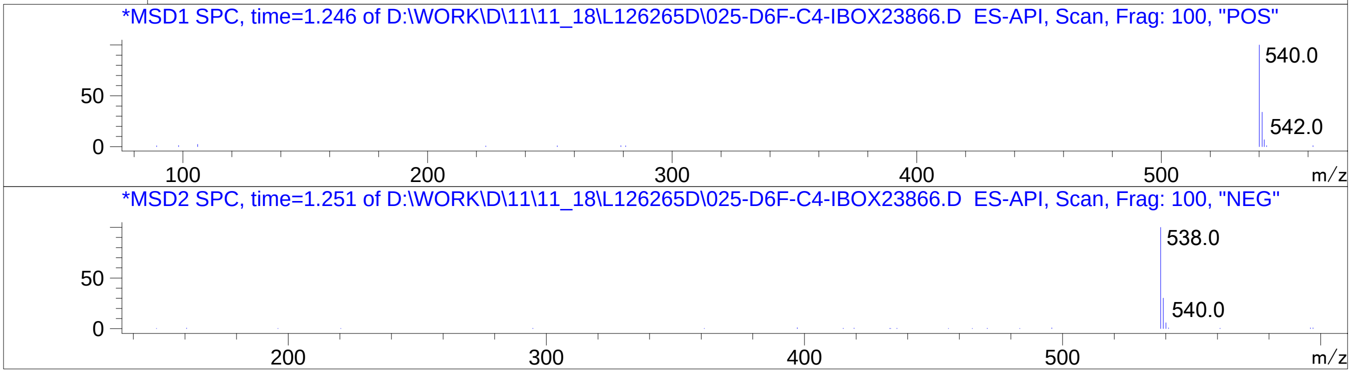

MaxPeak: 96.00%
Ret_Time: 1.243 min

IBOX22351

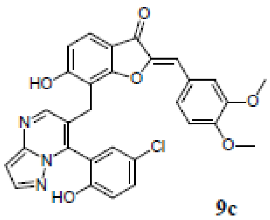

9c

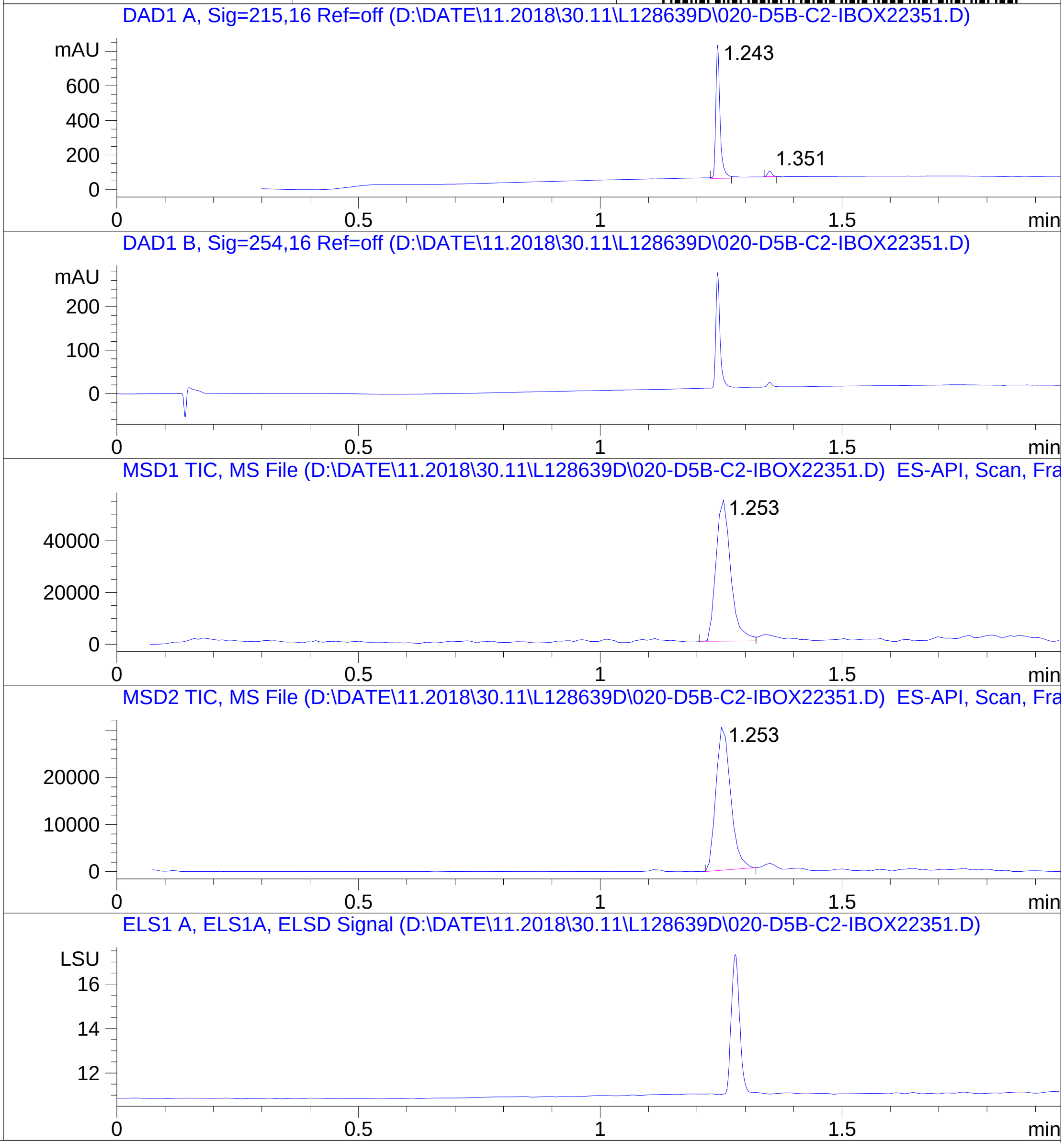

Mol Wt **0**

Exact Mass

#	Time	Area%
1	1.243	96.00
2	1.351	4.00

RT 1.253

RT 1.253

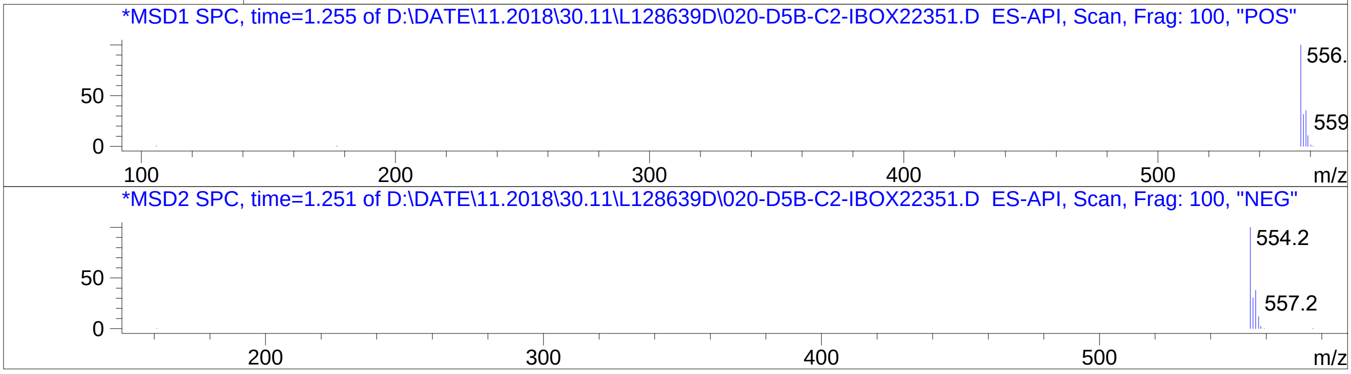

MaxPeak: 98.11%
Ret_Time: 1.292 min

IBOX22352

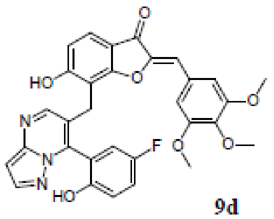

9d

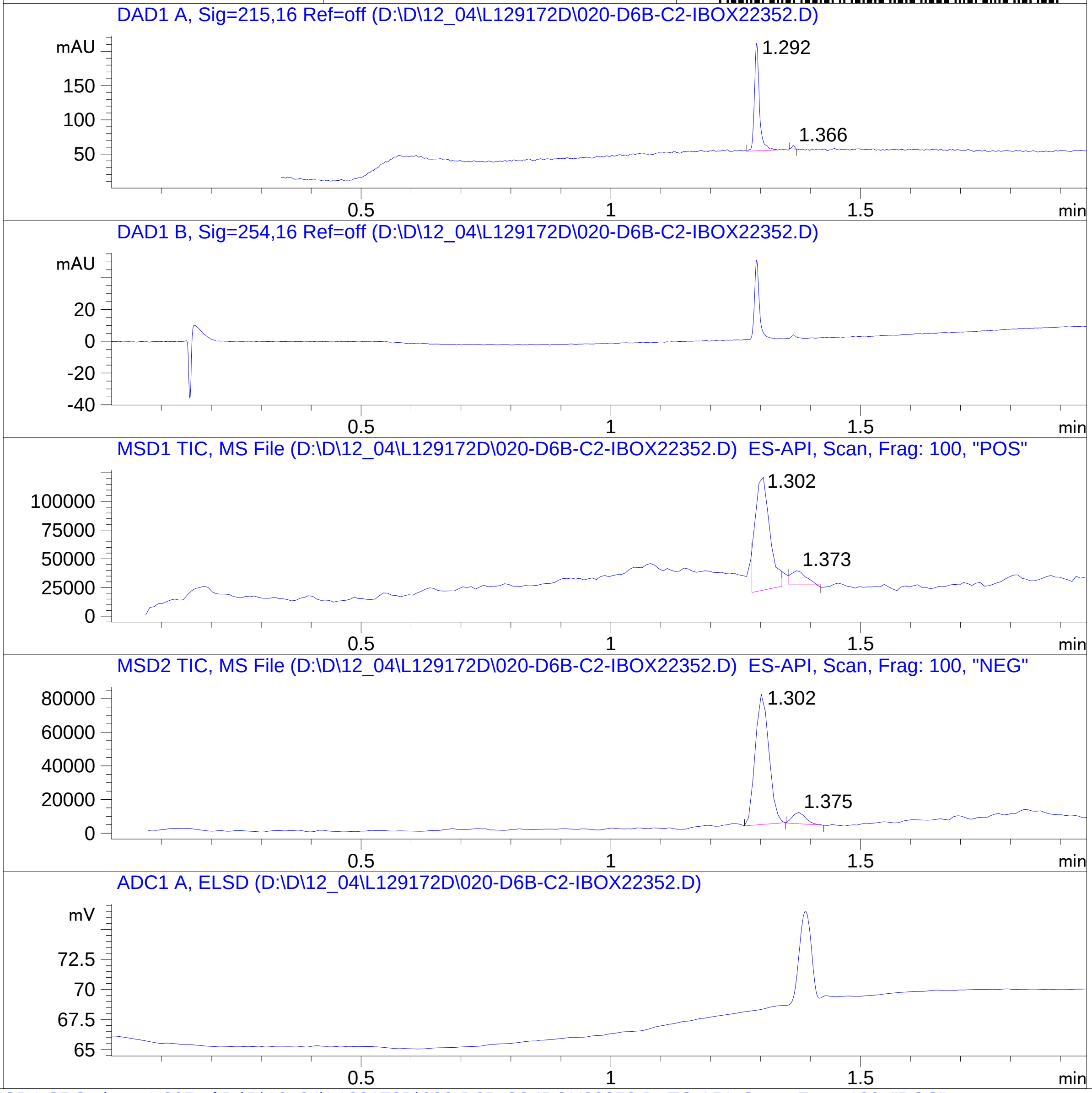

Mol Wt 0

Exact Mass

#	Time	Area%
1	1.292	98.11
2	1.366	1.89

RT 1.302

RT 1.373

RT 1.302

RT 1.375

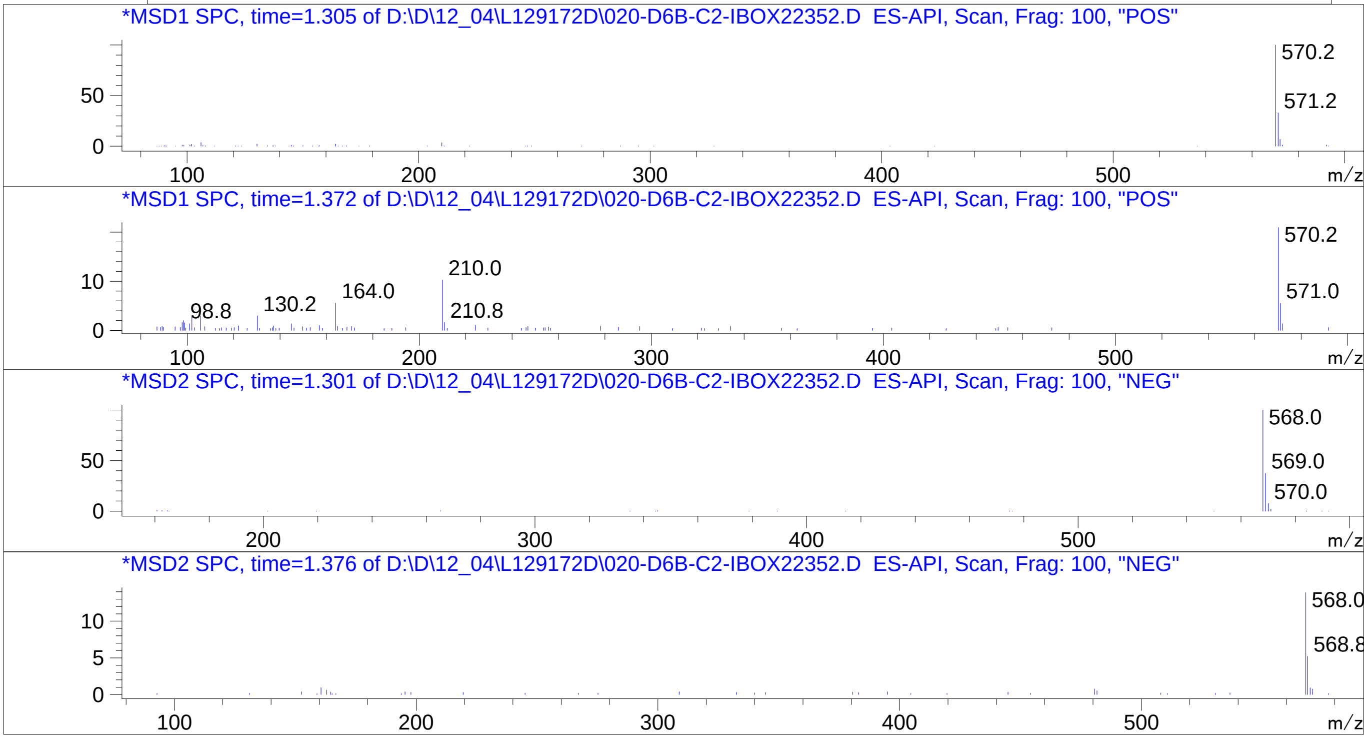

Inj.Date 12/4/2018 N <invalid> -8- Acq. Method C:\Chem32\-> ->
